# Binding Mechanism of the Active Form of Molnupiravir to RdRp of SARS-CoV-2 and Designing Potential Analogues: Insights from Molecular Dynamics Simulations

**DOI:** 10.1021/acsomega.4c05469

**Published:** 2024-09-24

**Authors:** Justin Carbone, Nicholas J. Paradis, Dylan Brunt, Chun Wu

**Affiliations:** College of Science and Mathematics, Rowan University, Glassboro, New Jersey 08028, United States

## Abstract

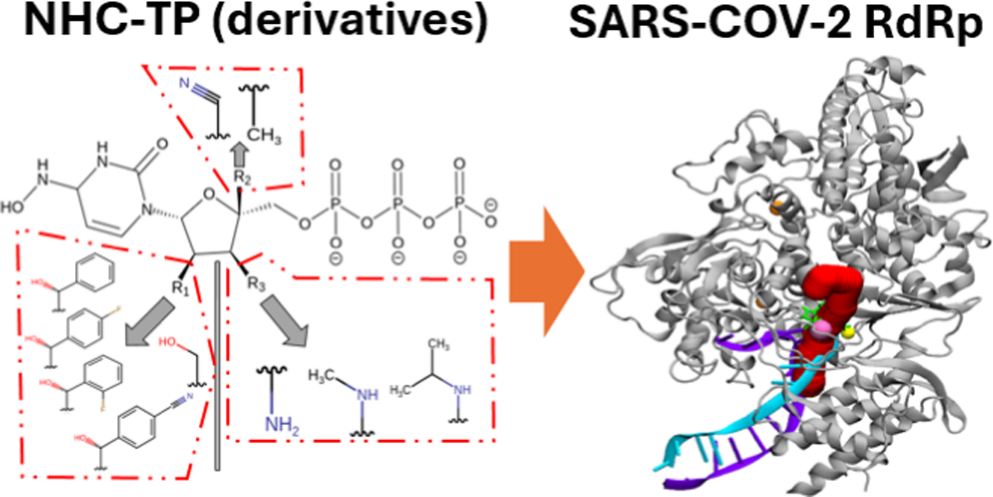

Molnupiravir, an FDA-approved nucleoside prodrug for treating COVID-19, converts into N4-hydroxycytidine triphosphate (NHC-TP), which integrates into SARS-CoV-2 RNA by its RNA-dependent RNA polymerase (RdRp) causing lethal mutations in viral proteins. Due to the risk of RdRp-mediated drug resistance and potential off-target effects on host polymerases (e.g., human polymerase II/HPolII), it is crucial to understand NHC-TP interactions at polymerase active sites for developing new, resistance-proof treatments. In this study, we used molecular dynamics (MD) simulations to probe key interactions between NHC-TP and SARS-CoV-2 RdRp and designed novel NHC-TP analogues with greater selectivity for SARS-CoV-2 RdRp over HPolII by a virtual screening workflow. We docked NHC-TP to a modified SARS-CoV-2 RdRp-Remdesivir triphosphate structure (PDB ID: 7BV2) and generated 71 NHC-TP analogues with bulky substituents to increase the interaction with RdRP and to reduce HPolII incorporation. MD simulations assessed the stability, binding affinity, and site interactions of these analogues. The top 7 candidates, with favorable ADMET properties, likely inhibit replication via potential dual mechanisms (the replicative stalling and the induction of lethal mutations) while maintaining selectivity for SARS-CoV-2 RdRp.

## 1 Introduction

**Figure 1:**
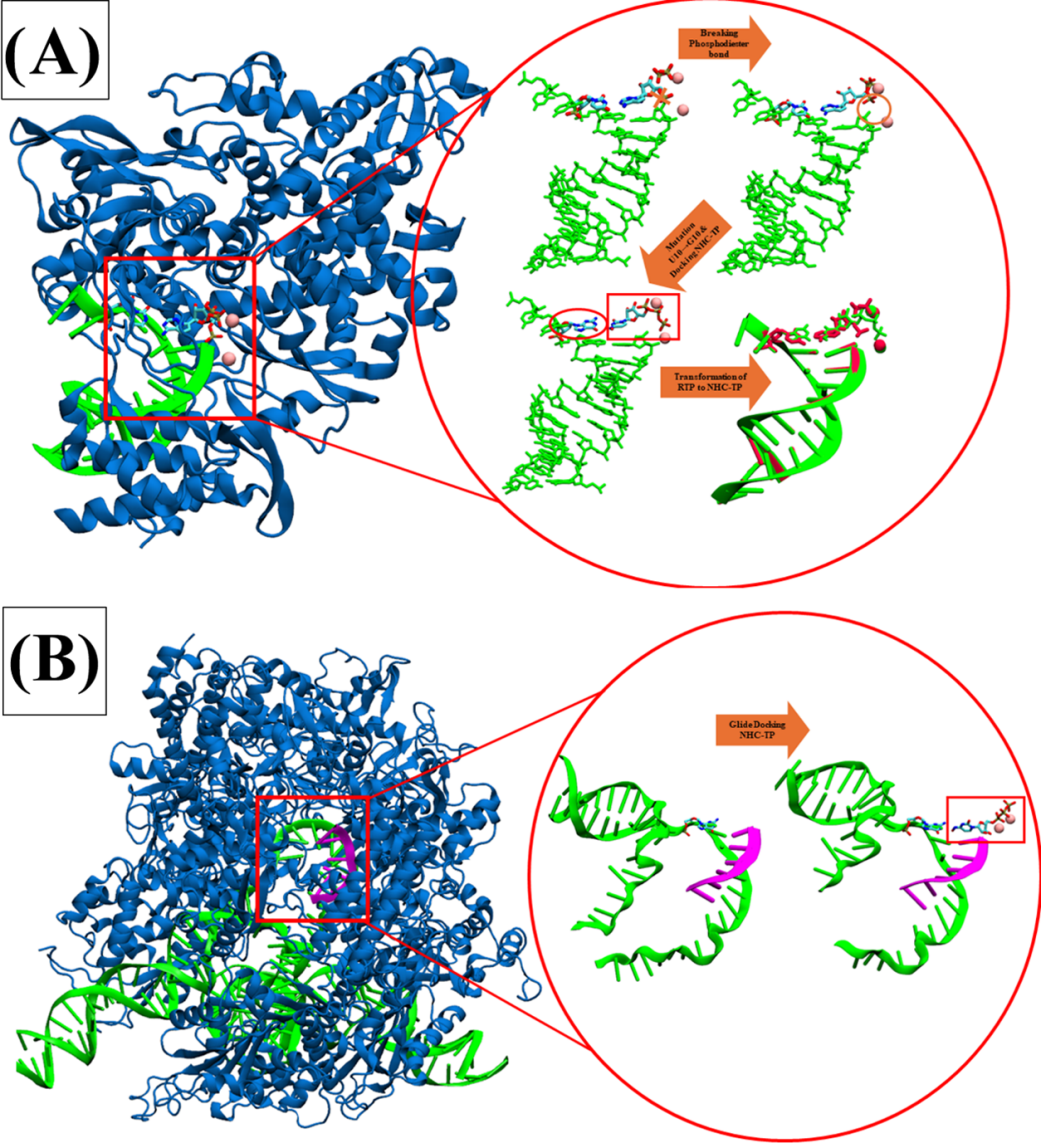
(A) Crystal structure of SARS-CoV-2 RdRp (PDB ID: 7BV2) (blue) in complex with template-primer RNA (green), covalently bound Remdesivir monophosphate (cyan), and Mg^2+^ ions (pink balls). Process of converting bound RDV to free-form RDV-triphosphate within the *i*-site shown by breaking the phosphodiester bond and the addition of diphosphate. (B) Crystal protein structure for HPolII (PDB ID: 5IYD) (blue) in complex with the RNA template and primer (green) and the product RNA strand (pink) and docked NHC-TP ligand (cyan) with Mg^2+^ ions (pink balls).

Within the past 4 years, the COVID-19 pandemic caused by SARS-CoV-2 has induced a considerable toll on human health and wellbeing. Increased immunization has prevented severe COVID-19 cases but the fast mutation rate of SARS-CoV-2 still poses an endemic threat for this virus.^1,2^ Inhibiting key enzymes crucial to the virus life cycle is a common method to prevent severe viral infection and symptoms; for SARS-COV-2, nonstructural protein 12 (nsp12, RNA-dependent RNA polymerase/RdRp) (Figure 1) is a critical protein responsible for replicating the virus genome. Like many RNA viruses, RdRp has a relatively conserved secondary structure, like that of Hepatitis C RdRp;^3^ upon cell infection, RdRp begins replication of the SARS-CoV-2 genome through hijacking free active nucleotides from host replication machinery. Traditionally, the inhibition of this enzyme has been approached by developing synthetic nucleoside analogues that mimic the standard ribonucleoside triphosphates through the NTP channel located near the palm subdomain.^4^ A nucleoside mimic operates by being incorporated into the growing RNA chain and can inhibit enzyme activity through delayed chain termination through active site interactions (Remdesivir/RDV)^5^ or by introducing sequential errors into the chain (molnupiravir). Though RDV was observed to treat severely infected COVID-19 patients, effective alternatives are needed due to the RDV-resistant strains causing ineffective inhibition of polymerization^6^ or removal via proofreading nsp14 (exonuclease).^7^ The results of this extra refinement could be the explanation of the increase in susceptibility of RdRp toward RDV in the absence of ExoN.^8^

Alternatively, molnupiravir (a.k.a., MK-4482, EIDD-2801) is another FDA-approved nsp12 inhibitor after successfully reducing the risk of hospitalization or death in patients by approximately 50% compared to a placebo, in a Phase III clinical trial.^9,10^ Furthermore, empirical and quantum mechanical studies of molnupiravir-treated cells suggest that molnupiravir works by inducing lethal mutations to the viral genome.^11^ Like other nucleotide inhibitors, molnupiravir’s active form, N4-hydroxycytidine triphosphate (NHC-TP) (Figure S1), binds to the catalytic site of SARS-COV-2 RdRp and is then incorporated into nascent RNA, inducing replicative errors within the viral sequence. Molnupiravir’s multiple tautomers promote the destructive mutation effect;^11^ hydroxylamine (CTP mimic) base-pairs with guanine, while its oxime form (UTP mimic) base-pairs with adenosine in the template strand.^8^ Unlike RDV, NHC-TP is less susceptible to drug-resistant mutations and proofreading rejection via exonuclease (ExoN) once incorporated into the viral genome.^12^ A recent study suggests that the hydroxylamine form, mimicking CTP, is the dominant tautomerization substrate *in vivo* with a higher concentration of the active substrate existing in hydroxylamine tautomerization but once incorporated into the RNA chain, both tautomerizations exist in equivalent concentrations.^12^ One such study performed a hypoxanthine phosphoribosyl transferase HPRT gene knockout assay in CHO-K1 cells to assess the genetic properties of NHC-TP, Favipiravir, and Ribavirin through loss of functions detected with 6-TG after 32 days.^8^ NHC-TP presented mutations in a dose-dependent manner up to 3 uM through the loss of HPRT function and resistance to the toxic base along with a high number of missense mutations in extracted colonies of the dNHC-TP metabolite.^8^ While mammalian proofreading exonuclease proteins are far more precise in the distinction of mispaired RNA/DNA, the addition of functional groups onto the 2′ position on the ribose portion could potentially stop the conversion of NHC-TP derivatives to their respective 2′-deoxy metabolite forms. Molnupiravir has also shown to be less effective in hospitalized patients^9^ due to the later stages of infection being unaffected by mutations.

**Figure 2:**
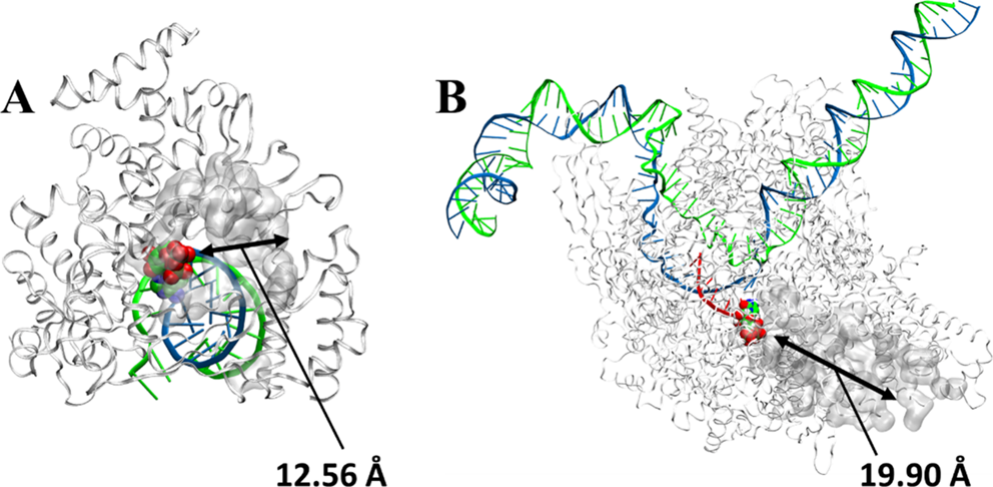
NTP entry channel analysis comparing SARS-CoV-2 RdRp with HPolII. (A) SARS-CoV-2 RdRp. (B) HPolII. Residues surrounding the NTP entry channel colored in white and bound NTP shown as VDW balls in elemental colors.

Nonetheless, nucleoside mimetics with a higher binding affinity than CTP and which ambiguously pair as CTP or UTP raise concern for the potential of host mammalian DNA incorporation via the conversion of NHC-TP into 2′-deoxyNHC-TP (dNHC-TP) by ribonucleotide reductase. Human RNA polymerase II (HPolII) is responsible for mRNA synthesis and RNA to DNA transcription and is relevant in many human genetic diseases promoted by unregulated transcription.^13^ When comparing the structures of SARS-CoV-2 RdRp and HPolII, the active site of nucleotide addition in HPolII is much larger and lies deep within the structure (Figures 1 and 2). HPolII also has two possible channels through which the NTP substrate can enter into the active site, with the secondary channel being most preferred.^13^ To reduce the introduction of nucleoside mimics into host mRNA, large bulky groups are added to NHC-TP analogues to present a better selectivity toward the viral RdRp enzymatic site and introduce steric hindrance into the active polymerization site of HPolII, creating a second line of defense for human proofreading machinery. While synthetic ribose additions are presumably not incorporated into NTPs,^14^ the molnupiravir base is still an active inhibitor. Along with the ligand size increase for each, ribose puckering has also been shown to play a key contribution to the selectivity between RNA and DNA polymerases.^14^ Multiple studies have shown that adding groups to C2′, C3′, and C4′ positions in the ribose sugar increases the selectivity toward viral polymerases via promoting RNA puckering of the C3′ endo form; moreover, human transcription polymerases will not incorporate synthetic sugars into product chains.^14^ Through the potential incorporation of these analogous and NHC-TP, we aim to disrupt SARS-COV-2 RdRp in a dual threat inhibition mechanism.

In this study, we utilize computer-aided drug discovery (CADD) methods to elucidate the binding of the active form NHC-TP to a prepared cryo-EM structure of SARS-CoV-2 RdRp (PDB ID: 7BV2)^15^ (Figure 1) along with 71 unique NHC-TP analogues with incorporations at C2′, C3′, and C4′ positions in the ribose moiety using combinatorial library enumeration (Tables S1, S3, and Figure S4) coupled with molecular docking and 200 ns molecular dynamics (MD) simulation across 58 independent systems (Table S4).

The goal of this study is to further elucidate the structure–activity relationships (SARs), binding behaviors, and stabilities of NHC-TP and its derivatives bound to SARS-COV-2 RdRp from MD simulations (Figures S8 and S11–S16). We then predicted the binding free energy of each ligand system to gain a better understanding of the relative binding affinity of NHC-TP and the analogues toward RdRp (Table S4 and Figure S15). Lastly, we computed the ADME property predictions of all ligands to determine their drug-likeness and toxicity profiles (Table S6). Seven top analogues were selected based on their competitive binding affinity relative to the NHC-TP substrate, favorable ADME properties, and interaction with critical active site residues, which may contribute toward novel inhibition mechanisms (Figure 5 and Table 3). Additionally, a brief analysis of the NTP entry of both SARS-CoV-2 RdRp and human RNA polymerase II (PDB: 5IYD)^16,17^ was performed to assess the binding potential of substituted NHC-TP derivatives in the effort to address the concern for mRNA-induced transcription errors within mammalian host cells due to their ambiguous binding as either CTP or UTP. The more preferred secondary channel within human polymerase^17^ is a deep channel within the enzyme, with a hydrophilic funnel-like opening at the surface elongating back to the catalytic center ∼20 Å deep and with a diameter of just ∼12 Å (Table 2, Figures 2 and 7).^18^ This is comparatively smaller and more sterically restrictive compared to SARS RdRp where the channel is very large and has very little hindrance for NTP entry. Our selected compounds present a better selectivity toward viral RdRp compared to HPolII while also exhibiting a better overall binding affinity than native NHC-TP for the potential treatment of SARS-CoV-2-induced COVID-19.

## 2 Experimental Section

### 2.1 Structure Building, Ligand Preparation, and Molecular Docking

The cryo-EM crystal structure of SARS-CoV-2 RdRp (nsp12 nsp7 nsp8) in complex with the template-primer RNA chain with the active form 3′ RDV covalently bound to the *i*-binding site was retrieved from the RCSB protein database (PDB ID: 7BV2)^15^ (Figure 1). We then selected just the protein chain (chain A) containing the catalytic site and template-primer RNA chains. The template strand was then modified at the corresponding base from uridine into guanine (U10G) through Maestro Schrodinger software^19^ for full NHC-TP hydroxylamine base pairing compatibility (Figure S2). The covalent bond holding 3′-configured Remdesivir monophosphate to the primer chain of U20 was broken and triphosphate was added to the 5′ carbon on the ribose portion to obtain free-form Remdesivir triphosphate (RTP) in the *i*-binding site. The SARS-COV-2 RdRp complex containing the new RNA template-primer was then preprocessed, optimized, and minimized using Maestro software’s built-in Protein Preparation Wizard^20^ to have the charge states optimized at pH = 7. Ligands were prepared by generating the ionization/tautomeric states at pH = 7 using Maestro’s built-in Epik tool^21−23^ and the OPLS3e force field assigned partial charges to all ligand atoms. A receptor docking grid was then generated in the space surrounding RTP. Area constraints were applied to the phosphate backbone of NHC-TP to be within 2.0 Å of Mg^2+^ ions embedded within the RdRp catalytic site (Figure 1).

### 2.2 Combinatorial Ligand Library Construction and Molecular Docking

**Figure 3:**
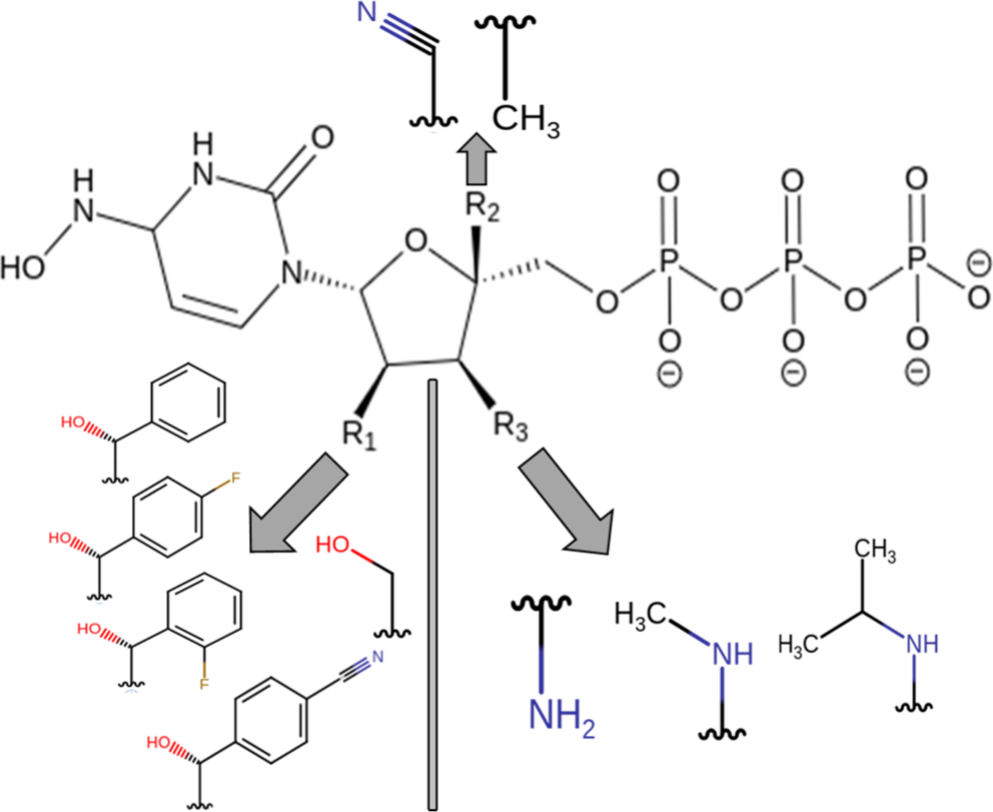
Substitutional groups selected for combinatorial library generation on sites R1–2′, R2–4′, and R3–3′ of NHC-TP.

We generated a combinatorial library of NHC-TP analogues using the R-Group creator module within Maestro software.^19^ The list of substitutional fragments on designated sites R1, R2, and R3 within the ribose sugar group is represented (Figure 3). Here, the enumerated addition of halogenated and cyanomethoxy aryl fragments is made on the 2′ position of the ribose ring. The 4′ position allowed for the addition of both methyl and cyanomethyl function groups. Lastly, the 3′ position allowed for the addition of increasing methylated amine functional groups (Figure 3, Tables S1 and S3). Using the R-group enumeration library creator within Maestro software, a library of 71 unique molnupiravir-substituted analogues was created in their active triphosphate forms. Out of the 71 ligands, nonsubstituted NHC-TP was included and used as the reference ligand for a total of 72 substrates. The SMILES codes for the two-dimensional (2D) chemical structure of the 72 ligands have been tabulated (Table S2).

The library containing the 72 ligands was docked to the NHC-TP binding site within SARS-COV-2 RdRp using the Glide SP scoring function within Maestro software^22,24^ that has been evaluated on multiple data sets.^25^ The protein structure for HPolII (PDB ID: 5IYD)^16^ was also prepared through the Schrodinger Preparation workflow. Then, the homology model of the RPB2 protein (UniprotID: P30876) was constructed using the Prime Homology Modeling tool from Schrodinger Suites.^19^ Using binding site detection, the NTP *i* + 1 addition site was analyzed and used to build a receptor grid box for Glide SP docking^24^ (Figure 1). The receptor docking grid was generated from the free-form mode nucleotide (RTP or NHC-TP). Additionally, a constrained Glide SP docking protocol was used for all 72 enumerate ligands to the SARS-COV-2 RdRp active site, where strict area constraints were set to the phosphate backbone and Mg ions to ensure correct positions of nucleotide addition to adjacent base pairing. Without these constraints, the docked pose represented a variety of biologically incorrect poses, incorrect base pairing, and a non C3′ endo pucker angle^14^ for nucleotide addition, causing a much lower binding affinity (Δ*G*_TOT_). The enumerate library was also docked to HPolII utilizing the same methodology as that of SARS-COV-2 RdRp, with distance constraints set to the centroid of Mg ions and the phosphate group of NTP ligands to the active site of HPolII. The SP docking scores for the 72 ligands on SARS-COV-2 and HPolII (Tables 1 and S3) and the ligand–protein interaction diagrams for the docked poses on SARS-COV-2 RdRp (Figure S7) have been generated.

**Table 1:** Ligand Size (Radius of Gyration/rGyr), Pre-MD and Glide SP Docking Scores, the Individual Energetic Terms for MM-GBSA Binding Free Energy, Average Protein and Ligand Root-Mean-Square Deviation (RMSD) and R-Group Substituents for NHC-TP (Reference Ligand) and the Top 7 NHC-TP Selected Compound Systems^a^

ligand	ligand size (radius of gyration)	7BV2 pre-MD Δ*G*_TOT_ (kcal/mol)	5IYD glide SP Δ*G*_TOT_ kcal/mol	Δ*E*_VDW_ (kcal/mol)	Δ*E*_ELE_ (kcal/mol)	Δ*E*_HYD_ (kcal/mol)	Δ*G*_TOT_ (kcal/mol)	avg. prot. RMSD (Å)	avg. lig. RMSD (Å)	R1	R2	R3
NHC-TP	5.010	–8.75	–8.43	–373.3 ± 62	125.7 ± 25	–97.6 ± 20	–345.3 ± 75	2.12	2.45			
E:03	4.711A	–9.31	–9.23	–384.6 ± 28	64.1 ± 11	–112.7 ± 7	–433.2 ± 28	2.05	1.95	(–C_7_H_7_O)	(–CH_3_)	(–NH_2_)
E:63	5.229A	–9.53	–8.62	–384.9 ± 16	85.1 ± 22	–108.4 ± 4.9	–408.1 ± 25	2.00	1.27		(–CH_3_)	(–NH_2_)
E:12	5.304A	–9.60	–9.26	–368.6 ± 22	78.0 ± 23	–105.7 ± 3.6	–396.4 ± 22	2.49	2.9	(–C_7_H_7_O)	(–CH_3_)	
E:66	4.856A	–9.42	–8.2	–416.1 ± 12	150.6 ± 24	–107.7 ± 3.4	–373.2 ± 28	2.64	1.68		(–CH_3_)	(–CH_3_NH)
E:65	5.142	–9.84	–9.30	389.1 ± 19	132.2 ± 16	–106.9 ± 4.4	–363.8 ± 16	2.07	2.21			(–CH_3_NH)
E:53	4.894A	–8.00	–8.62	–384.6 ± 33	133.1 ± 18	–104.1 ± 12	–355.6 ± 50	2.27	1.46	(–CH_2_OH)		(–CH_3_NH)
E:48	4.707A	–9.62	–9.30	372.9 ± 13	125.2 ± 11	–104.6 ± 4.1	–352.3 ± 20	1.82	1.61	(*o*-C_7_H_6_FO)	(–CH_3_)	

a Summary of docking scores from the pre-MD simulation (SP docking scores) to SARS-COV-2 RdRp (PDB ID: 7BV2) and Human Pol II (HPolII) (PDB ID: 5IYD), post-MD simulation binding free energy calculation (MM-GBSA), and average RMSD values were obtained from the last 50 ns of simulation.

### 2.3 Molecular Dynamics (MD) System Setup

The MD simulation system was created for each of the selected 58 ligands in complex with RdRp. The systems were generated with the addition of a 0.15 M NaCl salt concentration to neutralize the systems using the Desmond system builder.^26^ They were then placed into an orthorhombic water box with a buffer distance of 10 Å using the SPC water model.^27^ All system setup runs were performed under the broad-range OPLS3e^28^ force field to describe the parameters for each protein, ligand, ionic, and nucleic components. The OPLS3e force field parameters for NHC-TP and the top 7 NHC-TP analogues selected from our workflow are represented in Figure S17a–h in the mae file format.

### 2.4 Relaxation and Production MD Runs

Each MD system was relaxed using the default relaxation protocol for nonmembrane proteins utilizing the Desmond module within Maestro software.^26^ The protocol consists of eight stages: (1) system minimization with a restraint given to solute heavy atoms; (2) system minimization with no applied restraints; (3) system simulation with applied heating from 0 to 300 K, water barrier, and gradual restraining; (4) simulation under the NPT ensemble which is defined as a constant number of particles, a constant pressure of 1 bar, and a constant temperature of 300 K with the water barrier and heavy atoms being restrained; (5) simulation under the NPT ensemble with the equilibration solvent; (6) simulation under the NPT ensemble with protein heavy atoms annealing from 10.0 to 2.0 kcal/mol; (7) simulation under the NPT ensemble with Cα atoms restrained at 2.0 kcal/mol; and (8) simulation of 1.5 ns under the NPT ensemble with no restraints, followed by 58 production runs being run under the NPT ensemble using the default protocols with a duration of 200 ns for each complex at the PSC Bridges-2 GPU cluster.

### 2.5 Conformational Stability, Ligand Properties, Protein–Ligand Contacts, and Post-MD MM-GBSA Predictions

The Desmond Simulation Interaction Diagram (SID) tool^26^ was utilized to analyze the overall conformational stability and convergence of each SARS-COV-2 RdRp–ligand complex during the duration of each production run. The root-mean-square deviation (RMSD) of both the protein and the ligand (Figures 6Aand S9) was calculated to check the convergence of the SARS-COV-2 RdRp active site along with the ligand’s deviation from its initial docked pose (Figure S8). The root-mean-square fluctuation (RMSF) was analyzed for the SARS-COV-2 RdRp protein backbone (Figure S10), ligand heavy atoms (Figures 6Dand S13) to check the ligand stability within the SARS-COV-2 RdRp active site and O5′ RNA atoms (Figure 6B,6C). Ligand properties for the top 7 ligands (i.e., ligand RMSD, radius of hydration, intramolecular hydrogen bond count, molecular surface area, solvent-accessible surface area, and polar surface area) were also generated (Figure S12). Additionally, protein–ligand contacts (H-bond and ionic and hydrophobic interactions) were examined in 2D protein–ligand contact interaction diagrams (Figure S11) and as histograms (Figure S14).

The molecular mechanics/generalized Born surface area (MM-GBSA) energy was then obtained on each of the 58 SARS-COV-2 RdRp–ligand complexes to determine the potential structural stability and energetic improvement before MD simulation (after SP docking) and after MD simulation (last simulation step, at 200 ns) (Table S4). The OPLS3 force field,^28,29^ VSGB 2.0 solvation model,^26^ and default Prime procedure was used for the calculations. The default procedure consists of three steps: receptor-alone minimization, ligand-alone minimization, and receptor–ligand complex minimization. The total binding energy (Δ*G*_TOT_) equation is
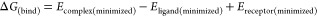
To gain a more detailed understanding of the binding nature, the original interaction terms (Coulombic + H-bond + GB solvation + van der Waals + π–π packing + self-contact + lipophilic) were merged into three components: *E*_electrostatics_, *E*_vdW_, and *E*_lipophilic_, where
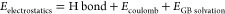

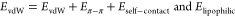
The Δ*G*_TOT_ values are calculated for the 58 SARS-COV-2 RdRp–ligand complexes (Table S4) The Δ*G*_TOT_ was calculated with the assumption that entropic terms for each ligand are similar due to the similarity of binding sites.^30^

### 2.6 Structure Clustering Analysis

Utilizing the Desmond trajectory clustering tool within Maestro software,^26^ the snapshots of the last 100 ns of simulation from the top seven ligands were grouped using a RMSD distance measure. The distance cutoff for merging was set to be 2.5 Å. The structure having the largest number of neighbors in the structural family otherwise known as the centroid structure was used to represent the structural family. The clusters with the most occupancy represent the most abundant conformations seen during each simulation (Figures 8 and S8).

### 2.7 NTP Entry Channel Analysis

Channel analysis on the most abundant cluster of the SARS-COV-2 RdRp complex from MD was analyzed using Caver 3.0^31^ and visualized using Pymol.^32^ Channel bottlenecks and lengths are tabulated in Table 2, showing differences between calculated tunnels of SARS-COV-2 RdRp and HPolII. The SARS-COV-2 RdRp channels were calculated from the centroid ligand for each system with default settings other than the shell depth increase from 0.9 to 1.5 and the max length set to 10 Å (Figures 7 and S5). Channels were also analyzed for the HPolII structure with the centroid as the starting point for calculation, and the max length was increased to 25 Å due to the size of the molecule to calculate all possible channels (Figures 7 and S6).

**Table 2:** NTP Entry Channel Properties of SARS-COV-2 RdRp and HPolII from Caver Analysis^a^

ligand	RdRp bottleneck radius (Å)	RdRp tunnel length (Å)	HPol2 bottleneck radius (Å)	HPol2 tunnel length (Å)	RdRp lining residues
E:71 (NHC-TP)	3.9	43.5	3.1	37.4	553, 798, 555, 760, 761, 618, 621, 619, A:811, 622, 623, 545, 620
E:03	2.4	17.9	3.1	36.9	551, 798, 553, 623, 618, 621, 555, 620, 619
E:63	1.8	26.1	3.3	44.9	451, 455, 452, 553, 448, 621, 453, 454, 450
E:12	3.2	18.6	3.1	36.9	760, 555, 551, 798, 761, 618, 553, 623
E:66	2.6	12.2	3.1	44.9	553, 551, 552, 444, 549, 554, 550
E:65	2.5	42.2	3.1	36.5	618, 555, 551, 760, 798, 619, 761, 620, 553, 621, 622
E:53	2.5	19.7	3.1	44.9	623, 760, 553, 555, 761, 618, 624, 620, 621, 619, 622, 691
E:48	1.7	36.1	3.4	31.5	553, 455, 621, 452, 552, 624

a Calculated tunnels are done through Methods section 2.7. Comparison of RdRP 7BV2 and Human Pol2 5IYD and lining residues of the RdRP channel.

### 2.8 ADMET Property Predictions

The absorption, distribution, metabolism, excretion, and toxicity properties of all 58 ligands and RDV were calculated using the SwissADME Web server.^33^ The NHC-TP derivatives were evaluated using their respective prodrug forms relative to molnupiravir (Tables 3 and S6). RDV was used as the comparison for analysis in selecting superior derivatives. Properties tabulated for drug adsorption and bioavailability were Consensus LogP (+LogP: lipophilic; −LogP: hydrophilic, where decreasing LogP/lipophilicity is more favorable for drug-likeness, less sequestration, and systemic toxicity by fatty tissue and more easily penetrate necessary biological barriers to reach the intended target), LogS (ESOL, Ali, and Silicos-IT, where increasing LogS indicates increased water solubility), and GI absorption (critical for ensuring bioavailability). Inhibition of several CYP enzymes (CYP1A2, CPY2C19, CYP2C9, CYP2D6, and CYP3A4), Lipinski ruling, and synthetic accessibility scoring were tabulated (Tables 3 and S6).

**Table 3:** ADMET Property Summary of the Top 7 NHC-TP Analogues in the Prodrug Form with RDV and NHC-TP as the Reference^a^

ligand	consensus LogP	ESOL LogS	Ali LogS	silicos-IT LogSw	avg LogS	GI absorption	CYP1A2	CYP 2C19	CYP2C9	CYP2D6	CYP3A4	Lipinski violations	synthetic accessibility
RDV	1.53	–4.12	–6.01	–4.77	–4.97	low	no	no	no	no	yes	no; 2 violations: MW > 500, NorO > 10	6.33
E:03	0.95	–2.63	–3.25	–3.19	–3.02	low	no	no	no	no	no	yes; 0 violation	5.16
E:71 (NHC-TP)	–1.08	–0.83	–1.17	0.12	–0.63	low	no	no	no	no	no	yes; 0 violation	4.49
E:63	–0.69	–0.67	–0.89	–0.69	–0.75	low	no	no	no	no	no	yes; 0 violation	4.73
E:12	0.83	–2.79	–3.39	–2.97	–3.05	low	no	no	no	no	no	yes; 0 violation	5.04
E:66	–0.24	–1.00	–1.13	–1.48	–1.20	low	no	no	no	no	no	yes; 0 violation	4.81
E:65	–0.58	–1.00	–1.25	–0.89	–1.05	low	no	no	no	no	no	yes; 0 violation	4.69
E:53	–0.40	–1.28	–1.70	–1.28	–1.42	low	no	no	no	no	no	yes; 0 violation	4.77
E:48	1.25	–2.96	–3.49	–3.23	–3.23	low	no	no	no	no	no	yes; 0 violation	5.08

a Consensus LogP: average of the five lipophilicity scores of Log *P*_o/w_ (iLOGP, XLOGP3, WLOGP, MLOGP, SILICOS-IT); avg. LogS: average of the three water solubility scores (ESOL LogS, Ali LogS, LogSW); and CYP: inhibition character of CYP enzymes.

## 3 Results and Discussion

### 3.1 Enzyme Entry Channel Analysis and Ligand Library Creation

The entry channels of HPolII and SARS-COV-2 RdRp were analyzed to elucidate the incorporation mechanism of NHC-TP and their analogues (Figures 2, 7, S5, S6 and Table 2).

The secondary entry channel for HPolII is the path where NTP molecules enter the catalytic center one at a time, leading to the incorporation, elongation, and ejection of diphosphate (PPi).^34^ Looking at the crystal structure of HPolII, the opening has a funnel-like shape that extends approximately ∼19.90 Å toward the center. Some studies have explained the kinetics of NTP incorporation. Martinez-Rucobo and Cramer conducted a thorough review on HPolII transcription elongation, where NTP incorporation and fidelity processes will be highlighted here as a two-step mechanism. After NTP has been positioned for incorporation in the HPolII active site (open inactive state), NTP promotes folding of the trigger loop on NTP, moving NTP into the insertion site. NTP then makes contacts with several species within the active site, including hydrogen bonding with the template nucleobase, the trigger loop to promote its closing and stabilization of the NTP substrate in the active site, coordination with two Mg^2+^ cofactors at its triphosphate tail, and aspartate active site residues to mediate triphosphate cleavage and release of PPi; this combination of interactions promotes transition of the open inactive state to the closed active state, facilitating nucleoside addition.^35^ HPolII also utilizes a rigorous proofreading mechanism to prevent the incorporation of incorrect nucleobases; the instability arising from improper base matching between noncognate/incorrect complementary nucleobases with the template nucleobase can trigger backward translocation by HPolII, causing endonucleolysis of the scissile phosphodiester bond of the misincorporated nucleobase and its excision, helping to maintain fidelity in transcribed RNA.^35^ Moreover, while the incorporation of NTP via HPolII is more readily understood through biochemical and DFT experiments,^36^ the kinetics of the HPolII incorporation of NHC-TP and their derivatives are still unclear. In contrast, SARS-COV-2 RdRp does not have such a secluded active site, exhibiting a broader opening and shallow active site (Figures 1, 2, 7, and Table 2). Additionally, while SARS-COV-2 RdRp works in tandem with nsp14 (proofreading exoribonuclease) to excise misincorporated nucleosides and mimics; incorporated NHC-TP base-pairs readily with guanosine and adenosine nucleobases and allows the incorporation of incoming cognate nucleotides, thus evading excision.^11^ Nonetheless, building NHC-TP derivatives which include larger substituents on the ribose portion could possibly reduce the incorporation into HPolII while introducing selectivity toward SARS-COV-2 RdRp.

The combinatorial ligand library of 72 NHC-TP analogues including the native NHC-TC substrate using the subset of fragments applied to three substitutional sites (R1, R2, R3) on the ribose sugar was generated (Figure 3, Tables S1 and S3).

The substitutional site groups used for each site were determined by observing the available physical space surrounding the ribose portion of NHC-TP within the active site of SARS-COV-2 RdRp (Table 2). The availability of various polar and positively and negatively charged residues within a 6.0 Å radius of the *i*-binding site near the inner palm subdomain indicated the potential for critical and interesting ligand contacts to occur.^5,15,37,38^ The substitutional site on the C2′ (R1) position could establish new residue contacts with nearby (∼3.4 Å) residues R555, S682, Y456, and T687. Due to the space between these residues and the C2′ hydroxyl group seen on NHC-TP, we determined that the addition of halogen- and cyano-substituted phenyl ethanol groups would be appropriate due to both the hydrophobic bulk and hydrogen-bonding potentiality. The C4′ (R2) position of the ribose portion was selected to resemble a similar binding mechanism for RTP and for its close proximity (∼3.6 Å) to residues A688, N691, and S759. The addition of methyl and cyanomethyl substitution is proposed to both introduce new residue contacts and help add hydrophobic and van der Waals interactions, which contribute stability to the scaffold. Lastly, in the C3′ (R3) position of the ribose sugar and C3′ hydroxyl, we observed C622 and D623 nearby (∼4.6 Å). With the understanding of the active site region of polymerase displaying a variety of essential interactions for nucleotide addition, we selected amine, methyl, and isopropylamine groups to be appropriate for substitution to retain reactivity with the SARS-COV-2 RdRp active site (Figure 3).

### 3.2 Glide SP Docking to SARS-COV-2 RdRp and HPolII

Due to the rising concern surrounding the efficacy and mutagenicity of NTP inhibitors due to host genome incorporation by human replication machinery, the enumerate library was docked to both SARS-COV-2 RdRp and HPolII to examine the ligand selectivity profile. The Glide SP docking scores have been tabulated (Tables 1, S3 and S4) and the docking poses within the SARS-COV-2 RdRp active site are displayed as 2D interaction diagrams (Figure S8).

The standard NHC-TP substrate exhibited a docking score of −8.7 kcal/mol. Please note that molecular docking scores are considered very rough approximations of the binding free energy, which is why MD simulations and post-MD MM-GBSA binding free energy calculations are performed later. Both RDV-TP and NHC-TP demonstrated very similar initial poses with both nucleic portions, forming hydrogen pairing with their corresponding template nucleobase, and their triphosphate tails oriented toward the NTP channel near positively charged stabilizing Mg^2+^ ions (Figures 1, 7, S2 and S7). Crucially, the conformation of NHC-TP positioned the ribose ring angles of 2′ and 3′ hydroxyl stereocenters facing toward the inner palm subdomain.^5,15,37,38^ NHC-TP formed hydrogen bonds with R555 in addition to salt bridge interactions of the triphosphate tail with both nearby Mg^2+^ ions. This is seen in the preliminary docking of RTP to SARS-CoV-2 RdRp in previous studies.^38^ Additionally, the orientation of the triphosphate tails toward both Mg^2+^ ions is a crucial determinant for promoting endonucleolytic cleavage of the phosphodiester bonds to incorporate NHC and for initiating the release of PPi in coronavirus RdRp.^39^

Out of the 58 docked ligands, 37 ligands in the enumerate library exhibited more favorable docking scores than NHC-TP to HPolII, while the remaining 23 ligands exhibited more favorable ΔG* for SARS-COV-2 RdRp over HPolII (Table S4). The average difference in docking scores was calculated through ΔG* HPolII-ΔG* SARS-COV-2 RdRp, where the energetic preference was being shown as the average docking score ΔG*. The average ΔG* across the 58 docked ligands presented the preference in HPolII (+ΔG* values), where docking scores were slightly more favorable than RdRp with the overall average preferring HPolII at −0.1 kcal/mol (Table S4). Steric hindrance within the shallow binding site of SARS-COV-2 RdRp and large polar substituents are probable causes for the unsuccessful docking of the remaining 14 NHC-TP ligands (marked as X in Table S4) along with harsh constraints added to preserve correct base pairing, causing difficult pose incorporation in SARS-COV-2 RdRp. HPolII did not experience these same difficulties as the binding site is much larger, but constraints were also still applied to hold correct poses. The force field applied in docking, OPLS3e, is also a generic, wide-range force field when presented with large negative and positive charge interactions in the system of RNA, phosphate groups, Mg^2+^ ions, and charged residues. Hence, docking poses can become less realistic due the simplicity of the force field and the focus on free energy. To reduce the binding of ligands to HPolII, we picked the top 7 ligands with more favorable docking scores to SARS-COV-2 RdRp by ∼−0.7 kcal/mol (Tables 1, S4, and Figure 1).

### 3.3 Selection of the Top NHC-TP Candidates

**Figure 4:**
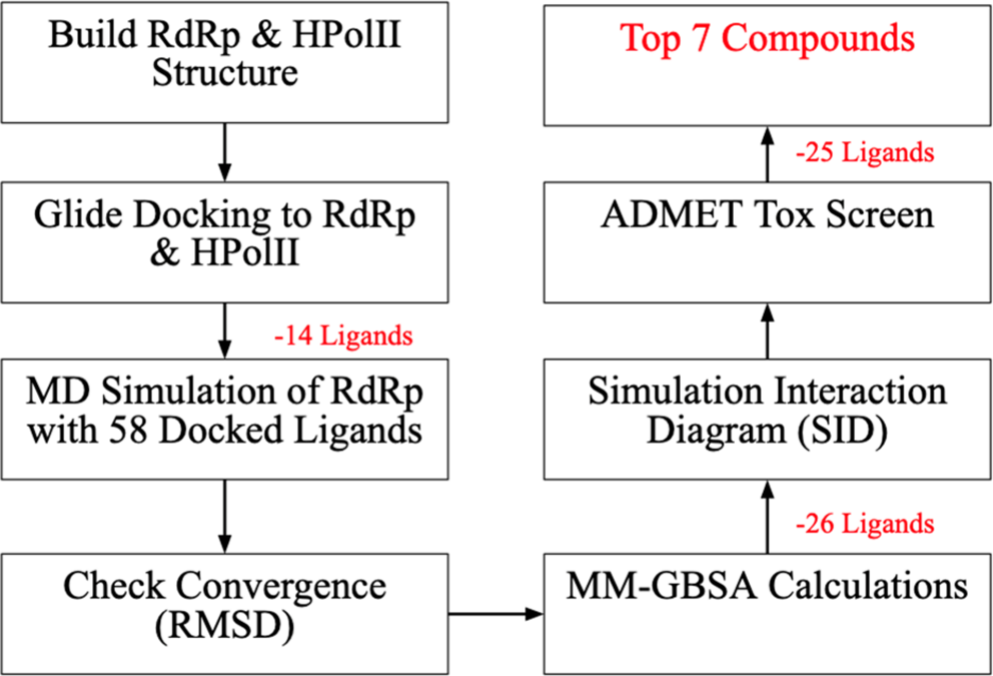
Workflow displaying the computational pathway and steps utilized in the selection of candidates. Steps denoting the filtering of compounds from the original 71 compounds generated from library enumeration are in red font.

To elucidate the best candidates that were obtained from this library screening, we utilized MD simulation results, post-MD analysis (RMSD and MM-GBSA analyses), and ADMET drug property prediction to ultimately identify just seven top analogues, which become the focus of this work. The logic used for this step is summarized in a workflow (Figure 4).

**Figure 5:**
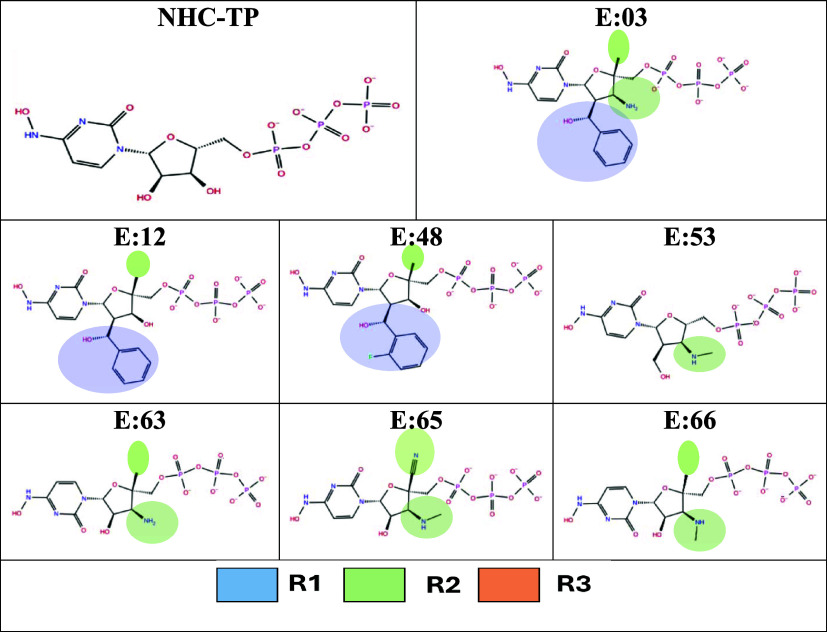
Chemical structure of the top seven NHC-TP analogue candidates with substitutional sites R1, R2, and R3.

Prioritization of simulation results in evaluating the simulation stability (RMSD) over the last 50 ns of the ligand main atoms to be more favorable (lower) than seen in the SARS-COV-2 RdRp system containing NHC-TP. Then, the top compounds with the best MM-GBSA (Δ*G*_TOT_) binding free energy calculations compared to NHC-TP were selected to present a better binding affinity to SARS-COV-2 RdRp than HPolII (Tables 1 and S4). Lastly, we assessed the prodrug adsorption, distribution, metabolism, excretion, and toxicity (ADMET) properties with similar criteria to the reference compound molnupiravir, specifically concentrating on the average LogS and consensus LogP values (Tables 3 and S5). The top seven NHC-TP analogues selected are shown with the addition sites highlighted in Figure 5 and will be the focus throughout this analysis.

### 3.4 Post-MD RMSD, RMSF, and Radius of Gyration Analyses of 58 SARS-COV-2 RdRp-NHC-TP Systems

After this initial filtering, the 58 enumerate ligand complexes were subjected to 200 ns MD simulations each to gain more information on the stability of the binding pose. The trajectory convergence of each of the 58 ligand complexes after simulation is visualized using the protein and ligand-fit of protein RMSD shown in Figure S9, with the averaged values of the last 50 ns of simulation time being tabulated in Table S4.

**Figure 6:**
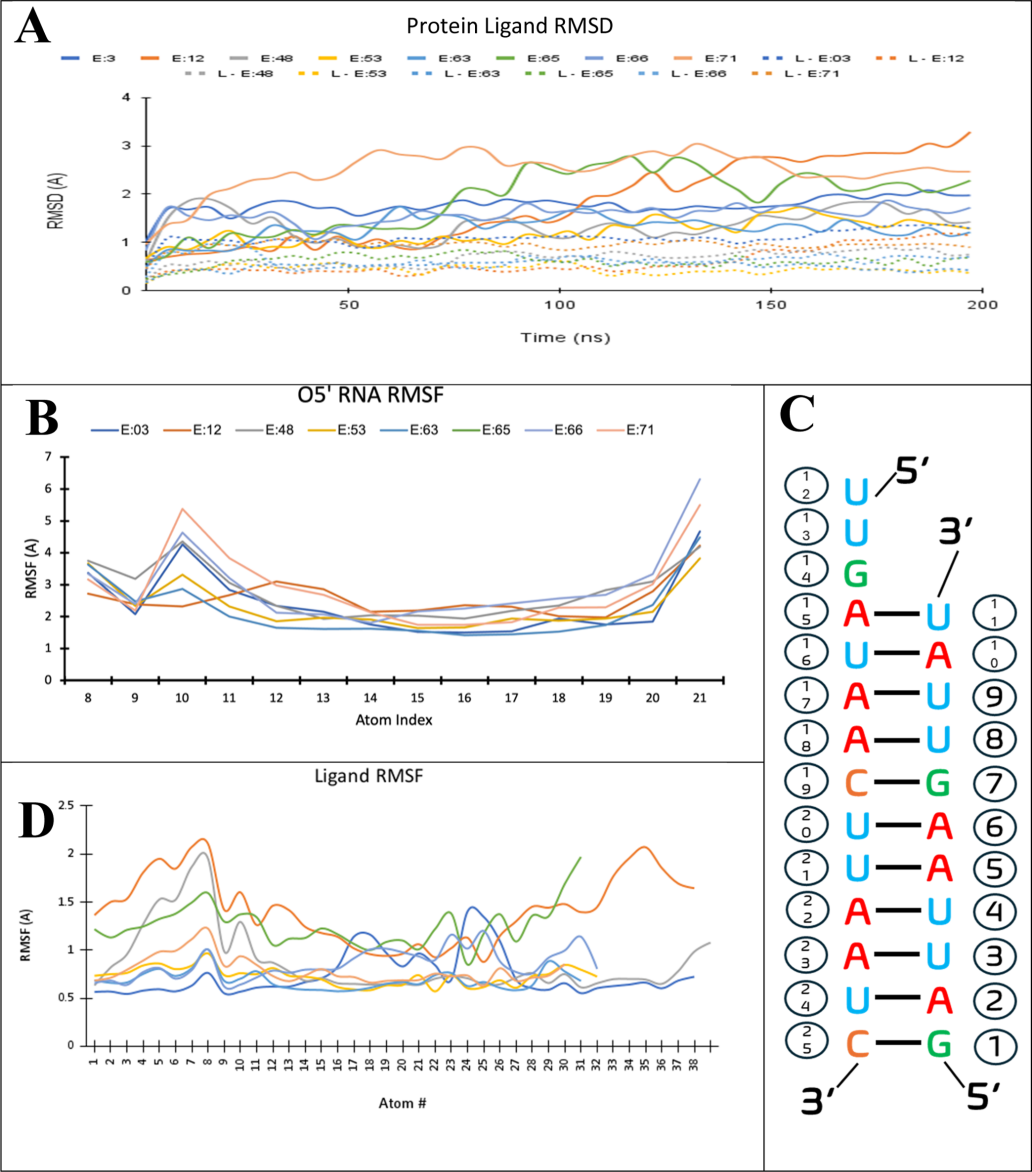
(A) Protein–ligand RMSD and ligand RMSD for NHC-TP and top 7 ligand trajectories for the SARS-COV-2 RdRp systems. (B) RMSF values of the template-primer RNA O5′ atoms for NHC-TP and top 7 ligand trajectories. (C) dsRNA strand with incorporation of G10 mutation. (D) Ligand RMSF values of the ligand heavy atoms of each analogue SARS-COV-2 RdRp system during 200 ns MD simulation.

The RMSD of the ligand heavy atoms (C, O, N, P) and protein Cα atoms can give insight into the total movement of the atoms of each species over the run time of the simulation compared to the reference compound NHC-TP. The average protein and ligand RMSD values over the last 50 ns of simulation for NHC-TP and the top 7 selected analogues are tabulated in Table 1 and are shown in Figure 6A.

The protein receptor Ca achieved stability very early into the simulation (∼25 ns) and remained stable during the remaining duration at RMSD ∼ 2–3 Å and the ligand RMSD was below that of the protein (RMSD ∼ 1 Å) (Figure 6A). Both protein and ligand RMSD values for all systems were stable with the highest protein RMSD value being 2.0 Å for E:63. As expected, Mg^2+^ ions remained in close proximity to the phosphate tail of NHC-TP and the 58 analogues, indicating their crucial role in stabilizing nucleoside triphosphate ligands within the active site. The average ligand RMSD values were selected to be lower than the NHC-TP ligand with the intention of selecting ligands with little spatial deviation from the starting frame, indicating conformational stability (Figure 6A, Tables 1 and S4).

Furthermore, to gain more insight into how each atom of NHC-TP and the top 7 analogues behaved during the simulation, the ligand RMSF can be explored. The RMSF value plots of the ligand main atoms are shown in Figures 6Dand S14. Starting with conserved atom indices 1–27 belonging to the reference scaffold of the ribose-nucleic and triphosphate tail portion, RMSF values were lower in analogues E:63, E:12, E:66, E:65, E:53, and E:03 (RMSF < 1.0 Å) compared to NHC-TP, indicating the higher atomic stability for these compounds. High fluctuations were observed at N4-hydroxyl oxygen on the cytidine motif (atom 8) for all eight ligands, indicating a slight increase in instability due to the intermittent hydrogen pair formation with G10 (Figure 6D). E:12 is observed to have the highest overall fluctuation at this site with a peak value of 2.1 Å, showing that the addition of substitutional sites at the R1, R2, and R3 positions have enhanced the stability of fluctuations to be less than 2 Å. Likewise, atomic stabilities on the ribose ring (atoms 1, 2, 11, 13, and 28) are observed to have a lower overall fluctuation in the above-mentioned analogues because of the added substitutional group stabilizing interactions within the enzyme active site (Figure 6D). A strong peak at atom positions of E:63 (atom 23) compared to NHC-TP and the other seven ligands with a value of 1.3 Å is due to the movement of interacting Mg^2+^ ions with the phosphate backbone. A peak of fluctuation seen in E:03 and E:53 at β-phosphate (atom 25) had an RMSF value of 1.45 Å, which is above the average for the rest of the phosphate tail. While most RMSF peaks are due to the phosphate tail interactions, all of the ligands showed a peak in their hydroxylamine group (atoms 5, 8, 10) binding to adjacent guanosine. Otherwise, these systems remain very stable (0.5–1.5 Å) throughout the trajectory presenting a proper nucleotide addition pose.

The RNA O5′ RMSF of the model of SARS-CoV-2 RdRp containing 11 bases in the primer strand and 14 bases in the template strand was then performed. The RNA O5′ RMSF displayed fluctuations at atoms 10 and 11 associated with A10 and U11 of the product strand and showed the most fluctuation with NHC-TP (Figure 6B,6C). The ligand fluctuation remained around 1 Å from start to finish, while the protein fluctuation was also around 1–0.5 Å after achieving stability (Figure 6D). The fluctuation values of template-primer RNA O5′ atoms were explored for NHC-TP, as shown in Figure 6B,6C. The RMSF values remained stable (<3.0 Å), while peaks at atoms 10, 11, and 21 were observed; the O5′ peak at atom 21 is likely due to RNA being out of the active site, causing a high RMSD, whereas atoms 22–25 are neglected for instability out of the active site. A significant peak of atom 12 or U8 on the 5′ terminus of the template strand showed a fluctuation value of 5.5 Å. The other peaks at atoms 1 and 21 correlate to the nucleobase primer strand 5′ terminal G10 and template strand 3′ terminal C21, respectively. Atom 14 or G10 in the template strand retained a low RMSF value, indicating a stable conformation with NHC-TP during simulation. Overall, the RMSF values of NHC-TP were considered stable (Figure 6B). The same can be stated for the seven analogue systems with similar peaks being seen at nucleosides U8 and A10, indicating an expected fluctuation for RNA in the *i*-site of SARS-COV-2 RdRp. However, the magnitudes of these peaks, especially for the systems containing E:53 or E:71 (3.0 Å), are higher and show more fluctuation in midhelix bases.^2,6,18,20^ indicating an induced instability within the RNA chain, which could potentially contribute to a decrease in the overall SARS-COV-2 RdRp turnover rate *in vitro*.

Lastly, the radius of gyration (rGyr) was calculated for the top 7 ligand analogues to determine their size relative to NHC-TP (Table 1). Relative to NHC-TP, a higher rGyr indicates that the ligand atoms are further away from their center of mass (larger ligand), whereas the opposite is true if a lower rGyr is observed (smaller ligand). Hence, ligands with a higher rGyr could pose as a selectivity factor against HPolII due to the increased ligand size. The rGyr calculations for NHC-TP (5.010 Å) is the reference size; in comparison, E:63 (5.229 Å), E:12 (5.304 Å), and E:65 (5.142 Å) demonstrate a slightly larger ligand size, whereas E:66 (4.856 Å), E:53 (4.894 Å), E:48 (4.707 Å), and E:03 (4.711 Å) demonstrate a slightly smaller ligand size. These differences in ligand sizes could pose advantageous selectivity factors against HPolII for E:63, E:12, and E:65, which could also explain their enhanced binding free energy scores over ligands E:53, E:48, and E:03 to SARS-COV-2 RdRp, as is noted later.

### 3.5 MM-GBSA Binding Free Energy for the Post-MD Ligand Set

All 58 ligand SARS-COV-2 RdRp systems were subjected to MM-GBSA predictions to better assess relative ligand binding affinities toward the enzyme. The summary of the predicted binding free energy values is tabulated in Table S4. NHC-TP received a binding free energy value (Δ*G*_TOT_) of −345.3 kcal/mol. Out of the total number of production runs, 58 out of the 71 ligands were predicted to have a lower overall Δ*G*_TOT_ lower than NHC-TP with the most negative value of −408.1 kcal/mol belonging to E:63. Lastly, trending in a more expected direction as with the pre-MD results, substitution on the R3 position saw 32 analogues with either methylamine or isopropylamine addition.

Table 1 shows the average Δ*G*_TOT_ values for NHC-TP, E:03, E:63, E:66, E:65, E:48, and E:53. The graphs showing the Δ*G*_TOT_ over the last 50 ns of simulation time for the top 7 ligands are also shown in Figure S15. As shown in Figure S15, similar to the RMSD, convergence of Δ*G*_TOT_ is met when the value stabilizes over the simulation time. All 7 top ligands demonstrated convergence in their Δ*G*_TOT_ (Figure S15), indicating that their average Δ*G*_TOT_ values, as listed in Tables 1 and S3, should exhibit relatively low standard deviation values. The Δ*G*_TOT_ for each analogue system was shown to have a higher affinity than NHC-TP, with E:63 exhibiting the lowest Δ*G*_TOT_ value (−408.1 ± 25 kcal/mol) and E:03 exhibiting the highest Δ*G*_TOT_ value (−332.0 ± 28 kcal/mol). While the Δ*G*_TOT_ value of E:03 is slightly worse than the Δ*G*_TOT_ value of the NHC-TP substrate (+13.3 kcal/mol difference), E:03 is still included in the post-MD analyses due to it bearing modifications on its R1, R2, and R3 positions, which could offer selectivity against HPolII. Furthermore, E:03 exhibited a more favorable SP docking score (−9.31 kcal/mol) compared to NHC-TP (−8.75 kcal/mol) for SARS-COV-2 RdRp (Table 1), and thus it was passed for MD simulation.

Additionally, Δ*G*_TOT_ consists of three major contributing terms, namely, the van Der Waals (Δ*E*_vdW_), electrostatic (Δ*E*_ELE_), and hydrophobic energies (Δ*E*_HYD_). Each term was analyzed for each ligand to elucidate their different Δ*G*_TOT_ values between each other. Six of the 7 top ligands showed favorable van der Waals energies (Δ*E*_vdW_) over NHC-TP except for E:48, which contains a large fluoromethyl phenolate group causing large van der Waals force changes due to the electronegativity in the R1 group addition at C2′. This large electronegative side chain has been tested previously by Williams et al., where electronegative groups such as OH and F on C2′ showed the locking of conformations for the ribose in DNA that showed puckering at C3′ endo (RNA favored) and at C2′ endo (DNA) favored.^40^ The electrostatic energy (Δ*E*_ELE_) was seen to contribute to the total affinity with the highest belonging to E:66 with a value of −150.6 ± 24 kcal/mol. Ligands E:03, E:12, and E:63 were seen to have less contributing energy from Δ*E*_ELE_ due to lesser residue interactions between charged amino acids and the phosphate tail seen in the most abundant conformations. Lastly, the hydrophobic contributions (Δ*E*_HYD_) added to the total energies for all six ligands, with E:03 exhibiting the highest Δ*E*_HYD_ value of −112.7 ± 7 kcal/mol. From this, all 7 top ligands saw significant improvement in their binding affinities after 200 ns MD simulation.

### 3.6 Caver Channel Analysis and Comparison with SARS-COV-2 RdRp and HPolII

**Figure 7:**
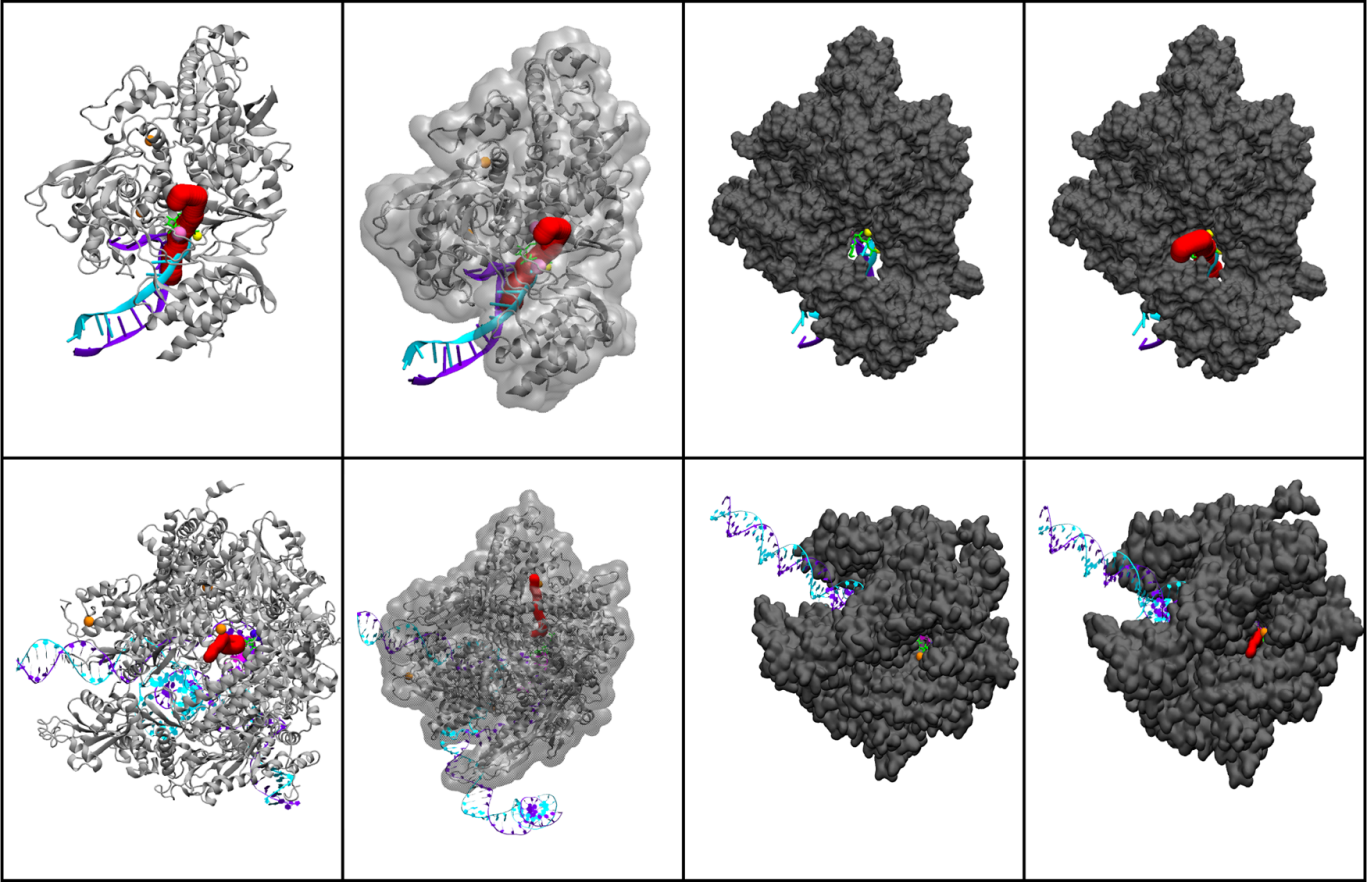
Caver analysis of SARS-COV-2 RdRp from left to right. (Left) ribbon representation of the SARS-CO-2 RdRp protein (silver) with the template RNA strand (magenta) and product strand (cyan), enumerate ligands (green), Zn^2+^ ions (orange balls), and Mg^2+^ ions (purple balls) with the calculated channel (red). (Middle) ribbon and transparent surface representation showing the channel through the protein’s surface. (Right) surface representation (gray) and calculated entry channel for nucleotide incorporation (red). Row 1: Caver analysis of SARS-COV-2 RdRp in complex with the top 7 enumerate ligands. Row 2: Caver analysis of HPolII in complex with the top 7 enumerate ligands.

The Caver 3.0 channel analysis program was used to compute tunnels in the most abundant cluster SARS-COV-2 RdRp (Figure 7 and S5) and enumerate docked HPolII (Figure 7 and S6) and compare the channels of SARS-COV-2 RdRp and HPolII for potential NTP entry (Figure 2 and Table 2).

Channels were computed at the centroid of the NTP docked ligands. Surface representations of the computed channels show the length and overall size of each channel (Figure 7, S5 and S6). The structure of SARS-COV-2 RdRp showed a significantly greater entry size with multiple tunnels of entry (Figure 7 and S5).

In comparison, the bottlenecks in HPolII in which the radius was the smallest throughout the tunnel were larger in NHC-TP and E:12 (Table 2, Figure 7 and S6). The length of the channels differ greatly as HPolII is much larger than SARS-COV-2 RdRp. The SARS-COV-2: E:65 complex computed a larger channel traversing the outside of the protein and into the RNA channel, generating a large channel length (42.2 Å). Among these results, SARS-COV-2 RdRp overall showed a significantly smaller bottleneck size with enumerate ligands bound, possibly due to large conformational changes in motifs F and C upon binding of NTP in the active site. HPolII exhibits a much larger active site than SARS-COV-2 RdRp and less interactions with the incoming NHC-TP substrate and analogue, which likely causes the stable bottleneck size of 3.0–3.4 Å. From this data, we can infer that upon natural NTP addition, bound NHC-TP with a large bottleneck 3.87 Å at the *i* + 1 site may be more practical for our enumerate binding, while adjacent binding of our top 7 enumerate ligands may be less likely.

### 3.7 Clustering Analysis, MD, and SP Ligand Docking Pose Comparison and Ligand–Protein Contacts

**Figure 8:**
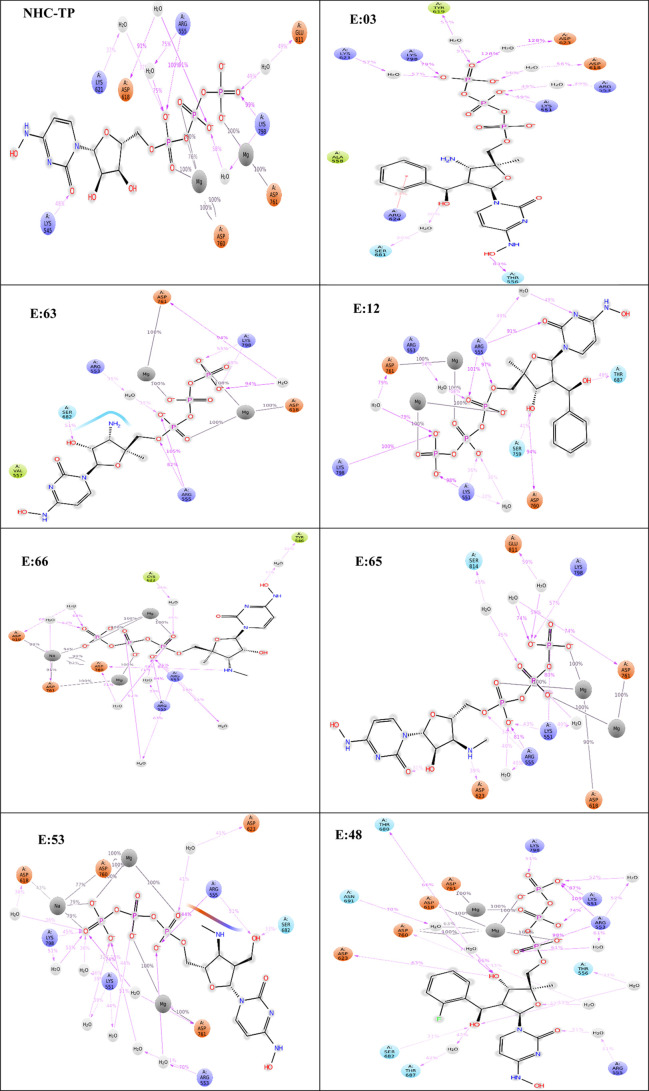
Most abundant conformation obtained from the MD simulation of the full SARS-CoV-2 RdRp enzyme (silver) in complex with the template (red) and primer RNA (blue) and reference NHC-TP (E:71) colored by the element. Ligand–protein contact summary during simulation with residue contacts displayed on ≥30% occupancy.

Figure 8 (protein + RNA + ligand) and Figure S8 (RNA + ligand) show the most abundant conformational cluster extracted from the MD trajectories of the SARS-COV-2 RdRp systems as well as the ligand–protein contact occupancies during the trajectory, similar to the findings in previous statistical mechanics, QM/MM, and DFT/MM-MD studies performed by Aranda et al. and Bignon et al.^41^

In addition to the more favorable Δ*G*_TOT_ binding free energy values from MM-GBSA analysis, we also measured the hydrogen-bonding distance of three atoms (1′, 2′, and 3′) within NHC-TP and the top 7 ligands to three atoms in the G10 nucleoside for the post-MD most abundant cluster pose and pre-MD SP docked pose to elucidate if these interactions improved after MD simulation. The hydrogen-bonding distances in Figure S8 were tabulated in Table S5. A decrease in the hydrogen-bonding distance indicates pose improvement following MD simulation. The NHC-TP hydrogen-bonding distances in its post-MD pose (4.53, 5.27, and 6.90 Å for P1′, P2′, and P3′ positions, respectively) were overall decreased compared to its SP docked pose distances (4.81, 5.44, and 7.15 Å) (Table S5). Indeed, this was also generally observed for the top 7 ligands in E:63 (2.64/2.76, 2.99/2.98, and 3.65/2.91 Å for post-MD and pre-MD SP docked poses in P1′, P2′, and P3′, respectively), E:12 (6.00/8.42, 5.09/5.53, and 4.78/2.60 Å), E:66 (2.92/2.89, 2.94/2.99, and 2.69/2.79 Å), E:65 (2.67/2.93, 2.86/2.81, and 2.95/3.04 Å), E:53 (2.73/2.74, 2.99/2.93, and 3.00/2.74 Å), E:48 (5.23/6.66, 5.88/5.94, and 8.04/7.30 Å), and E:03 (3.12/2.88, 2.87/3.00, and 2.84/2.94 Å) (Table S5). An increased hydrogen-bonding distance occurred the most at P3′ and less frequently in P1′ and P2′. When viewed another way, the post-MD pose hydrogen-bonding distances in NHC-TP for P1′ (4.53 Å), P2′ (5.27 Å), and P3′ (6.90 Å) were reduced for most of the top 7 ligands in P1′ (2.64–6.00 Å), P2′ (2.86–5.88 Å), and P3′ (2.69–8.04 Å). This could suggest that while MD simulations generally refined the conformational poses for NHC-TP and the top 7 ligands, the top 7 ligands could have enhanced conformational poses relative to NHC-TP, which may contribute toward their enhanced MM-GBSA Δ*G*_TOT_ values.

Figure S11 shows the 2D protein–ligand interaction diagrams after MD simulation and Figure S15 shows the occurrence histogram of each interaction during the 200 ns MD trajectory. To measure the likelihood of protein–ligand contacts, the percentage of interaction time between the ligand and its contacts will be represented as *XXX*%. The ligand–protein contacts shown for E:71 as the reference shows numerous contacts with active site residues, both Mg^2+^ ions and water molecules (Figure 8). Favorable ligand–receptor contacts for NHC-TP incorporation should be similar to NTP incorporation: specifically, electrostatic interactions between acidic receptor residues (D760), basic residues (R555, K551, R553, K621), Mg^2+^, and complementary base pairing with the template RNA nucleoside should facilitate phosphodiester cleavage and hence the incorporation of NHC-TP analogues into the complementary RNA strand.

NHC-TP maintained interactions with R555 (185%) and D760 (190%) with high occupancy. Note that binding occupancies greater than 100% occur when a residue has multiple chemical interactions with the same ligand atom and is indicative of more favorable binding. The salt bridge formation of positive residue amino acids such as K551, R553, R555, and K621 with the triphosphate tail of NHC-TP is commonly seen within both RTP and ATP binding to RdRp;^42^ hence, our trajectory results for NHC-TP are consistent with the reported literature. Interestingly, NHC-TP interactions with R553 (75%) and K621 (50%) were of lower occurrence. Several new interactions with NHC-TP were maintained with high occupancy, like ionic interactions with D760, negative interactions (e.g., water bridges with D618 (95%) and K798 (165%)), and Mg^2+^ interactions with the triphosphate tail were observed. Clearly, NHC-TP had the ability to maintain contacts with the active site residue and both Mg^2+^ ions with moderate occupancy in SARS-COV-2 RdRp, supporting its potential NHC-TP incorporation. Next, the results with the top 7 ligands were analyzed to determine if these contacts were conserved and if novel contacts formed.

E:63 displayed the highest MM-GBSA binding free energy out of the 7 top ligands and exhibited selective contacts with SARS-COV-2 RdRp active site residues, both Mg^2+^ ions and water ions (Figure 8). Negatively charged D761 and D618 interact with Mg^2+^ ions interacting with the phosphate tail of E:63. The C3′ amine substitution of E:63 did not interact with the active site. The methyl addition to C4′ did not engage in charged or polar interactions. Positively charged residues K798, R553, and R555 showed favorable bonding to the phosphate tail of E:63. A polar interaction with S682 with the C2′ hydroxyl group of E:63 (51%) suggests a possible selectivity advantage in the binding site from this new interaction. Negatively charged aspartate residues interact with bonded Mg^2+^ ions with the phosphate tail of E:63. Along with these interactions, D623 showed H bonding with a nearby water molecule facilitating a bond network with C3′ of E:63 (88%). Positively charged residues K553, K798, and R555 showed favored bonding to the phosphate tail of E:63 along with two hydrogen bond networks facilitated by water molecules between γ-phosphate of E:63 and Y619 (94%) and C622 (58%).

E:12 (Figure 8) exhibited numerous strong contacts with K545, creating a salt bridge with both the nucleobase (42%) and β-phosphate (69%). A contact between R555 (38%) involving pi–pi stacking with the 2′ incorporated side chain of E:12 also occurred. Additionally, many negatively charged interactions occurred between the phosphate tail of E:12 and both Mg^2+^ ions (100%). Finally, water bridges formed with D618 (78%) and D760 (34%) and D761 (32%) and E811 (70%) with the triphosphate tail of E:12. Other stabilizing interactions on the phosphate backbone include K551 (80%) and K798 (30%) with γ-phosphate of E:12.

E:66 (Figure 8) displayed a new contact within its hydroxylamine nucleobase group with T546 and a water molecule, possibly displaying a guided hydrogen-bonding interaction for the entering NTP. Along with this interaction, D760 displayed interactions through a hydrogen-bonding network with the methylamine addition to C3′ of E:66 (86%). The C4′ methyl addition of E:66 did not display any contacts within 30% occupancy. The phosphate tail of E:66 displayed interactions with nearby Na^+^ ions (from the neutralizing 0.15 NaCl salt), both Mg^2+^ ions (100%), and negatively charged interactions with D618 and D761. Positively charged contacts with the phosphate backbone of E:66 and R553 (71%) and R555 (94%) were also observed.

E:65 (Figure 8) displayed a new negative interaction without a water molecule at its C3′-substituted methylamine with D623 through possible hydrogen bonding. Intramolecular interactions also occurred between the unsaturated carbonyl oxygen of the hydroxylamine group and the C2′ position of E:65. This has been shown to occur in tautomeric states of hydroxylamine groups^21^ and shows the possible equilibrium change to an oxime form. Among these interactions, charged networks from D618, D761, and both Mg^2+^ ions with the phosphate tail of E:65 are involved. A new polar interaction with S814 was maintained with the phosphate tail of E:65 (45%), along with a new negatively charged Glu-driven water network with E:65. Positively charged interactions with the phosphate tail of E:65 and K798 (57%), K551 (80%), and R555 (81%) were also observed.

Interestingly, E:53 only displayed 2 protein–ligand contacts apart from the triphosphate tail (Figure 8). One polar interaction with S682 (33%) and a positively charged interaction with Arg555 (51%) occurred at 2′ hydroxyl of E:53. As for the phosphate tail of E:53, the main interactions include the most negative interactions with D760 (100%) and D761 (100%) and interactions with both Mg^2+^ ions, which in turn interacted favorably with β- and γ-phosphates of E:53. Other positive interactions also occurred between R555 (44%) with β-phosphate of E:53 and K798 (45%) and negatively charged interactions that formed water bridges of H bonding between D623 (41%) and β-phosphate of E:53 (41%). Interestingly, this system had a Na^+^ interaction with γ-phosphate (79%) and interactions stabilizing the Na^+^ ion were cycled between D618 (43%) and D760 (77%) of the receptor. This interaction may not be significant due to the Na^+^ ion being sourced from the neutralizing 0.15 M NaCl salt.

E:48 was one of the compounds that introduced an electronegative fluoro-phenolate group to its substituents (Figure 8), causing large charge differences throughout the molecule. While no interactions are directly noted with this group, new interactions with positively charged R555 are shown to form water H-bond networks with hydroxylamine of the nucleobase of E:48 (62%). Along with this interaction, T556, S682, and T687 all form interactions with the hydroxyl group at the C2′ additions of E:48 (<35%). The O4′ position of E:48 also showed a new interaction with a water molecule (33%). C3′ hydroxyl of E:48 also shows negative interactions with D623 (63%), along with a H-bond network with T680 (66%). Among these new interactions, conserved interactions with the phosphate tail of E:48 with Mg^2+^ ions and D618, D761, K798, K551, and R553 retained high occupancy. With the new interactions caused through the electronegative group addition, it can be concluded that there are large amounts of charge interactions occurring on an intramolecular level.

Lastly, E:03 (Figure 8) established strong activity with negatively charged residues D623 (128%) and D618 (56%), as well as water bridge formation with α- and γ-phosphate of E:03. More modest contacts were seen including positively charged residues K798 (79%), K551 (59%), and R553 (49%) also forming water bridges with β-phosphate of E:03. The incorporated side chains had increased interactions with the active site such as S681, forming a water bridge (36%) and R624 (33%) via pi–pi stacking with the phenyl ring side chain. Unlike RDV, we did not observe the amine side chain reacting with active site residues. Additionally, E:03 was the only NHC-TP analogue out of the top 7 which did not exhibit interactions with Mg^2+^ ions and its triphosphate tail; in the 2D protein–ligand interaction diagram, however, both Mg^2+^ ions are within the active site and seem to be in close contact with the triphosphate tail of E:03.

As like with the other NHC-TP analogues, a pocket of several stabilizing charged residues (D760, D761, R553) formed contact with the triphosphate portion and/or nearby Mg^2+^ ions with very high occupancies, which further affirms the crucial role of these residues in nucleoside entry and Mg^2+^ stabilization for SARS-COV-2 RdRp function.^15^ During the trajectories, several new residue contacts not seen with significant occurrence in NHC-TP binding were seen with the top 7 analogues. Residues K551, R555, D618, D760, and D761 are of high importance in binding of NHC-TP analogues with 2/3, 5/6, and 1/2 of all systems seeing the interaction with each, respectively. T687 was also shown in the interaction with the 2′ hydroxyl as well as the hydroxylamine portions of molnupiravir. Previous studies have concluded that D760 and D761 is crucial for binding.^15,40^ T687 and S759 were seen to help stabilize the ribose ring and phosphate tail portions, respectively.

### 3.8 ADMET Property Predictions

Lastly, specific adsorption, distribution, metabolism, excretion, and toxicity (ADMET) properties were predicted for the prodrug forms of each NHC-TP analogue using the SwissADME Web server and compared to FDA-approved RDV in its prodrug form. The computed properties for the top 7 ligands and for the 58 filtered ligands following SP docking and MD simulation are tabulated in Tables 3 and S6.

The computed ADMET properties of RDV indicate its low GI absorption and inhibition of the CYP3A4 enzyme. In comparison, NHC-TP and the top 7 NHC-TP derivatives showed low GI absorption, so formulation of a prodrug would be needed to improve GI absorption. The average LogS was calculated based on the mean values determined by the ESOL, Ali, and Silicos-IT methods. Compared to RDV, each derivative saw a slight increase in their Average LogS values, with the highest belonging to E:48 with a value of −2.96. Likewise, the synthetic accessibility was also examined and compared to Remdesivir. On a 0–10 scoring system, lower scores indicate compounds that are easier to synthesize as predicted using the FP2 method by machine learning. All prodrugs saw a slight improvement (decrease) in values, with the lowest also being E:65 with a value of 4.69. Additionally, the prodrug pharmacokinetic information was compared to RDV. All seven prodrug derivatives were predicted to not be major inhibitors of cytochrome P450 enzymes.

## 4 Conclusions

This study was performed with the aim to gain a better understanding of molnupiravir’s active form NHC-TP toward SARS-CoV-2 RdRp by coupling molecular docking, MD simulation, and MM-GBSA binding free energy predictions. Post-MD simulation results show that the key residues binding to NHC-TP were R545, K551, R555, T556, D618, T687, D760, and D761, all with a modest to high occurrence throughout 200 ns MD simulation, and the MM-GBSA binding free energy simulation was −345.3 kcal/mol. Then, an enumerate library of 71 NHC-TP derivatives bearing several functional groups to substitution sites R1, R2, and R3 was generated to identify potential ligands with selectivity for SARS-COV-2 RdRp over HPolII, using the same computational methodology. Seven out of the 71 top derivatives (E:03, E:63, E:12, E:66, E:65, E:48, and E:53) were selected based on the SP docking score, MM-GBSA binding free energy, and ADMET property evaluation compared to NHC-TP and RDV. All 7 derivatives possess higher MM-GBSA values compared to NHC-TP, with E:03 exhibiting the highest Δ*G*_TOT_ of −433.2 kcal/mol. The increased MM-GBSA energy saw an increased contribution from Δ*E*_VDW_ and Δ*E*_HYD_ terms due to novel active site residue–ligand contacts occurring during the simulation (e.g., aromatic groups on the R1 site), an increase in the molecular size, and additional opportunities for a polar-induced dipole interaction with surrounding active site residues. A list of novel interacting residues that contacted the derivatives not seen in NHC-TP binding include R551, S681, T556, Y629, and S759. The significance of finding the correct NHC-TP pose for nucleotide incorporation was of highest priority when generating binding poses to begin MD and this is seen throughout our study for the low free energy outputs associated with the ability to keep the G–C base pairing of this keto hydroxylamine with guanosine in the RNA template strand. The top 7 NHC-TP derivatives incorporate two strategies of silent mutagenesis and replication stalling for a double threat approach to targeting SARS-COV-2 with a higher selectivity than HPolII: decreased binding affinity through molecular docking and NTP entry channel hindrances due to increased ligand size. Lastly, the predicted ADMET properties of the top 7 NHC-TP derivatives were similar to or slightly better than those properties by RDV and NHC-TP. In conclusion, this study provides a greater insight into the atomistic detail of potential NHC-TP and NHC-TP derivative incorporation into SARS-COV-2 RdRp and HPolII. The top 7 NHC-TP derivatives obtained from this study should be subjected to experimental testing and optimization to validate these findings and continue generating interest in the development of more small molecule therapeutics against COVID-19 infection as well as other viruses bearing RNA polymerases. These discoveries will contribute toward the effort in discovering more effective dual threat inhibitors for SARS-CoV-2 RdRp as well as presenting a brief SAR of molnupiravir bound to RdRp at the NTP addition site.

## Data Availability

We used Schrodinger 2016-3 for protein preparation. The Desmond package within the Schrodinger suite was used to calculate the RMSD timer series. All data generated or analyzed during this study are included in this published article and its Supporting Information document.

## Supporting Information Available

Docking score, molecular dynamics simulation information for all 72 enumerate ligand systems, and SMILES and OPLS3e force field parameters for top 7 enumerate ligands (PDF)
