# Supplementary material for: Binding Mechanism of the Active Form of Molnupiravir to RdRp of SARS-CoV-2 and Designing Potential Analogues: Insights from Molecular Dynamics Simulations
Source: ACS Omega. 2024 Sep 24;9(40):41583–98. doi: 10.1021/acsomega.4c05469 (PMC11465654; doi:10.1021/acsomega.4c05469)
Supplement: Supplementary file 1 — ao4c05469_si_001.pdf [file ao4c05469_si_001.pdf]

Binding Mechanism of Active Form of Molnupiravir to RdRp of SARS-CoV-2 and Designing Potential Analogues: Insights from Molecular Dynamic Simulations

Justin Carbone, Nicholas J. Paradis, Dylan Brunt and Chun Wu*

College of Science and Mathematics, Rowan University, Glassboro, NJ 08028 USA.

*To whom correspondence should be addressed: wuc@rowan.edu

Functional Group	R1	R2	R3
A	($-C_7H_7O$)	(-CN)	($-NH_2$)
B	(*p*-C_7H_6FO)	($-CH_3$)	($-CH_3NH$)
C	($-C_8H_6NO$)	-	($-C_3H_6NH$)
D	(*o*-C_7H_6FO)	-	-
E	($-CH_2OH$)	-	-

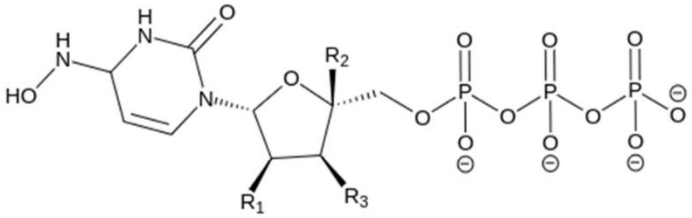

R_1 = (C_7H_7O), (*p*-C_7H_6FO), (*o*-C_7H_6FO), (C_8H_6NO), (CH_2OH)
R_2 = CN, CH_3
R_3 = NH_2, (CH_3NH), (C_3H_6NH)

Table S1. Tabulated functional groups selected on each of three substitutional sites on NHC-TP (R1, R2, R3) for the generation of the combinatorial library.

Ligand	SMILES
E:01	c1ccccc1[C@H](O)[C@H]([C@@H]2N)[C@H](n3ccc(NO)nc3=O)O[C@]2(C#N)COP([O-])(=O)OP([O-])(=O)OP([O-])([O-])=O
E:02	c1ccccc1[C@H](O)[C@H]2[C@H](n3ccc(NO)nc3=O)O[C@@H]([C@H]2N)COP([O-])(=O)OP([O-])(=O)OP([O-])([O-])=O
E:03	c1ccccc1[C@H](O)[C@H]([C@@H]2N)[C@H](n3ccc(NO)nc3=O)O[C@]2(C)COP([O-])(=O)OP([O-])(=O)OP([O-])([O-])=O
E:04	c1ccccc1[C@H](O)[C@H]([C@@H]2NC)[C@H](n3ccc(NO)nc3=O)O[C@]2(C#N)COP([O-])(=O)OP([O-])(=O)OP([O-])([O-])=O
E:05	c1ccccc1[C@H](O)[C@H]2[C@H](n3ccc(NO)nc3=O)O[C@@H]([C@H]2NC)COP([O-])(=O)OP([O-])(=O)OP([O-])([O-])=O
E:06	c1ccccc1[C@H](O)[C@H]([C@@H]2NC)[C@H](n3ccc(NO)nc3=O)O[C@]2(C)COP([O-])(=O)OP([O-])(=O)OP([O-])([O-])=O
E:07	c1ccccc1[C@H](O)[C@H]([C@@H]2NC(C)C)[C@H](n3ccc(NO)nc3=O)O[C@]2(C#N)COP([O-])(=O)OP([O-])(=O)OP([O-])([O-])=O
E:08	c1ccccc1[C@H](O)[C@H]2[C@H](n3ccc(NO)nc3=O)O[C@@H]([C@H]2NC(C)C)COP([O-])(=O)OP([O-])(=O)OP([O-])([O-])=O
E:09	c1ccccc1[C@H](O)[C@H]([C@@H]2NC(C)C)[C@H](n3ccc(NO)nc3=O)O[C@]2(C)COP([O-])(=O)OP([O-])(=O)OP([O-])([O-])=O
E:10	c1ccccc1[C@H](O)[C@H]([C@@H]2O)[C@H](n3ccc(NO)nc3=O)O[C@]2(C#N)COP([O-])(=O)OP([O-])(=O)OP([O-])([O-])=O
E:11	c1ccccc1[C@H](O)[C@H]2[C@H](n3ccc(NO)nc3=O)O[C@@H]([C@H]2O)COP([O-])(=O)OP([O-])(=O)OP([O-])([O-])=O
E:12	c1ccccc1[C@H](O)[C@H]([C@@H]2O)[C@H](n3ccc(NO)nc3=O)O[C@]2(C)COP([O-])(=O)OP([O-])(=O)OP([O-])([O-])=O
E:13	c1cc(F)ccc1[C@H](O)[C@H]([C@@H]2N)[C@H](n3ccc(NO)nc3=O)O[C@]2(C#N)COP([O-])(=O)OP([O-])(=O)OP([O-])([O-])=O
E:14	c1cc(F)ccc1[C@H](O)[C@H]2[C@H](n3ccc(NO)nc3=O)O[C@@H]([C@H]2N)COP([O-])(=O)OP([O-])(=O)OP([O-])([O-])=O
E:15	c1cc(F)ccc1[C@H](O)[C@H]([C@@H]2N)[C@H](n3ccc(NO)nc3=O)O[C@]2(C)COP([O-])(=O)OP([O-])(=O)OP([O-])([O-])=O
E:16	c1cc(F)ccc1[C@H](O)[C@H]([C@@H]2NC)[C@H](n3ccc(NO)nc3=O)O[C@]2(C#N)COP([O-])(=O)OP([O-])(=O)OP([O-])([O-])=O
E:17	c1cc(F)ccc1[C@H](O)[C@H]2[C@H](n3ccc(NO)nc3=O)O[C@@H]([C@H]2NC)COP([O-])(=O)OP([O-])(=O)OP([O-])([O-])=O
E:18	c1cc(F)ccc1[C@H](O)[C@H]([C@@H]2NC)[C@H](n3ccc(NO)nc3=O)O[C@]2(C)COP([O-])(=O)OP([O-])(=O)OP([O-])([O-])=O
E:19	c1cc(F)ccc1[C@H](O)[C@H]([C@@H]2NC(C)C)[C@H](n3ccc(NO)nc3=O)O[C@]2(C#N)COP([O-])(=O)OP([O-])(=O)OP([O-])([O-])=O
E:20	c1cc(F)ccc1[C@H](O)[C@H]2[C@H](n3ccc(NO)nc3=O)O[C@@H]([C@H]2NC(C)C)COP([O-])(=O)OP([O-])(=O)OP([O-])([O-])=O
E:21	c1cc(F)ccc1[C@H](O)[C@H]([C@@H]2NC(C)C)[C@H](n3ccc(NO)nc3=O)O[C@]2(C)COP([O-])(=O)OP([O-])(=O)OP([O-])([O-])=O
E:22	c1cc(F)ccc1[C@H](O)[C@H]([C@@H]2O)[C@H](n3ccc(NO)nc3=O)O[C@]2(C#N)COP([O-])(=O)OP([O-])(=O)OP([O-])([O-])=O
E:23	c1cc(F)ccc1[C@H](O)[C@H]2[C@H](n3ccc(NO)nc3=O)O[C@@H]([C@H]2O)COP([O-])(=O)OP([O-])(=O)OP([O-])([O-])=O
E:24	c1cc(F)ccc1[C@H](O)[C@H]([C@@H]2O)[C@H](n3ccc(NO)nc3=O)O[C@]2(C)COP([O-])(=O)OP([O-])(=O)OP([O-])([O-])=O
E:25	c1cc(C#N)ccc1[C@H](O)[C@H]([C@@H]2N)[C@H](n3ccc(NO)nc3=O)O[C@]2(C#N)COP([O-])(=O)OP([O-])(=O)OP([O-])([O-])=O
E:26	c1cc(C#N)ccc1[C@H](O)[C@H]2[C@H](n3ccc(NO)nc3=O)O[C@@H]([C@H]2N)COP([O-])(=O)OP([O-])(=O)OP([O-])([O-])=O
E:27	c1cc(C#N)ccc1[C@H](O)[C@H]([C@@H]2N)[C@H](n3ccc(NO)nc3=O)O[C@]2(C)COP([O-])(=O)OP([O-])(=O)OP([O-])([O-])=O
E:28	c1cc(C#N)ccc1[C@H](O)[C@H]([C@@H]2NC)[C@H](n3ccc(NO)nc3=O)O[C@]2(C#N)COP([O-])(=O)OP([O-])(=O)OP([O-])([O-])=O
E:29	c1cc(C#N)ccc1[C@H](O)[C@H]2[C@H](n3ccc(NO)nc3=O)O[C@@H]([C@H]2NC)COP([O-])(=O)OP([O-])(=O)OP([O-])([O-])=O
E:30	c1cc(C#N)ccc1[C@H](O)[C@H]([C@@H]2NC)[C@H](n3ccc(NO)nc3=O)O[C@]2(C)COP([O-])(=O)OP([O-])(=O)OP([O-])([O-])=O
E:31	c1cc(C#N)ccc1[C@H](O)[C@H]([C@@H]2NC(C)C)[C@H](n3ccc(NO)nc3=O)O[C@]2(C#N)COP([O-])(=O)OP([O-])(=O)OP([O-])([O-])=O
E:32	c1cc(C#N)ccc1[C@H](O)[C@H]2[C@H](n3ccc(NO)nc3=O)O[C@@H]([C@H]2NC(C)C)COP([O-])(=O)OP([O-])(=O)OP([O-])([O-])=O
E:33	c1cc(C#N)ccc1[C@H](O)[C@H]([C@@H]2NC(C)C)[C@H](n3ccc(NO)nc3=O)O[C@]2(C)COP([O-])(=O)OP([O-])(=O)OP([O-])([O-])=O
E:34	c1cc(C#N)ccc1[C@H](O)[C@H]([C@@H]2O)[C@H](n3ccc(NO)nc3=O)O[C@]2(C#N)COP([O-])(=O)OP([O-])(=O)OP([O-])([O-])=O
E:35	c1cc(C#N)ccc1[C@H](O)[C@H]2[C@H](n3ccc(NO)nc3=O)O[C@@H]([C@H]2O)COP([O-])(=O)OP([O-])(=O)OP([O-])([O-])=O
E:36	c1cc(C#N)ccc1[C@H](O)[C@H]([C@@H]2O)[C@H](n3ccc(NO)nc3=O)O[C@]2(C)COP([O-])(=O)OP([O-])(=O)OP([O-])([O-])=O
E:37	c1cccc(F)c1[C@H](O)[C@H]([C@@H]2N)[C@H](n3ccc(NO)nc3=O)O[C@]2(C#N)COP([O-])(=O)OP([O-])(=O)OP([O-])([O-])=O
E:38	c1cccc(F)c1[C@H](O)[C@H]2[C@H](n3ccc(NO)nc3=O)O[C@@H]([C@H]2N)COP([O-])(=O)OP([O-])(=O)OP([O-])([O-])=O
E:39	c1cccc(F)c1[C@H](O)[C@H]([C@@H]2N)[C@H](n3ccc(NO)nc3=O)O[C@]2(C)COP([O-])(=O)OP([O-])(=O)OP([O-])([O-])=O
E:40	c1cccc(F)c1[C@H](O)[C@H]([C@@H]2NC)[C@H](n3ccc(NO)nc3=O)O[C@]2(C#N)COP([O-])(=O)OP([O-])(=O)OP([O-])([O-])=O
E:41	c1cccc(F)c1[C@H](O)[C@H]2[C@H](n3ccc(NO)nc3=O)O[C@@H]([C@H]2NC)COP([O-])(=O)OP([O-])(=O)OP([O-])([O-])=O
E:42	c1cccc(F)c1[C@H](O)[C@H]([C@@H]2NC)[C@H](n3ccc(NO)nc3=O)O[C@]2(C)COP([O-])(=O)OP([O-])(=O)OP([O-])([O-])=O
E:43	c1cccc(F)c1[C@H](O)[C@H]([C@@H]2NC(C)C)[C@H](n3ccc(NO)nc3=O)O[C@]2(C#N)COP([O-])(=O)OP([O-])(=O)OP([O-])([O-])=O
E:44	c1cccc(F)c1[C@H](O)[C@H]2[C@H](n3ccc(NO)nc3=O)O[C@@H]([C@H]2NC(C)C)COP([O-])(=O)OP([O-])(=O)OP([O-])([O-])=O
E:45	c1cccc(F)c1[C@H](O)[C@H]([C@@H]2NC(C)C)[C@H](n3ccc(NO)nc3=O)O[C@]2(C)COP([O-])(=O)OP([O-])(=O)OP([O-])([O-])=O
E:46	c1cccc(F)c1[C@H](O)[C@H]([C@@H]2O)[C@H](n3ccc(NO)nc3=O)O[C@]2(C#N)COP([O-])(=O)OP([O-])(=O)OP([O-])([O-])=O
E:47	c1cccc(F)c1[C@H](O)[C@H]2[C@H](n3ccc(NO)nc3=O)O[C@@H]([C@H]2O)COP([O-])(=O)OP([O-])(=O)OP([O-])([O-])=O
E:48	c1cccc(F)c1[C@H](O)[C@H]([C@@H]2O)[C@H](n3ccc(NO)nc3=O)O[C@]2(C)COP([O-])(=O)OP([O-])(=O)OP([O-])([O-])=O
E:49	O=c1nc(NO)ccn1[C@@H]([C@@H]([C@@H]2N)CO)O[C@]2(C#N)COP([O-])(=O)OP([O-])(=O)OP([O-])([O-])=O
E:50	O=c1nc(NO)ccn1[C@@H]([C@@H]2CO)O[C@@H]([C@H]2N)COP([O-])(=O)OP([O-])(=O)OP([O-])([O-])=O
E:51	O=c1nc(NO)ccn1[C@@H]([C@@H]([C@@H]2N)CO)O[C@]2(C)COP([O-])(=O)OP([O-])(=O)OP([O-])([O-])=O

Ligand	SMILES
E:52	O=c1nc(NO)ccn1[C@@H]([C@H](CO)[C@@H]2NC)O[C@]2(C#N)COP([O-])(=O)OP([O-])(=O)OP([O-])([O-])=O
E:53	O=c1nc(NO)ccn1[C@@H]([C@@H]2CO)O[C@@H]([C@H]2NC)COP([O-])(=O)OP([O-])(=O)OP([O-])([O-])=O
E:54	O=c1nc(NO)ccn1[C@@H]([C@H](CO)[C@@H]2NC)O[C@]2(C)COP([O-])(=O)OP([O-])(=O)OP([O-])([O-])=O
E:55	O=c1nc(NO)ccn1[C@@H]([C@H](CO)[C@@H]2NC(C)C)O[C@]2(C#N)COP([O-])(=O)OP([O-])(=O)OP([O-])([O-])=O
E:56	O=c1nc(NO)ccn1[C@@H]([C@@H]2CO)O[C@@H]([C@H]2NC(C)C)COP([O-])(=O)OP([O-])(=O)OP([O-])([O-])=O
E:57	O=c1nc(NO)ccn1[C@@H]([C@H](CO)[C@@H]2NC(C)C)O[C@]2(C)COP([O-])(=O)OP([O-])(=O)OP([O-])([O-])=O
E:58	O=c1nc(NO)ccn1[C@@H]([C@@H]([C@@H]2O)CO)O[C@]2(C#N)COP([O-])(=O)OP([O-])(=O)OP([O-])([O-])=O
E:59	O=c1nc(NO)ccn1[C@@H]([C@@H]2CO)O[C@@H]([C@H]2O)COP([O-])(=O)OP([O-])(=O)OP([O-])([O-])=O
E:60	O=c1nc(NO)ccn1[C@@H]([C@@H]([C@@H]2O)CO)O[C@]2(C)COP([O-])(=O)OP([O-])(=O)OP([O-])([O-])=O
E:61	O=c1nc(NO)ccn1[C@@H]([C@H](O)[C@@H]2N)O[C@]2(C#N)COP([O-])(=O)OP([O-])(=O)OP([O-])([O-])=O
E:62	O=c1nc(NO)ccn1[C@@H]([C@@H]2O)O[C@@H]([C@H]2N)COP([O-])(=O)OP([O-])(=O)OP([O-])([O-])=O
E:63	O=c1nc(NO)ccn1[C@@H]([C@H](O)[C@@H]2N)O[C@]2(C)COP([O-])(=O)OP([O-])(=O)OP([O-])([O-])=O
E:64	O=c1nc(NO)ccn1[C@@H]([C@H](O)[C@@H]2NC)O[C@]2(C#N)COP([O-])(=O)OP([O-])(=O)OP([O-])([O-])=O
E:65	O=c1nc(NO)ccn1[C@@H]([C@@H]2O)O[C@@H]([C@H]2NC)COP([O-])(=O)OP([O-])(=O)OP([O-])([O-])=O
E:66	O=c1nc(NO)ccn1[C@@H]([C@H](O)[C@@H]2NC)O[C@]2(C)COP([O-])(=O)OP([O-])(=O)OP([O-])([O-])=O
E:67	O=c1nc(NO)ccn1[C@@H]([C@H](O)[C@@H]2NC(C)C)O[C@]2(C#N)COP([O-])(=O)OP([O-])(=O)OP([O-])([O-])=O
E:68	O=c1nc(NO)ccn1[C@@H]([C@@H]2O)O[C@@H]([C@H]2NC(C)C)COP([O-])(=O)OP([O-])(=O)OP([O-])([O-])=O
E:69	O=c1nc(NO)ccn1[C@@H]([C@H](O)[C@@H]2NC(C)C)O[C@]2(C)COP([O-])(=O)OP([O-])(=O)OP([O-])([O-])=O
E:70	O=c1nc(NO)ccn1[C@@H]([C@H](O)[C@@H]2O)O[C@]2(C#N)COP([O-])(=O)OP([O-])(=O)OP([O-])([O-])=O
E:71	O=c1nc(NO)ccn1[C@@H]([C@@H]2O)O[C@@H]([C@H]2O)COP([O-])(=O)OP([O-])(=O)OP([O-])([O-])=O
E:72	O=c1nc(NO)ccn1[C@@H]([C@H](O)[C@@H]2O)O[C@]2(C)COP([O-])(=O)OP([O-])(=O)OP([O-])([O-])=O

Table S2. SMILES information for the NHC-TP substrate (E:71) and the 71 NHC-TP analogs generated through library enumeration (E:1-E:70, E:72).

Ligand	#Subs	Groups	Glide SP Score (kcal/mol)	Ligand	#Subs	Groups	Glide SP Score (kcal/mol)
E:01	3	R1a,R2a,R3a	-7.5	E:37	3	R1d,R2a,R3a	-8.7
E:02	2	R1a,R3a	X	E:38	2	R1d,R3a	-9.3
**E:03**	**3**	**R1a,R2b,R3a**	**-9.3**	E:39	3	R1d,R2b,R3a	-8.1
E:04	3	R1a,R2a,R3b	-9.3	E:40	3	R1d,R2a,R3b	-8.8
E:05	2	R1a,R3b	X	E:41	3	R1d,R3b	-9.0
E:06	3	R1a,R2b,R3b	-9.7	E:42	3	R1d,R2b,R3b	-8.6
E:07	3	R1a,R2a,R3c	-7.4	E:43	3	R1d,R2a,R3c	-9.7
E:08	2	R1a,R3c	-8.1	E:44	2	R1d,R3c	X
E:09	3	R1a,R2b,R3c	X	E:45	3	R1d,R2b,R3c	X
E:10	2	R1a,R2a	-8.8	E:46	2	R1d,R2a	-8.5
E:11	1	R1a	-9.1	E:47	1	R1d	-8.6
**E:12**	**2**	**R1a,R2b**	**-9.6**	**E:48**	**2**	**R1d,R2b**	**-9.6**
E:13	3	R1b,R2a,R3a	X	E:49	3	R1e,R2a,R3a	-9.1
E:14	2	R1b,R3a	X	E:50	2	R1e,R3a	-9.5
E:15	3	R1b,R2b,R3a	-11.5	E:51	3	R1e,R2b,R3a	-9.4
E:16	3	R1b,R2a,R3b	-8.1	E:52	3	R1e,R2a,R3b	-8.5
E:17	2	R1a,R3b	-7.8	**E:53**	**2**	**R1e,R3b**	**-9.5**
E:18	3	R1b,R2b,R3b	-11.3	E:54	3	R1e,R2b,R3b	-7.7
E:19	3	R1b,R2a,R3c	X	E:55	3	R1e,R2a,R3c	-9.2
E:20	2	R1a,R3c	-7.6	E:56	2	R1e,R3c	-7.6
E:21	3	R1b,R2b,R3c	X	E:57	3	R1e,R2b,R3c	-8.2
E:22	2	R1b,R2a	-8.7	E:58	2	R1e,R2a	8.0
E:23	1	R1b	X	E:59	1	R1e	-7.9
E:24	2	R1b,R2b	-8.9	E:60	2	R1e,R2b	-8.1
E:25	3	R1c,R2a,R3a	X	E:61	2	R2a,R3a	-8.7
E:26	2	R1c,R3a	-8.8	E:62	1	R3a	-9.7
E:27	3	R1c,R2b,R3a	-8.9	**E:63**	**2**	**R2b,R3a**	**-9.5**
E:28	3	R1c,R2a,R3b	X	E:64	2	R2a,R3b	-9.2
E:29	2	R1c,R3b	X	**E:65**	**1**	**R3b**	**-9.8**
E:30	3	R1c,R2b,R3b	-11.3	**E:66**	**2**	**R2b,R3b**	**-9.4**
E:31	3	R1c,R2a,R3c	X	E:67	2	R2a,R3c	-9.4
E:32	2	R1c,R3c	-9.2	E:68	1	R3c	-8.6
E:33	3	R1c,R2b,R3c	-7.7	E:69	2	R2b,R3c	-8.8
E:34	2	R1c,R2a	-9.3	E:70	1	R2a	-8.4
E:35	1	R1c	-9.0	**NHC-TP**	**0**	**N/A**	**-8.8**
E:36	2	R1c,R2b	-9.4	E:72	1	R2b	-9.1

Table S3. Combinatorial library of NHC-TP analogues and Glide SP ligand docking scores to SARS-COV-2 RdRp active NTP binding site with mutated G10 nucleoside. X denotes ligands that did not fit the list of top 58 ligands are bolded. The NHC-TP unmodified substrate is shown in green font.

Ligand	HPolII Docking (kcal/mol)	PreMD SARS2 RdRp Docking (kcal/mol)	Docking score difference (kcal/mol) RdRp−HPolII	MM-GBSA ΔG_{TOT} (kcal/mol)	Avg. Prot. RMSD (Å)	Avg. Lig. RMSD (Å)	Ligand	HPolII Docking (kcal/mol)	PreMD SARS2 RdRp Docking (kcal/mol)	Docking score difference (kcal/mol) RdRp−HPolII	MM-GBSA ΔG_{TOT} (kcal/mol)	Avg. Prot. RMSD (Å)	Avg. Lig. RMSD (Å)
**NHC−TP**	**−8.4**	**−8.8**	**−0.3**	**−345.3±75**	**2.1**	**2.5**	E:36	−9.2	−9.4	−0.1	−387.9±31	2.4	1.5
E:01	−9.3	−7.5	1.8	−406.9±89	2.3	1.7	E:37	−9.3	−8.7	0.7	−260.4±112	2.3	2.5
E:02	−9.0	X	X	X	X	X	E:38	−9.7	−9.3	0.4	−191.8±15	2.2	3.0
**E:03**	**−9.2**	**−9.3**	**−0.1**	**−433.2±28**	**2.1**	**2.0**	E:39	−9.3	−8.1	1.2	−150.3±17	2.1	4.1
E:04	−9.1	−9.3	−0.3	−426.3±27	2.0	1.4	E:40	−9.9	−8.8	1.1	−195.1±11	2.5	2.2
E:05	−10.1	X	X	X	X	X	E:41	−9.7	−9.0	0.7	−207.4±20	2.5	1.2
E:06	−8.9	−9.7	−0.8	−268.8±9	2.2	1.3	E:42	−9.1	−8.6	0.5	−231.0±89	2.3	2.8
E:07	−9.8	−7.4	−2.4	−262.2±54	2.5	2.0	E:43	−10.7	−9.7	1.0	−161.0±23	9.2	3.9
E:08	−9.2	−8.1	1.1	−419.6±18	2.1	1.8	E:44	−9.6	X	X	X	X	X
E:09	−10.6	X	X	X	X	X	E:45	−9.6	X	X	X	X	X
E:10	−9.2	−8.8	0.5	−366.7±15	2.5	1.9	E:46	−9.3	−8.5	0.9	−410.0±13	2.7	2.1
E:11	−9.6	−9.1	0.5	−288.7±51	2.7	1.9	E:47	−9.1	−8.6	0.5	−387.5±14	2.5	2.2
**E:12**	**−9.3**	**−9.6**	**−0.3**	**−396.4±22**	**2.5**	**2.9**	**E:48**	−9.3	**−9.6**	**−0.4**	**−352.3±20**	**1.8**	**1.6**
E:13	−8.9	X	X	X	X	X	E:49	−8.2	−9.1	−0.9	−375.9±21	2.3	2.5
E:14	−9.5	X	X	X	X	X	E:50	−8.2	−9.5	−1.4	−286.7±97	2.1	2.3
E:15	−9.4	−11.5	−2.2	−333.6±17	45.3	19.8	E:51	−8.4	−9.4	−1.0	−234.3±82	2.5	2.2
E:16	−9.0	−8.1	1.0	−277.8±83	2.5	2.7	E:52	−8.6	−8.5	0.2	−352.9±13	2.2	2.1
E:17	−9.3	−7.8	1.5	−300.0±82	2.0	2.0	**E:53**	**−8.6**	**−9.5**	**−0.8**	**−355.6±50**	**2.3**	**1.5**
E:18	−10.0	−11.3	−1.4	−294.0±31	2.2	1.4	E:54	−8.2	−7.7	0.5	−316.8±24	2.4	2.5
E:19	−9.9	X	X	X	X	X	E:55	−8.8	−9.2	−0.4	−425.7±25	2.4	1.2
E:20	−9.3	−7.6	1.7	−300.2±26	2.6	2.7	E:56	−9.0	−7.6	1.4	−426.0±83	2.1	1.6
E:21	−8.7	X	X	X	X	X	E:57	−8.5	−8.2	0.3	−289.0±103	2.2	1.2
E:22	−8.8	−8.7	0.1	−289.0±91	2.4	2.3	E:58	−8.6	−8.0	0.6	−284.0±22	2.8	2.9
E:23	−9.3	X	X	X	X	X	E:59	−8.5	−7.9	0.6	−409.4±21.9	2.4	2.6
E:24	−9.6	−8.9	0.8	−294.0±92	1.8	2.0	E:60	−8.1	−8.1	0.0	−365.0±22	2.3	2.1
E:25	−9.1	X	X	X	X	X	E:61	−9.2	−8.7	0.5	−328.6±63	2.4	2.9
E:26	−8.7	−8.8	−0.1	−207.7±17	2.0	2.2	E:62	−7.6	−9.7	−2.0	−218.0±54	2.5	1.3
E:27	−9.0	−8.9	0.0	−343.0±33	2.6	2.9	**E:63**	**−8.6**	**−9.5**	**−0.9**	**−408.0±25**	**2.0**	**1.3**
E:28	−9.6	X	X	X	X	X	E:64	−8.6	−9.2	−0.5	−393.0±51	2.6	1.5
E:29	−9.3	X	X	X	X	X	**E:65**	**−9.3**	**−9.8**	**−0.5**	**−364.0±16**	**2.1**	**2.2**
E:30	−9.5	−11.3	−1.8	−302.9±89	2.3	2.4	**E:66**	**−8.2**	**−9.4**	**−1.2**	**−373.0±27**	**2.7**	**1.7**
E:31	−9.2	X	X	X	X	X	E:67	−8.3	−9.4	−1.1	−313.0±31	2.3	2.2
E:32	−9.8	−9.2	0.6	−352.2±51	2.4	1.5	E:68	−8.9	−8.6	−0.8	−276.0±78	2.3	2.4
E:33	−9.4	−7.7	1.8	−384.0±42	2.7	2.8	E:69	−8.5	−8.8	−0.3	−180.0±80	8.1	4.2
E:34	−9.4	−9.3	0.1	−300.8±21	2.4	1.8	E:70	−8.5	−8.4	0.1	−414.0±19	3.0	1.1
E:35	−9.7	−9.0	0.6	−332.2±63	3.3	1.9	E:72	−8.2	−9.1	−0.8	−311.0±27	2.1	2.4

Table S4. Glide docking of enumerated ligands to SARS-COV-2 RdRp and Human PolII results along with MD simulation results of 58 SARS-COV-2 RdRp systems. X denotes ligands that did not dock to the SARS-COV-2 RdRp active site. The binding free energy prediction performed before (Glide SP score) and after (MM-GBSA) MD production runs, and the difference in binding free energy (ΔG* difference (kcal/mol) = SARS-COV-2 RdRp− HPolII). The RMSD values were averaged over last 50 ns of simulation trajectory. The top 7 Ligands are bolded.

Ligand	Post-MD H-Bond Distance 1’(Å)	SP Dock H-Bond Distance 1’(Å)	Post-MD H-Bond Distance 2’(Å)	SP Dock H-Bond Distance 2’(Å)	Post-MD H-Bond Distance 3’(Å)	SP Dock H-Bond Distance 3’(Å)
NHC-TP	4.53	4.81	5.27	5.44	6.90	7.15
E:63	2.64	2.76	2.99	2.98	3.65	2.91
E:12	6.00	8.42	5.09	5.53	4.78	2.60
E:66	2.92	2.89	2.94	2.99	2.69	2.79
E:65	2.67	2.93	2.86	2.81	2.95	3.04
E:53	2.73	2.74	2.99	2.93	3.00	2.74
E:48	5.23	6.66	5.88	5.94	8.04	7.30
E:03	3.12	2.88	2.87	3.00	2.84	2.94

Table S5. Hydrogen bond distances between positions 1’, 2’ and 3’ of NHC-TP and G10 in the RNA template strand in the most abundant MD cluster structure pose and pre-MD SP docking pose. See **Figure S9** for the graphic representations. Green font represents hydrogen bond distances improved for post-MD poses.

Ligand	Consensus Log P	ESOL Log S	Ali Log S	Silicos−IT LogSw	Average LogS	GI absorption	CYP1A2 inhibitor	CYP2C19 inhibitor	CYP2C9 inhibitor	CYP2D6 inhibitor	CYP3A4 inhibitor	Lipinski #violations	Synthetic Accessibility
**RDV**	**1.53**	**−4.12**	**−6.01**	**−4.77**	**−4.97**	**Low**	**No**	**No**	**No**	**No**	**Yes**	**No: 2 violations: MW>500, NorO>10**	**6.33**
**NHC**	**−1.08**	**−0.83**	**−1.17**	**0.12**	**−0.63**	**Low**	**No**	**No**	**No**	**No**	**No**	**Yes; 0 violation**	**4.49**
E:01	0.07	−2.3	−3.12	−2.88	−2.77	Low	No	No	No	No	No	Yes; 1 violation: NorO>10	5.13
E:02	0.54	−2.43	−3.05	−2.59	−2.69	Low	No	No	No	No	No	Yes; 0 violation	5.05
**E:03**	**0.95**	**−2.63**	**−3.25**	**−3.19**	**−3.02**	**Low**	**No**	**No**	**No**	**No**	**No**	**Yes; 0 violation**	**5.16**
E:04	0.75	−2.63	−3.35	−3.66	−3.21	Low	No	No	No	No	Yes	Yes; 1 violation: NorO>10	5.24
E:05	0.98	−2.76	−3.29	−3.38	−3.14	High	No	No	No	No	No	Yes; 0 violation	5.14
E:06	1.09	−2.96	−3.48	−3.97	−3.47	High	No	No	No	No	Yes	Yes; 0 violation	5.25
E:07	1.36	−3.06	−3.75	−4.34	−3.72	Low	No	No	No	No	Yes	Yes; 0 violation	5.34
E:08	1.58	−3.36	−4.12	−3.79	−3.76	High	No	No	No	No	Yes	Yes; 0 violation	5.34
E:09	1.61	−3.56	−4.32	−4.38	−4.09	High	No	No	No	No	Yes	Yes; 0 violation	5.46
E:10	0.36	−2.47	−3.26	−2.66	−2.8	Low	No	No	No	No	No	Yes; 1 violation: NorO>10	5.01
E:11	0.43	−2.6	−3.2	−2.38	−2.73	Low	No	No	No	No	No	Yes; 0 violation	4.92
**E:12**	**0.83**	**−2.79**	**−3.39**	**−2.97**	**−3.05**	**Low**	**No**	**No**	**No**	**No**	**No**	**Yes; 0 violation**	**5.04**
E:13	0.46	−2.47	−3.22	−3.14	−2.94	Low	No	No	No	No	No	Yes; 1 violation: NorO>10	5.13
E:14	0.66	−2.59	−3.16	−2.85	−2.87	Low	No	No	No	No	No	Yes; 0 violation	5.03
E:15	1.25	−2.79	−3.35	−3.45	−3.2	Low	No	No	No	No	No	Yes; 0 violation	5.15
E:16	0.95	−2.8	−3.46	−3.92	−3.39	Low	No	No	No	No	Yes	Yes; 1 violation: NorO>10	5.25
E:17	1.11	−2.93	−3.39	−3.64	−3.32	High	No	No	No	No	Yes	Yes; 0 violation	5.15
E:18	1.55	−3.13	−3.59	−4.23	−3.65	High	No	No	No	No	Yes	Yes; 0 violation	5.26
E:19	1.62	−3.4	−4.29	−4.32	−4	Low	No	No	No	No	Yes	No: 2 violations: MW>500, NorO>10	5.45
E:20	1.78	−3.52	−4.22	−4.05	−3.93	High	No	No	No	No	Yes	Yes; 0 violation	5.35
E:21	2.19	−3.72	−4.42	−4.64	−4.26	High	No	No	No	No	Yes	Yes; 0 violation	5.47
E:22	0.71	−2.64	−3.37	−2.92	−2.98	Low	No	No	No	No	No	Yes; 1 violation: NorO>10	5.01
E:23	0.98	−2.76	−3.3	−2.64	−2.9	Low	No	No	No	No	No	Yes; 0 violation	4.91
E:24	1.47	−2.96	−3.49	−3.23	−3.23	Low	No	No	No	No	No	Yes; 0 violation	5.03
E:25	−0.07	−2.26	−3.33	−2.94	−2.84	Low	No	No	No	No	No	Yes; 1 violation: NorO>10	5.18
E:26	0.14	−2.39	−3.26	−2.66	−2.77	Low	No	No	No	No	No	Yes; 1 violation: NorO>10	5.12
E:27	0.77	−2.59	−3.46	−3.25	−3.1	Low	No	No	No	No	No	Yes; 1 violation: NorO>10	5.23
E:28	0.35	−2.6	−3.56	−3.72	−3.29	Low	No	No	No	No	Yes	Yes; 1 violation: NorO>10	5.29
E:29	0.7	−2.72	−3.5	−3.44	−3.22	Low	No	No	No	No	Yes	Yes; 1 violation: NorO>10	5.21
E:30	0.9	−2.92	−3.69	−4.03	3.55	Low	No	No	No	No	Yes	Yes; 1 violation: NorO>10	5.32
E:31	1.01	−3.2	−4.39	−4.12	−3.9	Low	No	No	No	No	Yes	No: 2 violations: MW>500, NorO>10	5.49
E:32	1.24	−3.32	−4.33	−3.85	−3.83	Low	No	No	No	No	Yes	Yes; 1 violation: NorO>10	5.41
E:33	1.53	−3.52	−4.52	−4.44	−4.16	Low	No	No	No	No	Yes	Yes; 1 violation: NorO>10	5.53
E:34	0.03	−2.43	−3.47	−2.72	−2.87	Low	No	No	No	No	No	Yes; 1 violation: NorO>10	5.06
E:35	0.11	−2.56	−3.41	−2.44	−2.8	Low	No	No	No	No	No	Yes; 1 violation: NorO>10	5
E:36	0.65	−2.75	−3.6	−3.03	−3.13	Low	No	No	No	No	No	Yes; 1 violation: NorO>10	5.11
E:37	0.43	−2.47	−3.22	−3.14	−2.94	Low	No	No	No	No	No	Yes; 1 violation: NorO>10	5.17
E:38	0.89	−2.59	−3.16	−2.85	−2.9	Low	No	No	No	No	No	Yes; 0 violation	5.08
E:39	1.28	−2.79	−3.35	−3.45	−3.2	Low	No	No	No	No	No	Yes; 0 violation	5.2
E:40	1.17	−2.8	−3.46	−3.92	−3.39	Low	No	No	No	No	Yes	Yes; 1 violation: NorO>10	5.29
E:41	1.01	−2.93	−3.39	−3.64	−3.32	High	No	No	No	No	Yes	Yes; 0 violation	5.2
E:42	1.75	−3.13	−3.59	−4.23	−3.65	High	No	No	No	No	Yes	Yes; 0 violation	5.31
E:43	1.52	−3.4	−4.29	−4.32	−4	Low	No	No	No	No	Yes	No: 2 violations: MW>500, NorO>10	5.49
E:44	1.72	−3.52	−4.22	−4.05	−3.93	High	No	No	No	No	Yes	Yes; 0 violation	5.4
E:45	2.26	−3.72	−4.42	−4.64	−4.26	High	No	No	No	No	Yes	Yes; 0 violation	5.52
E:46	0.85	−2.64	−3.37	−2.92	−2.98	Low	No	No	No	No	No	Yes; 1 violation: NorO>10	5.06
E:47	0.9	−2.76	−3.3	−2.64	−2.9	Low	No	No	No	No	No	Yes; 0 violation	4.97

Ligand	Consensus Log P	ESOL Log S	Ali Log S	Silicos−IT LogSw	Average LogS	GI absorption	CYP1A2 inhibitor	CYP2C19 inhibitor	CYP2C9 inhibitor	CYP2D6 inhibitor	CYP3A4 inhibitor	Lipinski #violations	Synthetic Accessibility
**E:48**	**1.25**	**−2.96**	**−3.49**	**−3.23**	**−3.23**	**Low**	**No**	**No**	**No**	**No**	**No**	**Yes; 0 violation**	**5.08**
E:49	−0.95	−0.82	−1.53	−0.78	−1.04	Low	No	No	No	No	No	Yes; 1 violation: NorO>10	4.78
E:50	−0.69	−0.95	−1.46	−0.49	−0.97	Low	No	No	No	No	No	Yes; 0 violation	4.7
E:51	−0.53	−1.15	−1.66	−1.09	−1.3	Low	No	No	No	No	No	Yes; 0 violation	4.81
E:52	−0.69	−1.16	−1.76	−1.57	−1.5	Low	No	No	No	No	No	Yes; 1 violation: NorO>10	4.87
**E:53**	**−0.4**	**−1.28**	**−1.7**	**−1.28**	**−1.42**	**Low**	**No**	**No**	**No**	**No**	**No**	**Yes; 0 violation**	**4.77**
E:54	−0.28	−1.48	−1.9	−1.88	−1.75	Low	No	No	No	No	No	Yes; 0 violation	4.88
E:55	0.05	−1.76	−2.59	−1.98	−2.11	Low	No	No	No	No	No	Yes; 1 violation: NorO>10	5.06
E:56	0.44	−1.88	−2.53	−1.7	−2.04	High	No	No	No	No	No	Yes; 0 violation	4.96
E:57	0.68	−2.08	−2.73	−2.29	−2.4	High	No	No	No	No	No	Yes; 0 violation	5.08
E:58	−0.68	−0.99	−1.68	−0.56	−1.08	Low	No	No	No	No	No	Yes; 1 violation: NorO>10	4.65
E:59	−0.6	−1.12	−1.61	−0.28	−1	Low	No	No	No	No	No	Yes; 0 violation	4.57
E:60	−0.21	−1.31	−1.8	−0.87	−1.33	Low	No	No	No	No	No	Yes; 0 violation	4.68
E:61	−1.23	−0.69	−1.33	−0.39	−0.8	Low	No	No	No	No	No	Yes; 1 violation: NorO>10	4.68
E:62	−1.06	−0.66	−1.02	−0.1	−0.59	Low	No	No	No	No	No	Yes; 0 violation	4.62
**E:63**	**−0.69**	**−0.67**	**−0.89**	**−0.69**	**−0.75**	**Low**	**No**	**No**	**No**	**No**	**No**	**Yes; 0 violation**	**4.73**
E:64	−0.7	−1.03	−1.57	−1.17	−1.26	Low	No	No	No	No	No	Yes; 1 violation: NorO>10	4.77
**E:65**	**−0.58**	**−1**	**−1.25**	**−0.89**	**−1.05**	**Low**	**No**	**No**	**No**	**No**	**No**	**Yes; 0 violation**	**4.69**
**E:66**	**−0.24**	**−1**	**−1.13**	**−1.48**	**−1.2**	**Low**	**No**	**No**	**No**	**No**	**No**	**Yes; 0 violation**	**4.81**
E:67	−0.13	−1.63	−2.4	−1.59	−1.87	Low	No	No	No	No	No	Yes; 1 violation: NorO>10	4.96
E:68	−0.12	−1.6	−2.08	−1.3	−1.66	High	No	No	No	No	No	Yes; 0 violation	4.88
E:69	0.21	−1.6	−1.96	−1.9	−1.82	High	No	No	No	No	No	Yes; 0 violation	5
E:70	−1.18	−0.86	−1.48	−0.17	−0.84	Low	No	No	No	No	No	Yes; 1 violation: NorO>10	4.55
E:72	−0.69	−0.83	−1.03	−0.48	−0.78	Low	No	No	No	No	No	Yes; 0 violation	4.6

Table S6. Summary of predicted ADME properties of the 72 NHC-TP ligands in their prodrug form with prodrug form of RDV-TP and NHC-TP being set as the reference.

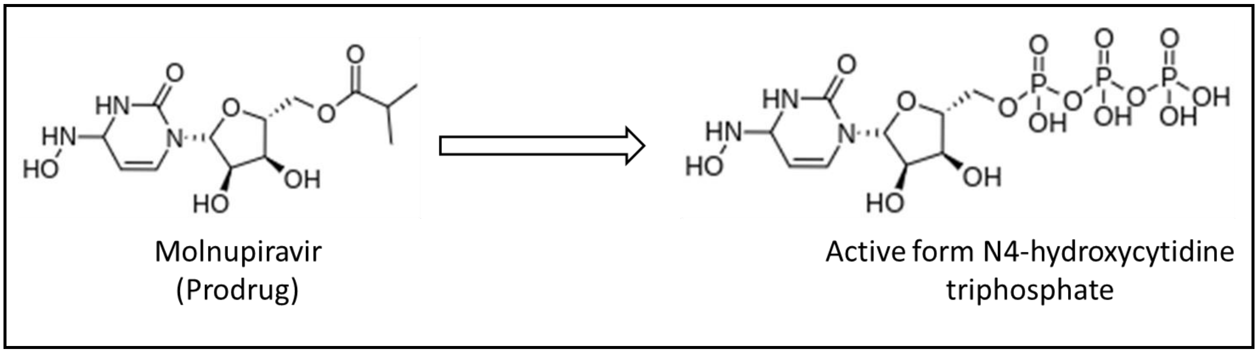

Figure S1. Chemical structures of prodrug Molnupiravir and its biochemically active form N4-hydroxycytidine triphosphate (NHC-TP).

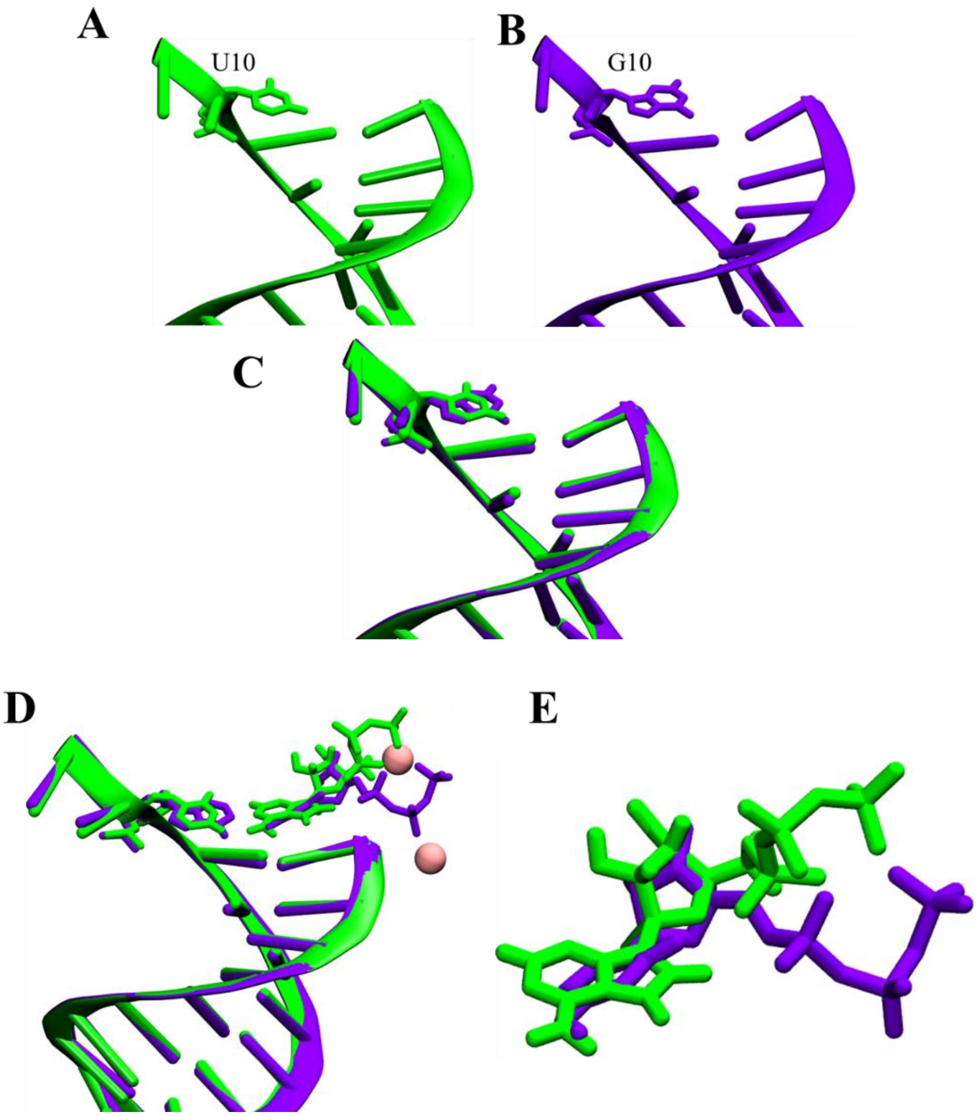

Figure S2. (**A**) Cryo-EM crystal structure of 7BV2 template-primer RNA with unpaired template uracil 10 (U10) shown in licorice representation (green). (**B**) Structure of modified crystal template-primer RNA with G10 mutation shown in licorice representation (purple). (**C**) Superimposed conformational comparison of both crystal template-primer RNA containing nucleobases U10 (green) and G10 (purple). (**D**) Superimposed conformational comparison of crystal template-primer RNA containing nucleobases U10 interacting with RDV-TP (green) and G10 interacting with NHC-TP (purple). (**E)** Superimposed conformational comparison of RDV-TP (green) and NHC-TP (purple).

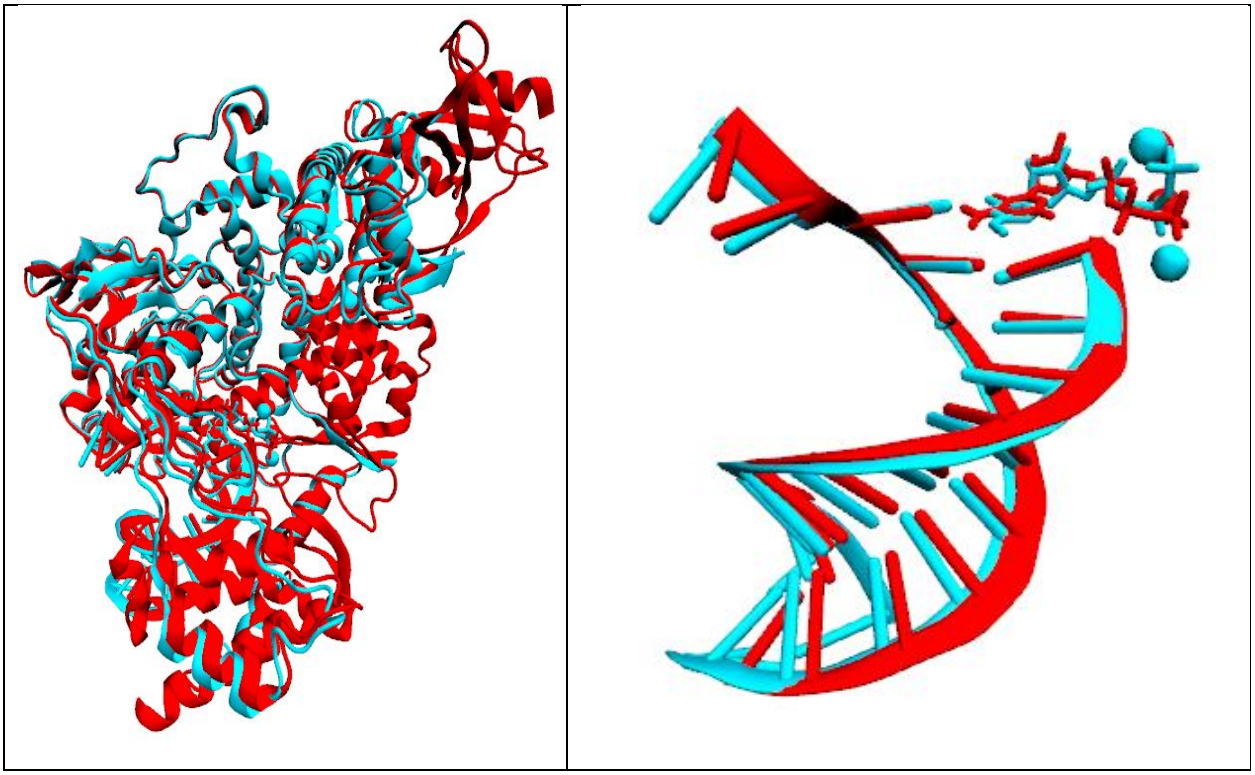

Figure S3. Structure comparison of modified 7BV2 and 7AAP NTP entry structures. 7AAP structure (red) and modified 7BV2 (cyan)

**NHC-TP**	**E:01**	**E:02**
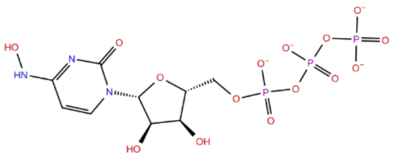	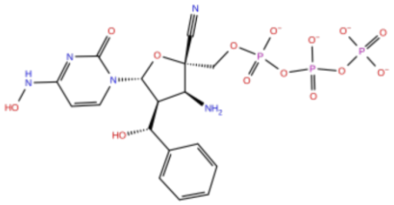	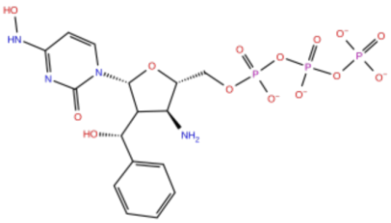
**E:03**	**E:04**	**E:05**
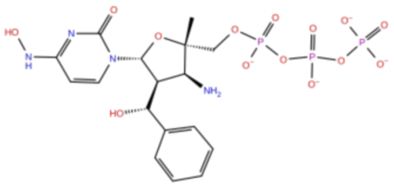	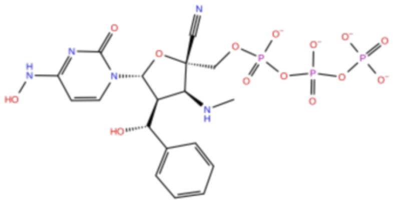	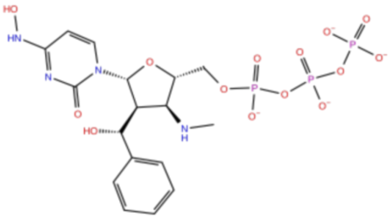
**E:06**	**E:07**	**E:08**
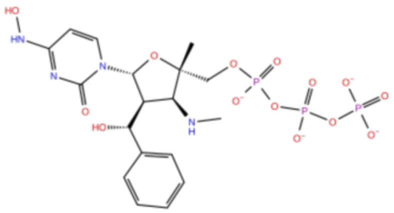	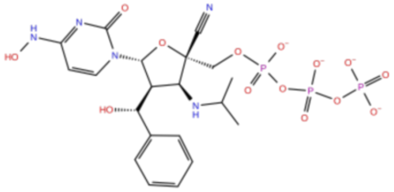	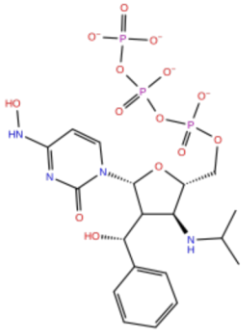
**E:09**	**E:10**	**E:11**
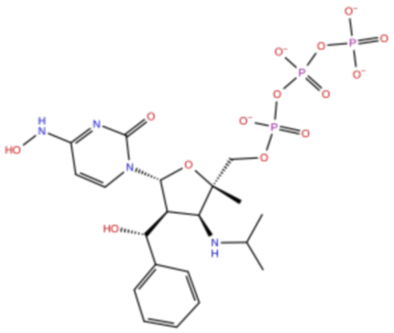	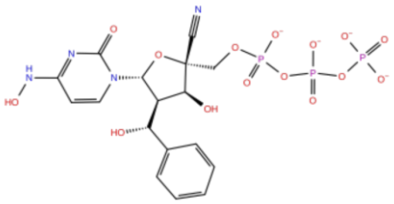	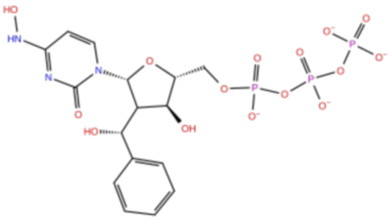

**E:12**	**E:13**	**E:14**
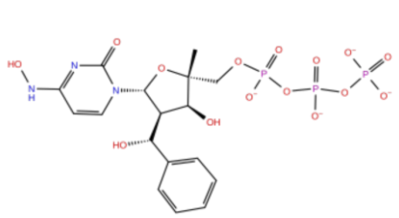	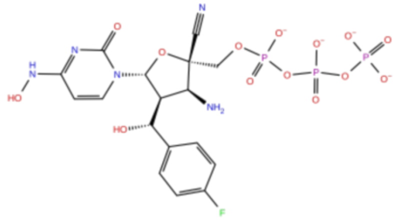	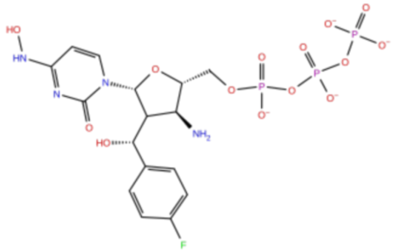
**E:15**	**E:16**	**E:17**
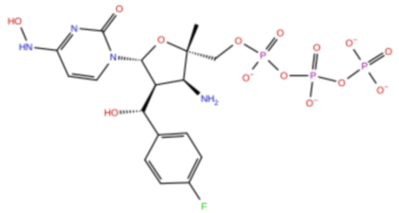	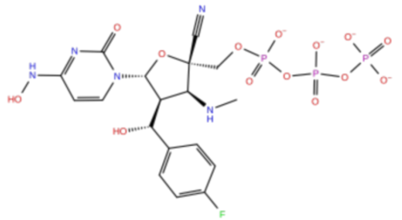	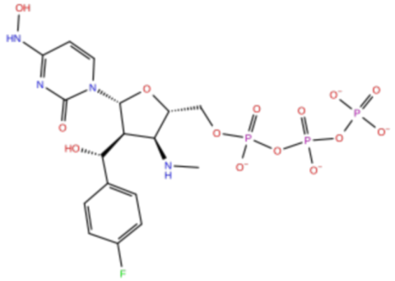
**E:18**	**E:19**	**E:20**
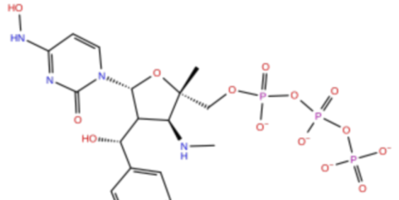	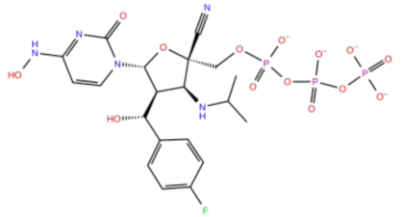	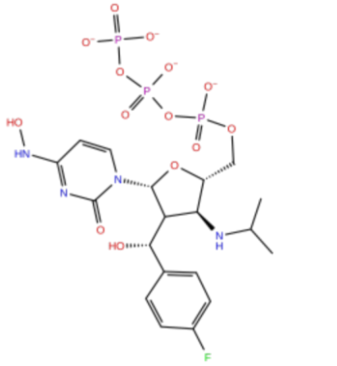
**E:21**	**E:22**	**E:23**
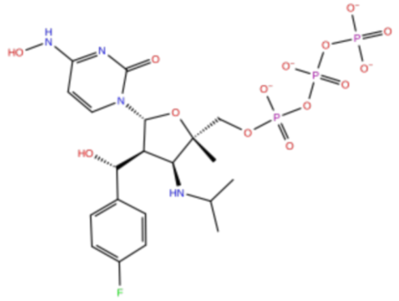	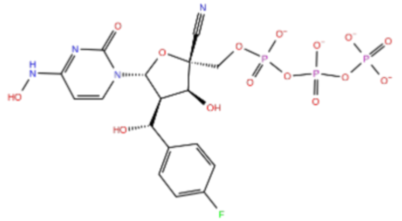	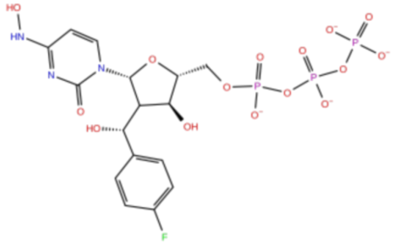

E:24	E:25	E:26
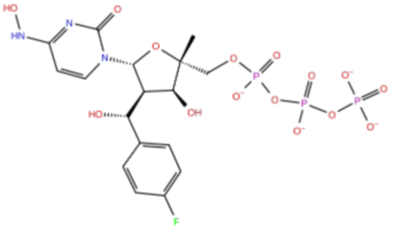	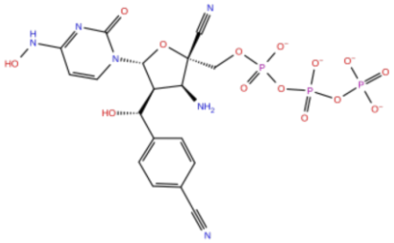	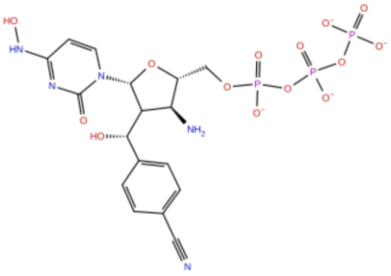
**E:27**	**E:28**	**E:29**
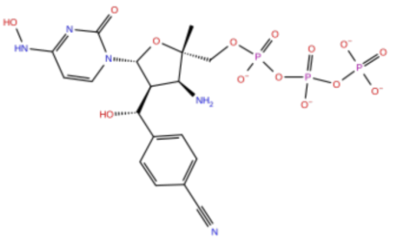	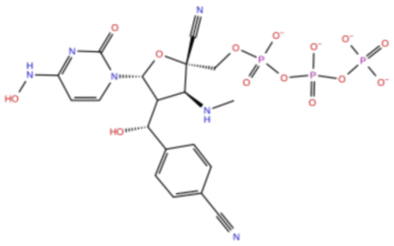	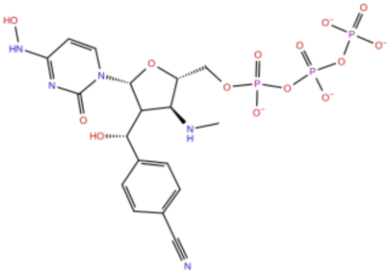
**E:30**	**E:31**	**E:32**
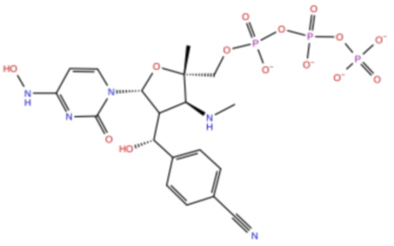	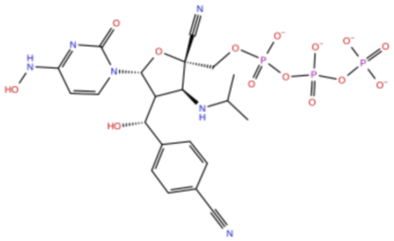	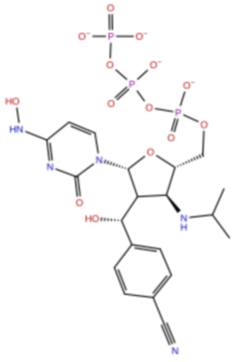
**E:33**	**E:34**	**E:35**
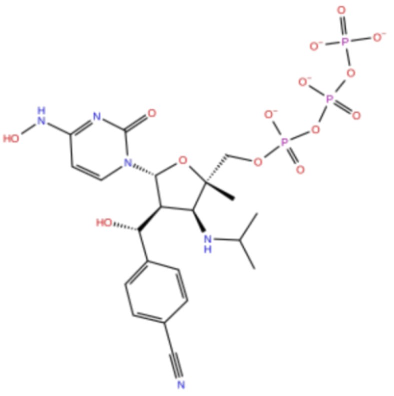	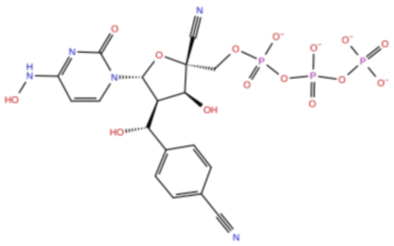	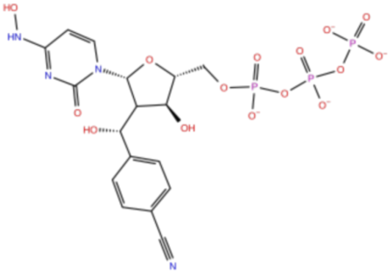

**E:36** 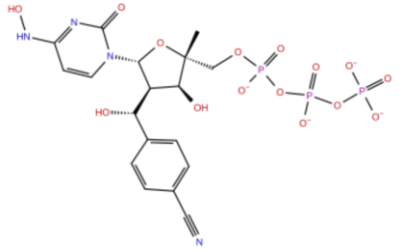	**E:37** 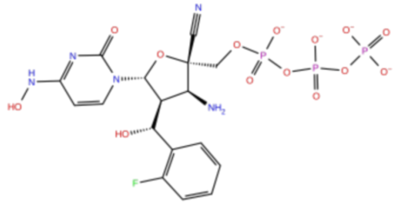	**E:38** 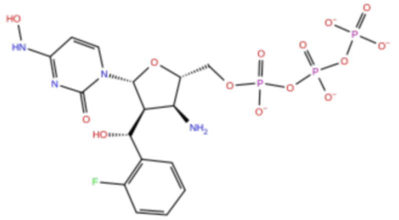
**E:39** 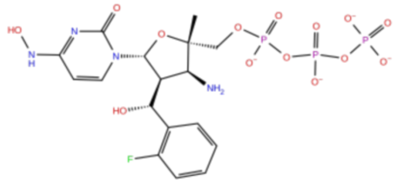	**E:40** 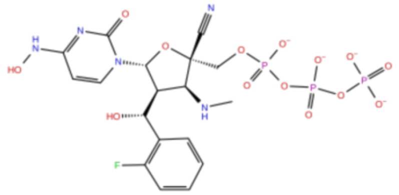	**E:41** 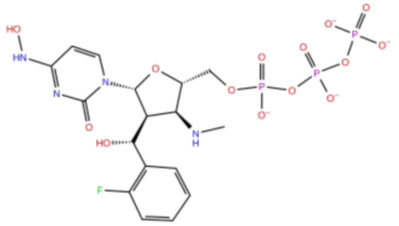
**E:42** 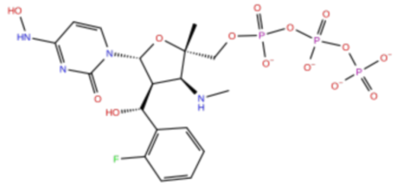	**E:43** 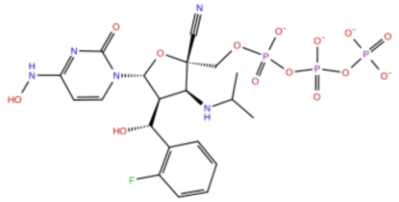	**E:44** 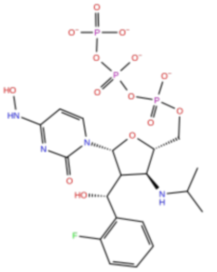
**E:45** 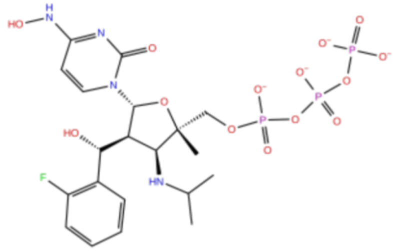	**E:46** 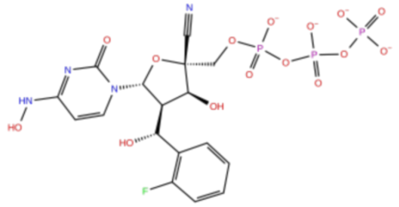	**E:47** 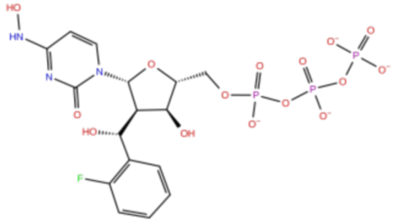
**E:48** 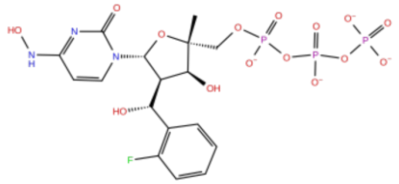	**E:49** 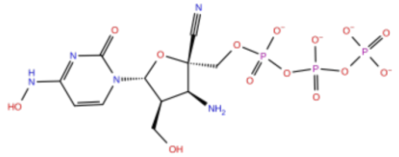	**E:50** 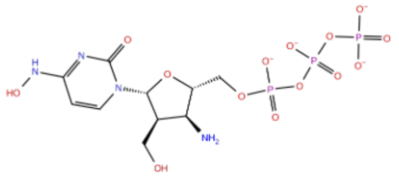

**E:51**<br>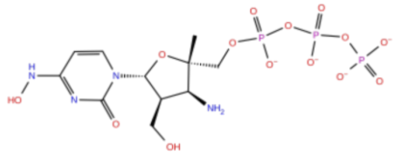	**E:52**<br>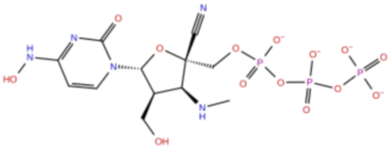	**E:53**<br>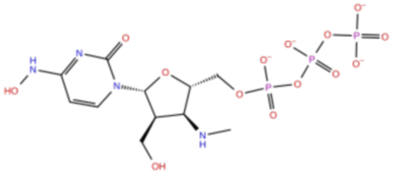
**E:54**<br>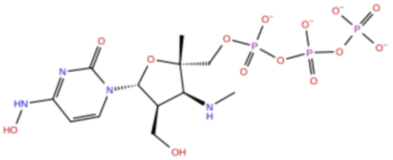	**E:55**<br>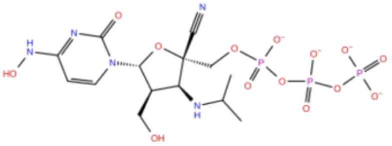	**E:56**<br>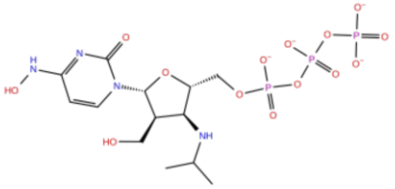
**E:57**<br>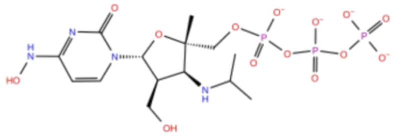	**E:58**<br>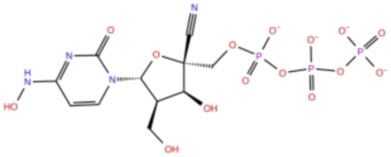	**E:59**<br>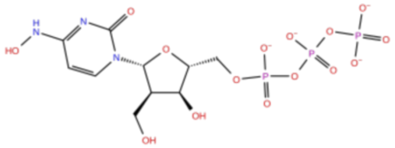
**E:60**<br>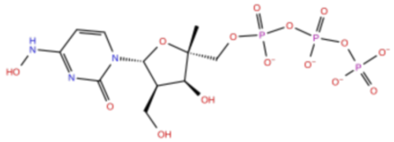	**E:61**<br>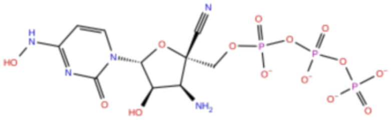	**E:62**<br>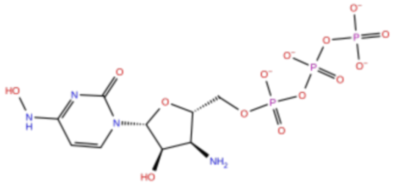
**E:63**<br>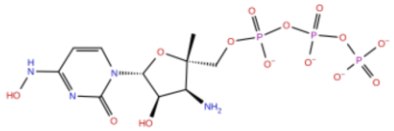	**E:64**<br>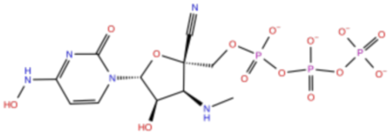	**E:65**<br>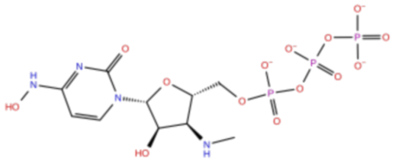
**E:66**<br>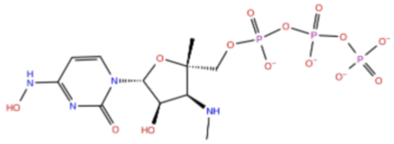	**E:67**<br>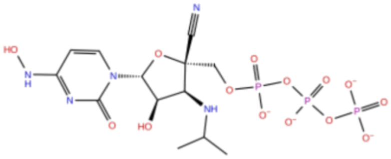	**E:68**<br>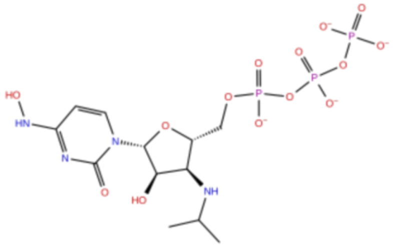

E:69	E:70	NHC-TP (E:71)
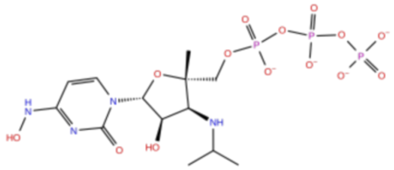	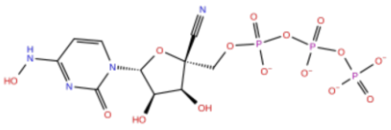	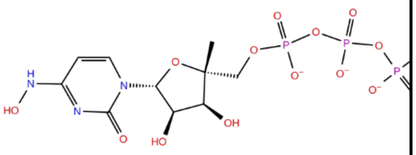

Figure S4. Chemical structures of NHC-TP analogue library and reference structure NHC-TP (E:71).

NHC-TP

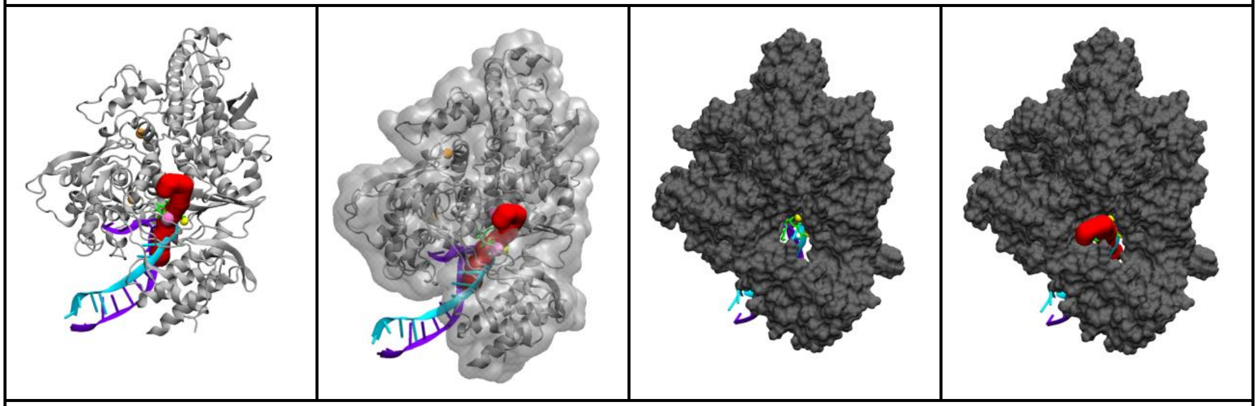

E:03

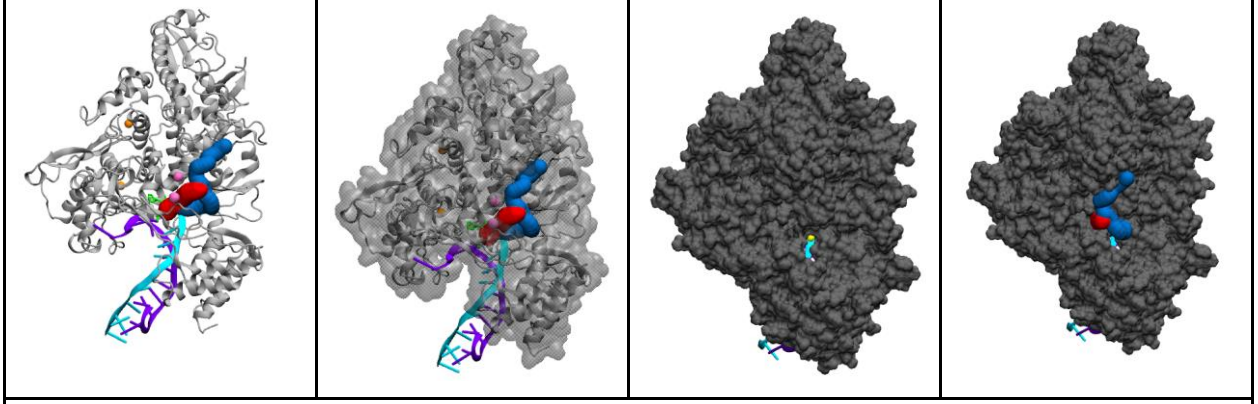

E:12

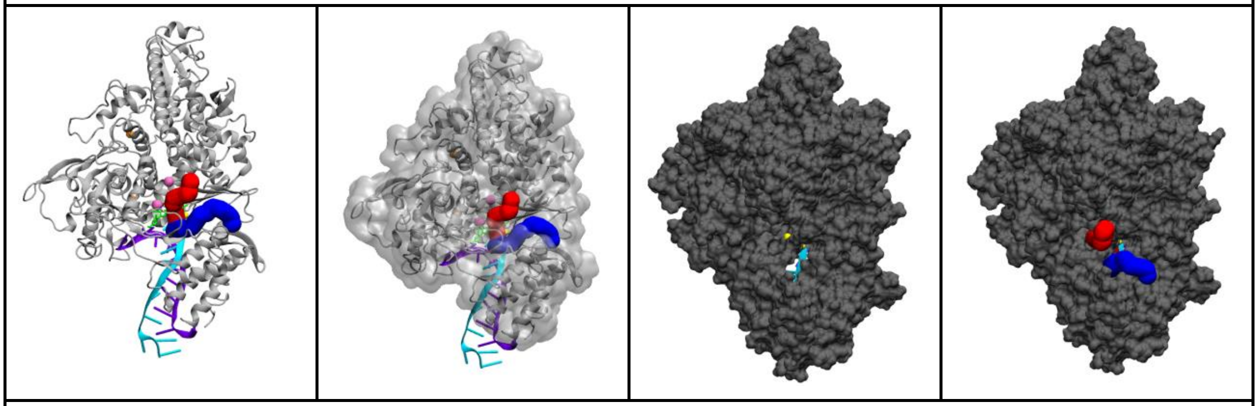

E:48

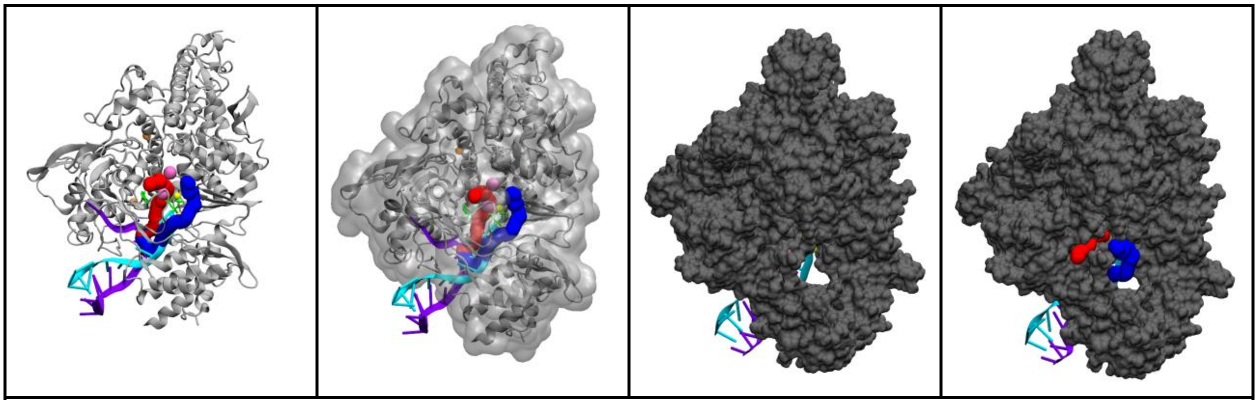

E:53

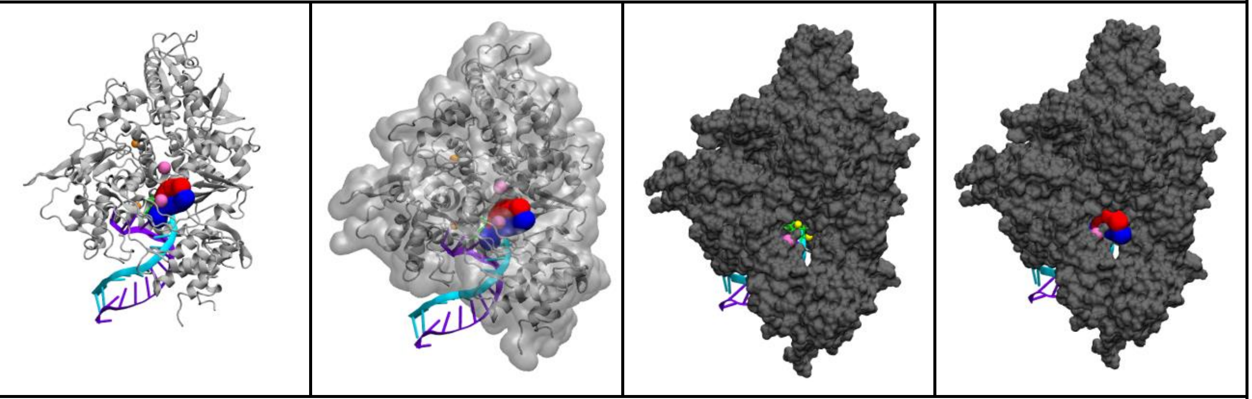

E:63

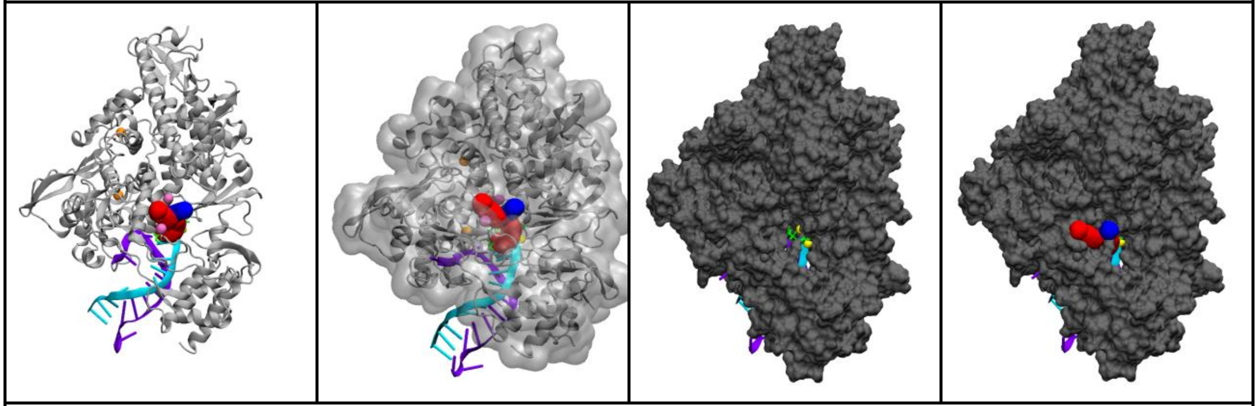

E:65

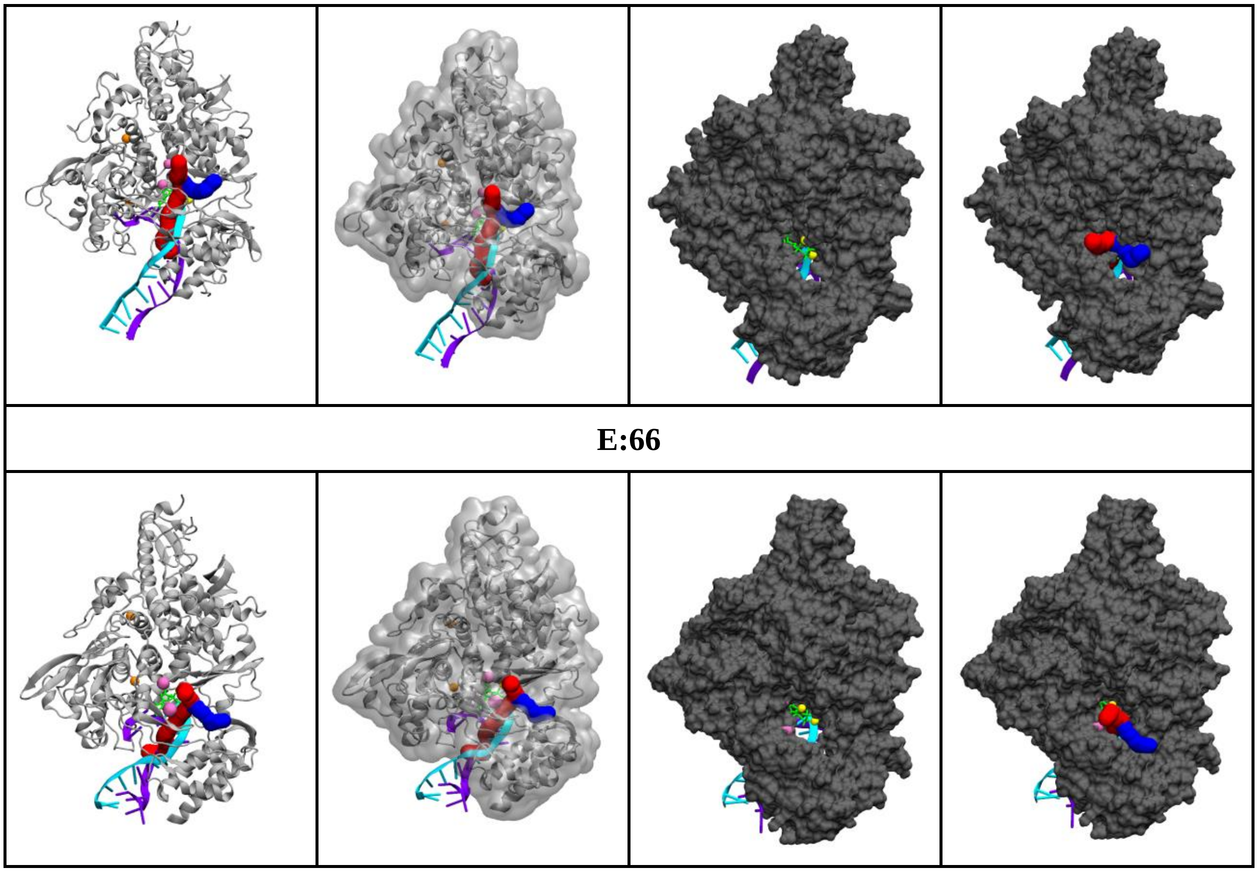

Figure S5. Caver analysis of SARS-COV-2 RdRp. Left ribbon representation of SARS-COV-2 RdRp protein (silver) with template RNA strand (magenta) and product strand (cyan), enumerate ligands (green) and Zn^{2+} (orange) and Mg^{2+} (purple) with calculated channel (red). Middle ribbon and transparent surface representation showing the channel through the protein's surface. Right, surface representation (gray) and calculated entry channel for nucleotide incorporation (red).

<table>
<tr><td colspan="3">NHC-TP</td></tr>
<tr><td>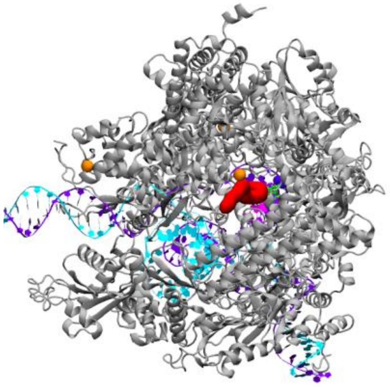</td><td>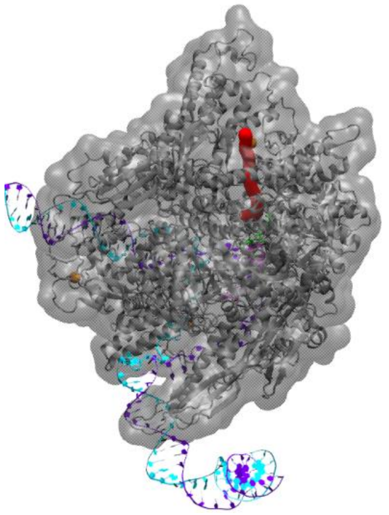</td><td>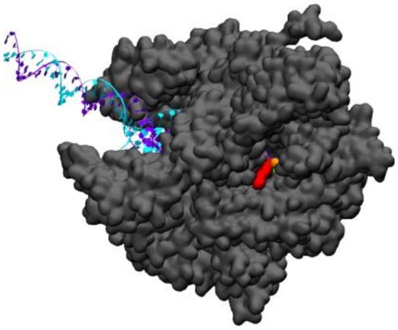</td></tr>
<tr><td colspan="3">E:03</td></tr>
<tr><td>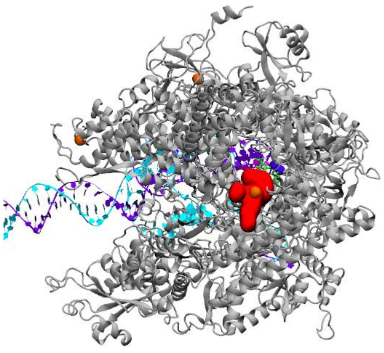</td><td>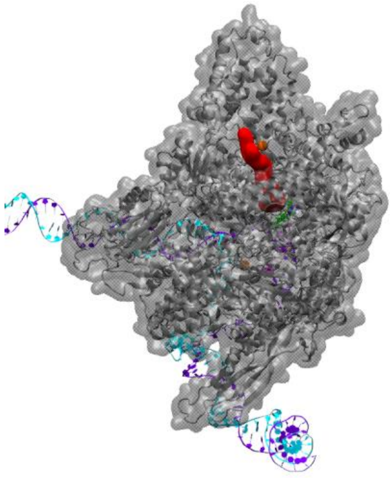</td><td>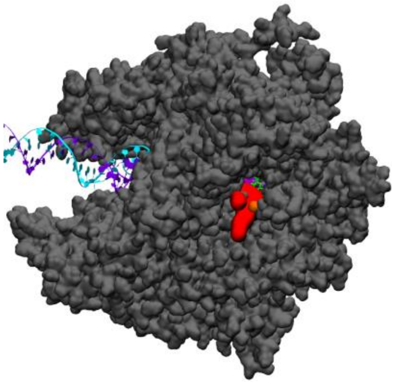</td></tr>
<tr><td colspan="3">E:12</td></tr>
</table>

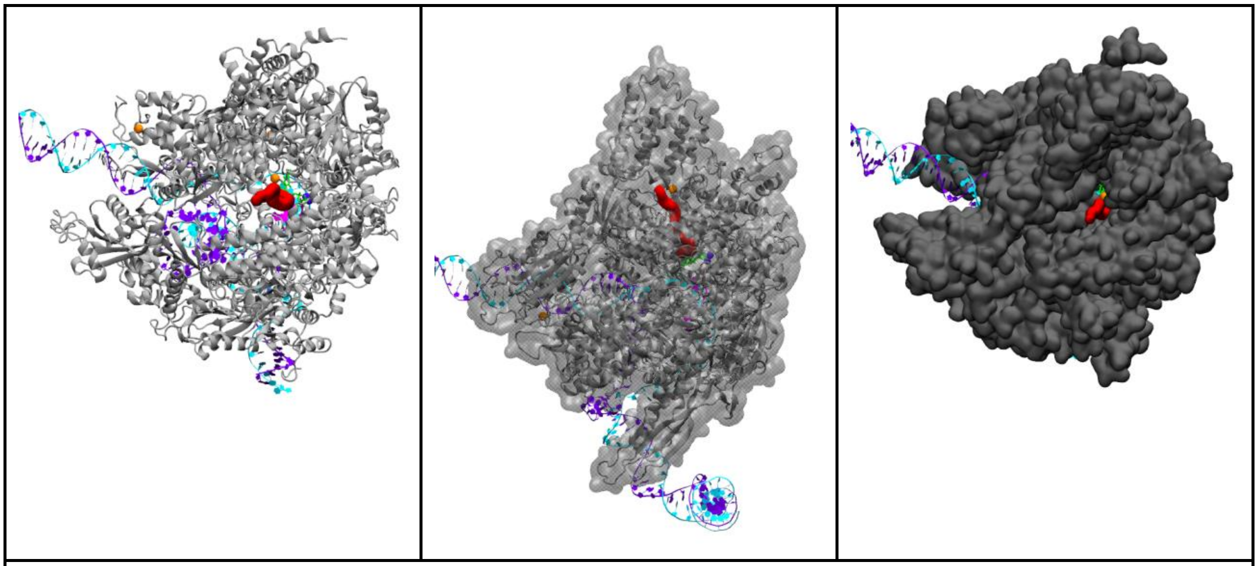

E:48

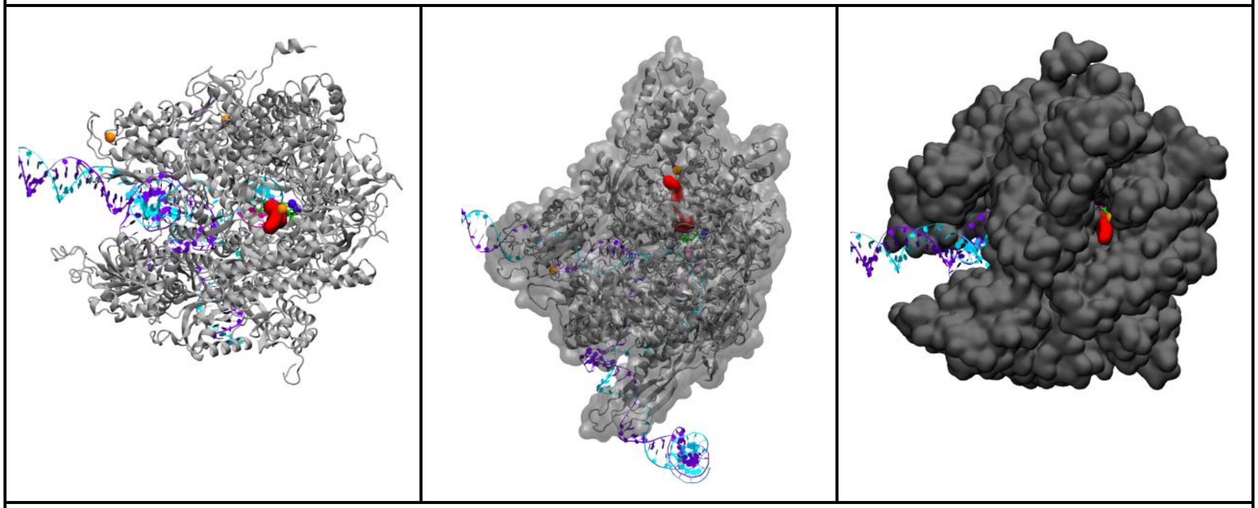

E:58

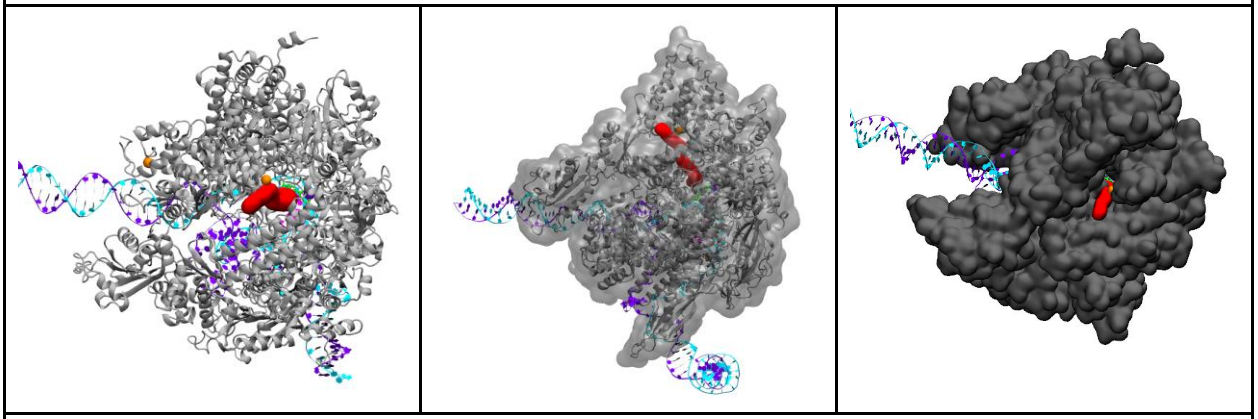

E:63

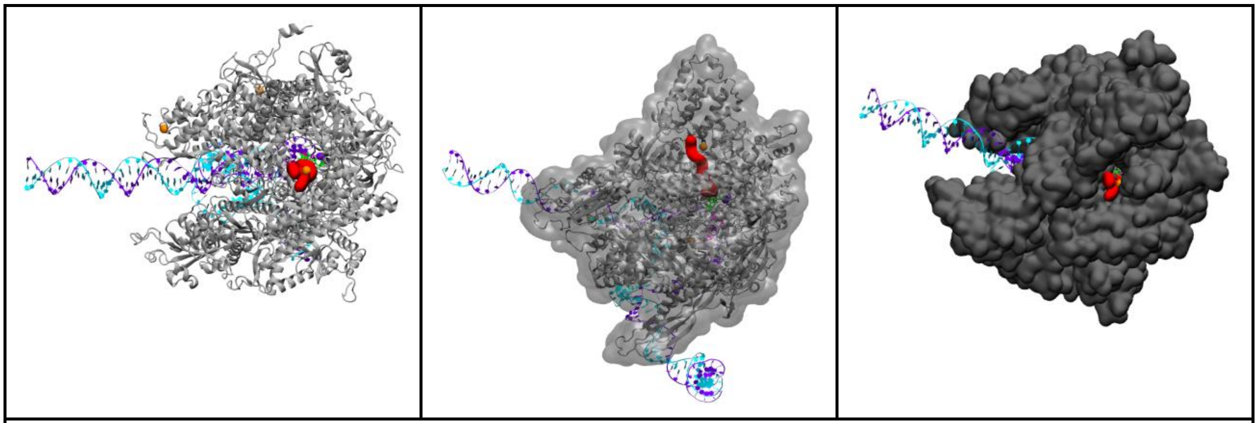

E:65

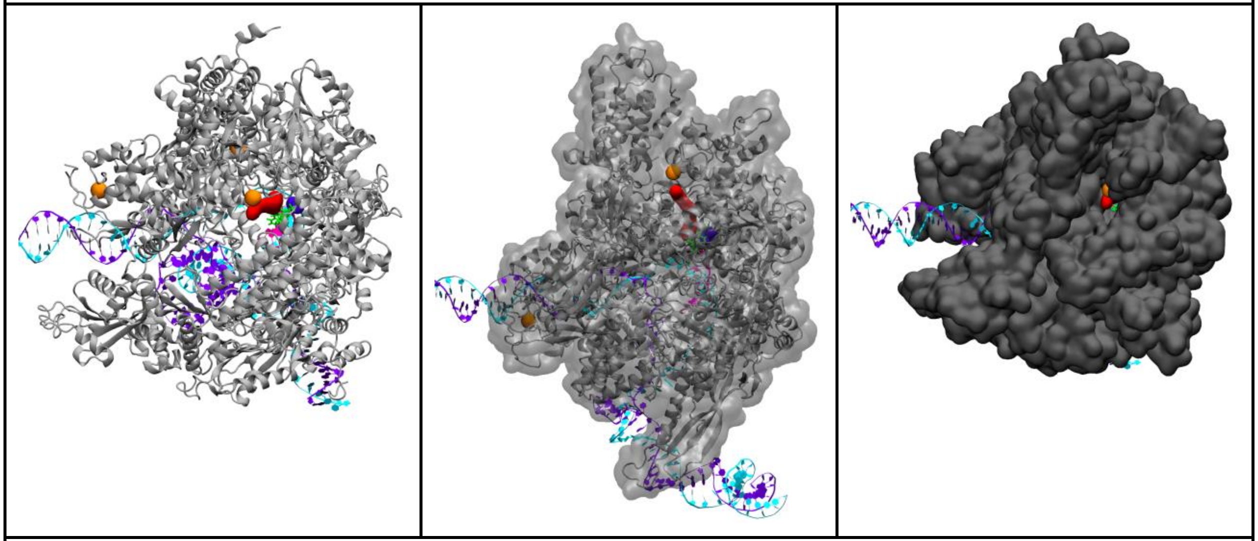

E:66

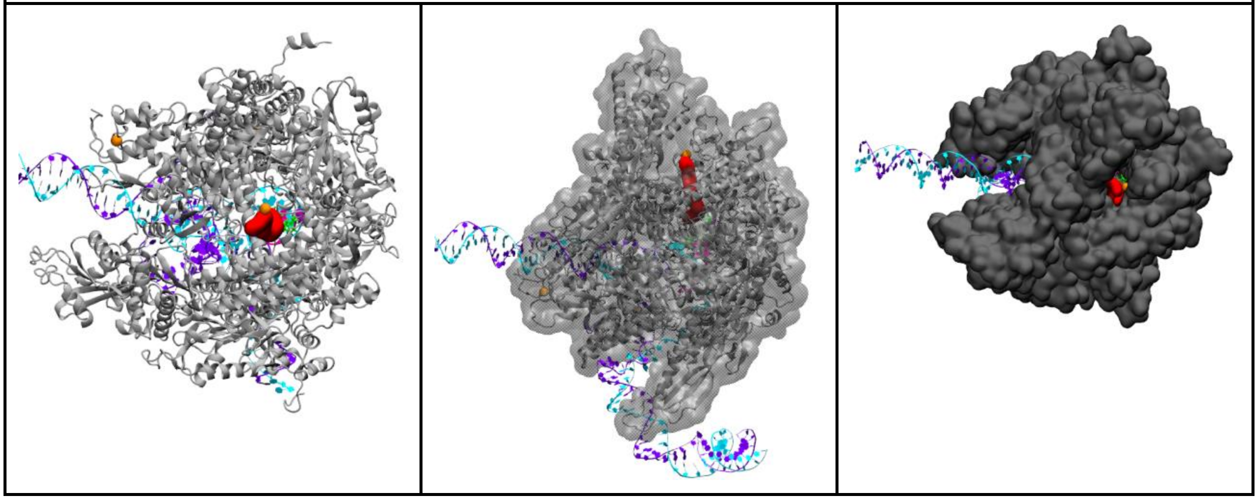

Figure S6. Caver analysis of Human PolII (PDBID: 5IYD) in complex with the top 7 Enumerate ligands. (Left) Cartoon representation of human PolII protein (silver) in-complex with template RNA strand (magenta) and product strand (cyan), enumerate ligands (green) and Zn^{2+} (orange) and Mg^{2+} (purple) with calculated channel (red). Middle ribbon and transparent surface representation showing the channel through the protein's surface. Right, surface representation (gray) and calculated entry channel for nucleotide incorporation (red).

Ligand	Interaction Diagram	Ligand	Interaction Diagram
E:71 (NHC-TP)	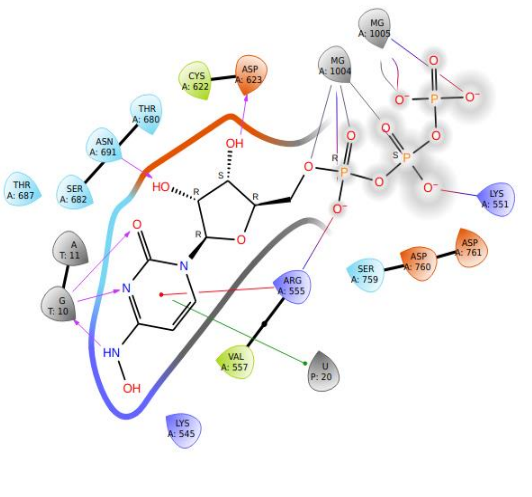	E:01	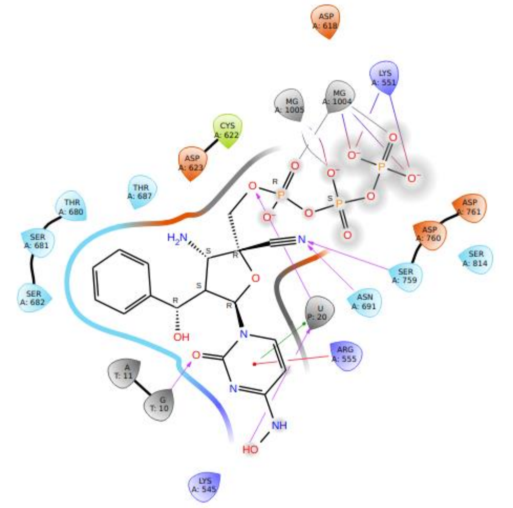
E:03	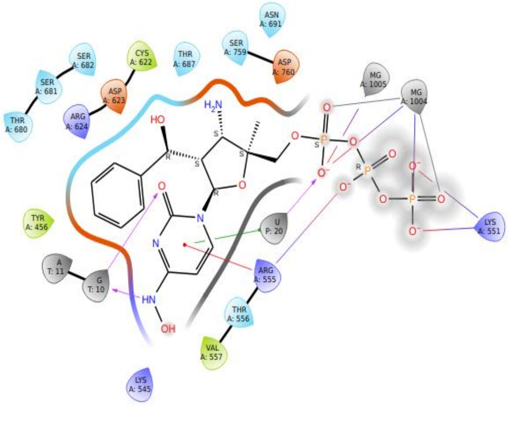	E:04	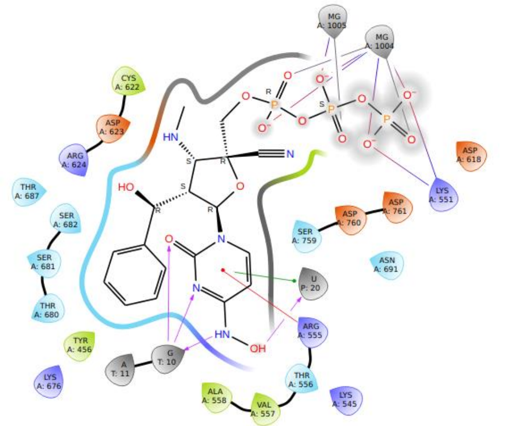
E:06	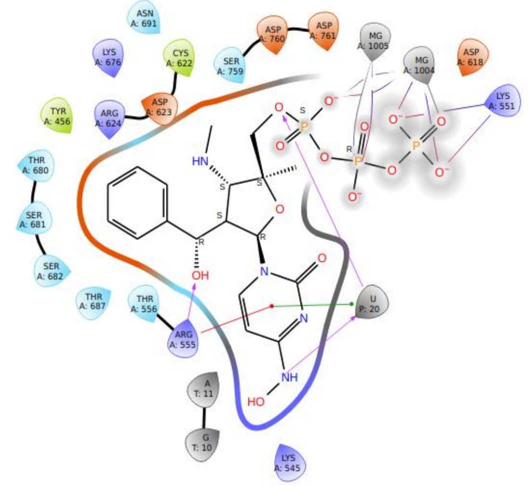	E:07	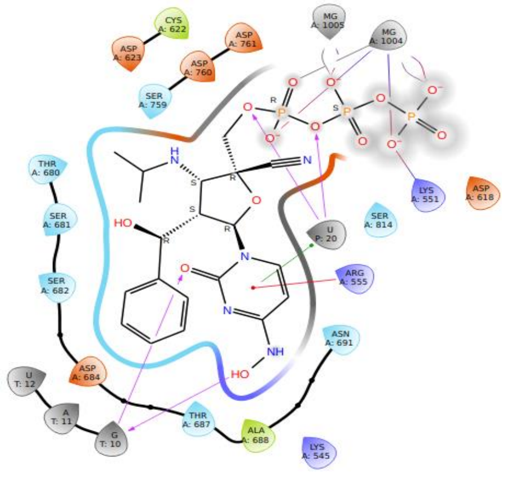

E:08	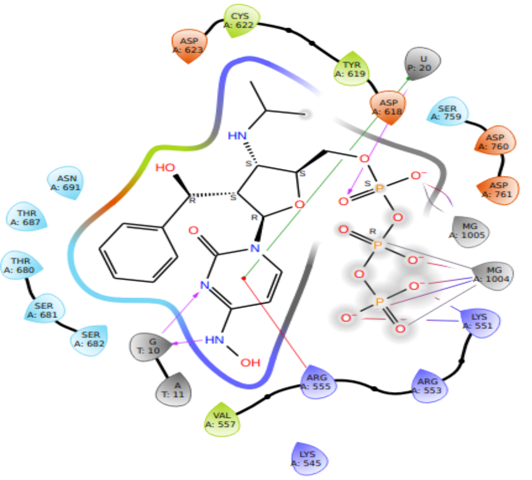	E:10	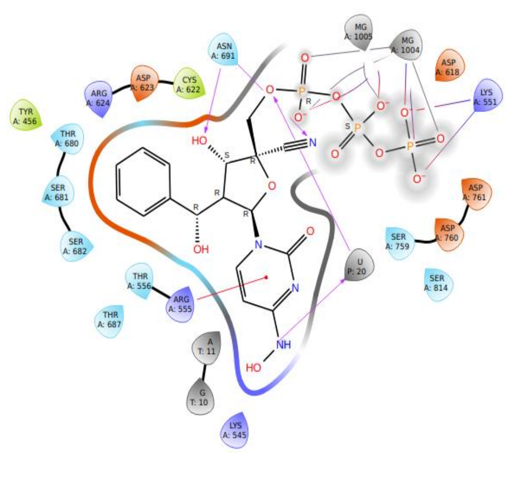
E:11	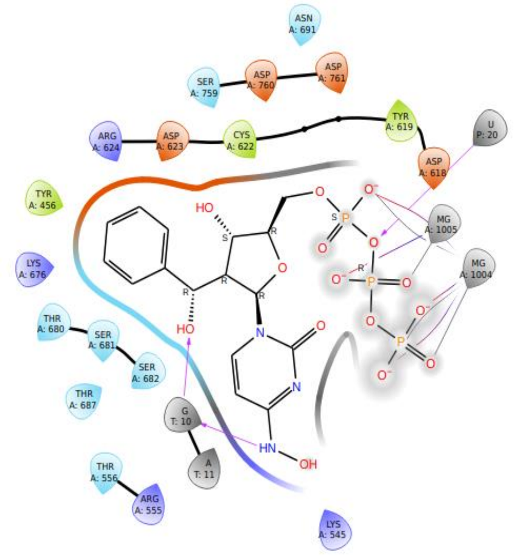	E:12	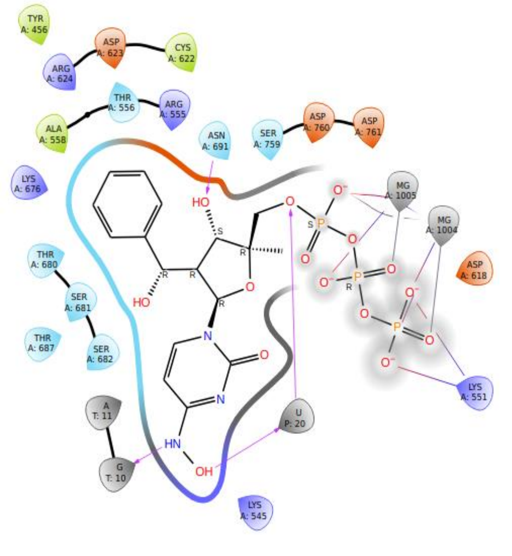
E:15	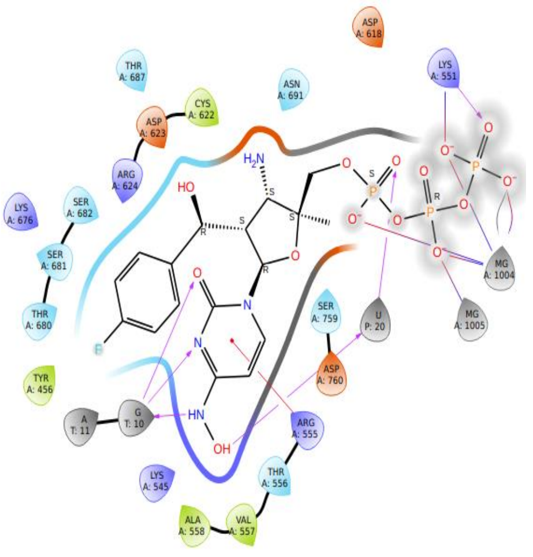	E:16	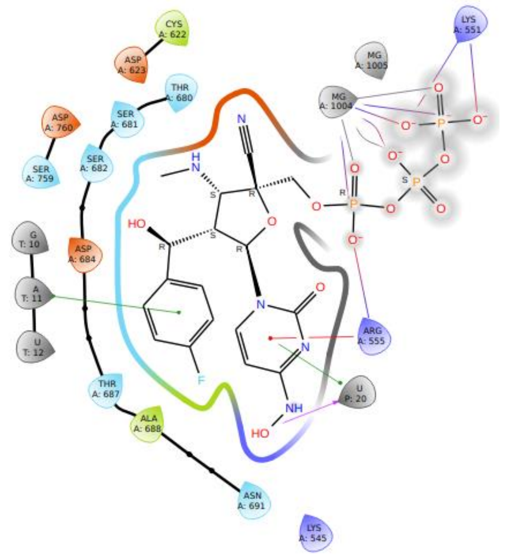

<table>
<tr><td>E:14</td><td>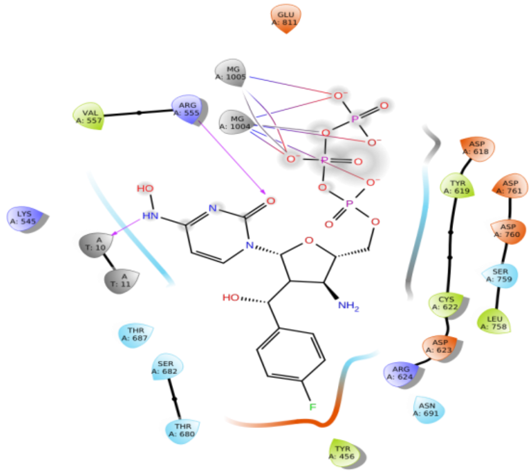</td><td>E:15</td><td>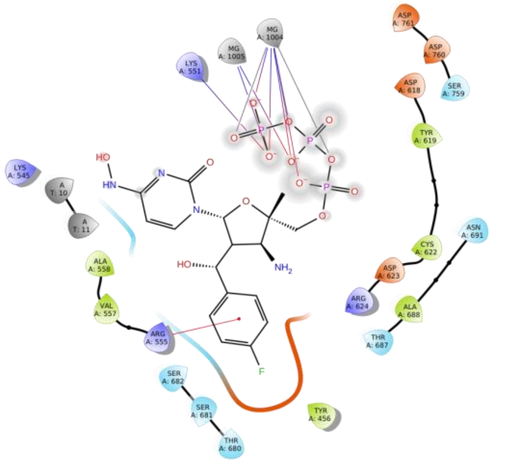</td></tr>
<tr><td>E:16</td><td>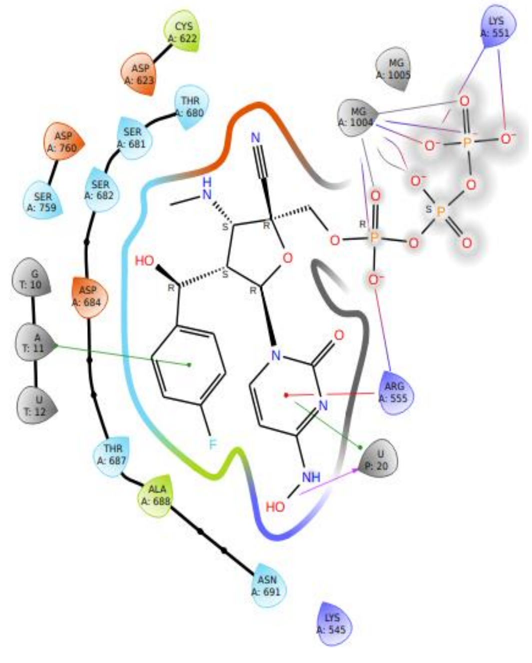</td><td>E:17</td><td>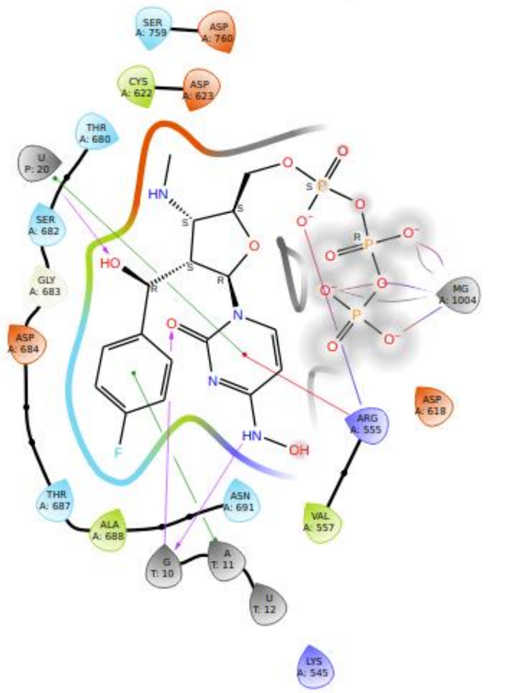</td></tr>
</table>

E:18	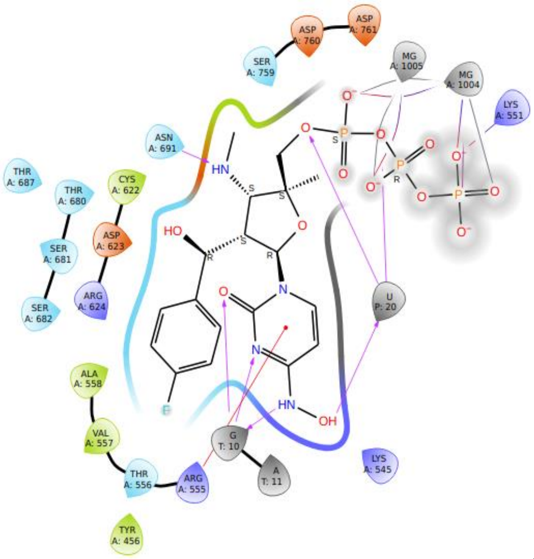	E:20	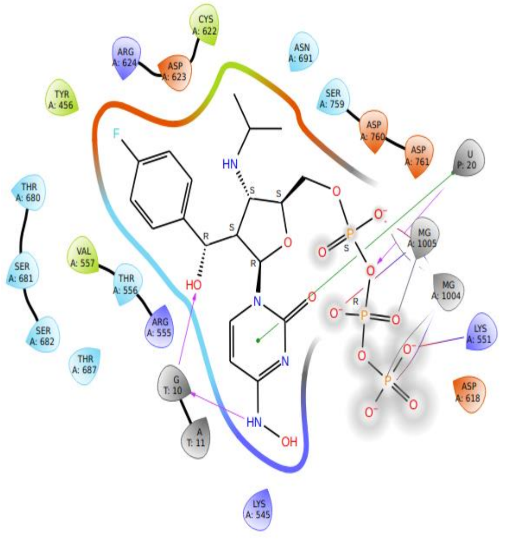
E:22	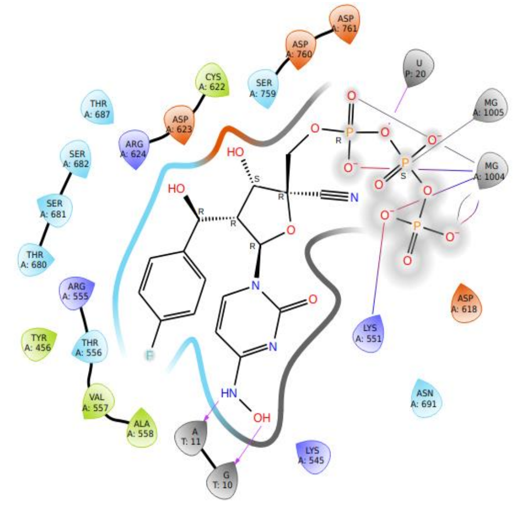	E:24	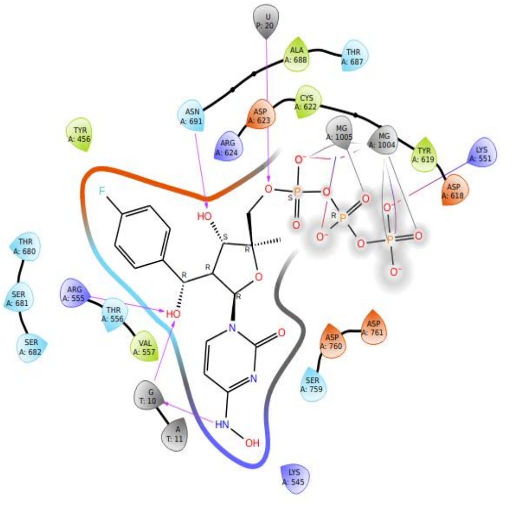
E:26	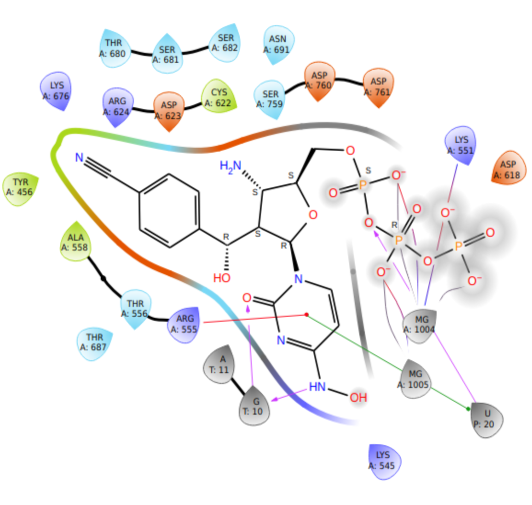	E:27	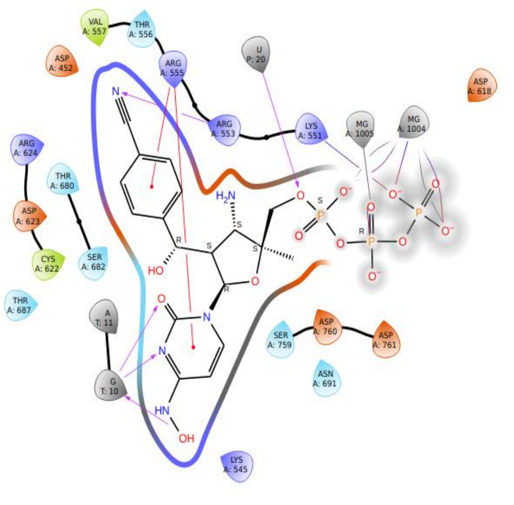

E:30	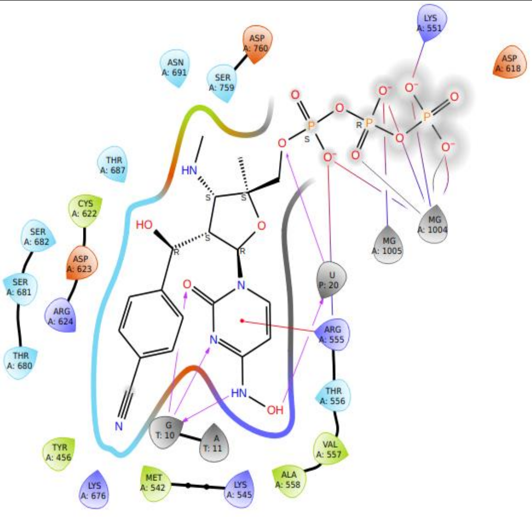	E:32	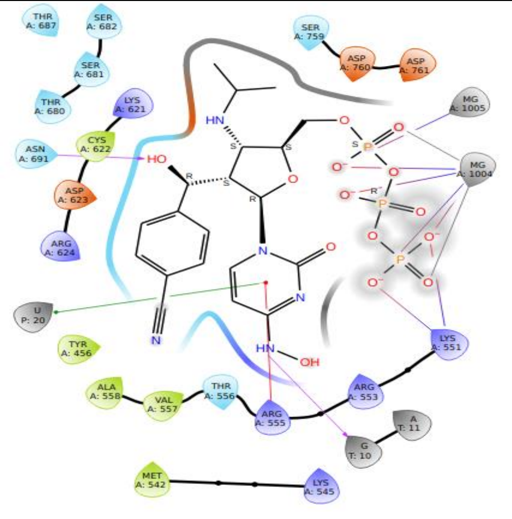
E:33	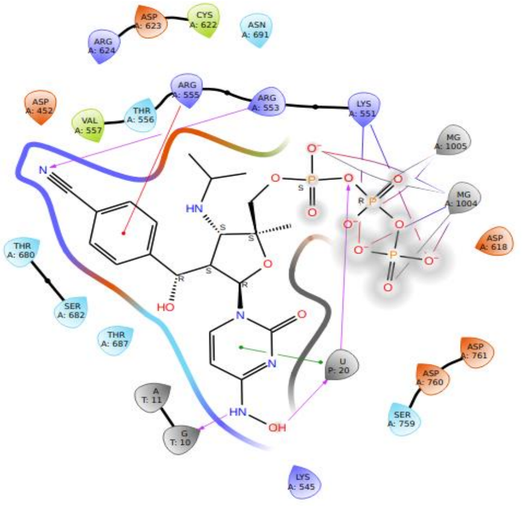	E:34	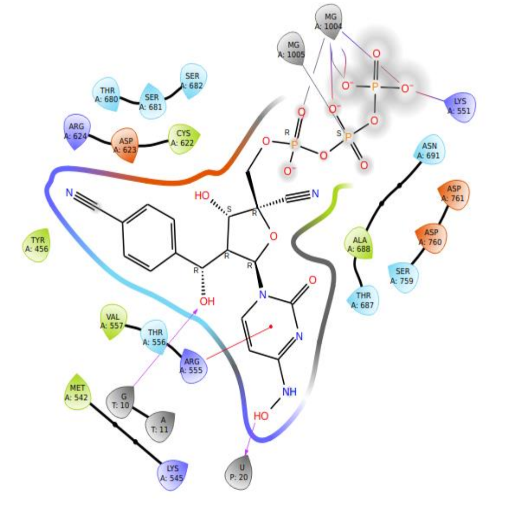
E:35	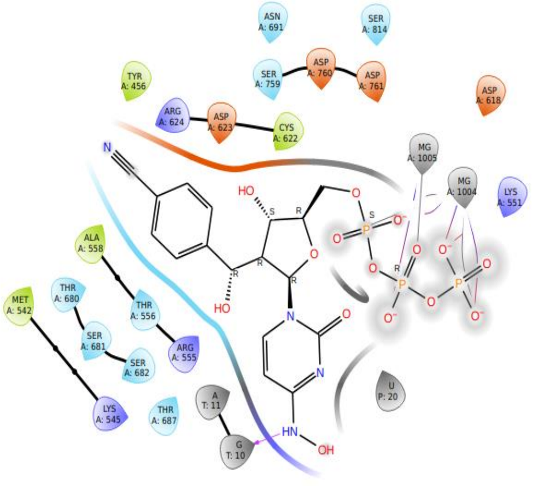	E:36	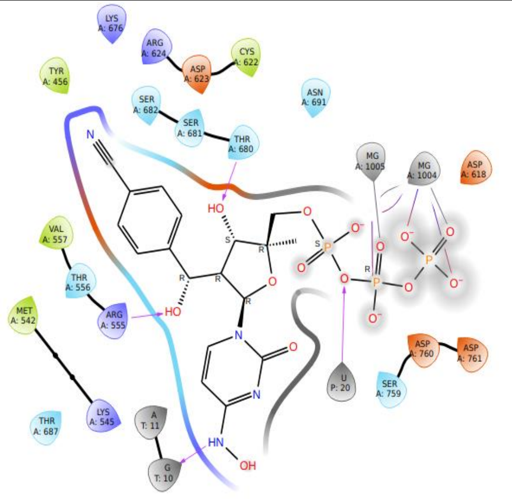

E:37

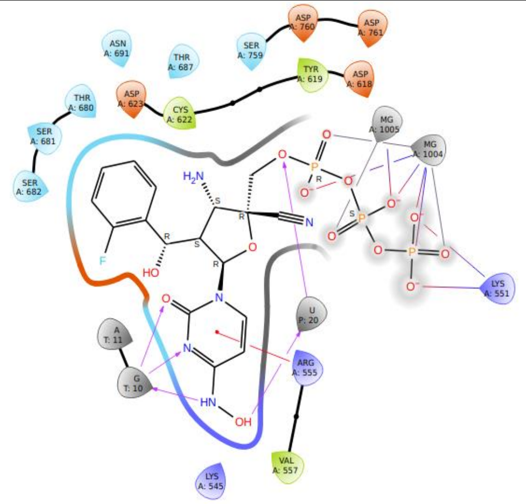

E:38

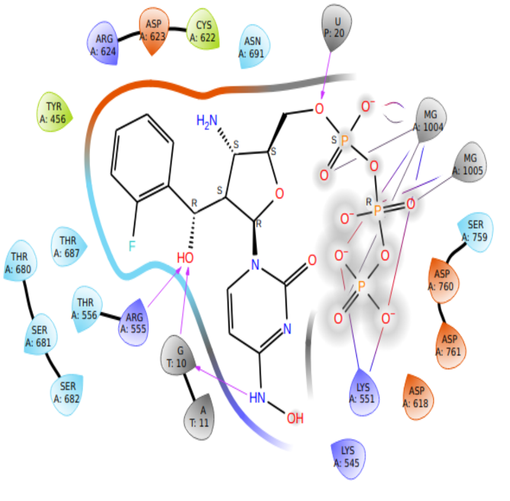

E:39

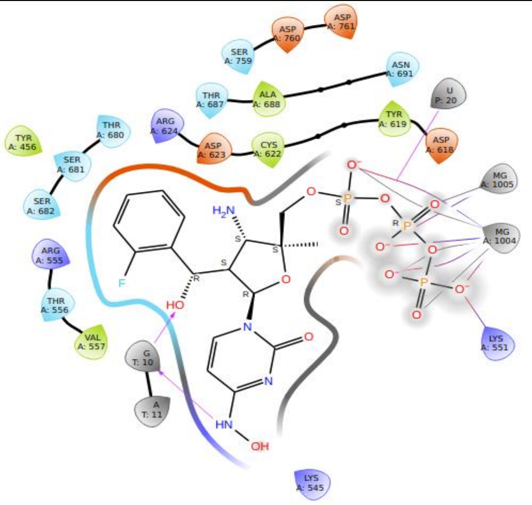

E:40

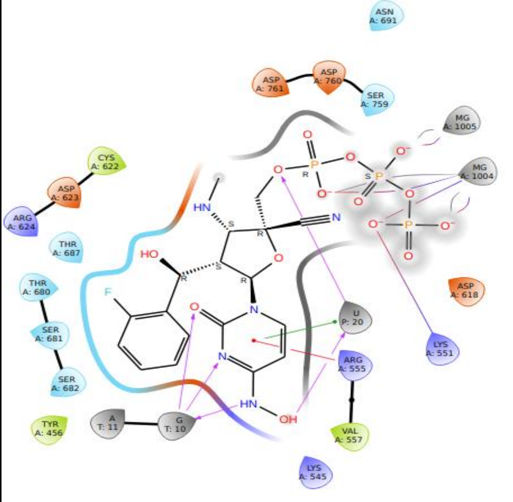

E:41

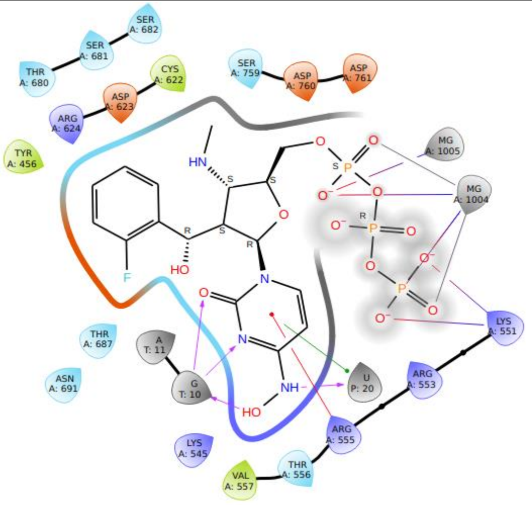

E:42

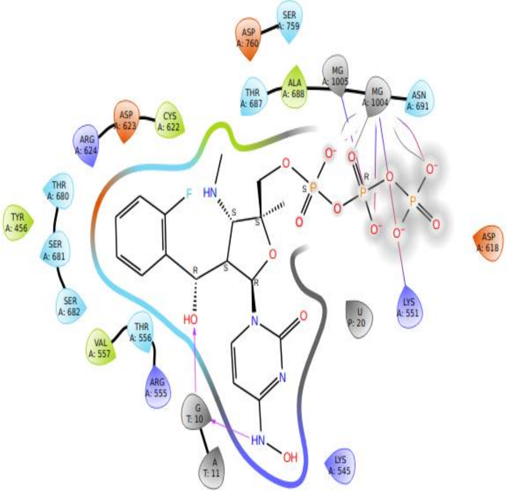

E:43	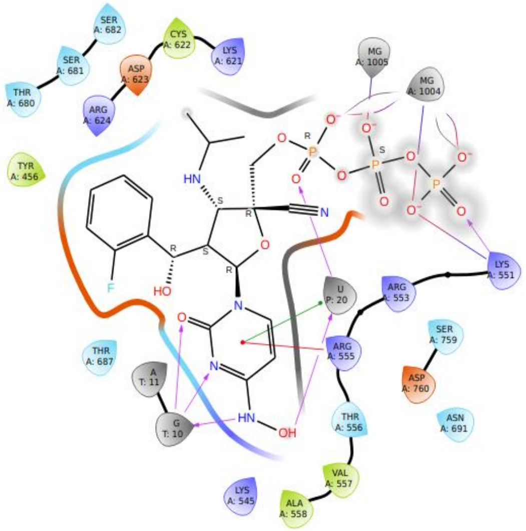	E:46	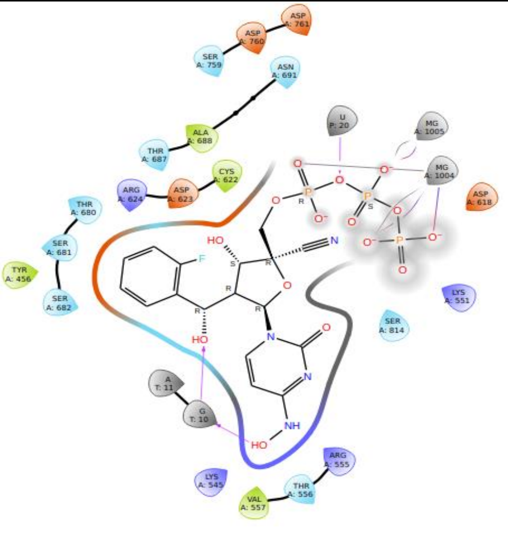
E:47	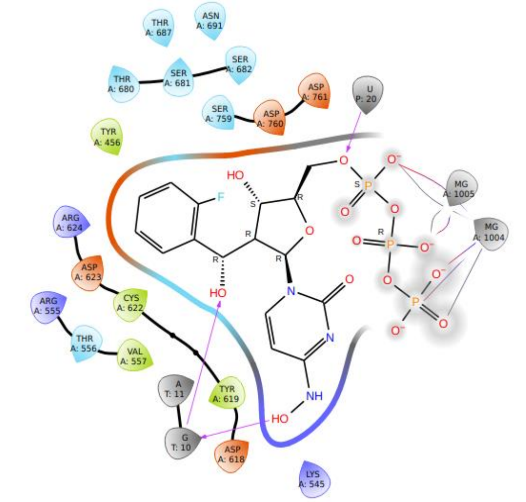	E:48	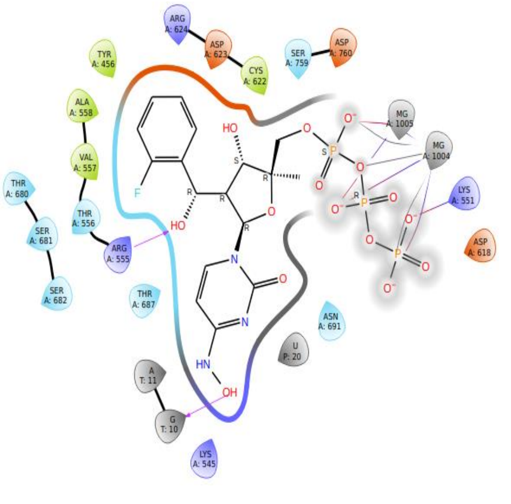
E:49	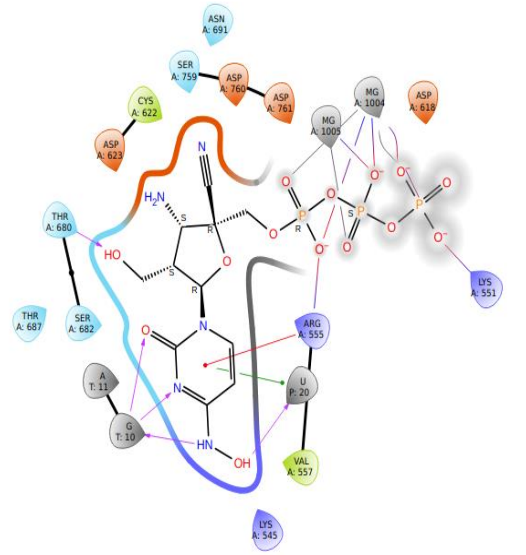	E:50	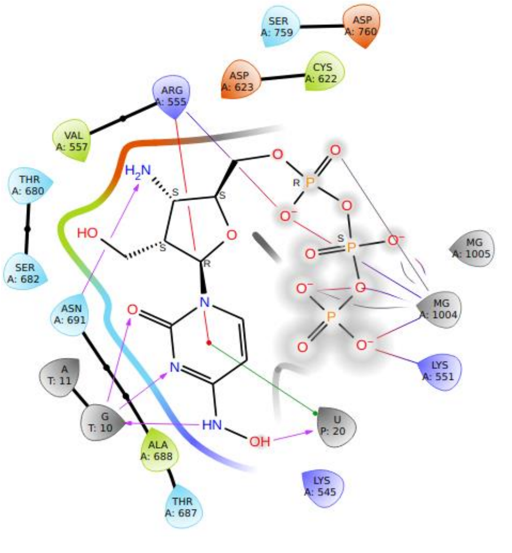

E:51	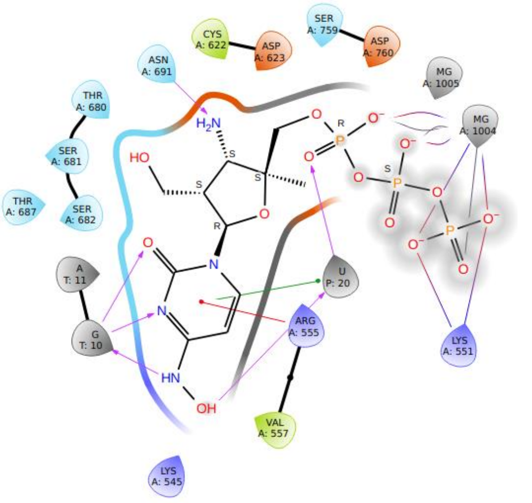	E:52	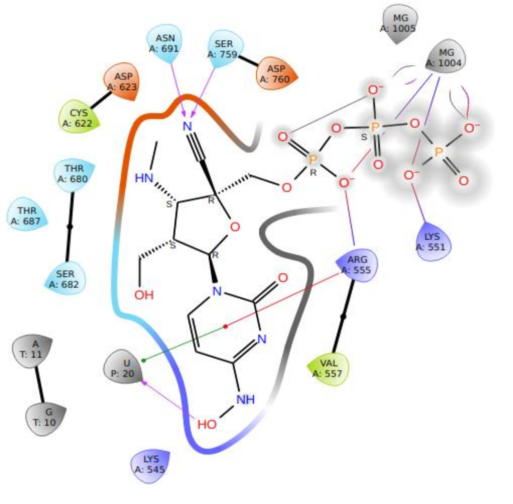
E:53	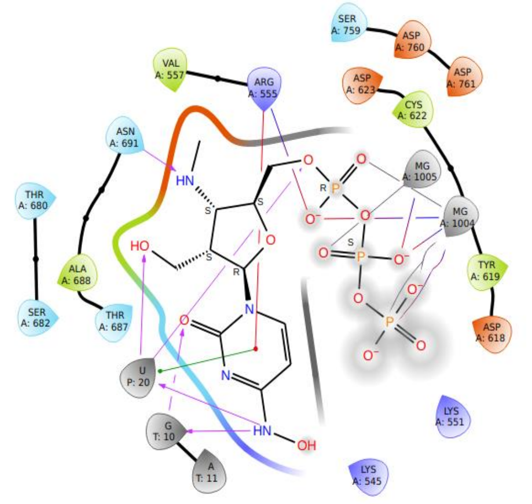	E:54	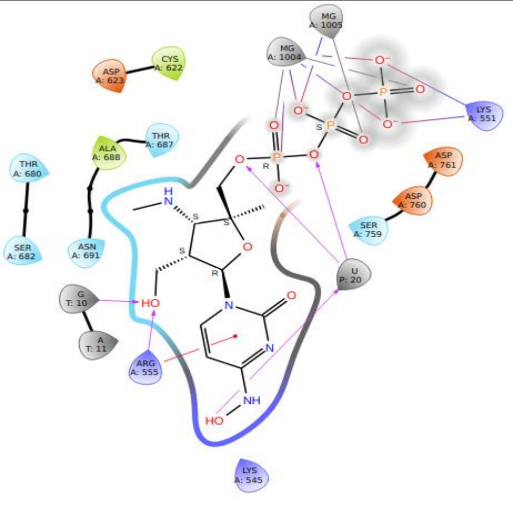
E:55	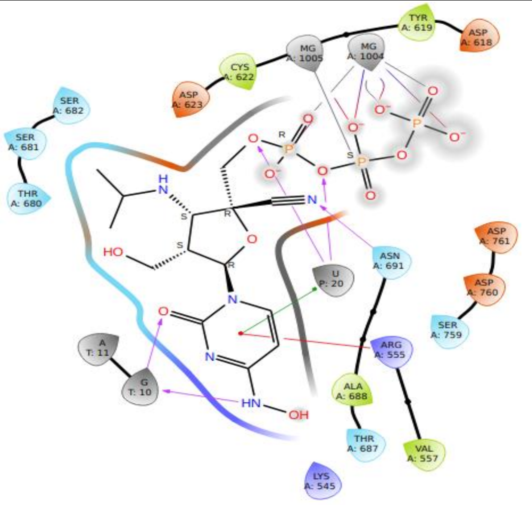	E:56	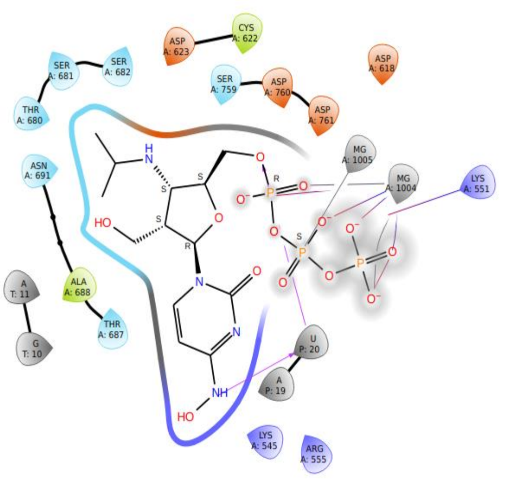

E:57	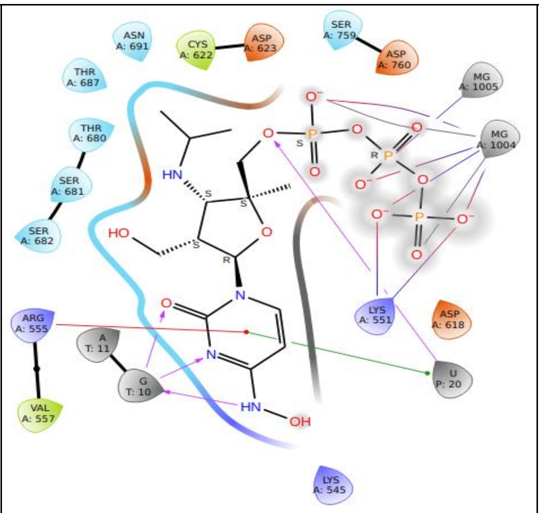	E:58	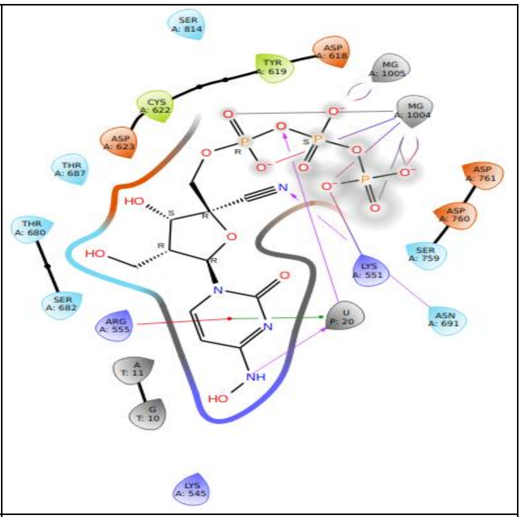
E:59	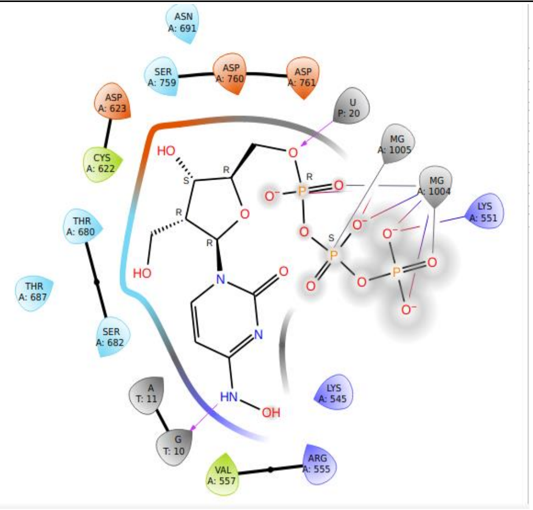	E:60	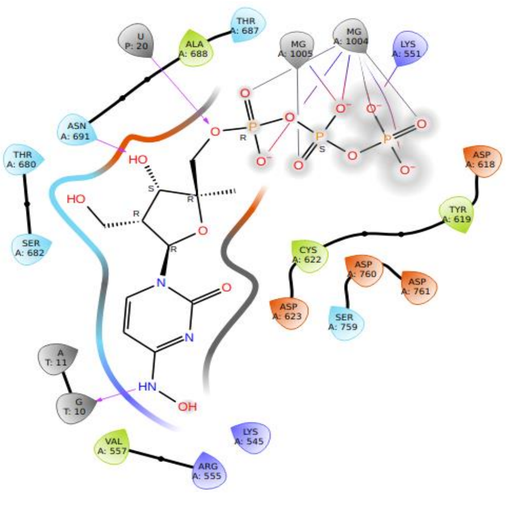
E:61	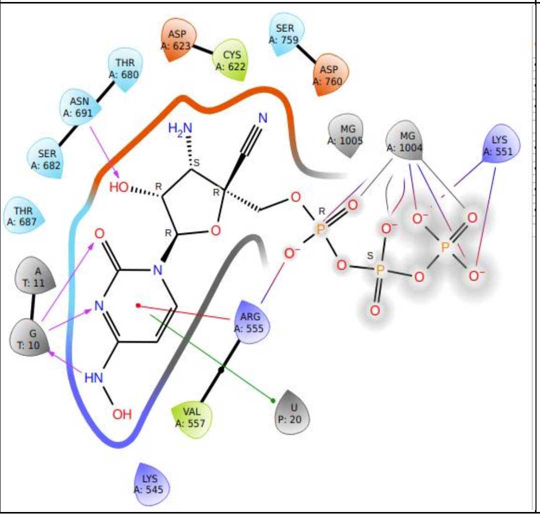	E:62	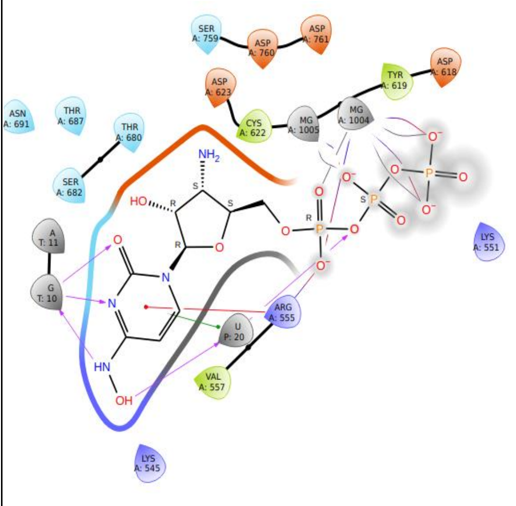

E:63	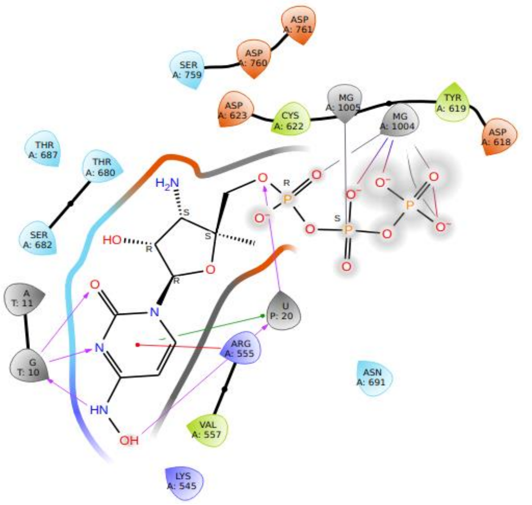	E:64	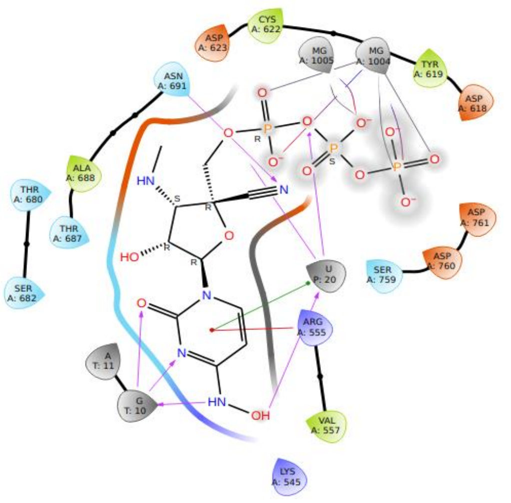
E:65	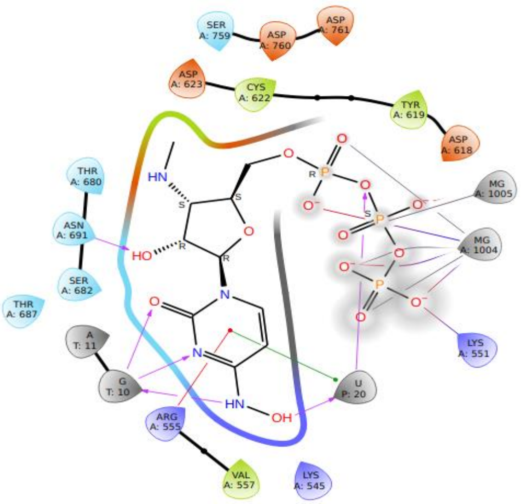	E:66	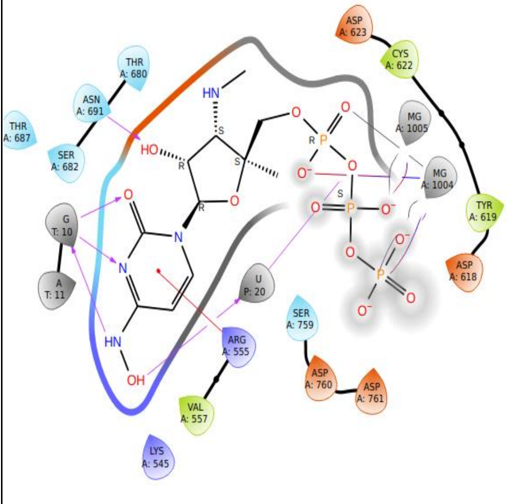
E:67	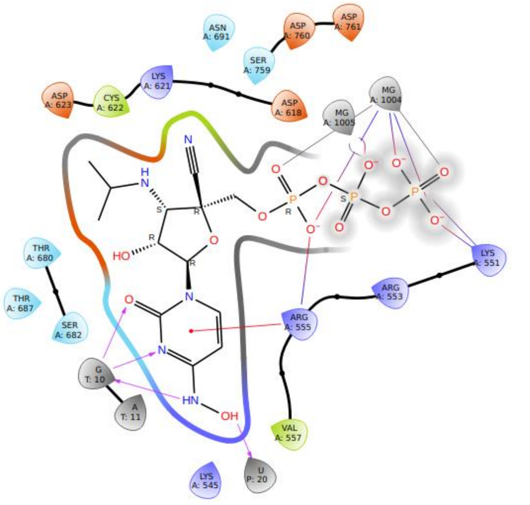	E:68	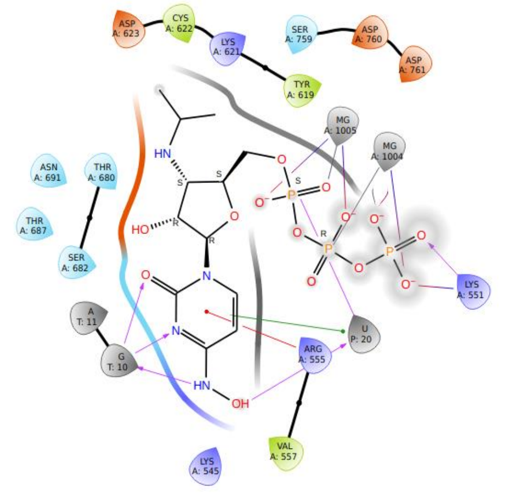

E:69	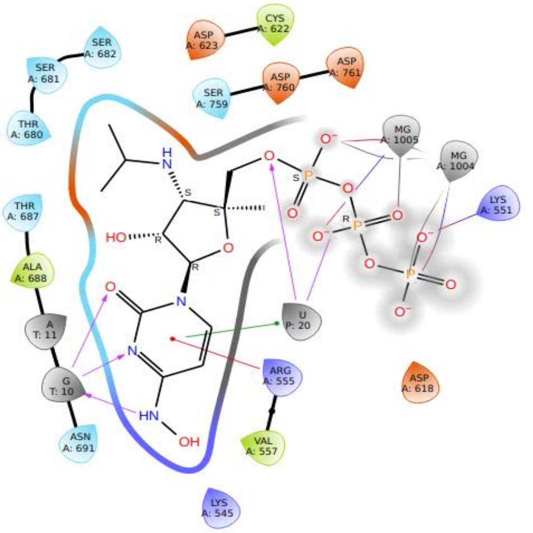	E:70	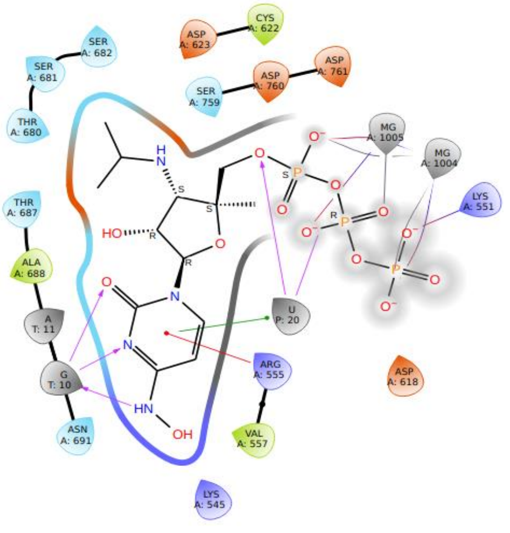
E:72	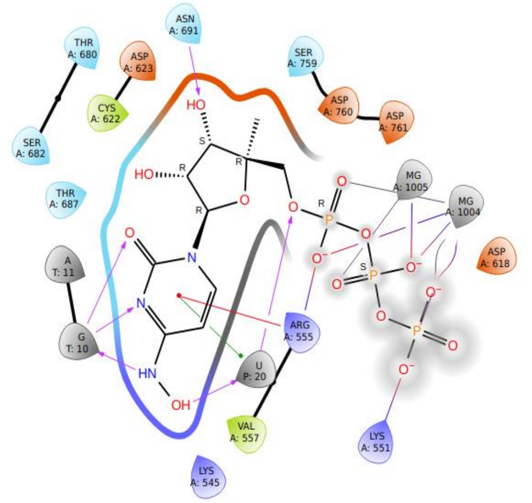		

Figure S7. Protein-ligand docking interactions of all NHC-TP analogs systems after Glide SP docking to the SARS-COV-2 RdRp active site.

<table>
<tr><td colspan="3">NHC-TP E:71</td></tr>
<tr>
<td>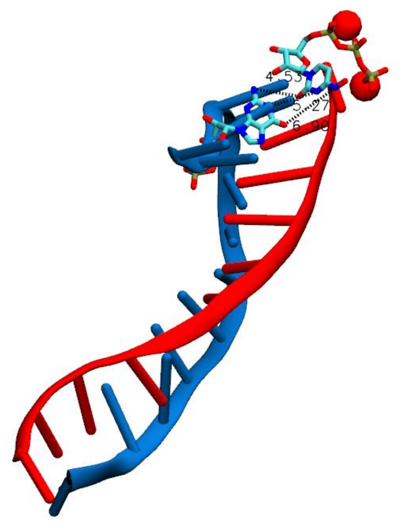
</td>
<td>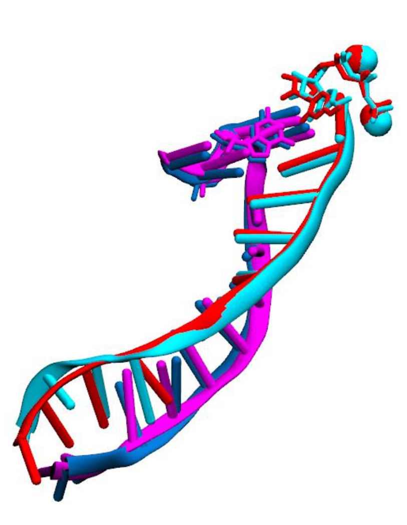</td>
<td>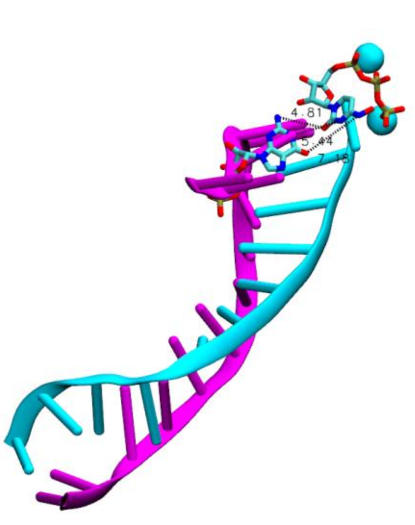
</td>
</tr>
<tr><td colspan="3">E:03</td></tr>
<tr>
<td>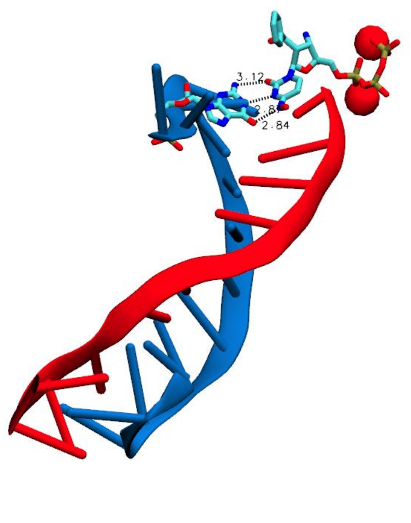
</td>
<td>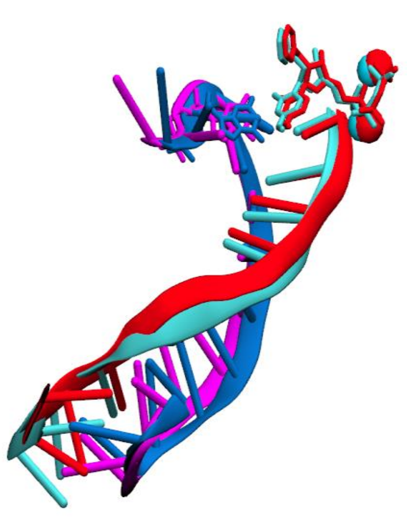</td>
<td>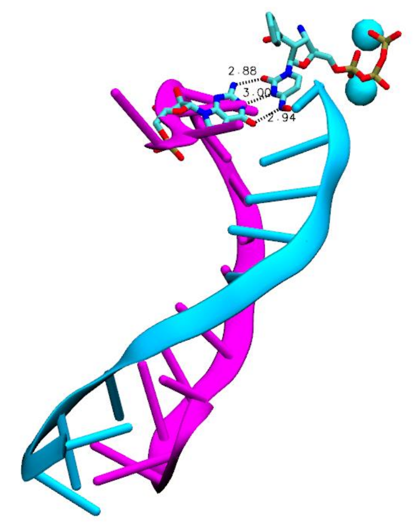
</td>
</tr>
<tr><td colspan="3">E:12</td></tr>
</table>

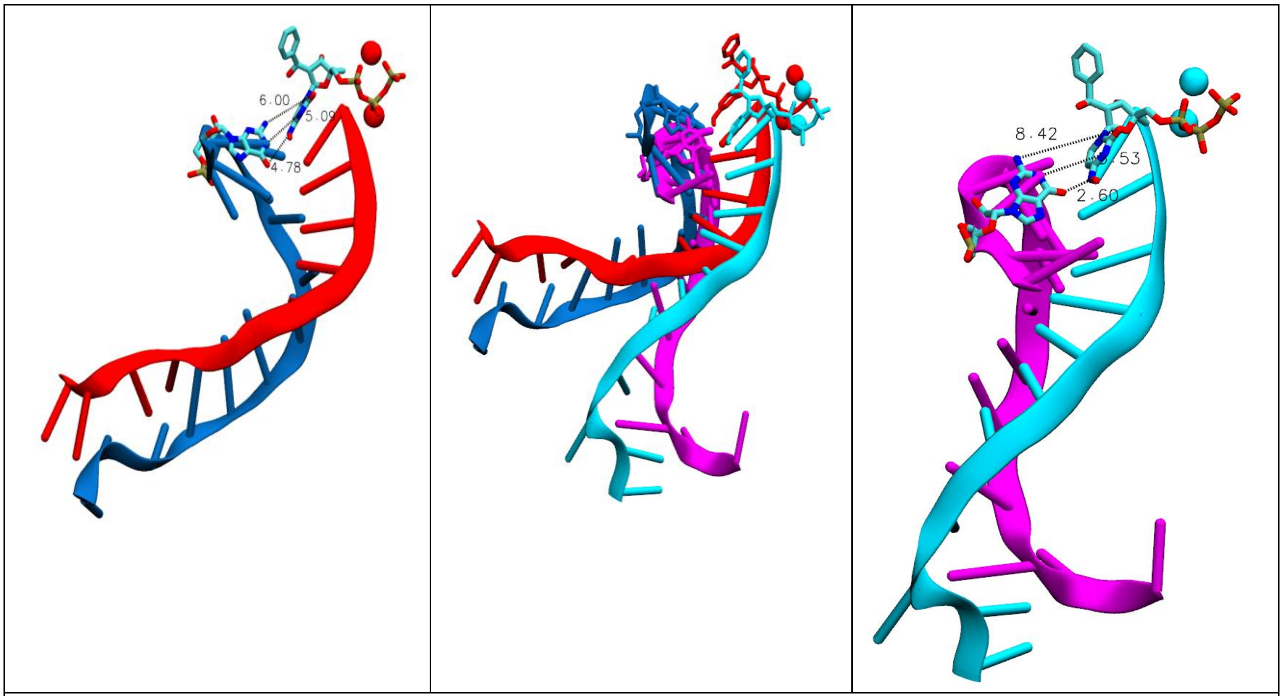

E:48

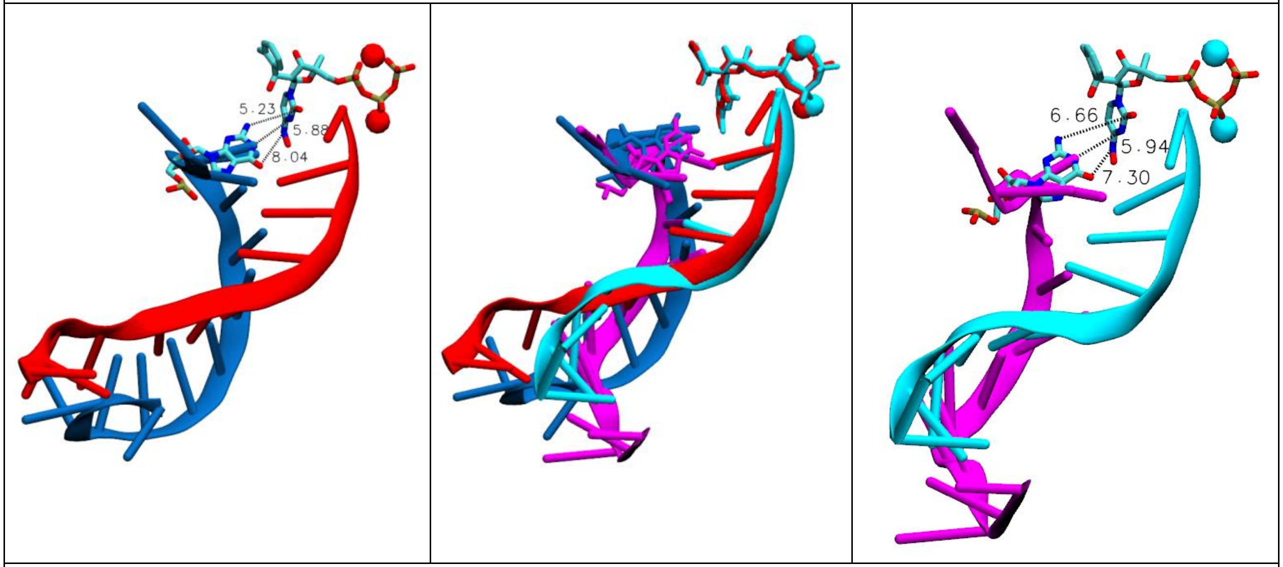

E:53

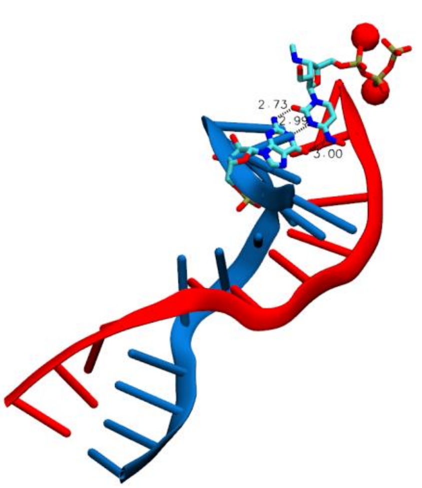	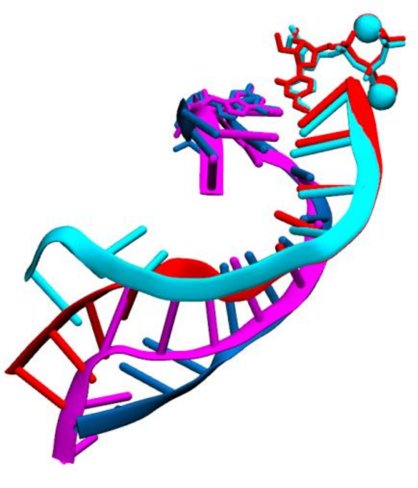	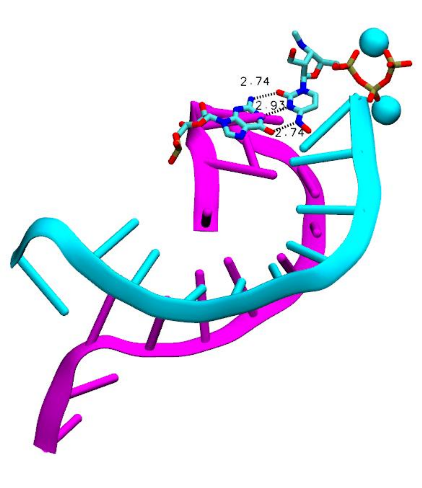
E:63		
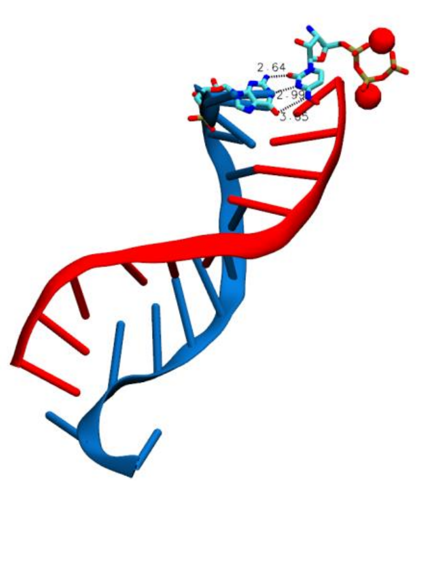	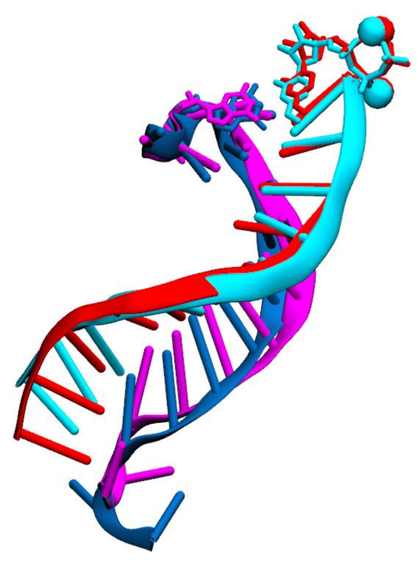	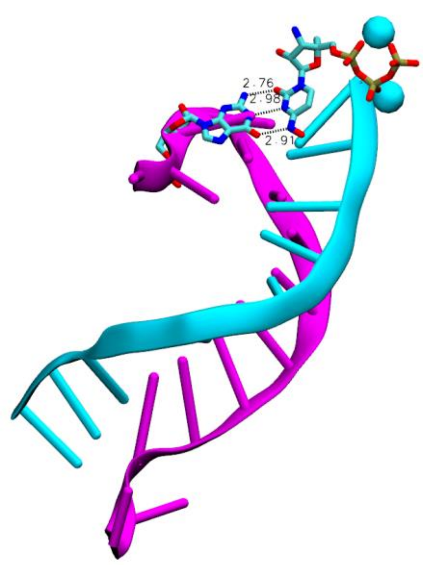
E:65		

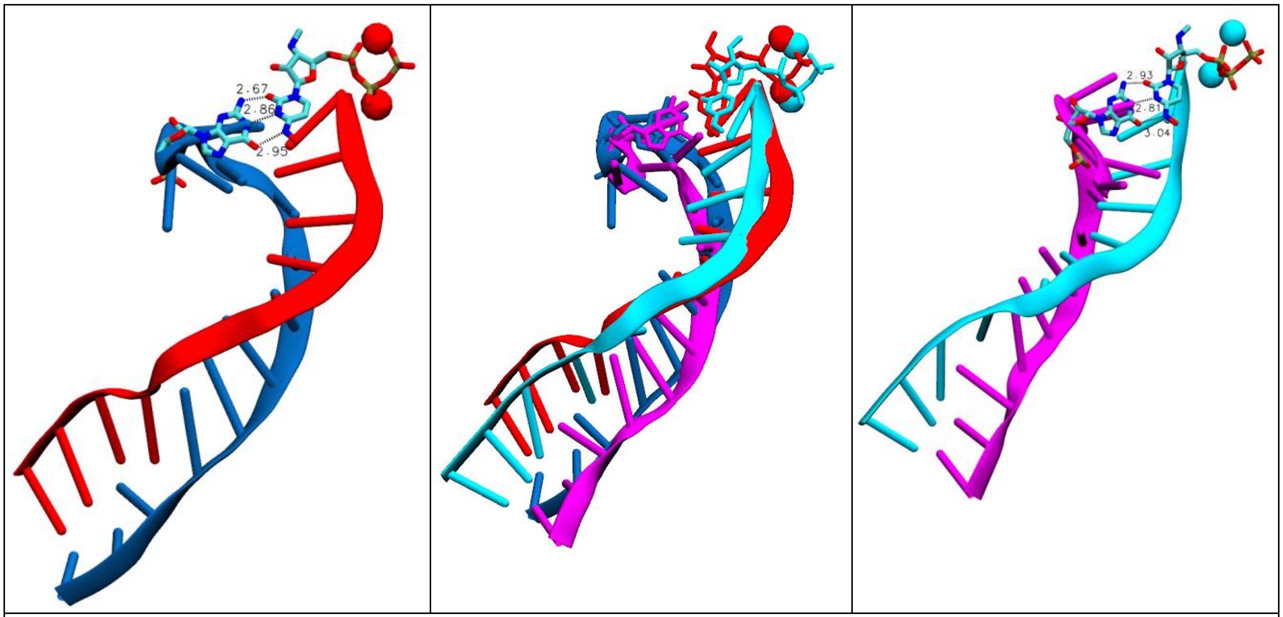

E:66

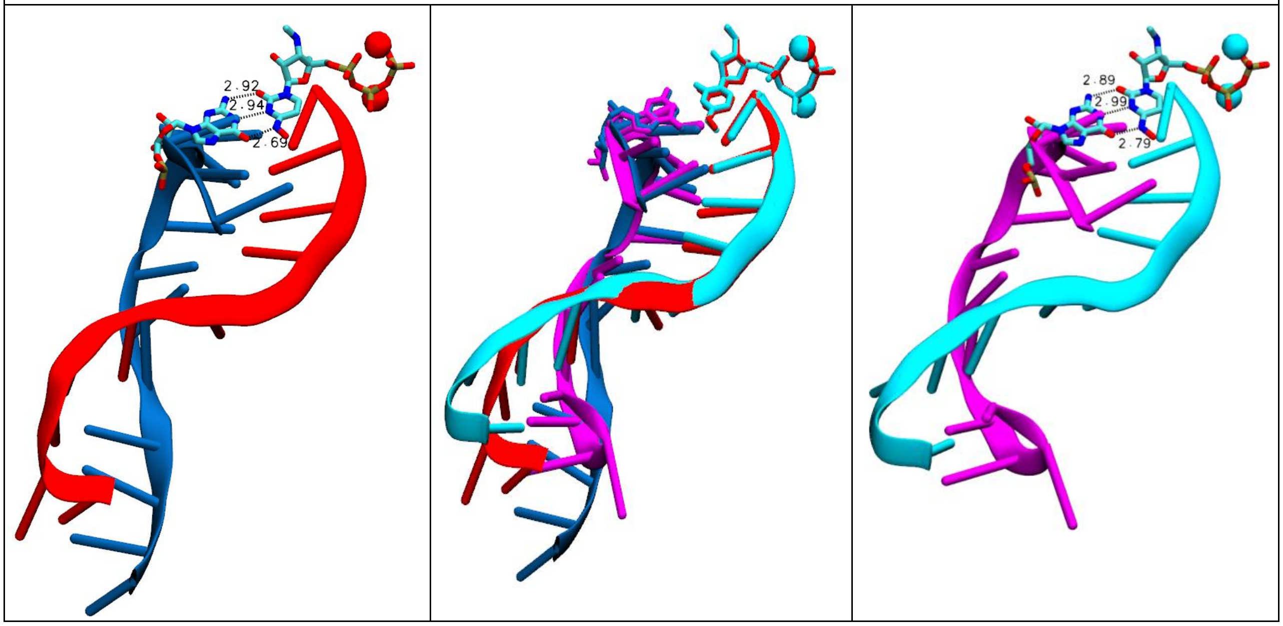

Figure S8. Superposition (middle) of the most abundant conformational ligand pose from MD simulation (left) onto initial molecular docking pose (right) of NHC-TP and the top 7 analogues with surrounding Mg^{2+} ions shown in red and cyan, respectively for the SARS-COV-2 RdRp. (Left) The most abundant conformation through the trajectory, template RNA strand (blue) product strand (red) and G10 of template shown as sticks with H bond distances shown in black to each enumerate ligand shown in sticks with Mg^{2+} ions shown as balls. (Right) Docked position with template strand RNA (magenta) and product strand (cyan). Template strand G10 shown in sticks with H bond distances to enumerate ligands shown in black. (Middle) Comparison of MD pose and docked pose. Template most abundant cluster blue - docking magenta, Product most abundant cluster red - docking cyan, G10 and enumerate ligands shown as licorice Mg^{2+} ions shown as balls.

E:71 (NHC-TP)

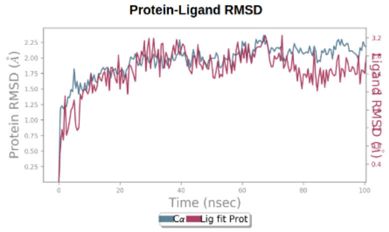

E:01

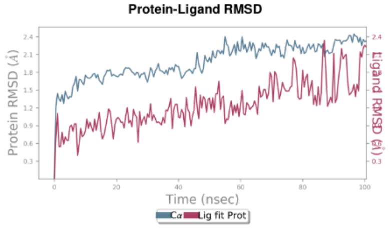

E:03

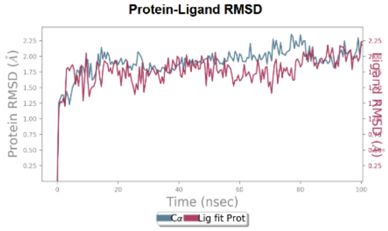

E:04

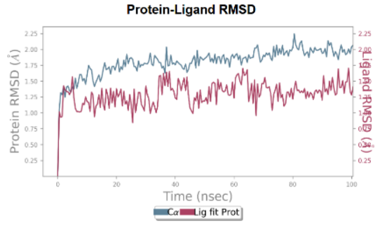

E:06

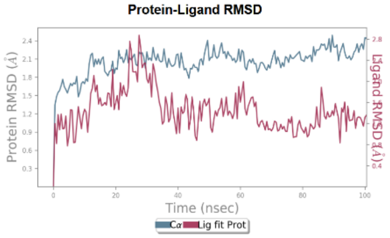

E:07

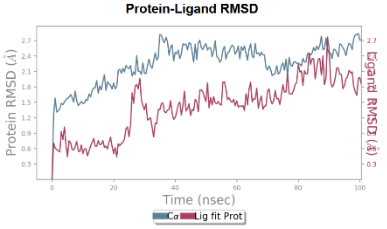

E:08

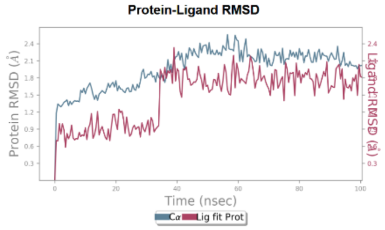

E:10

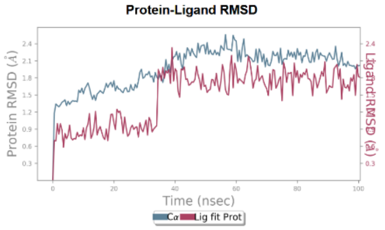

E:11

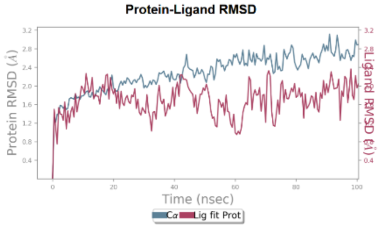

E:12

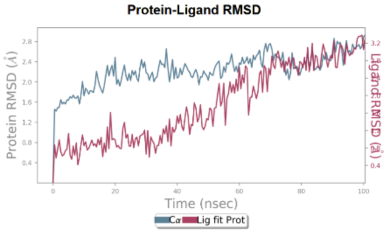

E:15

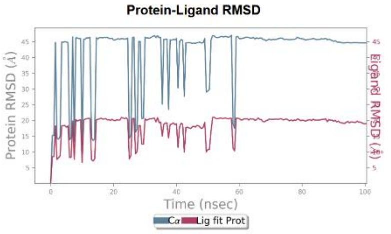

E:16

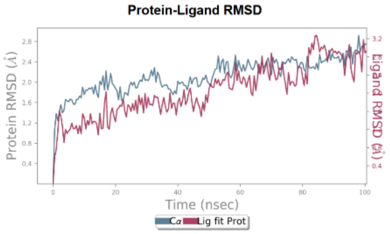

E:17

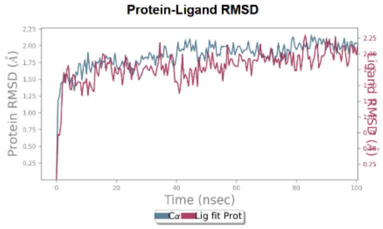

E:18

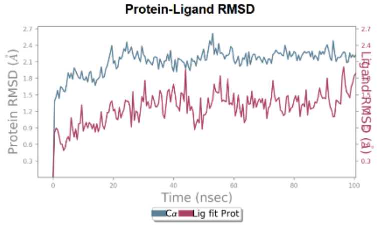

E:20

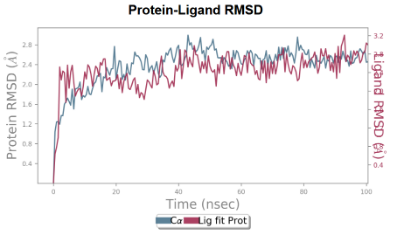

E:22

E:24

E:26

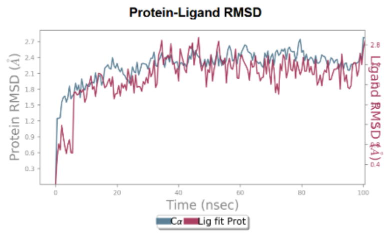

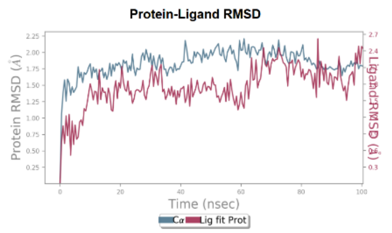

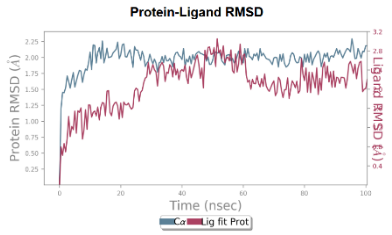

E:27 **E:30** **E:32**

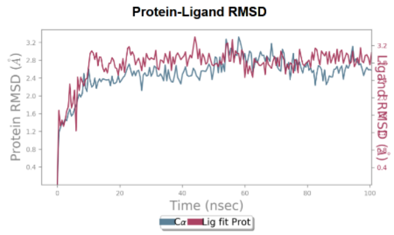

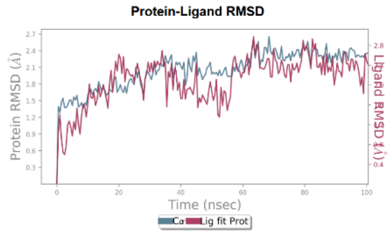

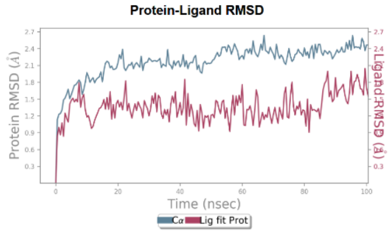

E:33 **E:34** **E:35**

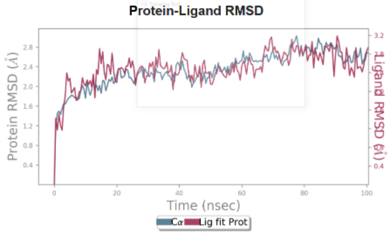

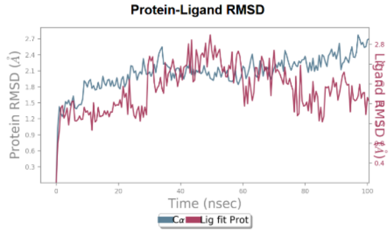

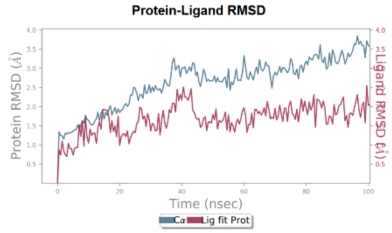

E:36 **E:37** **E:38**

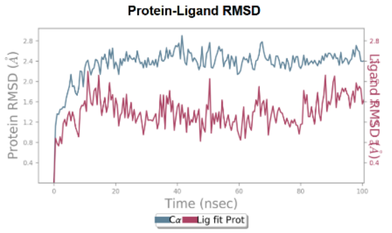

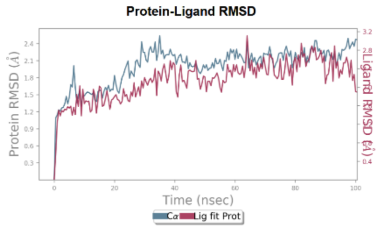

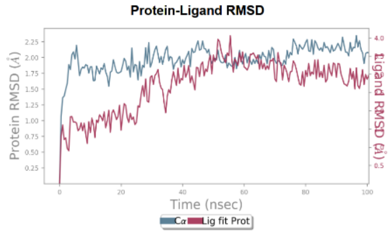

E:39 **E:40** **E:41**

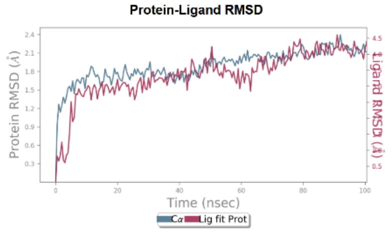

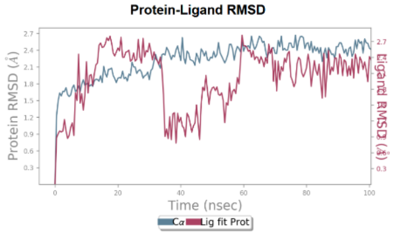

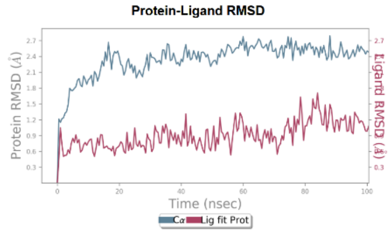

E:42 **E:43** **E:46**

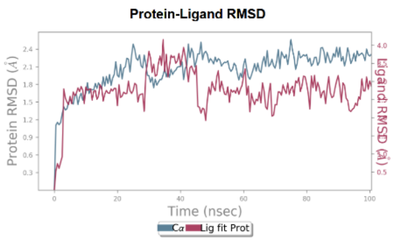

E:47

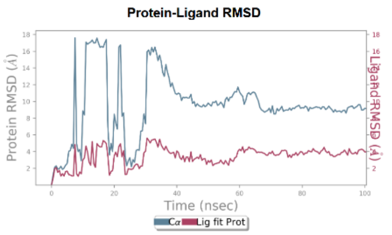

E:48

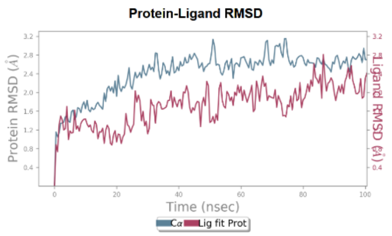

E:49

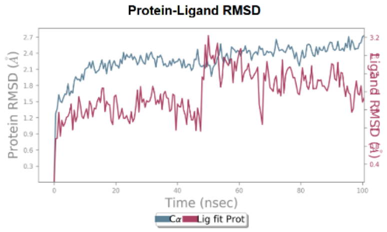

E:50

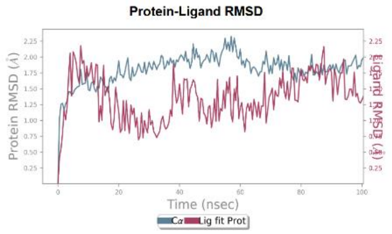

E:51

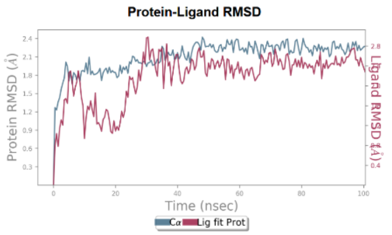

E:52

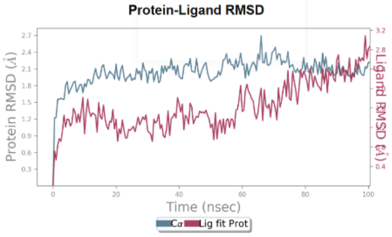

E:53

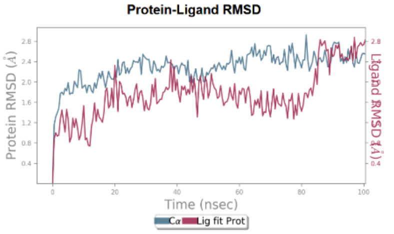

E:54

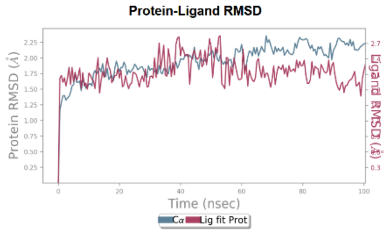

E:55

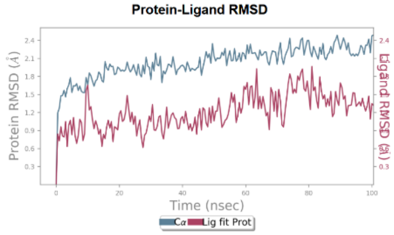

E:56

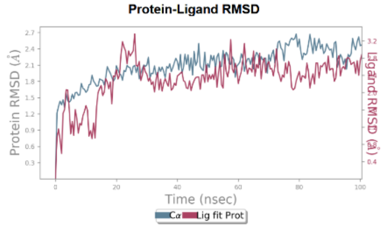

E:57

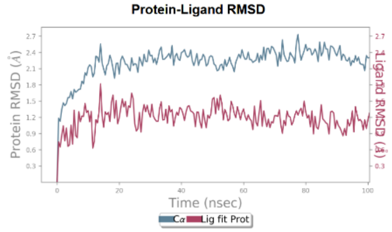

E:58

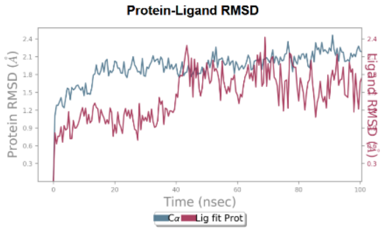

E:59

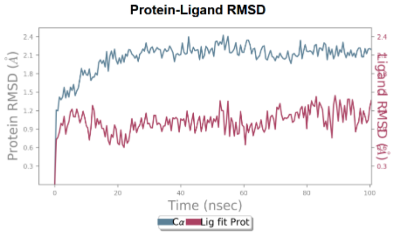

E:60

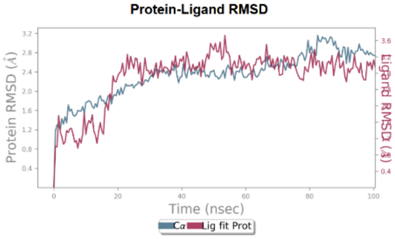

E:61

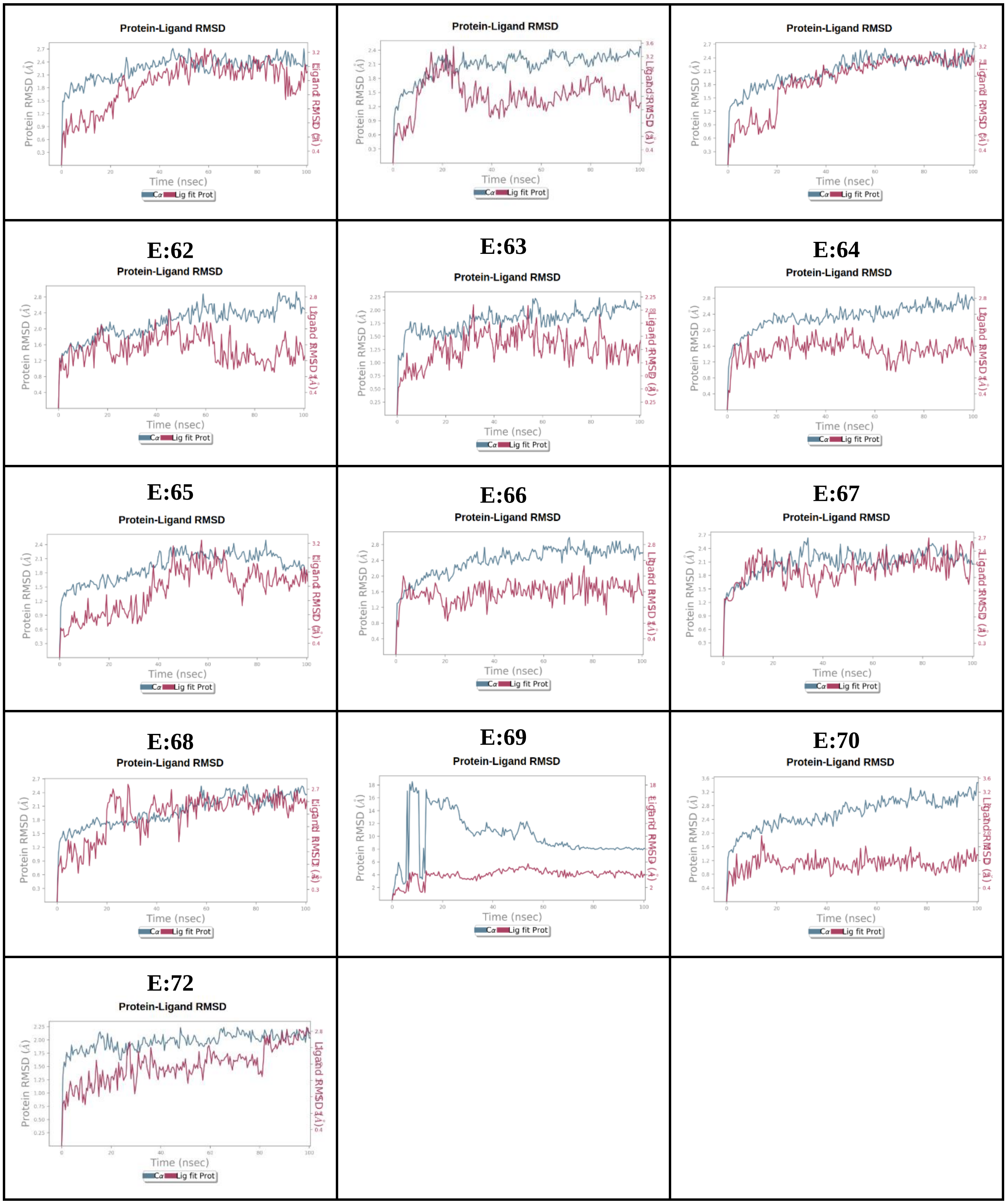

Figure S9. The RMSD values of the protein Cα atoms and ligand heavy atoms fit onto the first snapshot of the protein over 200 ns simulation of all 58 NHC-TP analogue containing RdRp systems.

E:71 (NHC-TP)

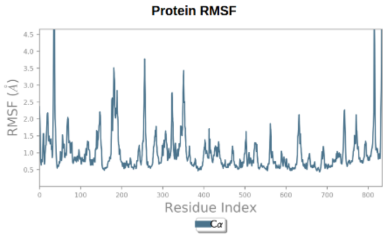

E:01

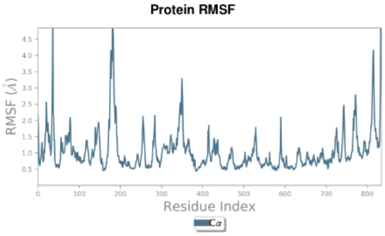

E:03

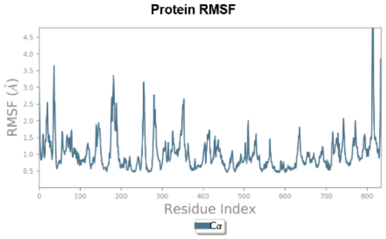

E:04

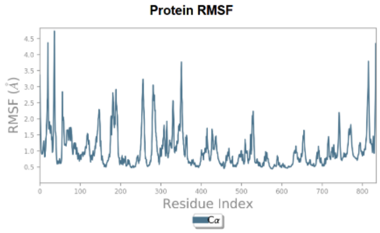

E:06

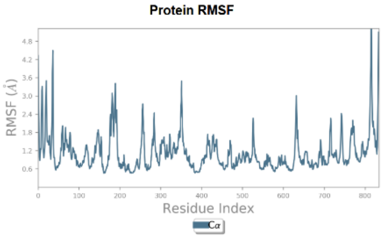

E:07

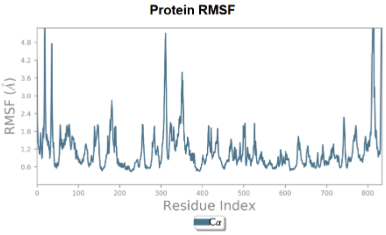

E:08

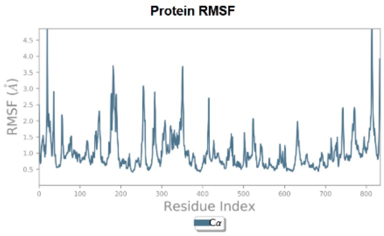

E:10

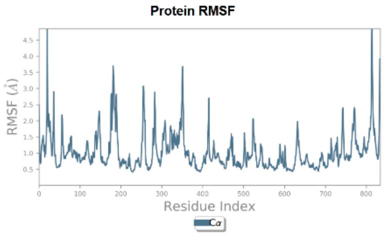

E:11

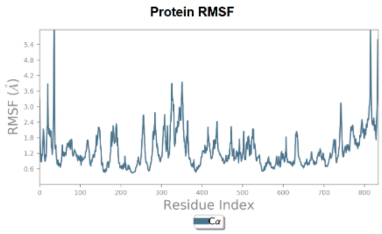

E:12

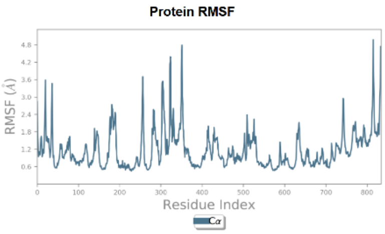

E:15

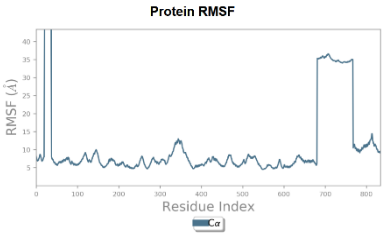

E:16

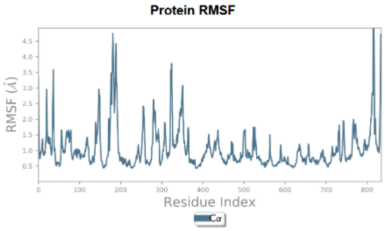

E:17

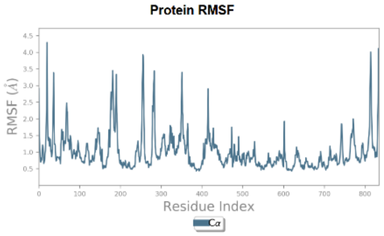

E:18

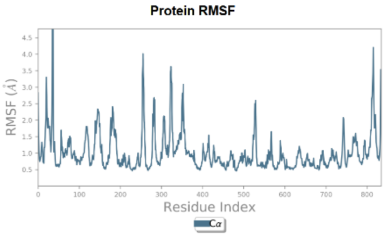

E:20

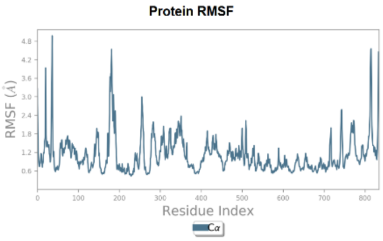

E:22

E:24

E:26

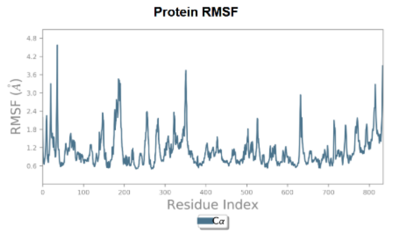

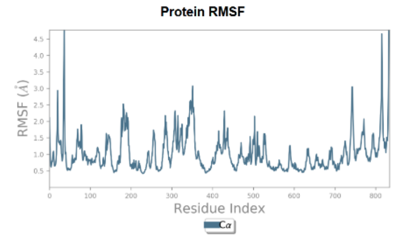

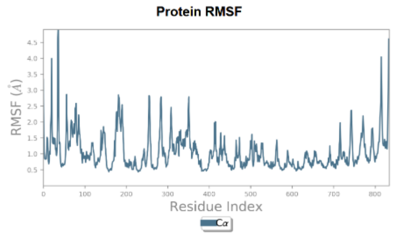

E:27 | E:30 | E:32

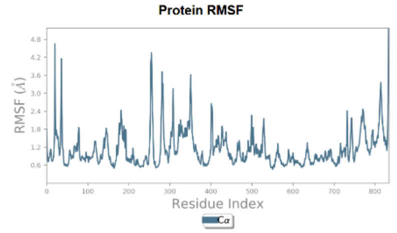

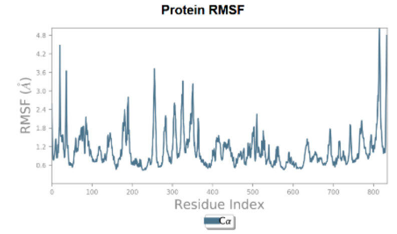

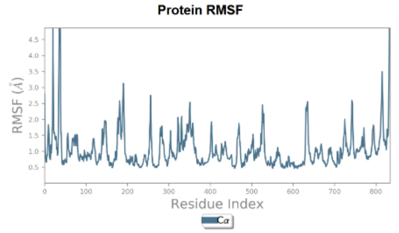

E:33 | E:34 | E:35

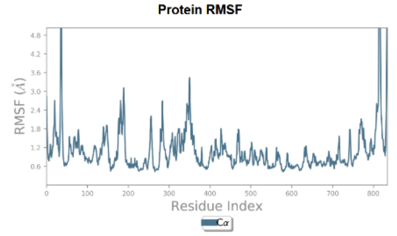

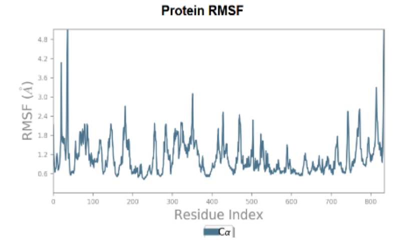

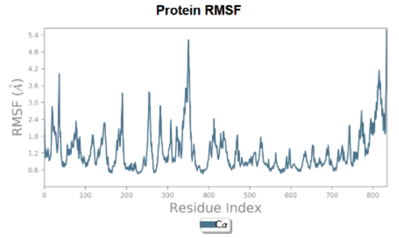

E:36 | E:37 | E:38

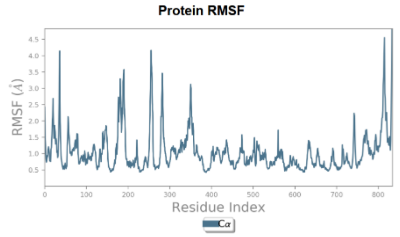

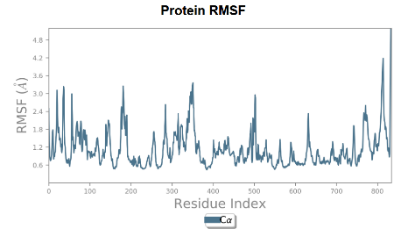

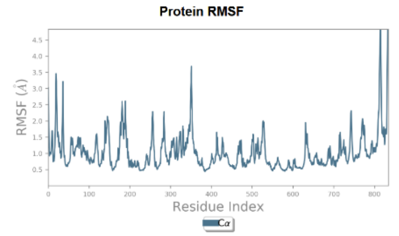

E:39 | E:40 | E:41

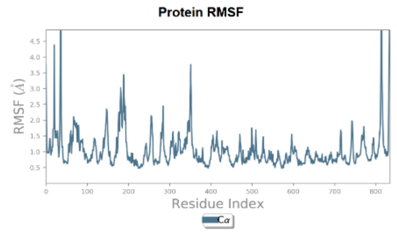

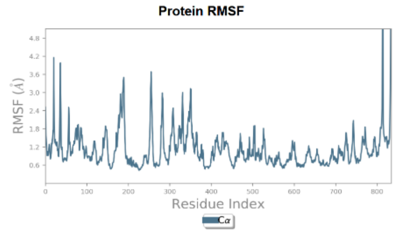

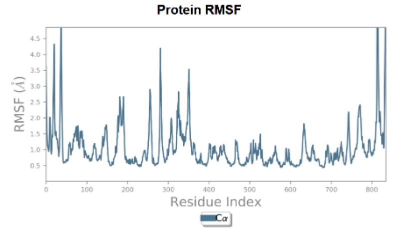

E:42 | E:43 | E:46

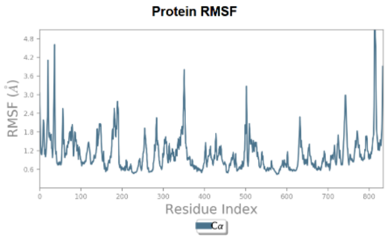

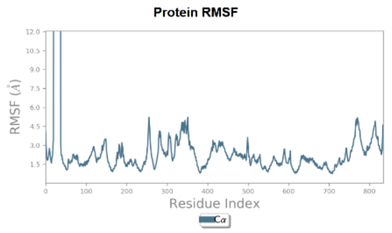

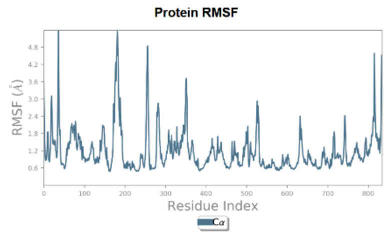

E:47

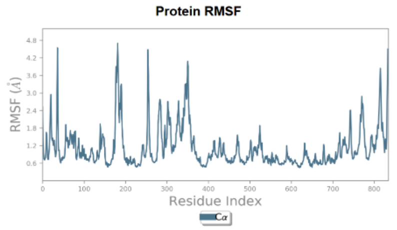

E:48

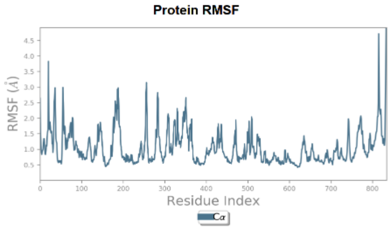

E:49

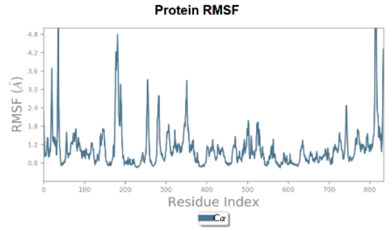

E:50

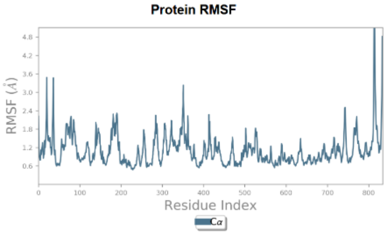

E:51

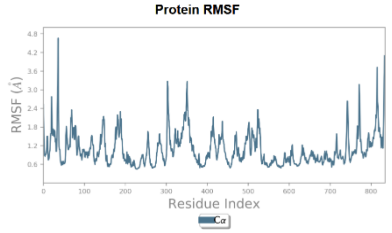

E:52

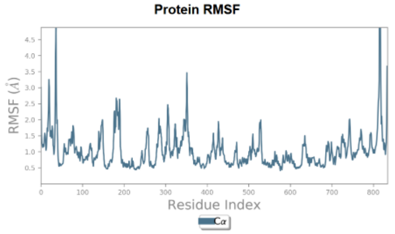

E:53

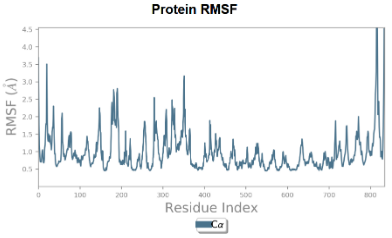

E:54

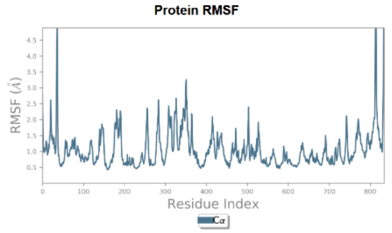

E:55

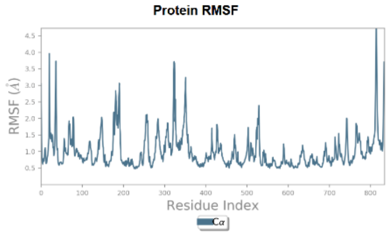

E:56

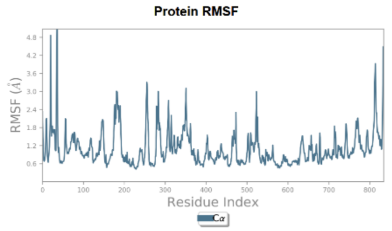

E:57

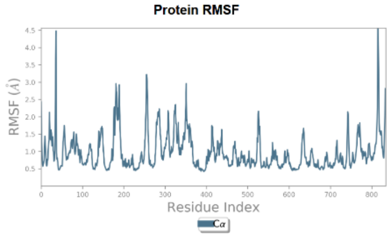

E:58

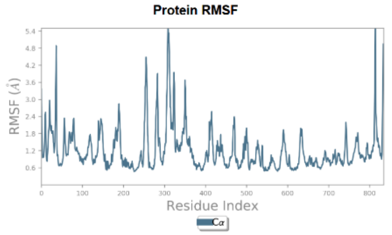

E:59

E:60

E:61

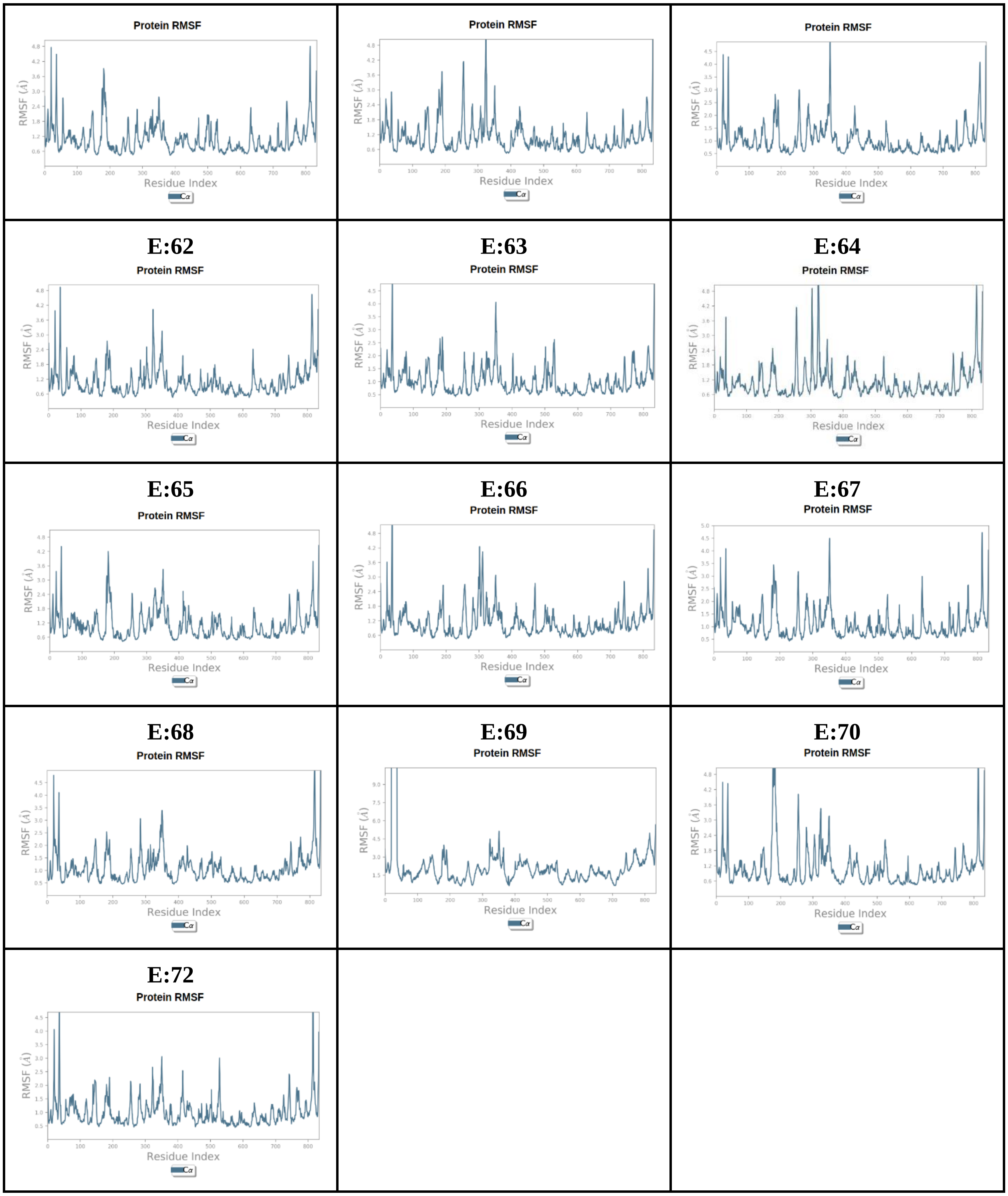

Figure S10. The RMSF values of the protein Cα atoms for all 58 SARS-COV-2 RdRp-NHC-TP MD systems.

Ligand	2D L/P Contacts Summary	Ligand	2D L/P Contacts Summary
E:71 (NHC-TP)	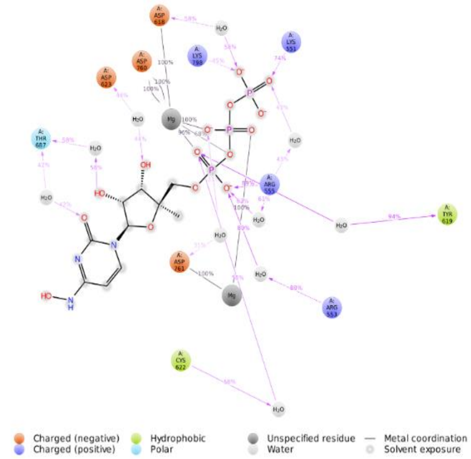	E:01	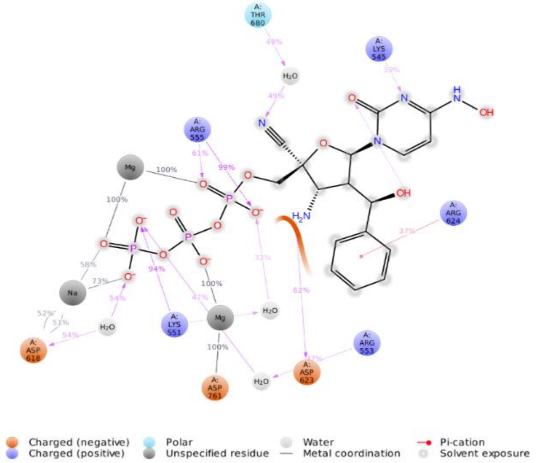
E:03	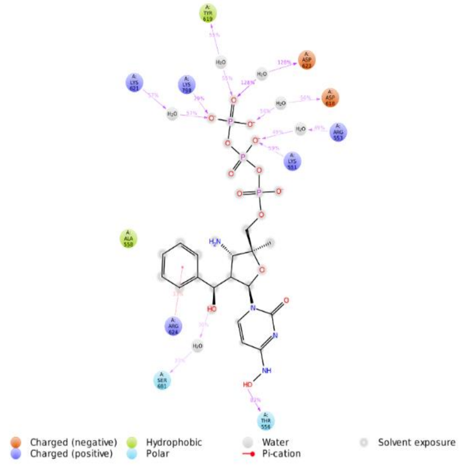	E:04	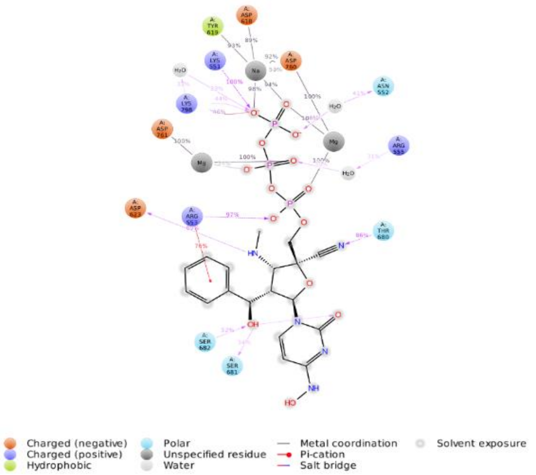
E:06	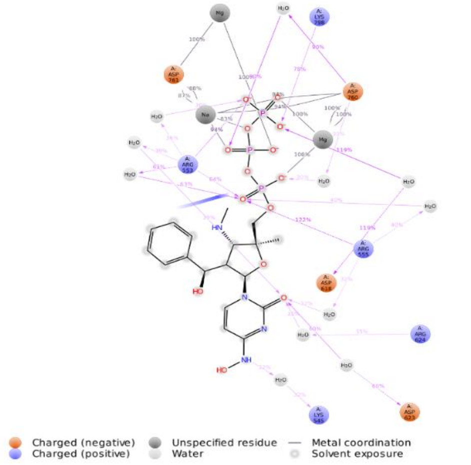	E:07	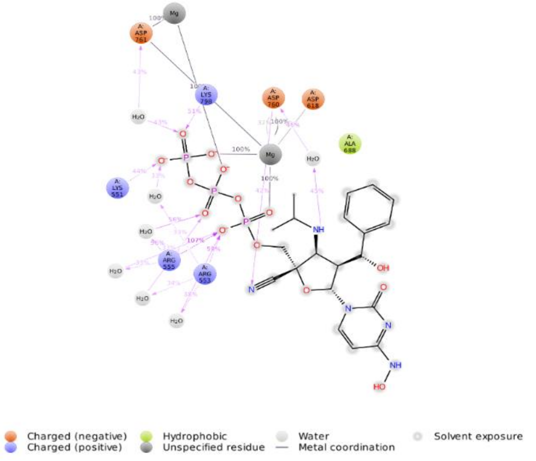

E:08

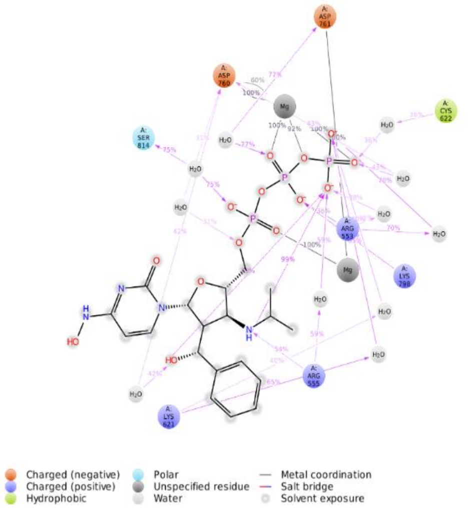

E:10

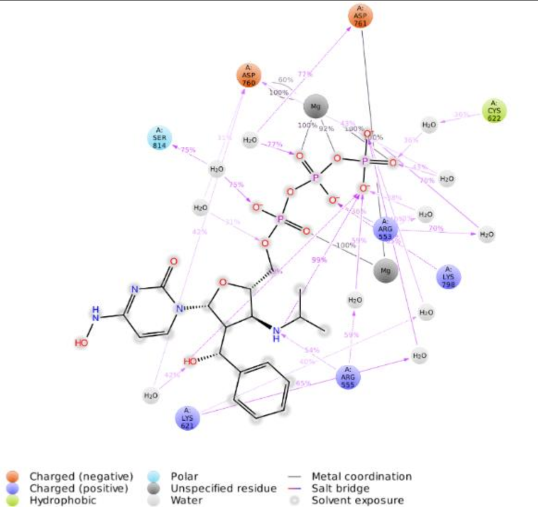

E:11

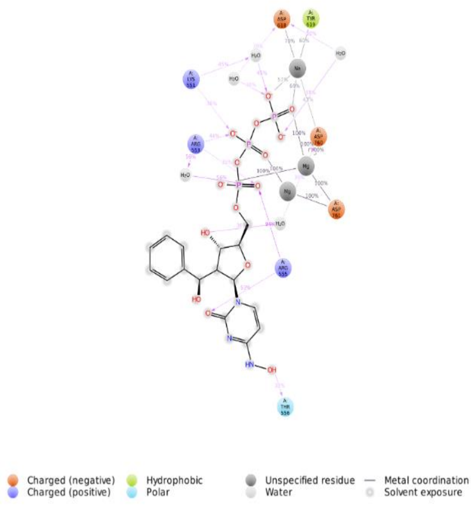

E:12

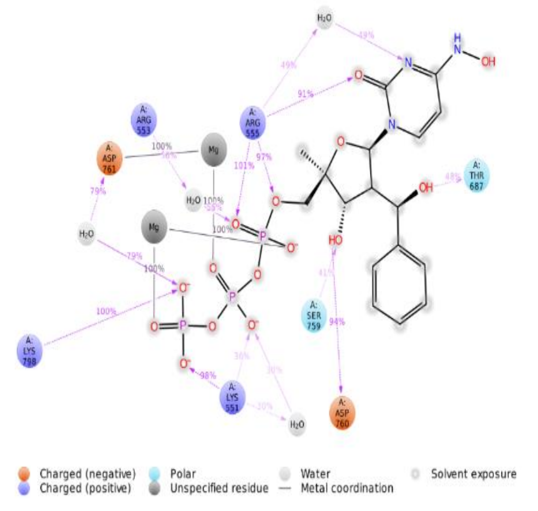

E:15

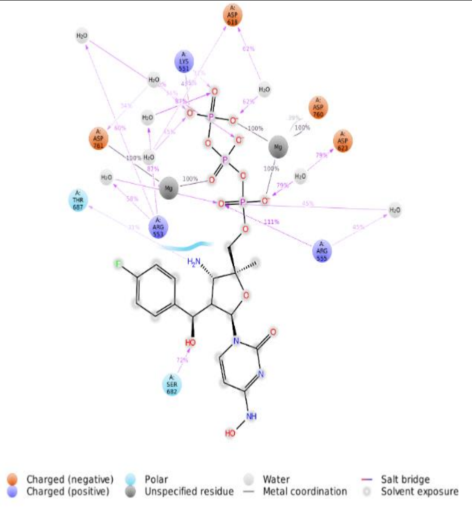

E:16

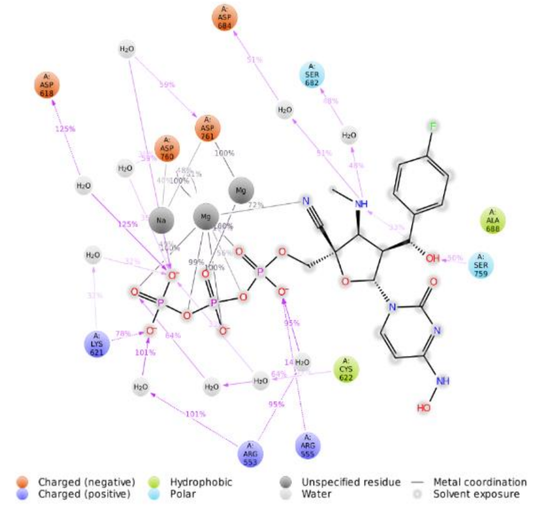

E:17	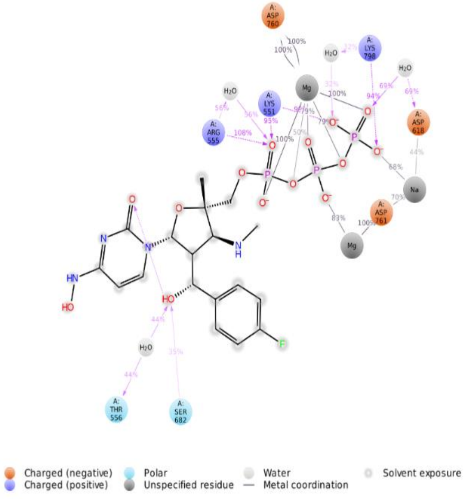	E:18	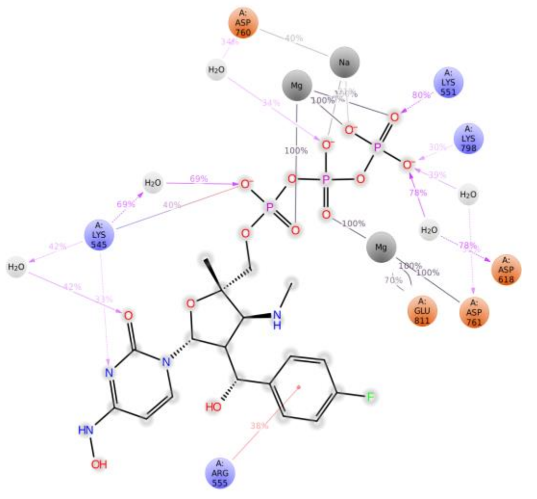
E:20	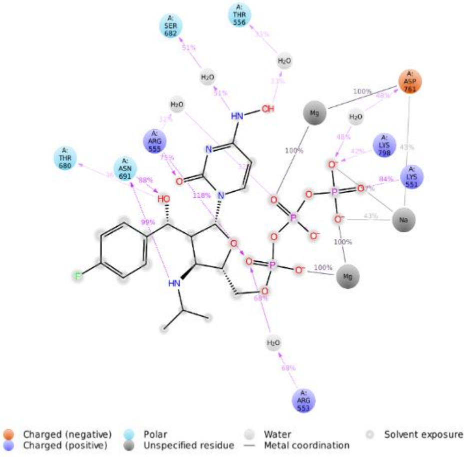	E:22	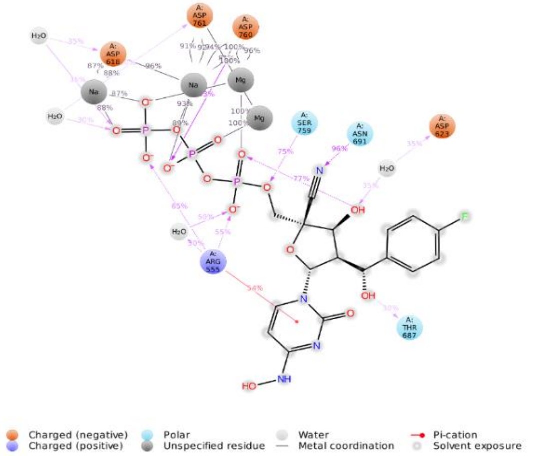
E:24	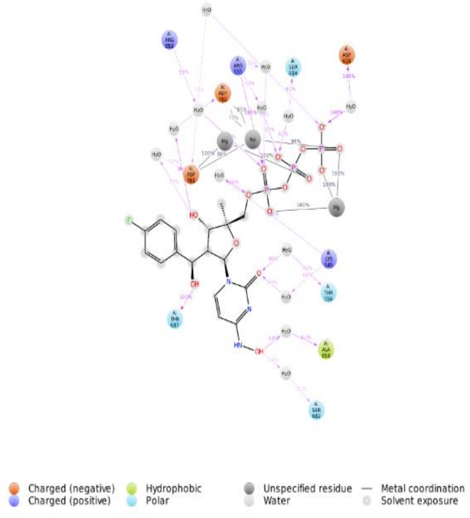	E:26	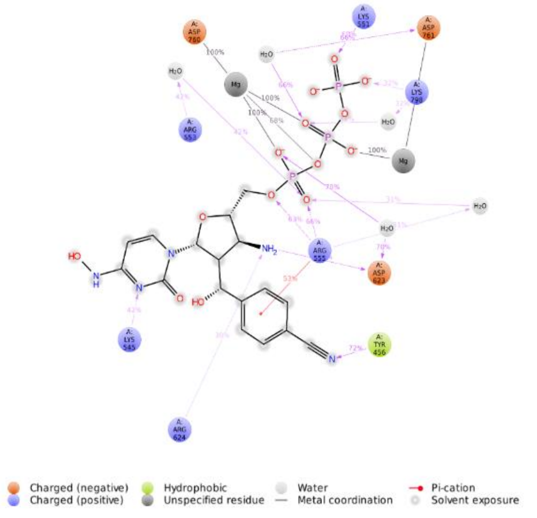

E:27

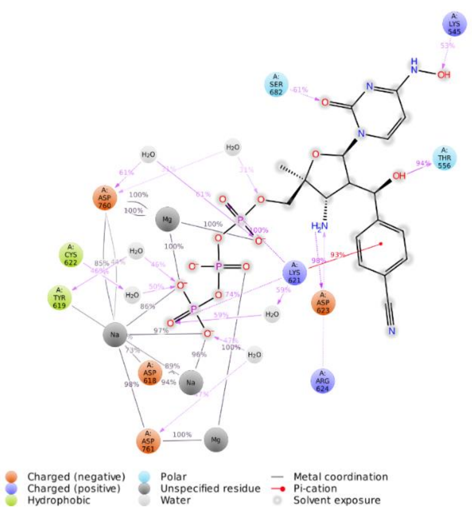

E:30

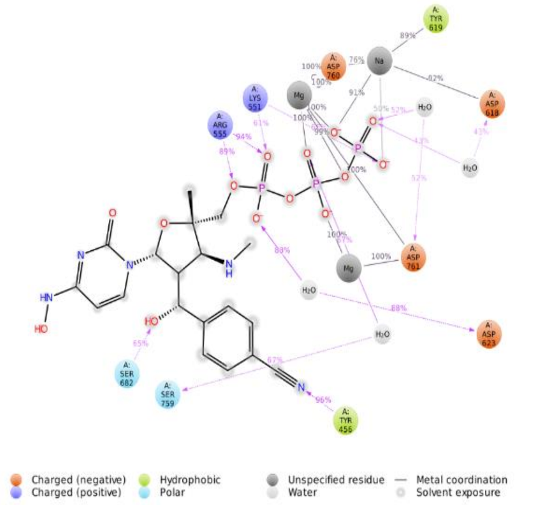

E:32

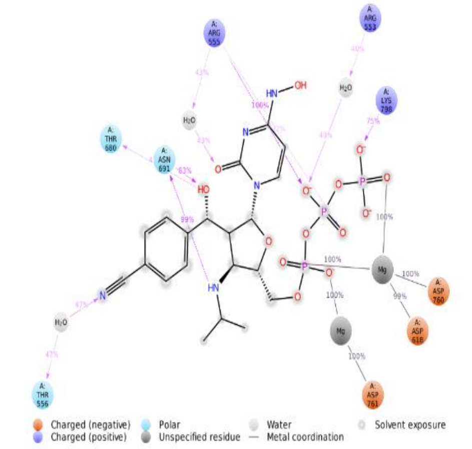

E:33

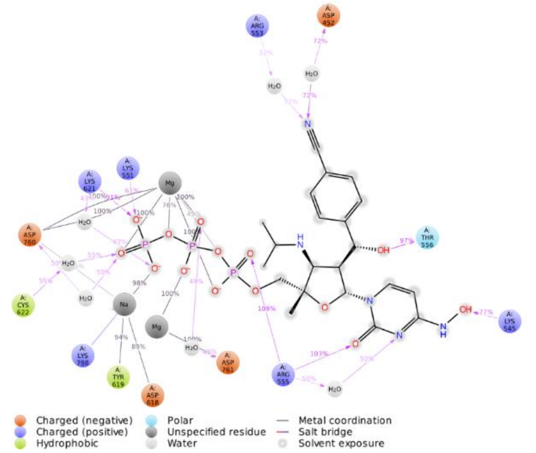

E:34

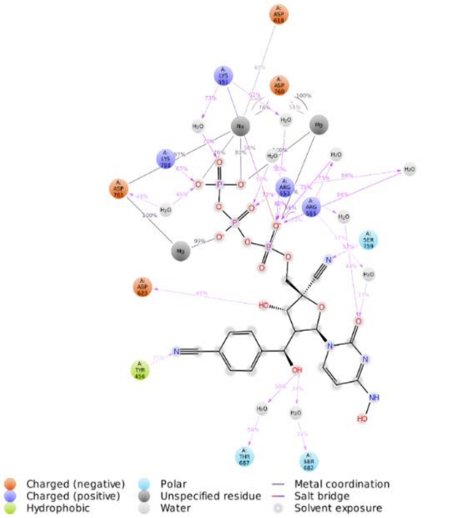

E:35

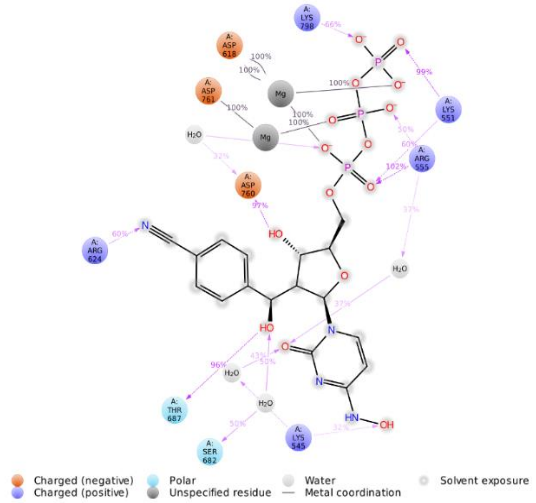

E:36	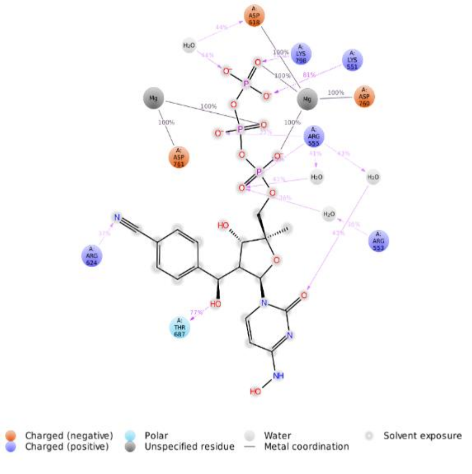	E:37	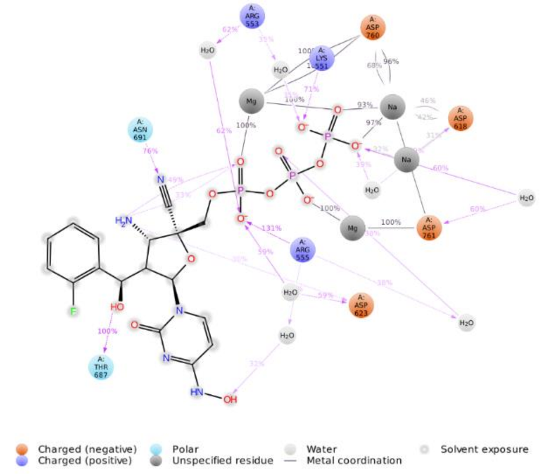
E:38	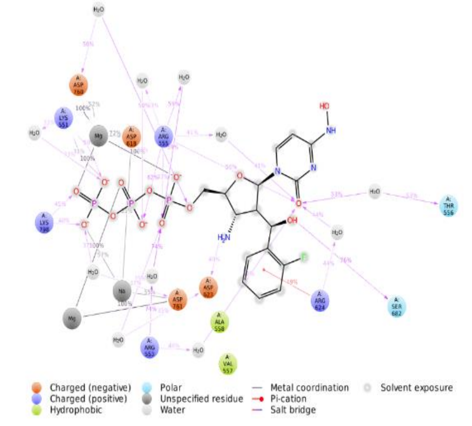	E:39	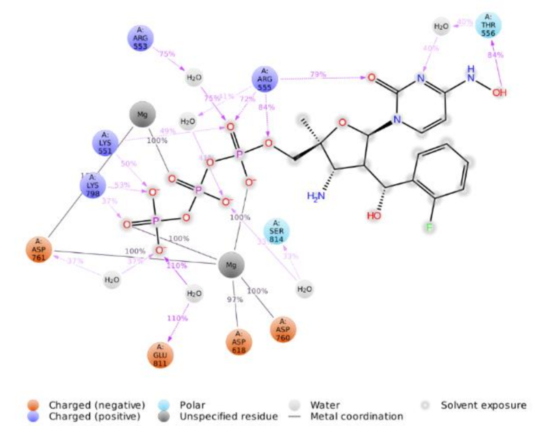
E:40	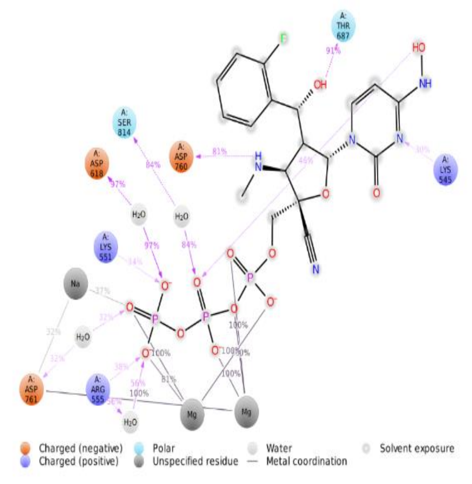	E:41	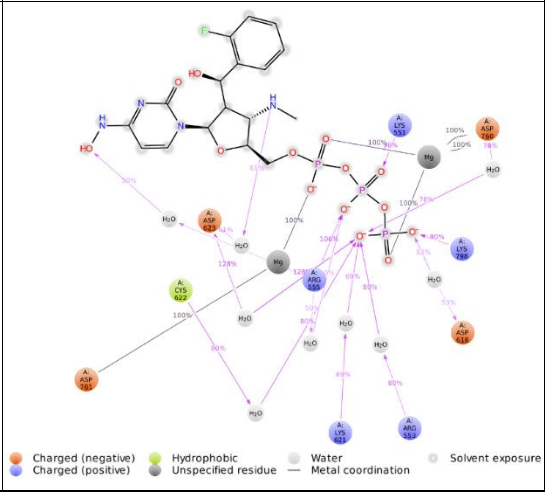

E:42	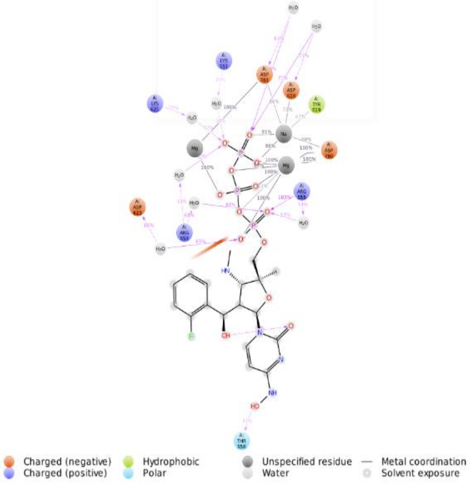	E:43	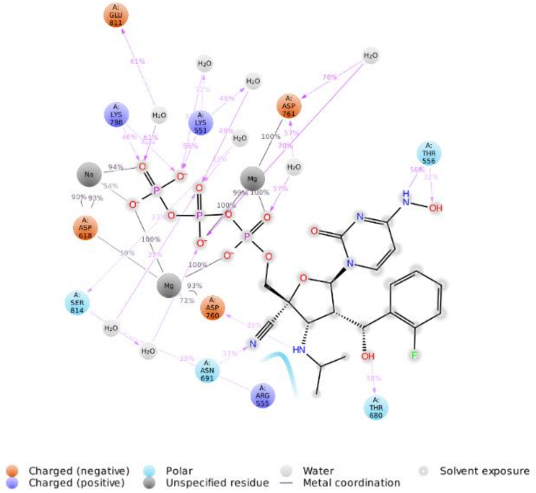
E:46	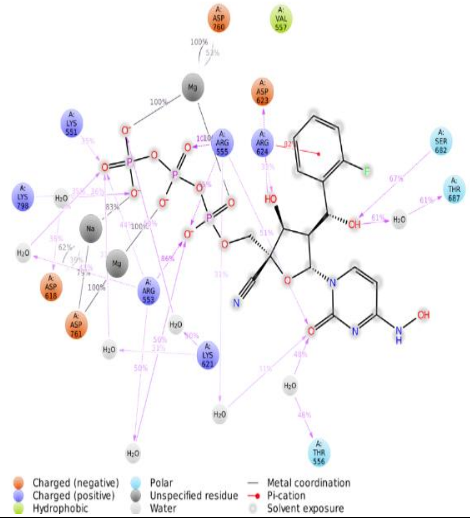	E:47	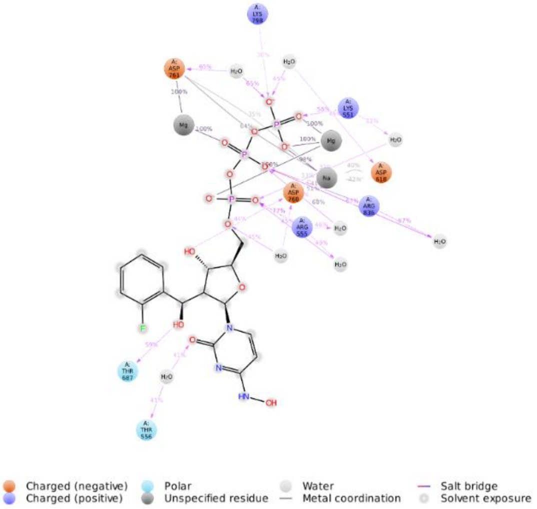
E:48	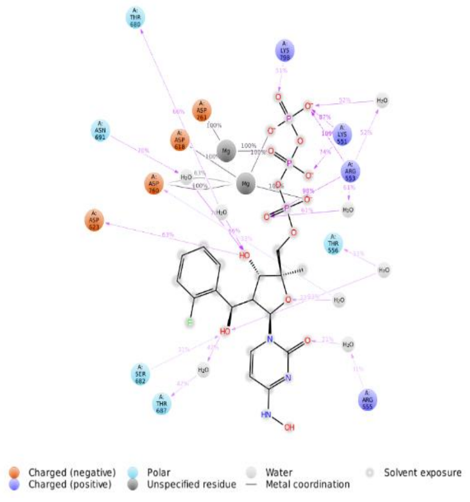	E:49	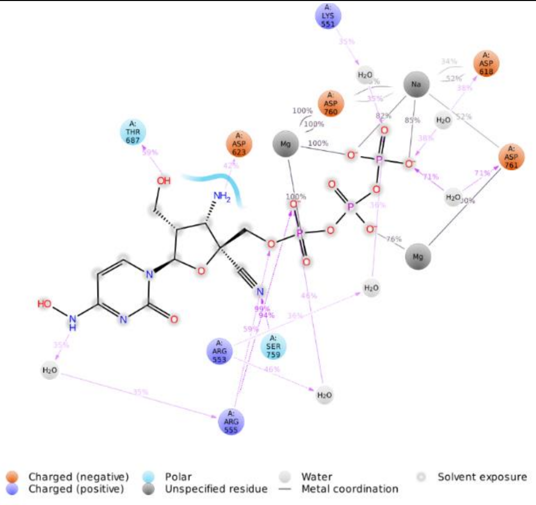

E:50

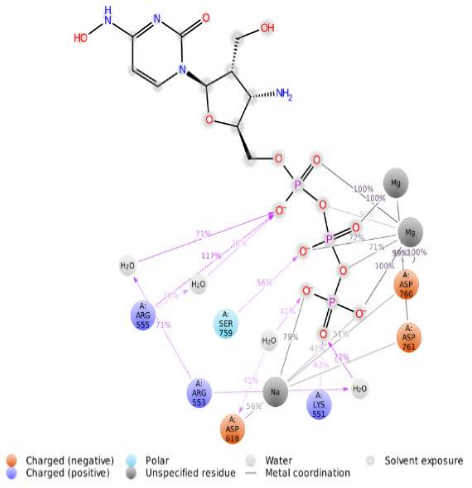

E:51

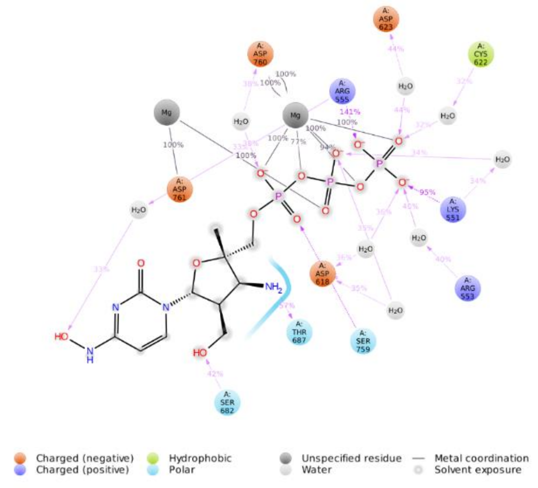

E:52

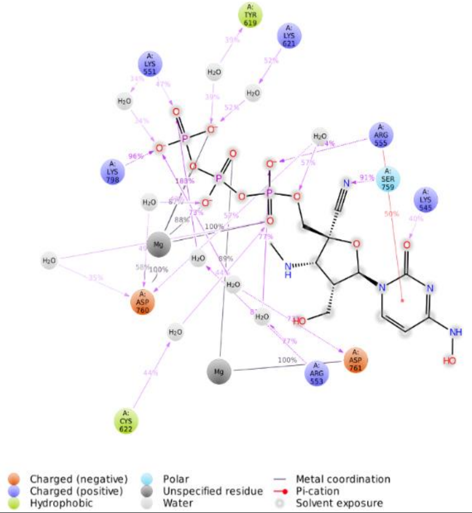

E:53

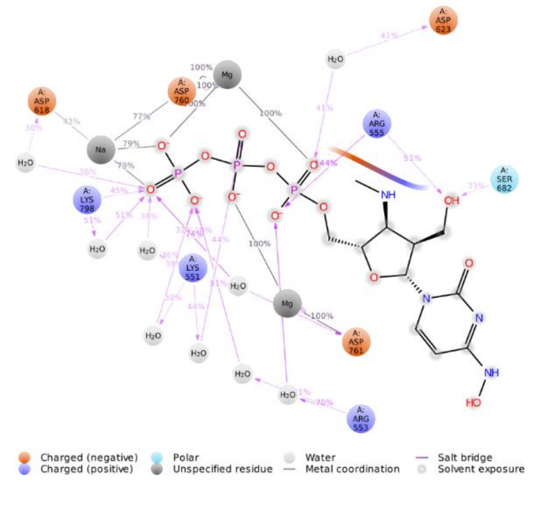

E:54

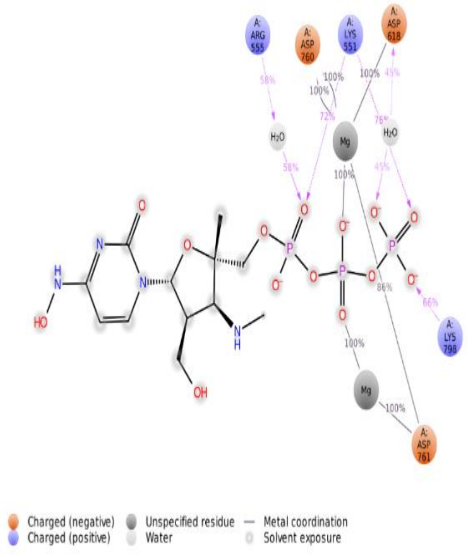

E:55

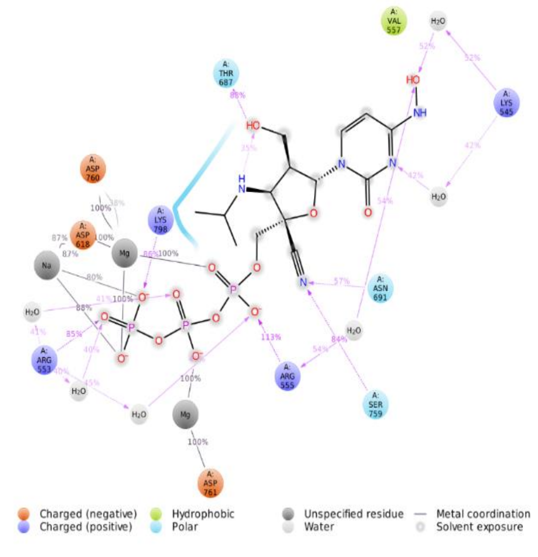

E:56

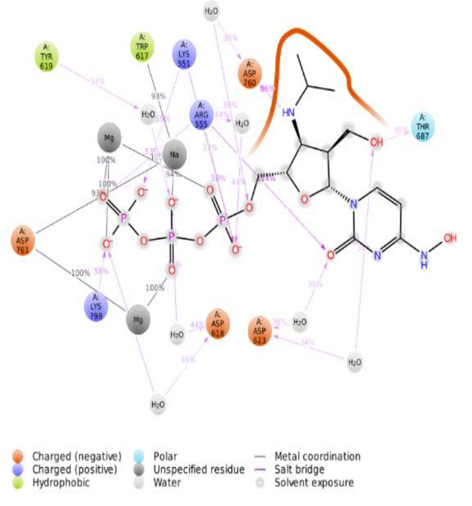

E:57

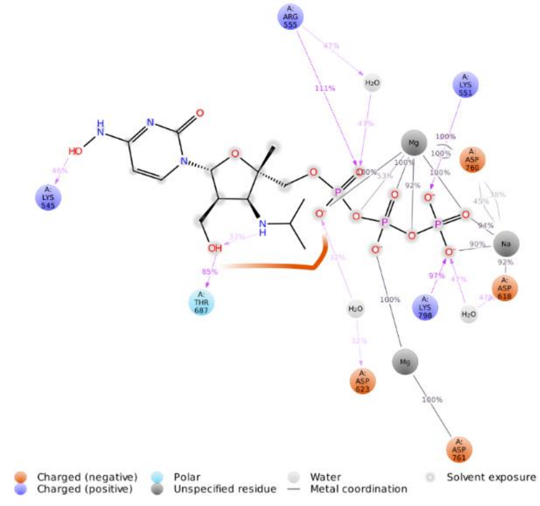

E:58

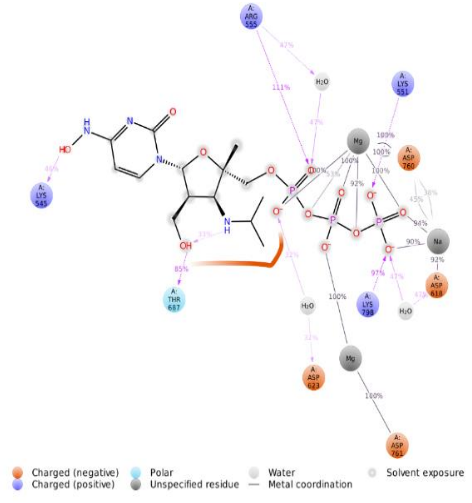

E:59

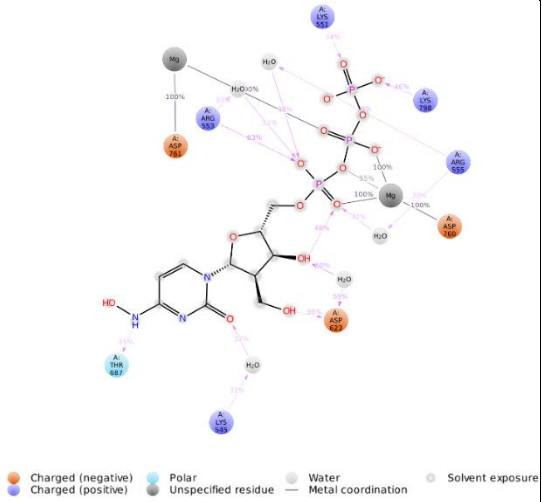

E:60

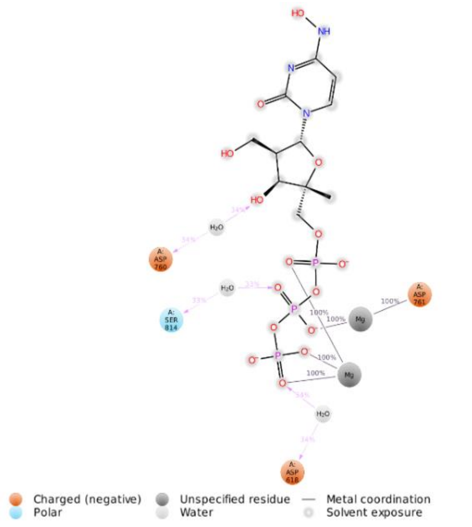

E:61

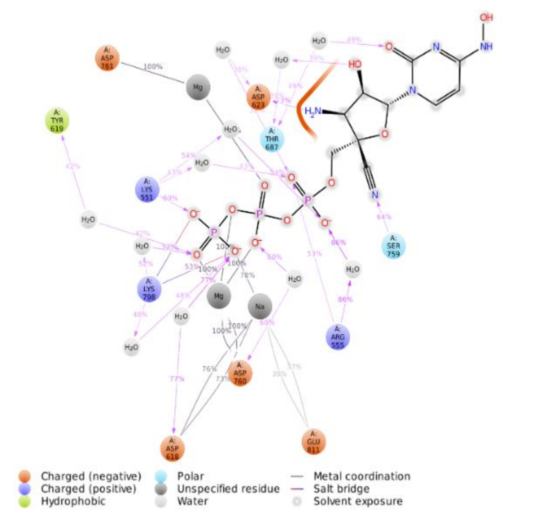

E:62	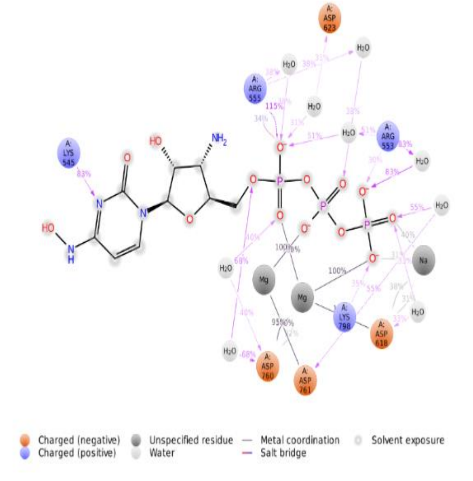	E:63	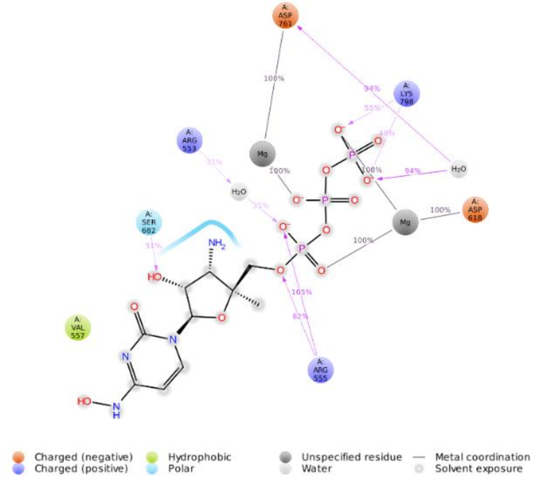
E:64	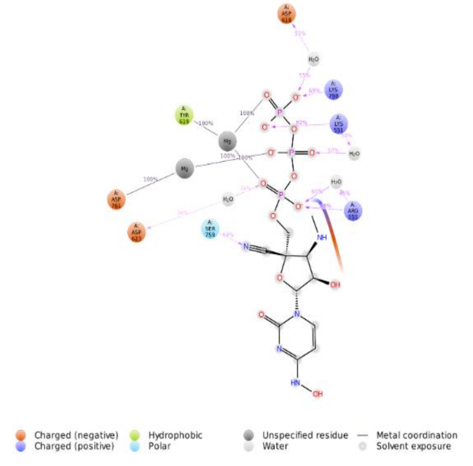	E:65	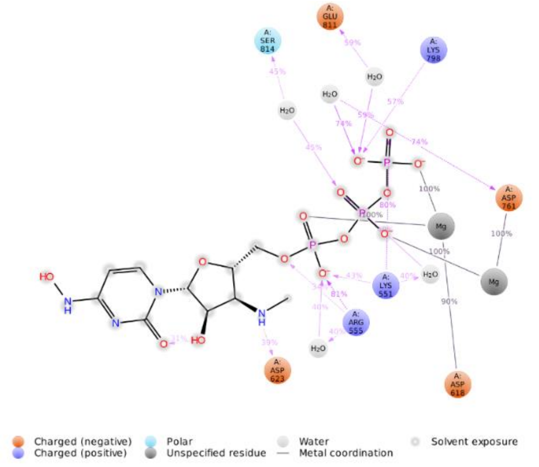
E:66	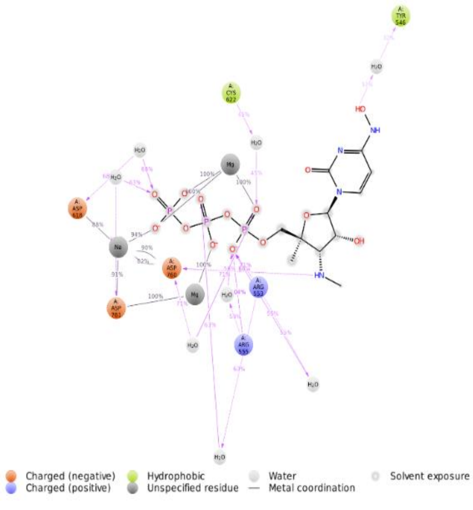	E:67	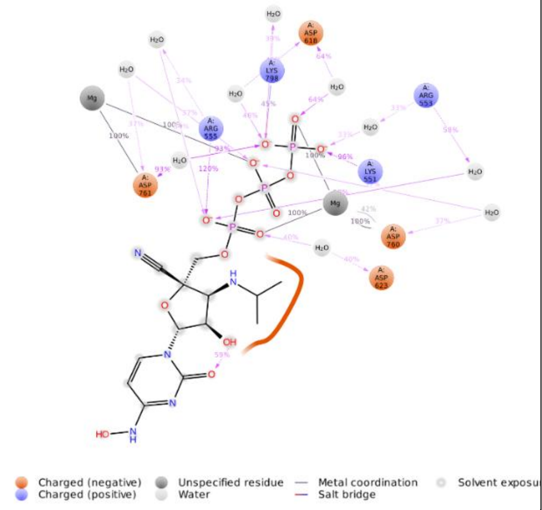

E:68	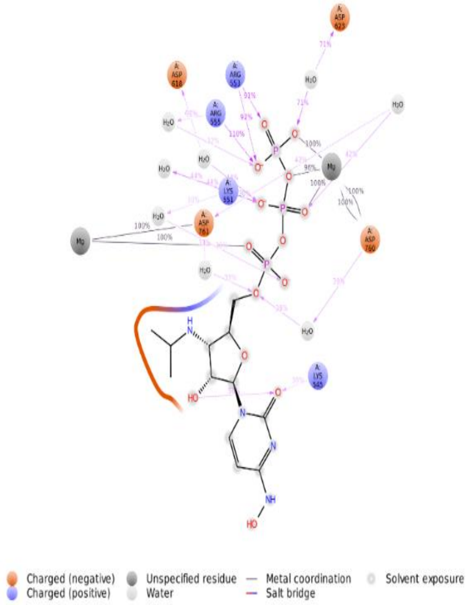	E:69	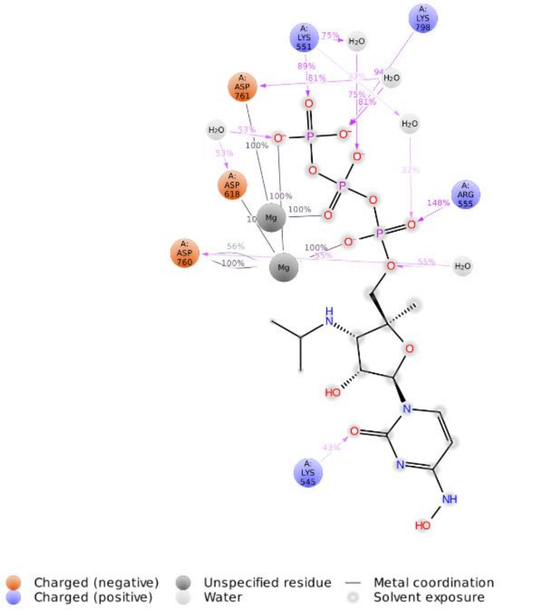
E:70	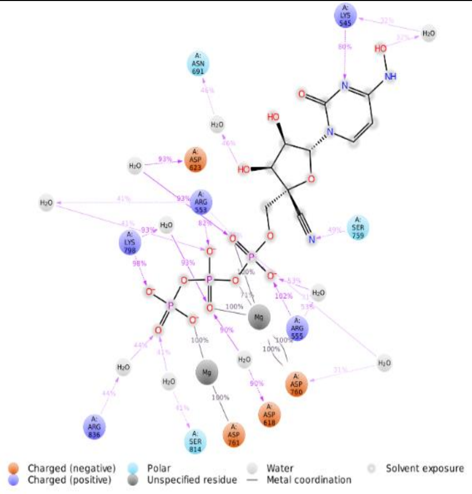	E:72	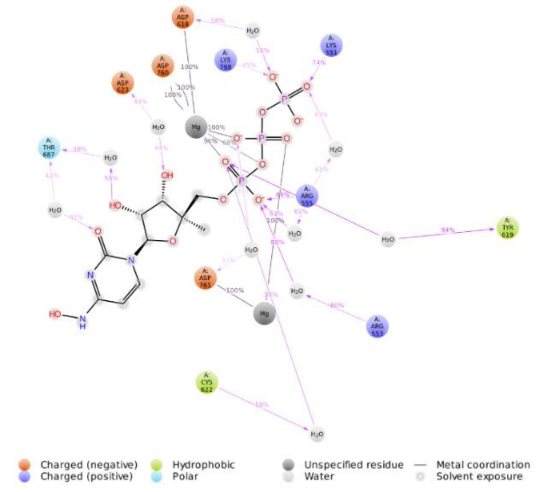

Figure S11. Protein-Ligand contact summaries of all 58 docked NHC-TP analogue systems with ≥30% occurrences shown in the MD simulation.

Ligand #	Ligand properties
**E:63**	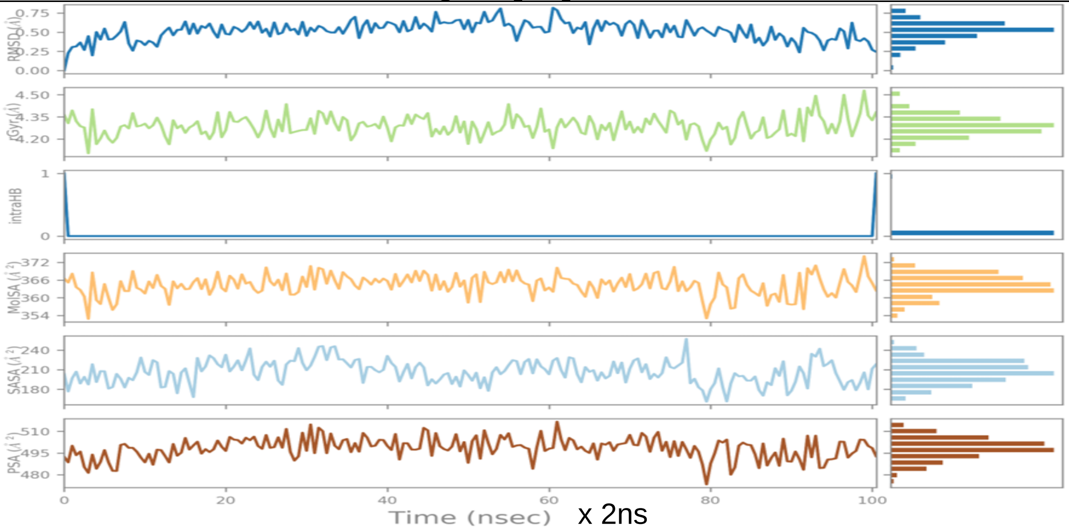
**E:12**	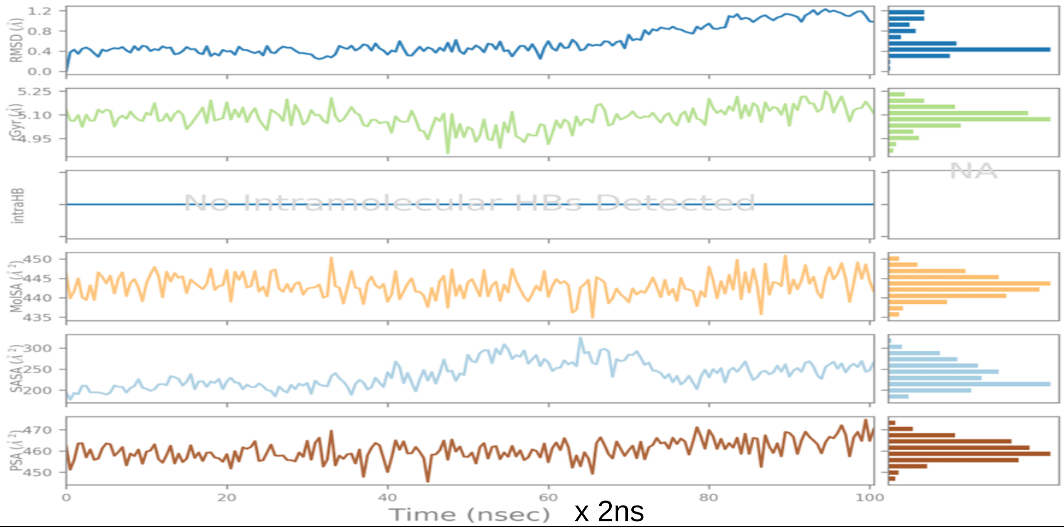
**E:66**	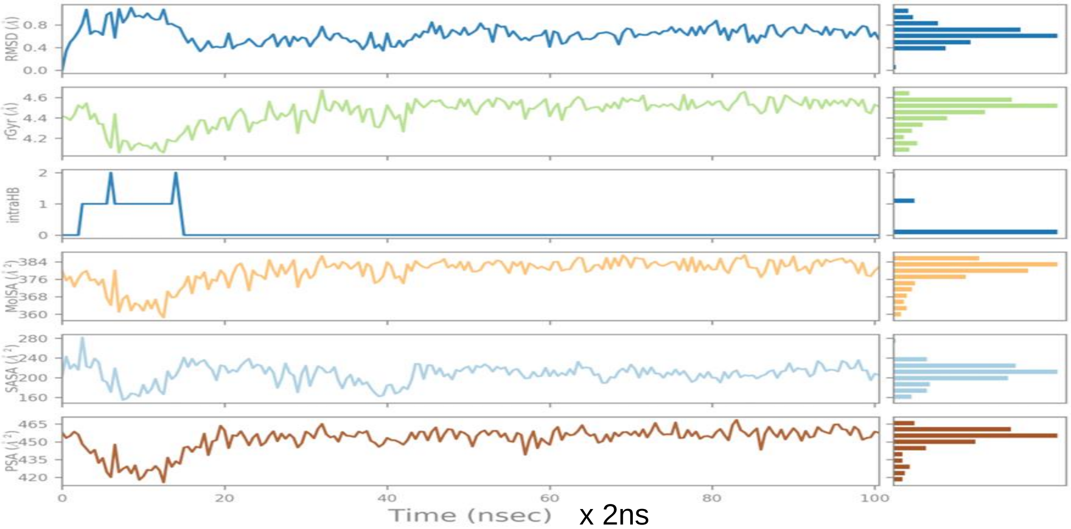

E:65

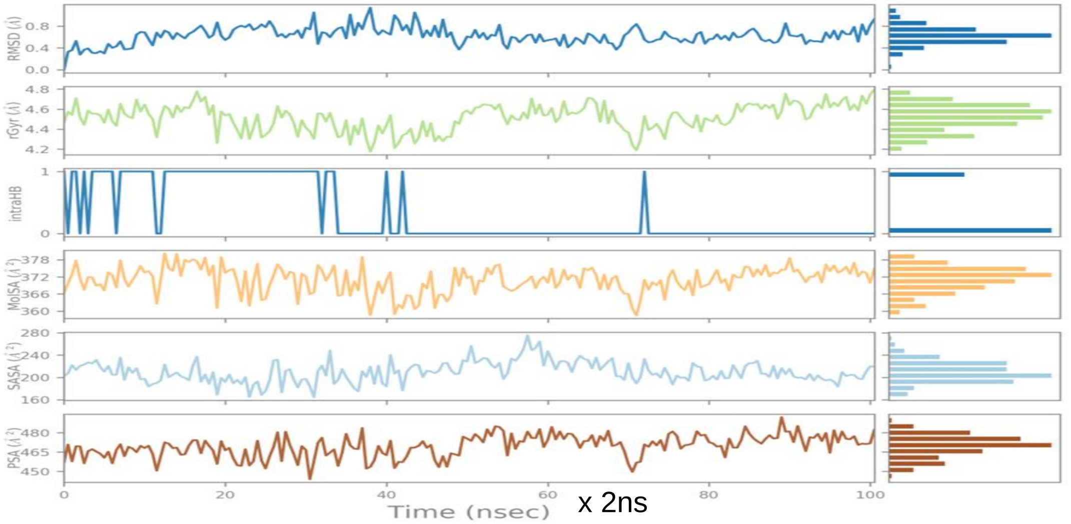

RMSD (Å)
rGyr (Å)
intraHB
MolSA (Å²)
SASA (Å²)
PSA (Å²)
Time (nsec)
x 2ns

E:53

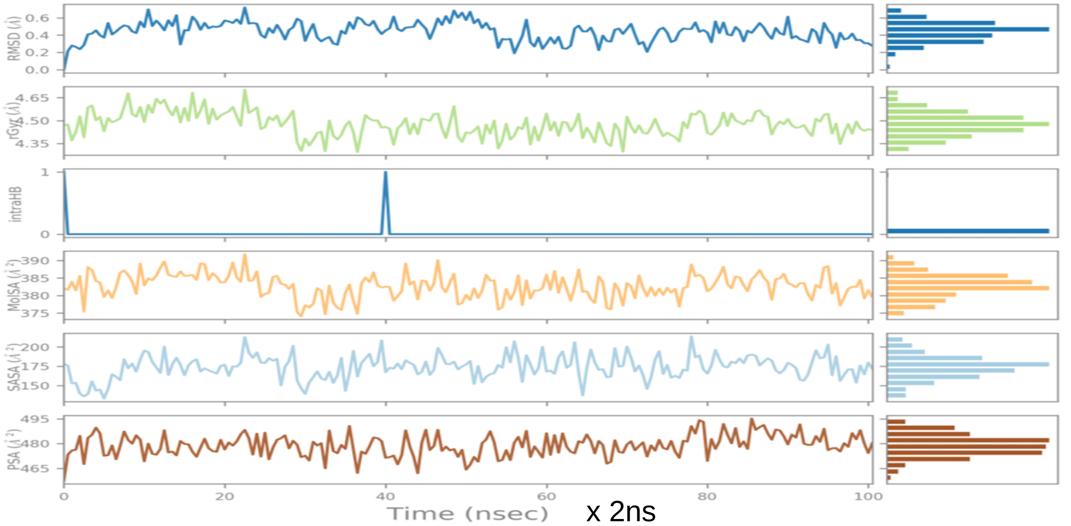

RMSD (Å)
rGyr (Å)
intraHB
MolSA (Å²)
SASA (Å²)
PSA (Å²)
Time (nsec)
x 2ns

E:48

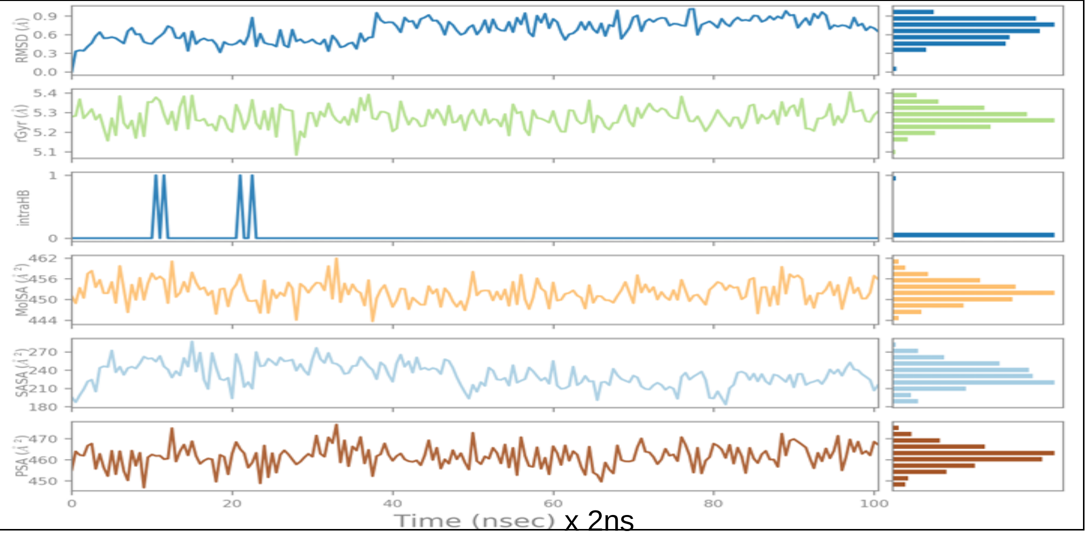

RMSD (Å)
rGyr (Å)
intraHB
MolSA (Å²)
SASA (Å²)
PSA (Å²)
Time (nsec)
x 2ns

**E:03**	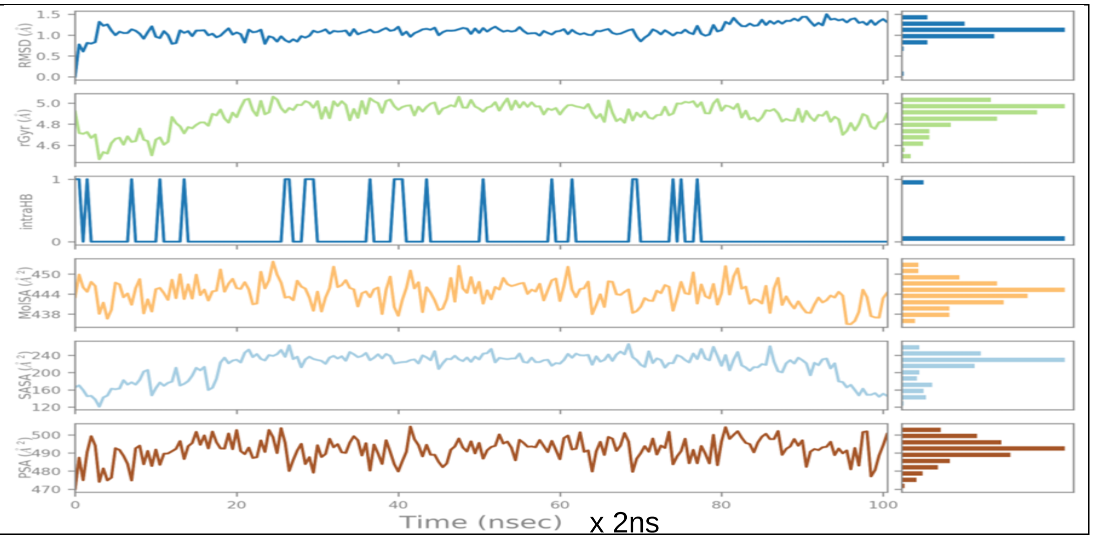

Figure S12. Ligand properties of the top 7 ligands calculated throughout the entirety of the simulation (Ligand RMSD, Radius of gyration (rGyr), intramolecular hydrogen bonds (intraHBs), molecular surface area (MolSA), solvent accessible surface area (SASA) and polar surface area (PSA)).

Ligand	Ligand RMSF	Ligand	Ligand RMSF
E:71 (NHC-TP)	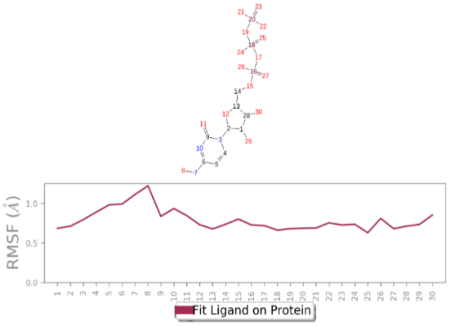	E:01	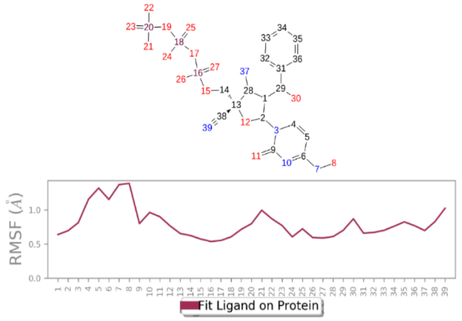
E:03	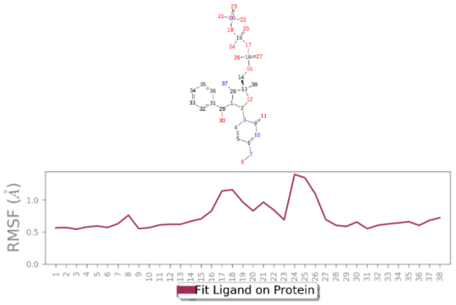	E:04	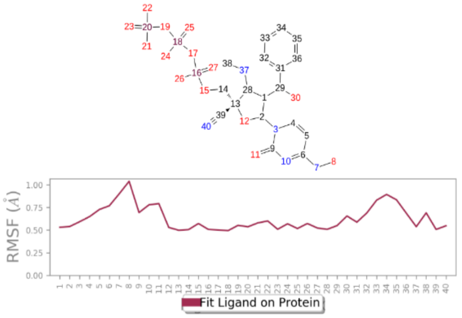
E:06	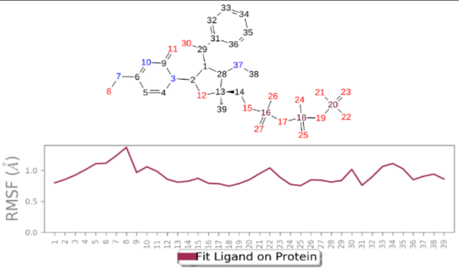	E:07	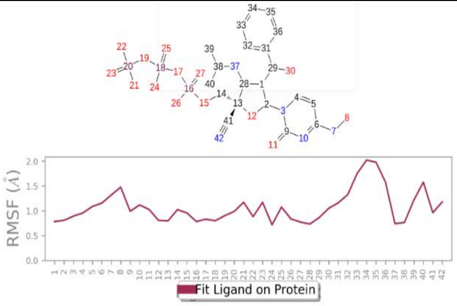
E:08	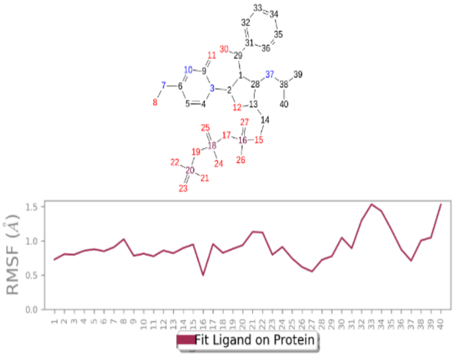	E:10	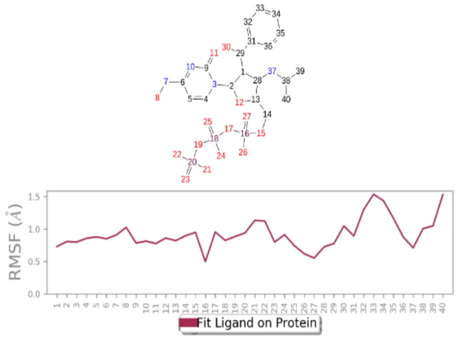

E:11	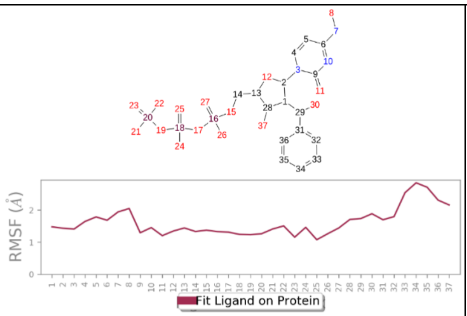	E:12	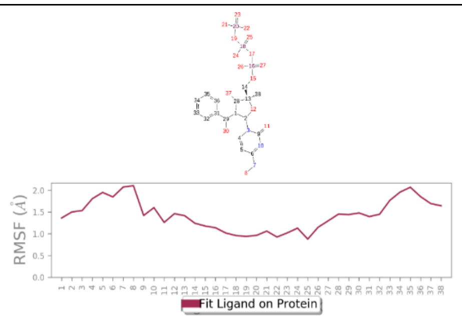
E:15	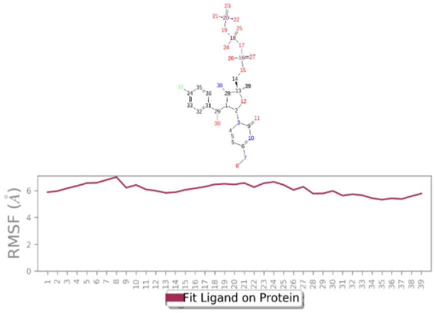	E:16	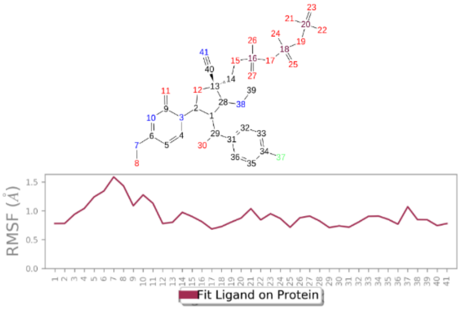
E:17	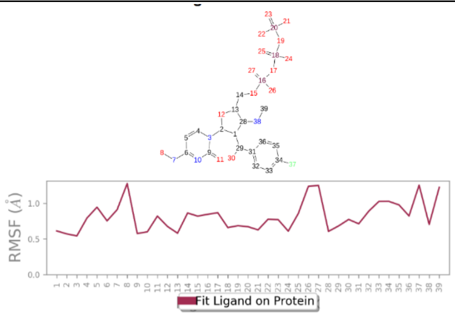	E:18	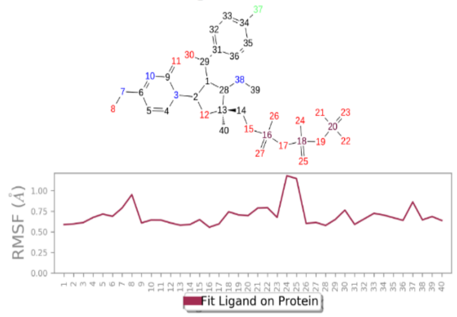
E:20	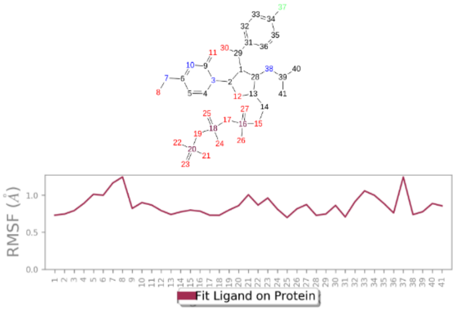	E:22	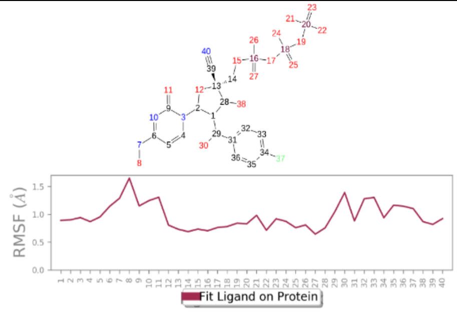
E:24	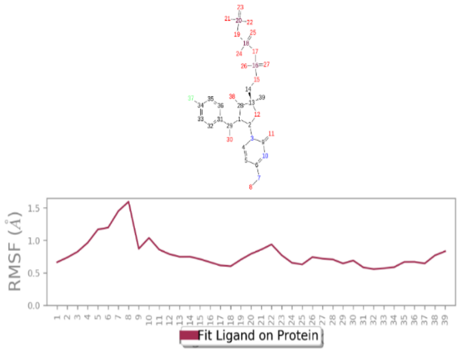	E:26	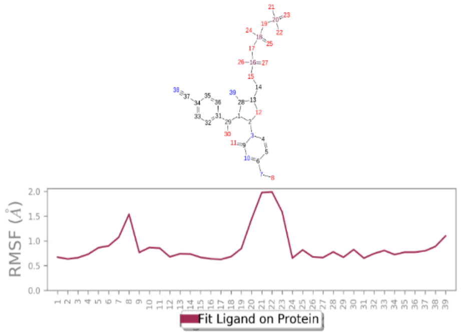

E:27	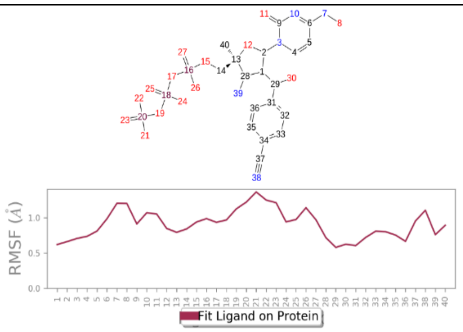	E:30	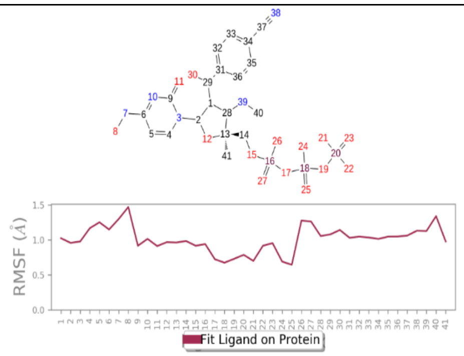
E:32	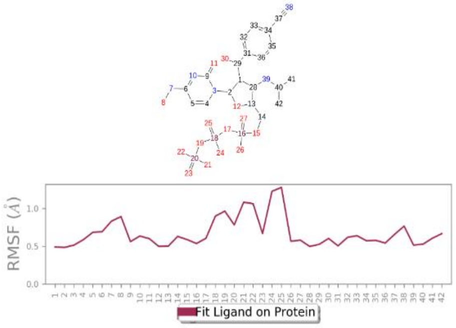	E:33	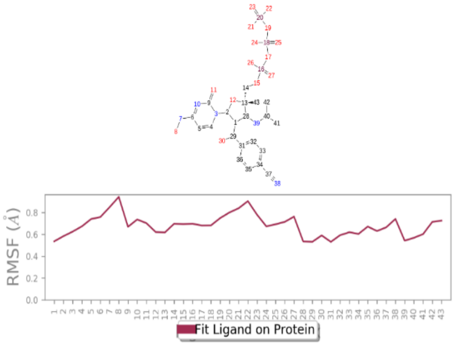
E:34	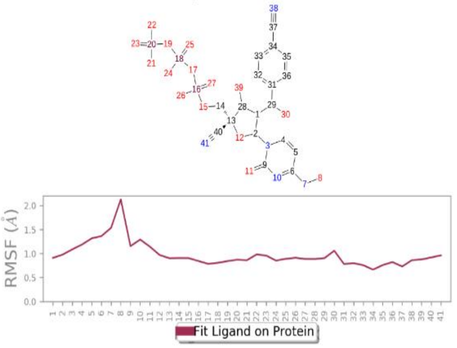	E:35	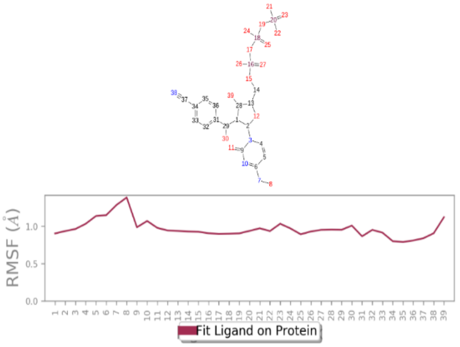
E:36	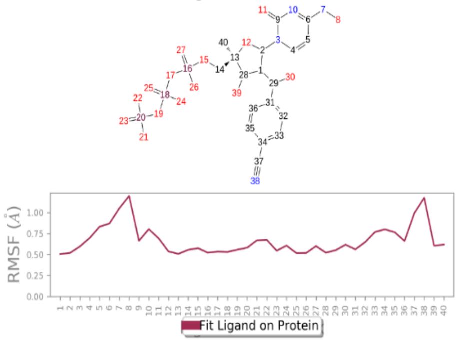	E:37	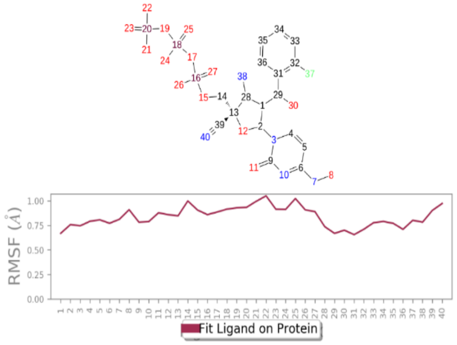

E:38	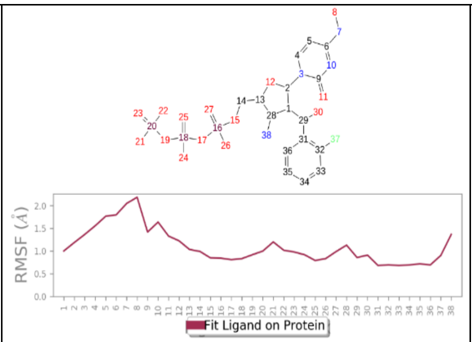	E:39	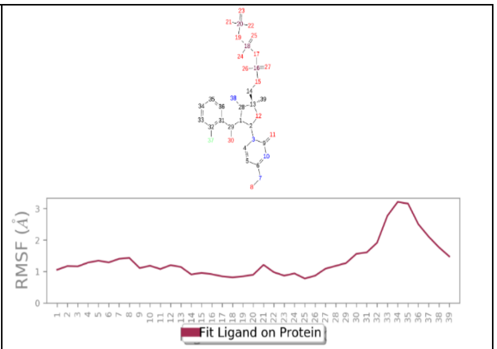
E:40	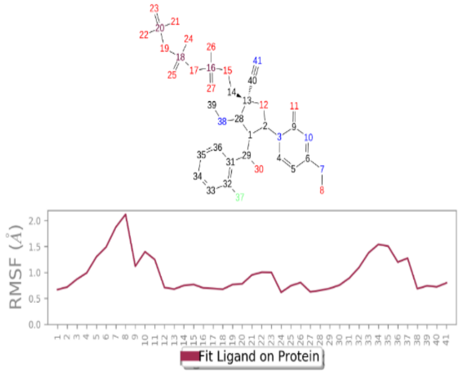	E:41	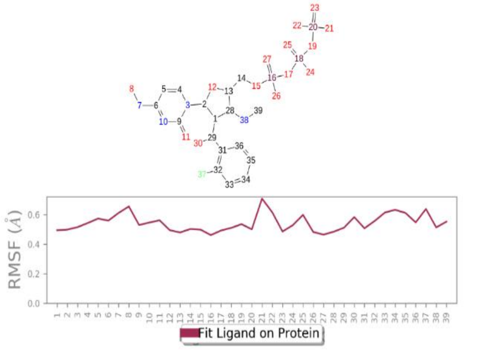
E:42	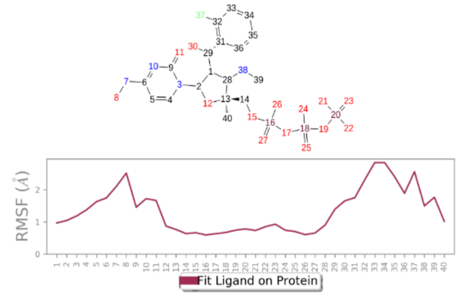	E:43	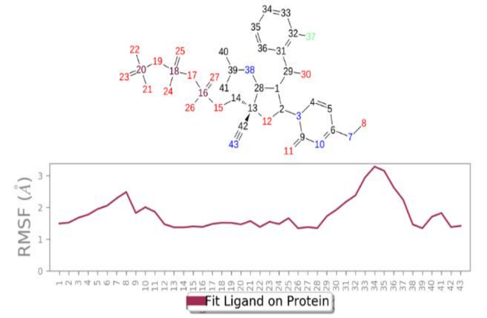
E:46	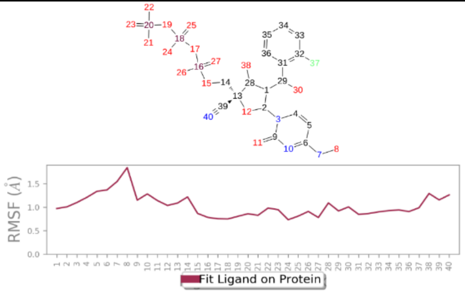	E:47	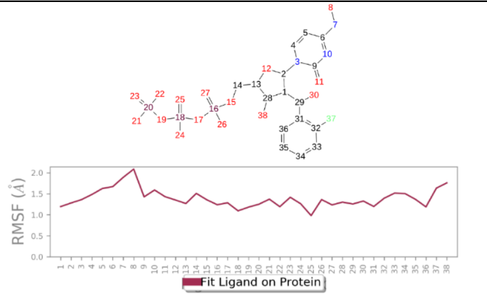
E:48	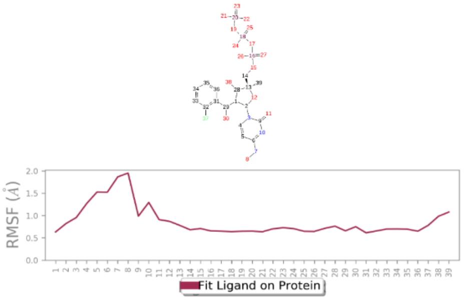	E:49	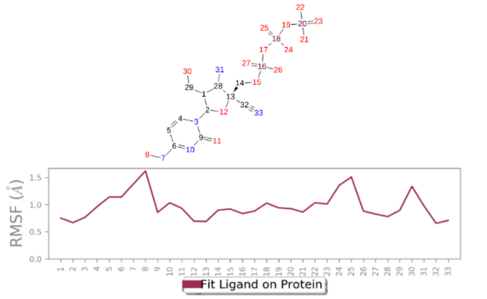

E:50	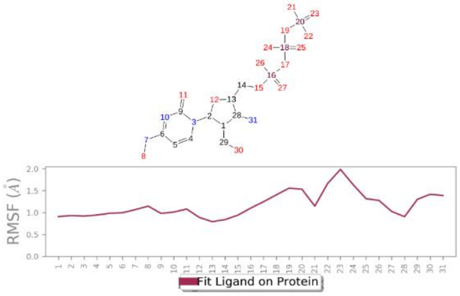	E:51	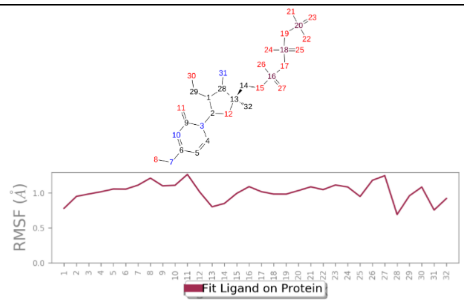
E:52	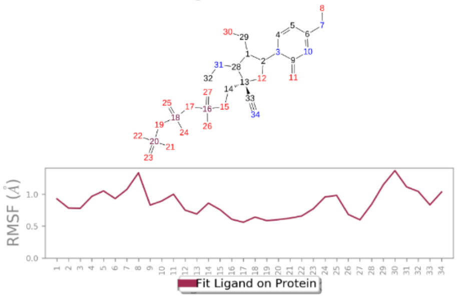	E:53	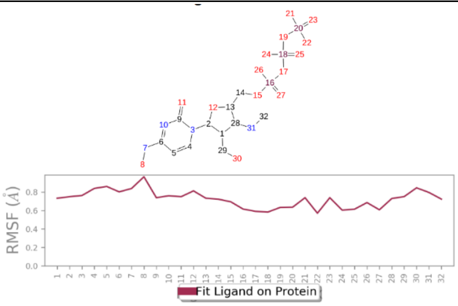
E:54	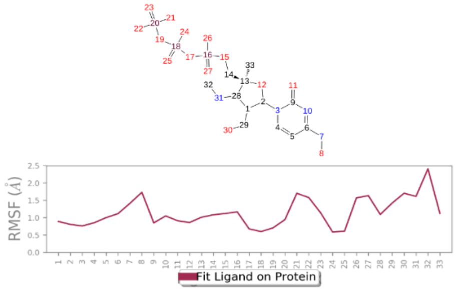	E:55	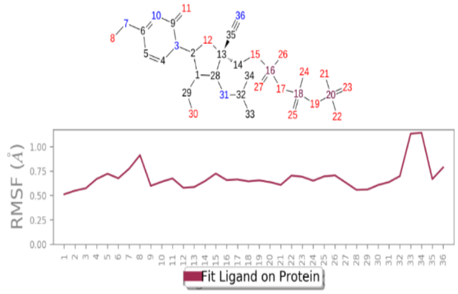
E:56	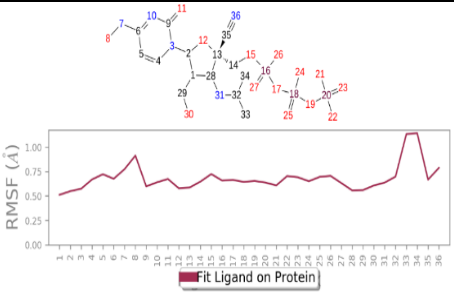	E:57	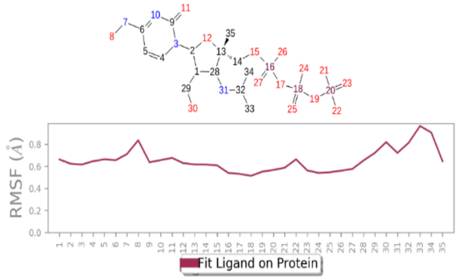
E:58	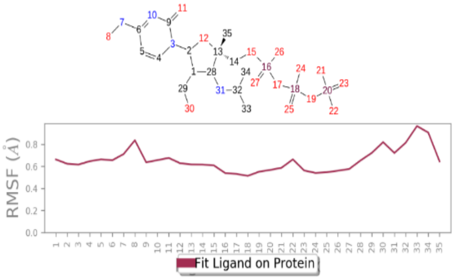	E:59	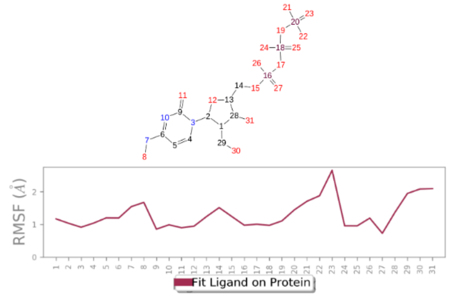

E:60	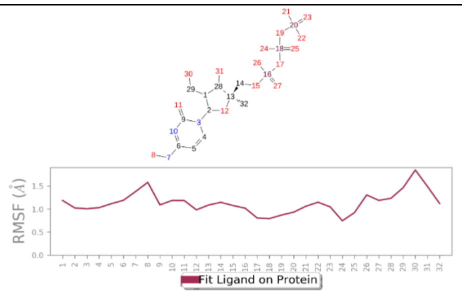	E:61	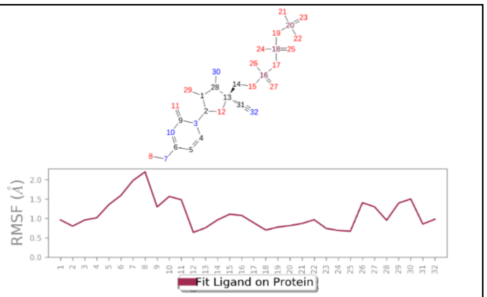
E:62	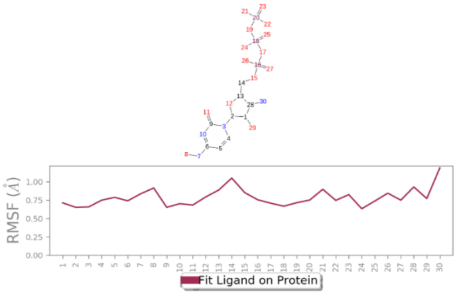	E:63	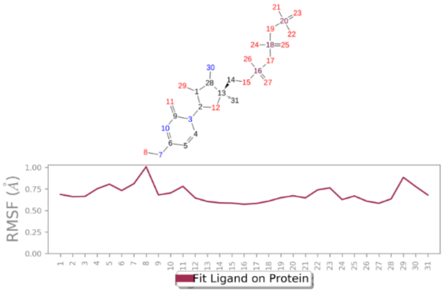
E:64	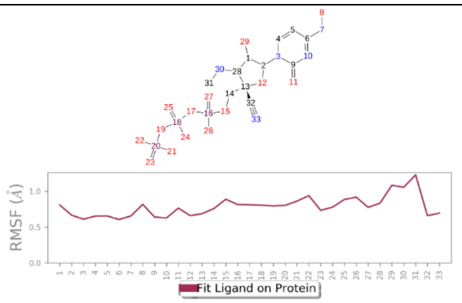	E:65	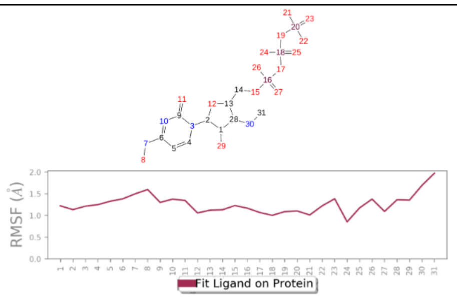
E:66	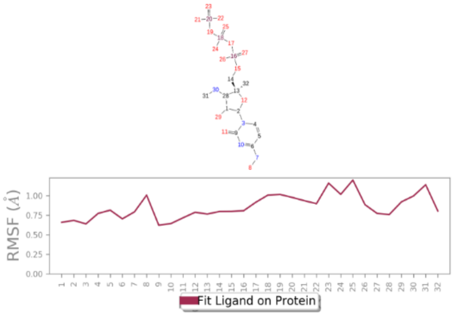	E:67	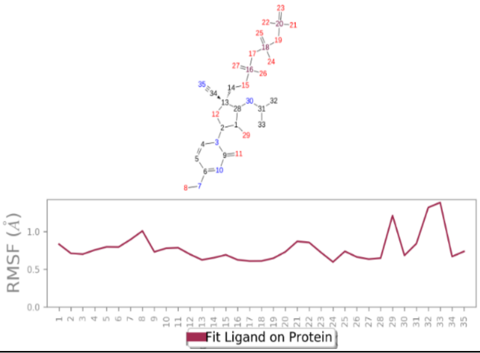
E:68	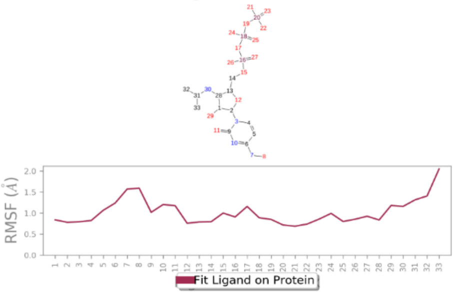	E:69	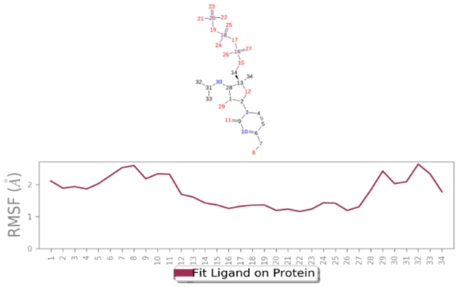

E:70	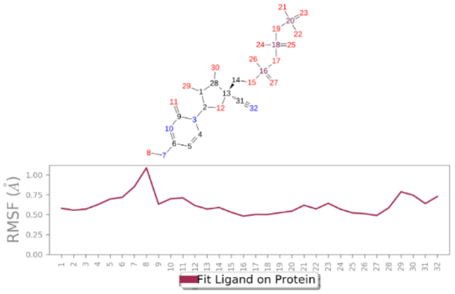	E:72	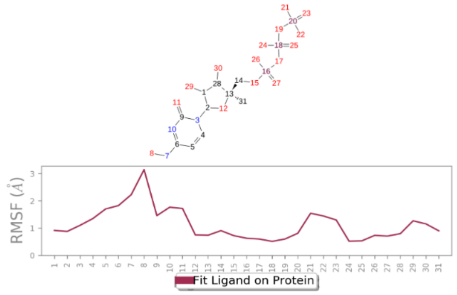

Figure S13. Ligand RMSF of 58 NHC-TP analogue systems from MD simulations.

Ligand	L/P Contacts Histogram	Ligand	L/P Contacts Histogram
E:71 (NHC-TP)	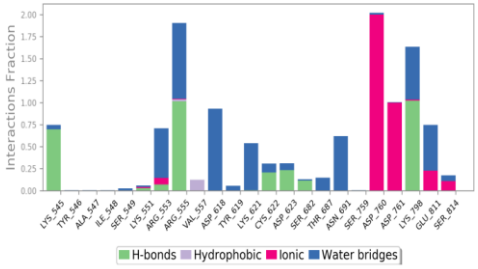	E:01	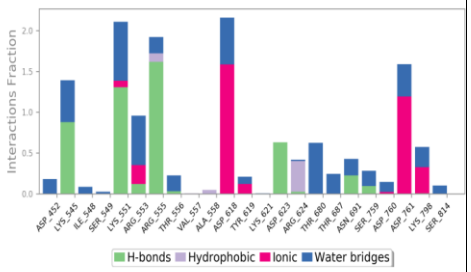
E:03	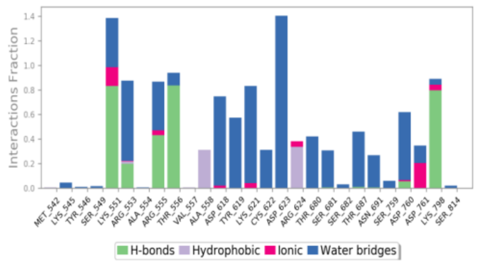	E:04	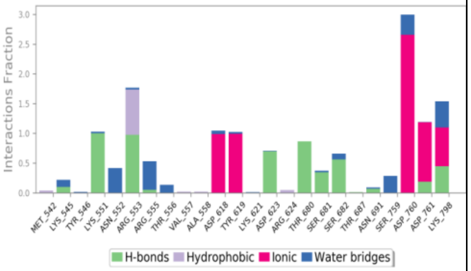
E:06	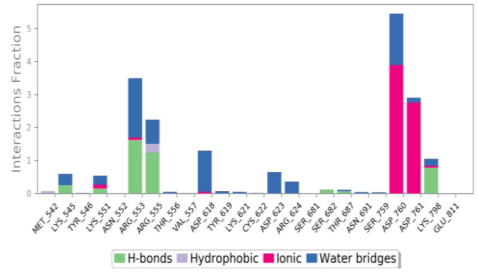	E:07	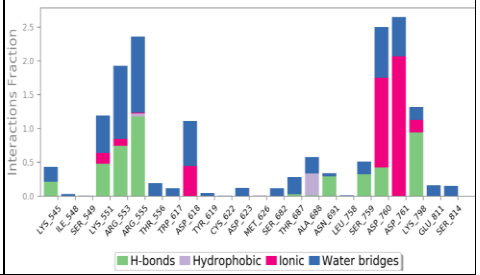
E:08	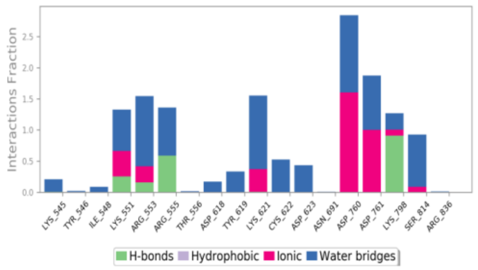	E:10	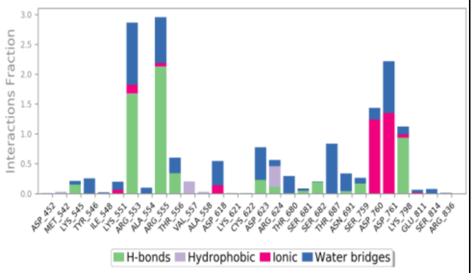
E:11	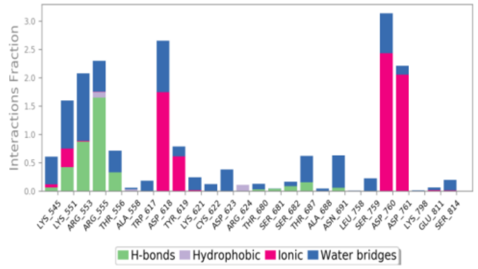	E:12	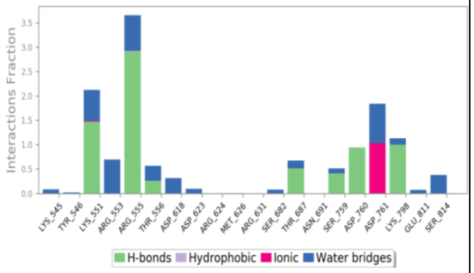
E:15	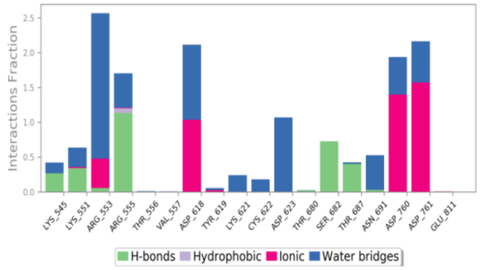	E:16	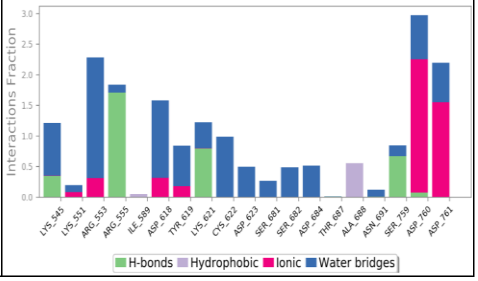

E:17	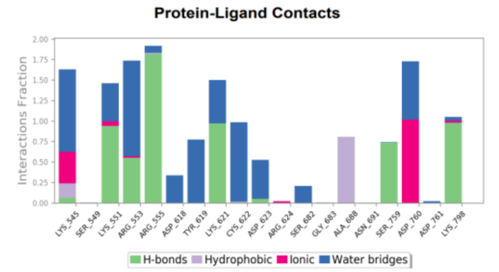	E:18	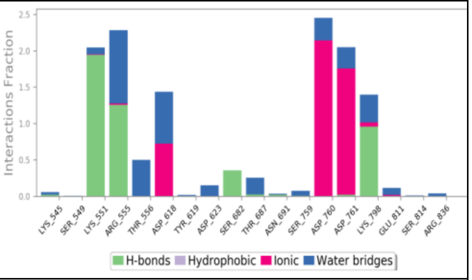
E:20	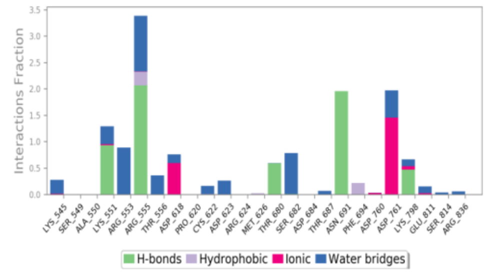	E:22	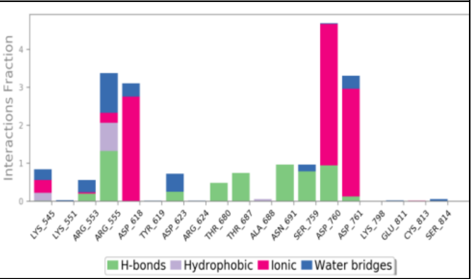
E:24	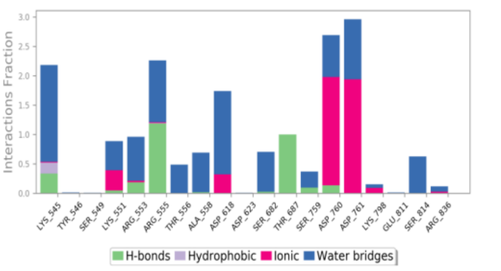	E:26	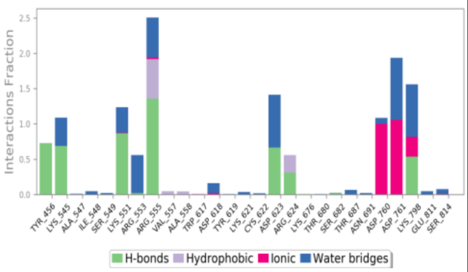
E:27	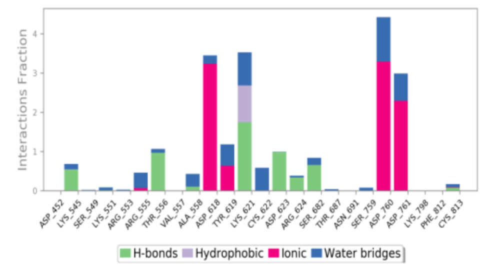	E:30	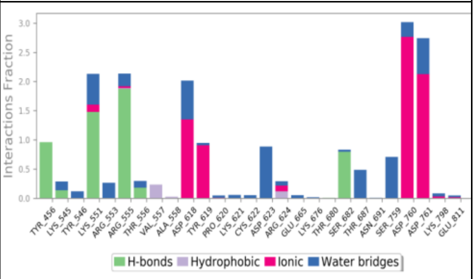
E:32	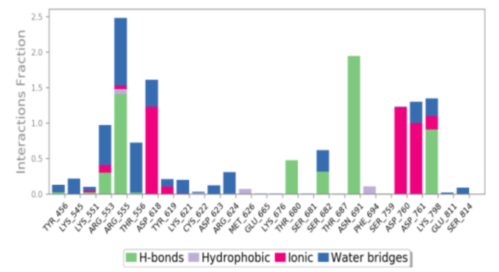	E:33	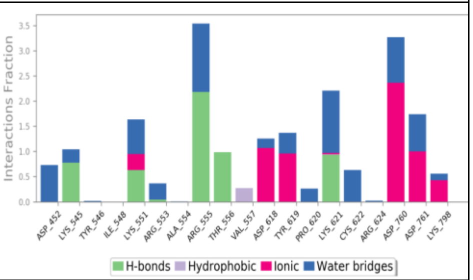
E:34	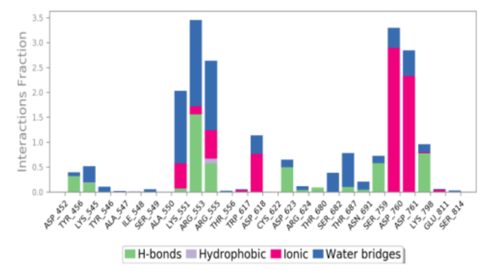	E:35	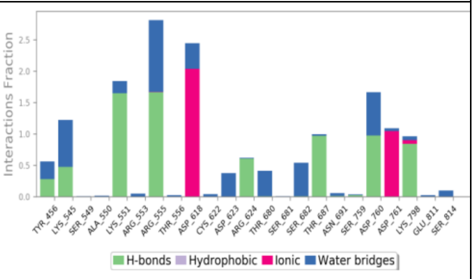

E:36	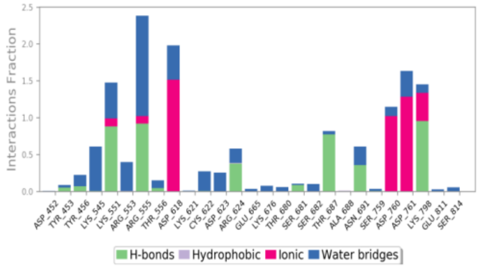	E:37	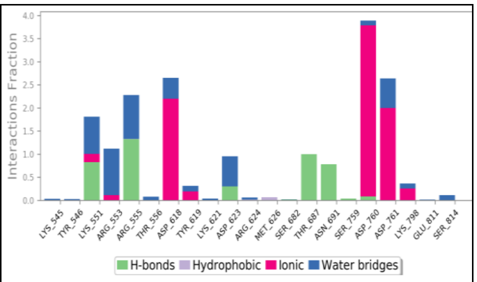
E:38	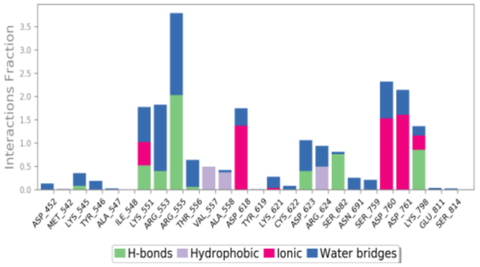	E:39	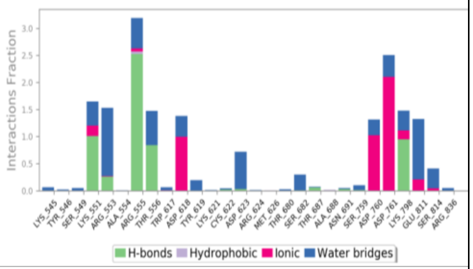
E:40	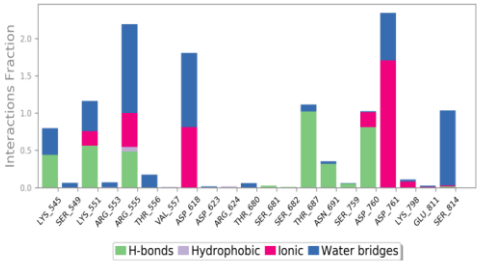	E:41	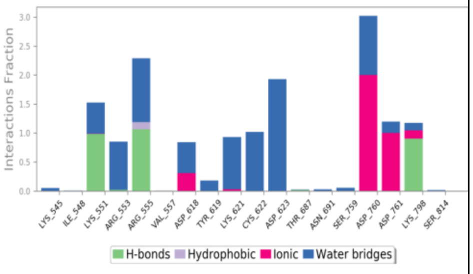
E:42	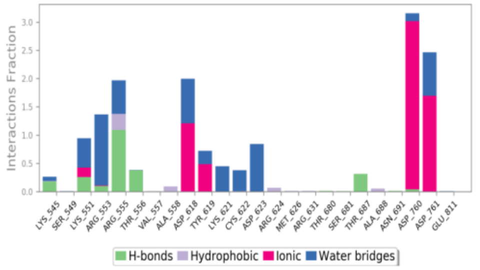	E:43	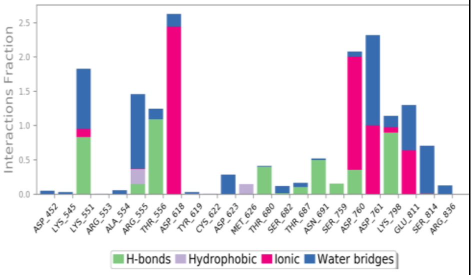
E:46	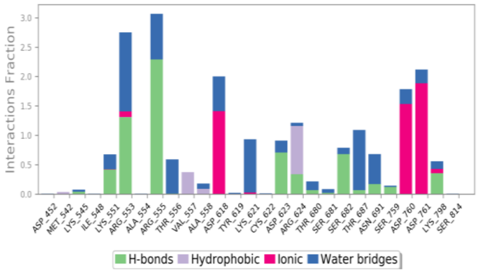	E:47	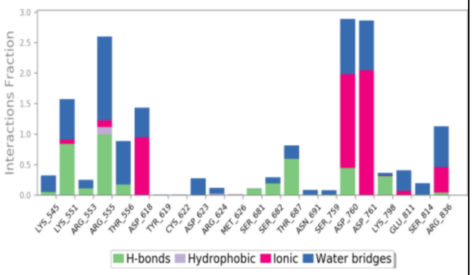
E:48	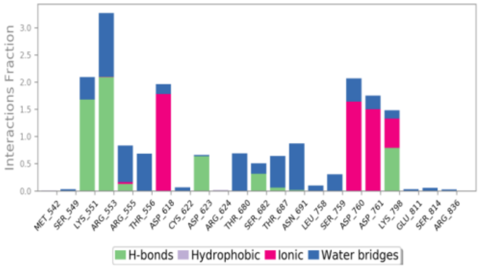	E:49	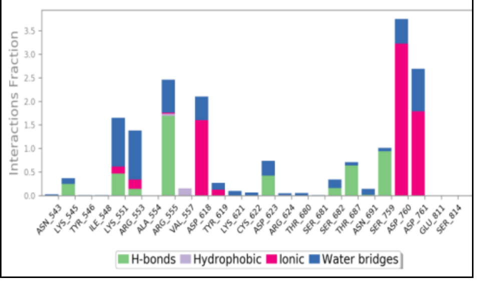

E:50	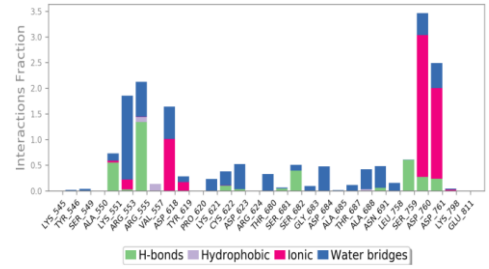	E:51	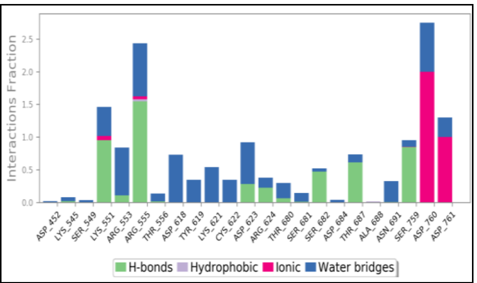
E:52	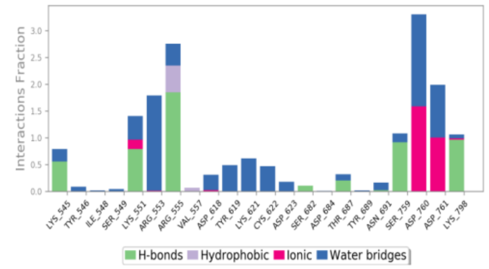	E:53	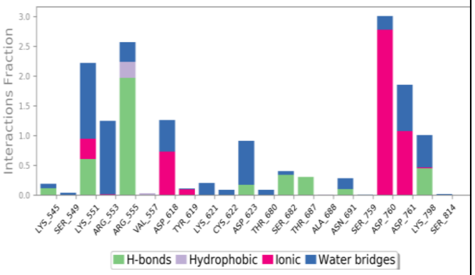
E:54	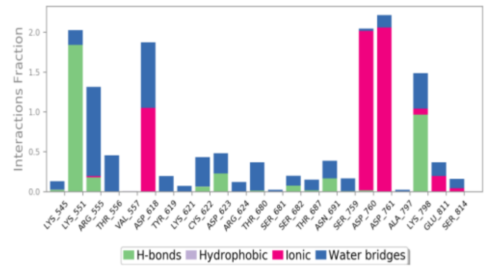	E:55	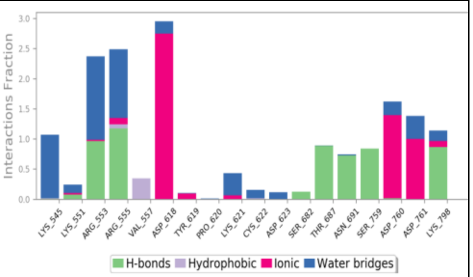
E:56	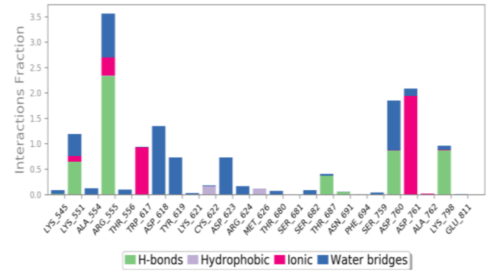	E:57	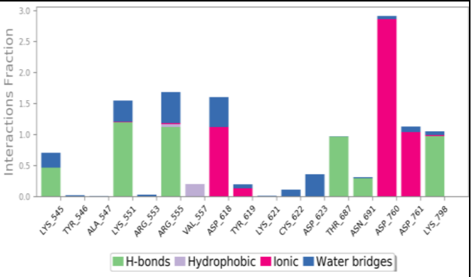
E:58	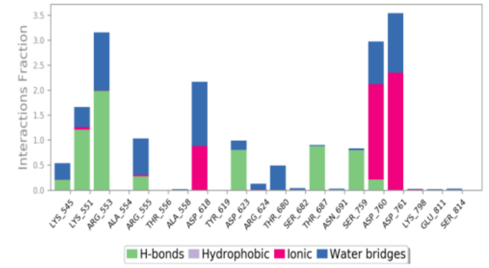	E:59	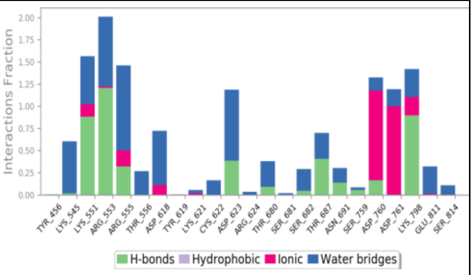
E:60	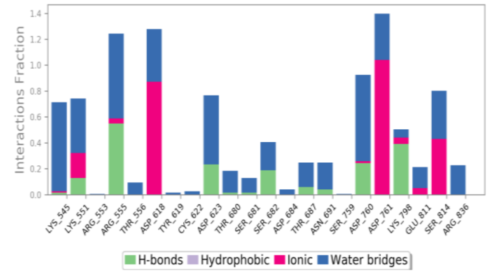	E:61	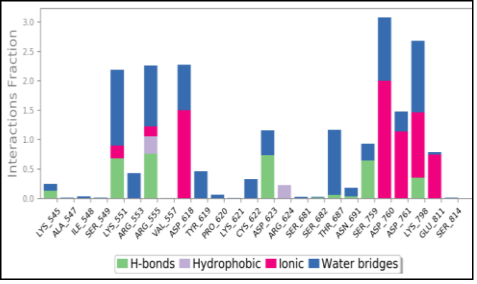

E:62

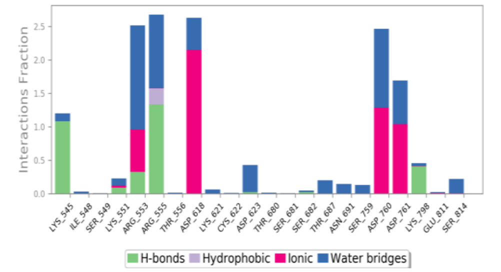

E:63

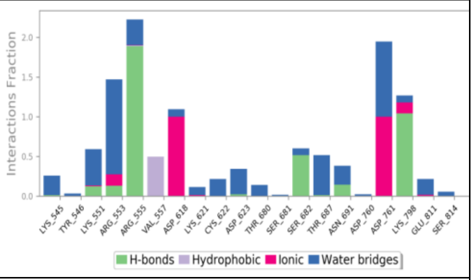

E:64

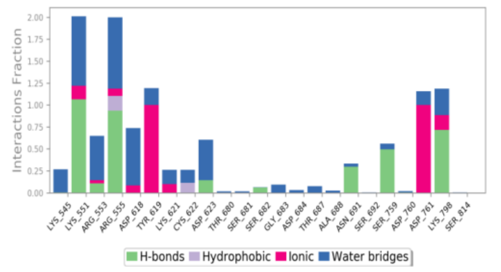

E:65

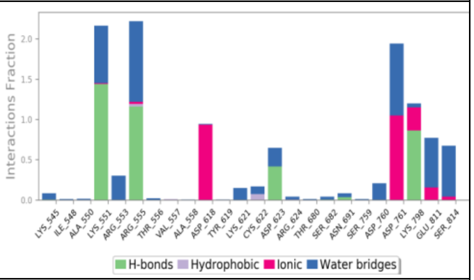

E:66

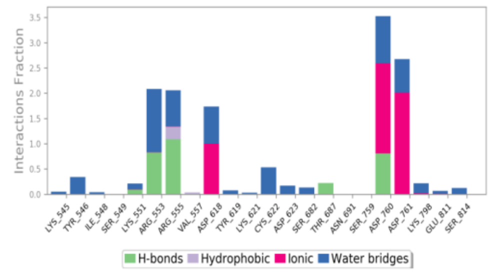

E:67

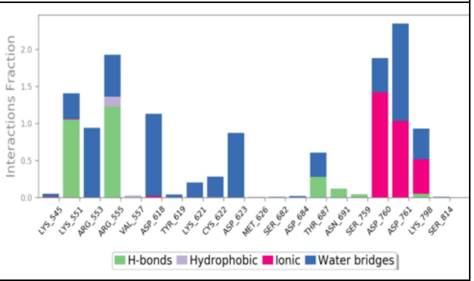

E:68

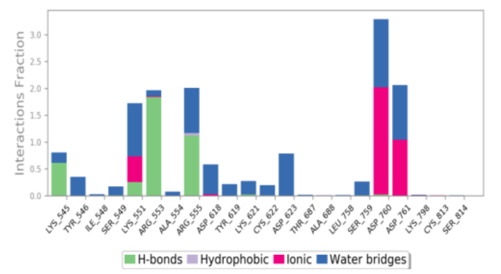

E:69

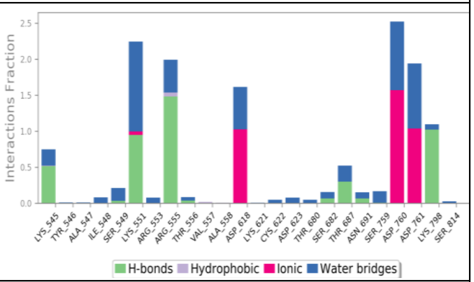

E:70

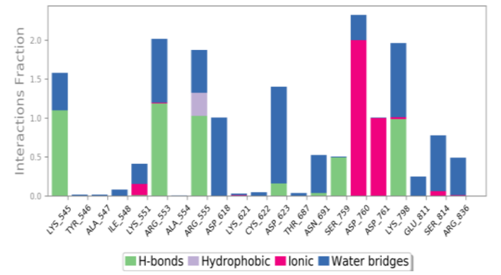

E:72

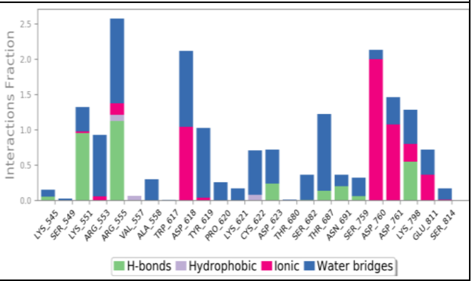

Figure S14. Ligand-protein contacts summary histogram of all NHC-TP analogue systems with ≥30% occurrences shown in MD simulations.

Ligand #	MM-GBSA (last 50 ns)
**E:63**	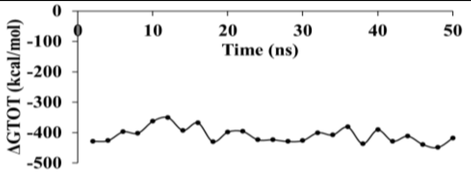
**E:12**	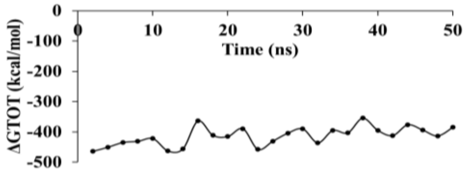
**E:66**	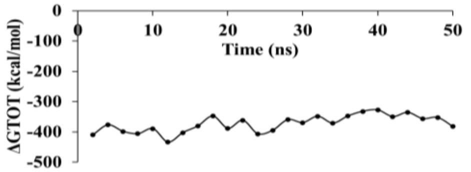
**E:65**	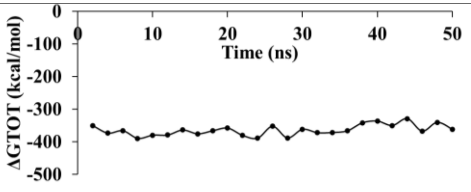
**E:53**	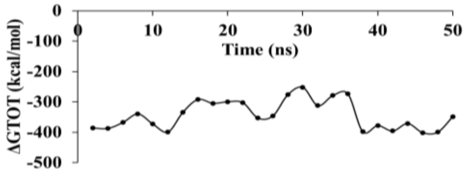
**E:48**	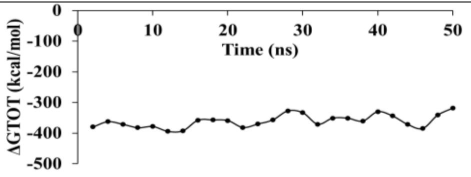
**E:03**	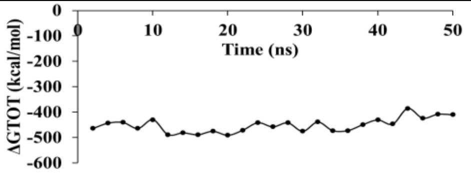

Figure S15. MM-GBSA binding free energy calculated between the top 7 ligands and the SARS-COV-2 RdRp active site for the last 50 ns of MD simulation (from 150 ns to 200 ns).

Entry	3D figure last frame	Transparent protein
E:03	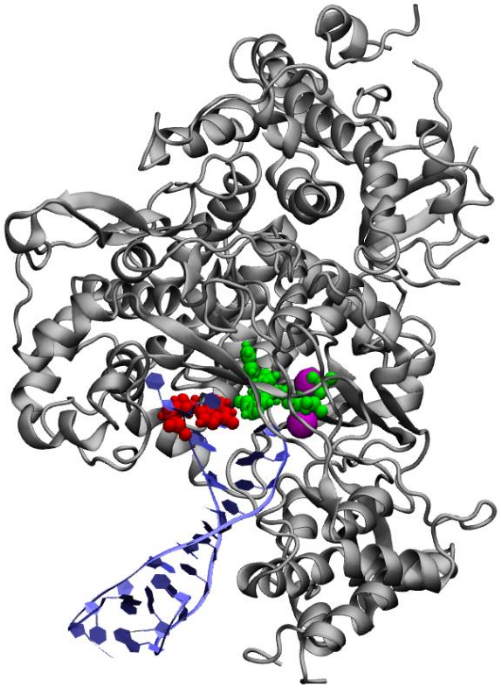	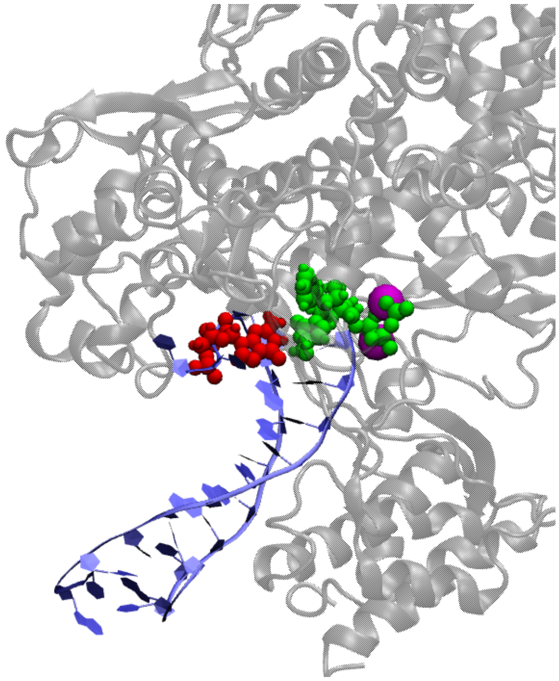
E:04	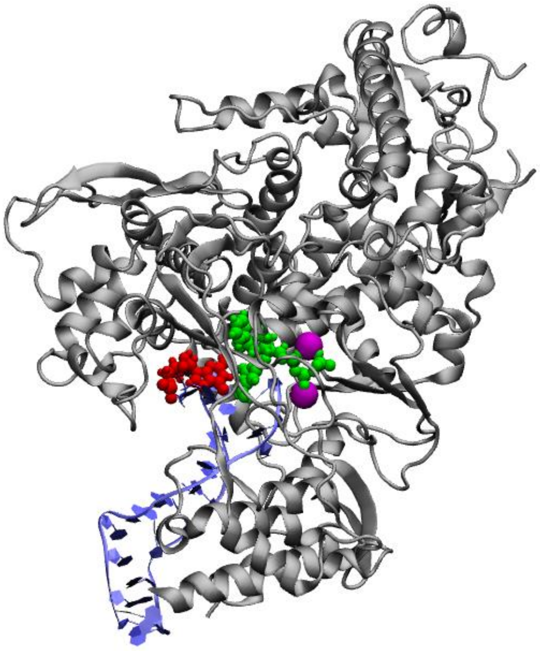	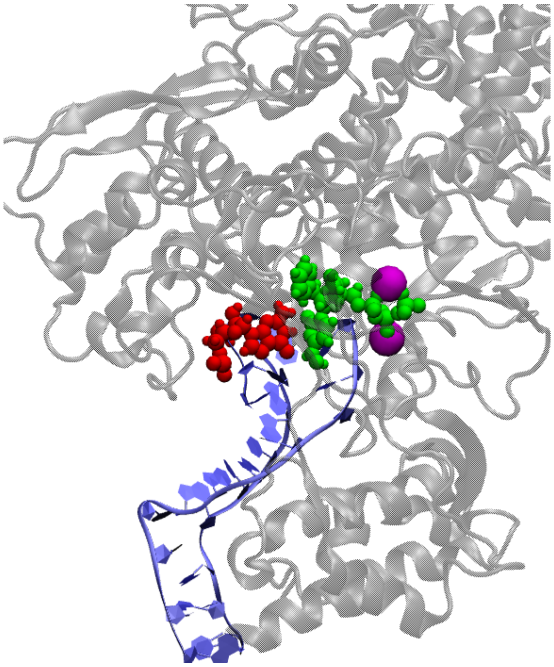

E:07	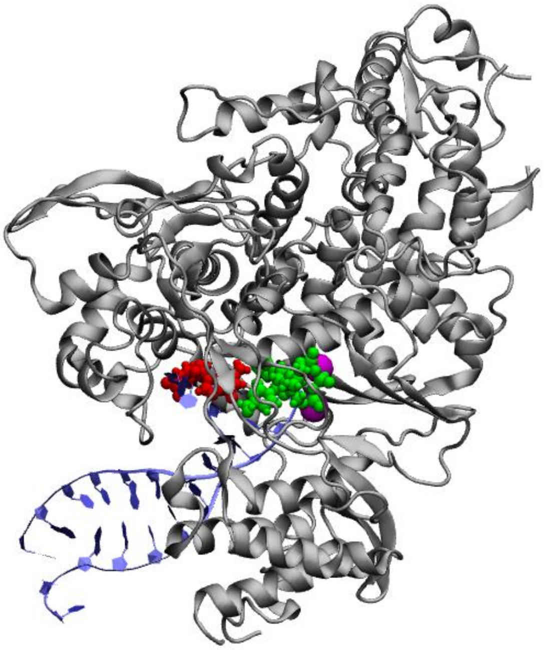	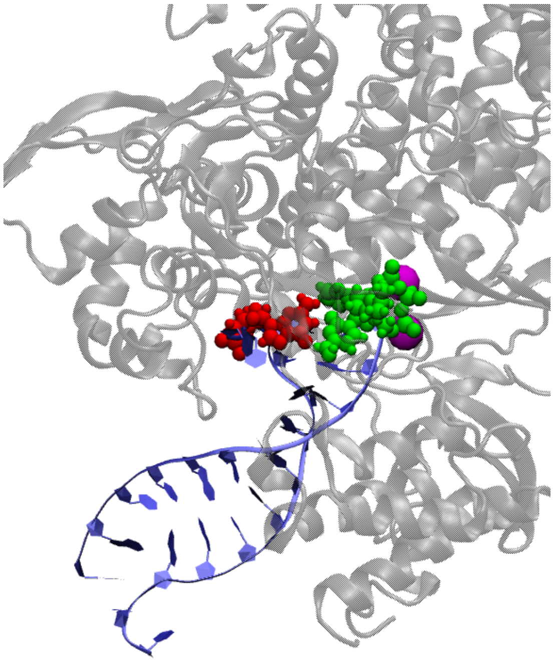
E:15	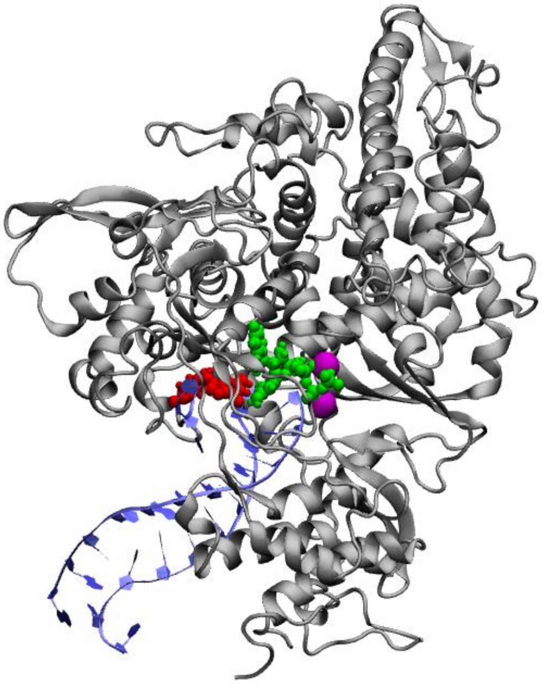	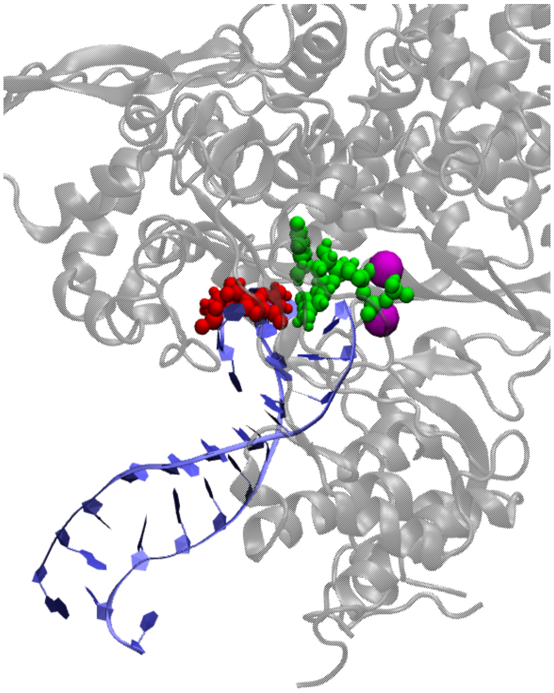

E:18	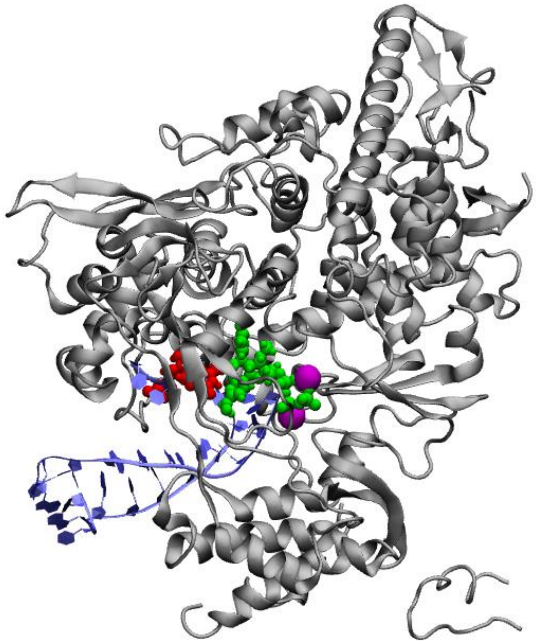	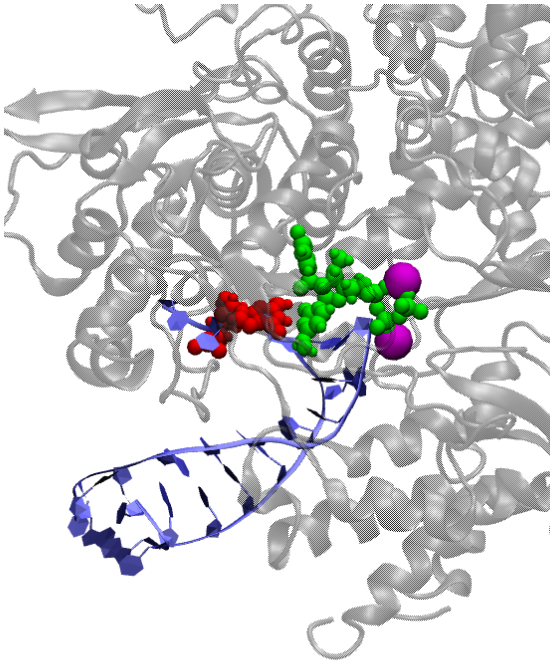
E:37	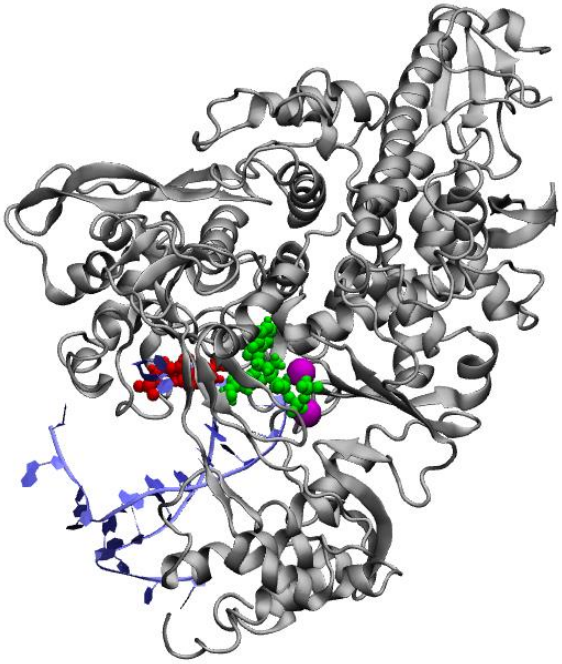	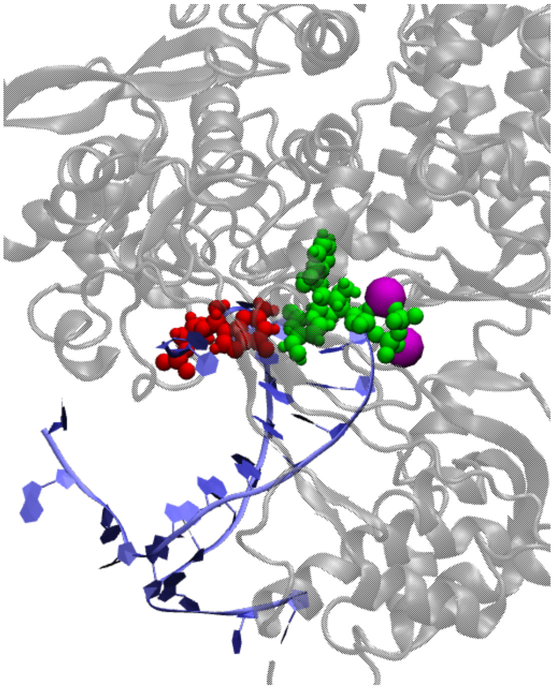

E:50	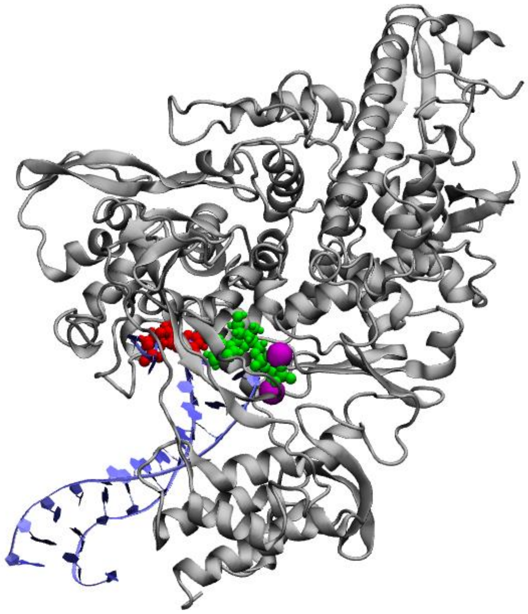	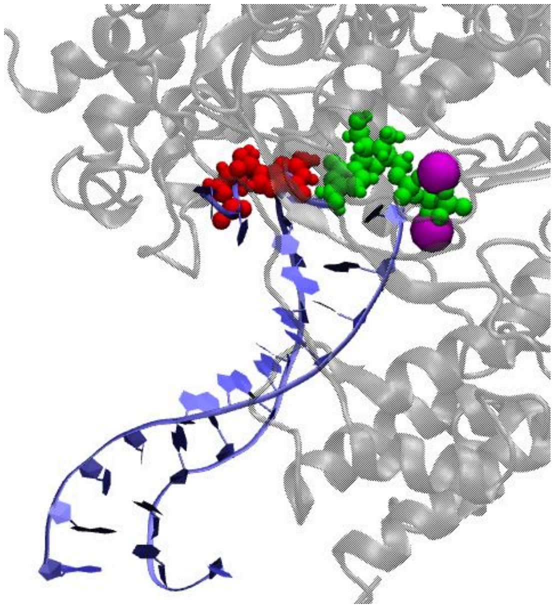
E:51	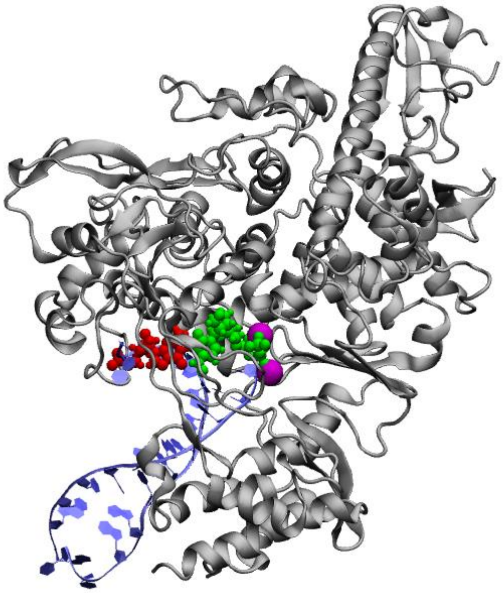	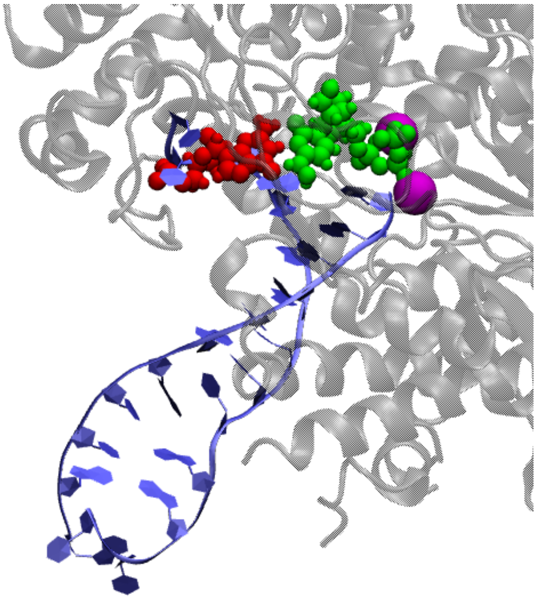

E:53	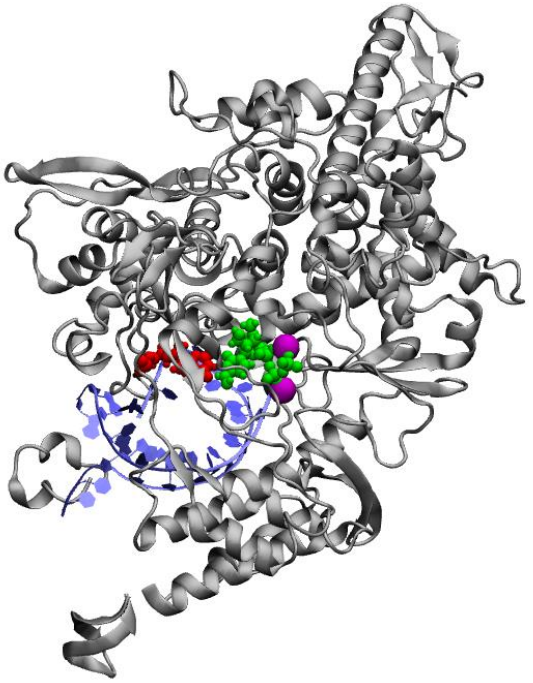	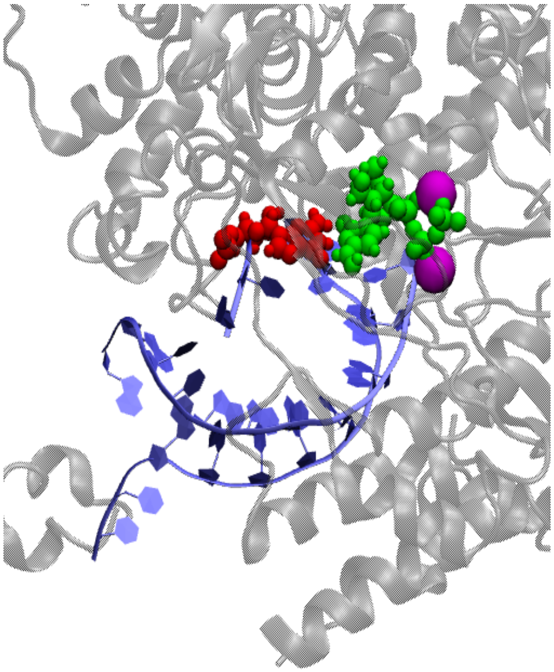
E:55	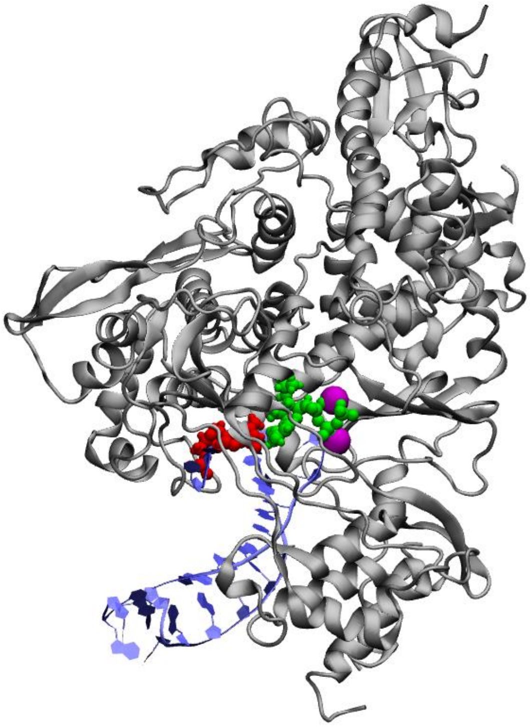	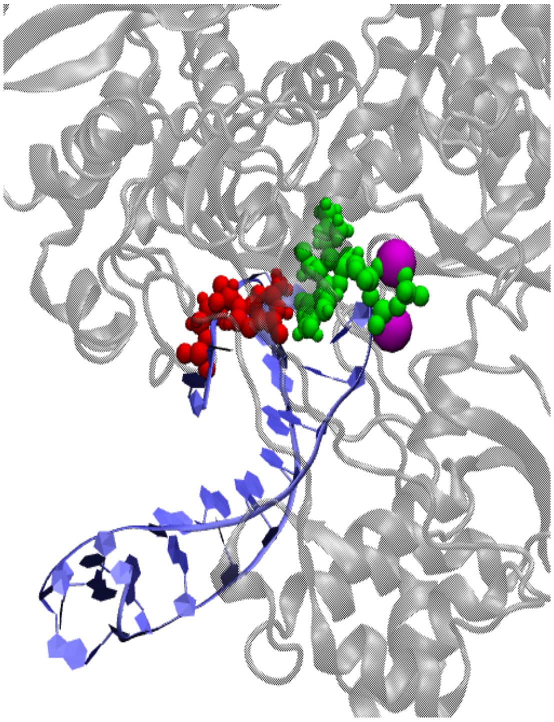

E:57	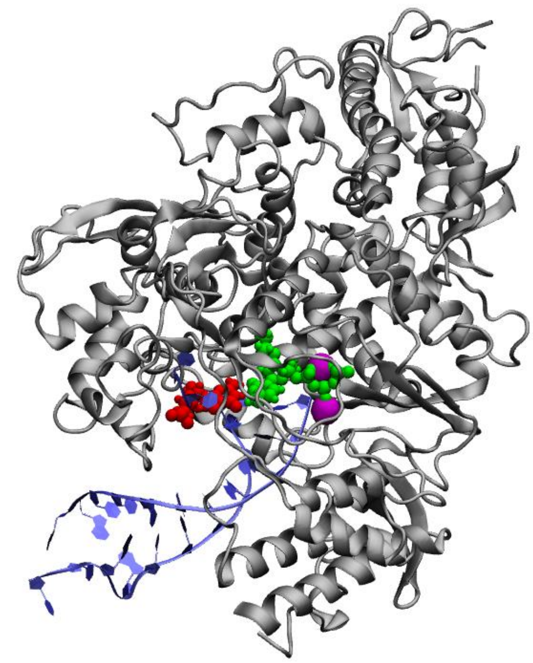	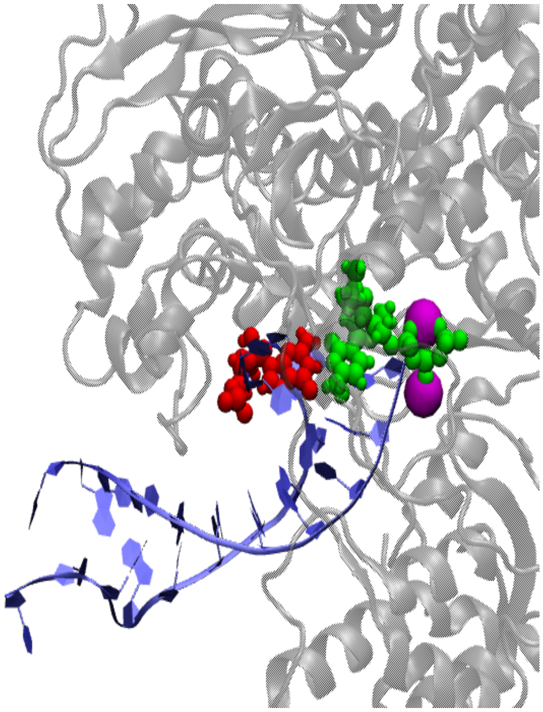
E:61	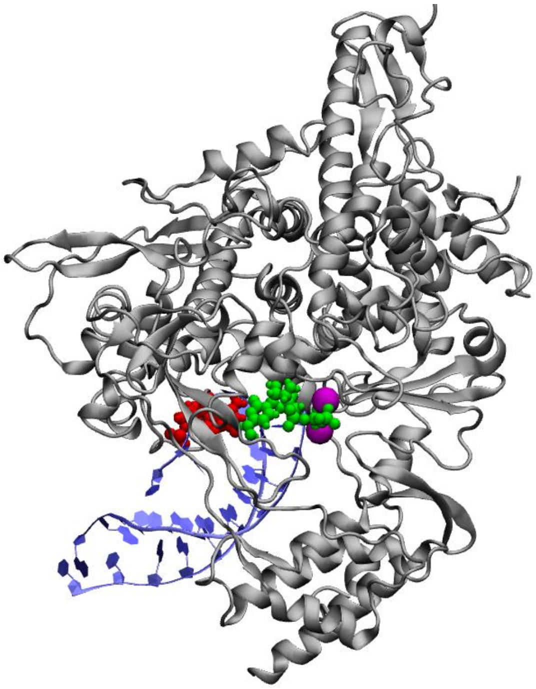	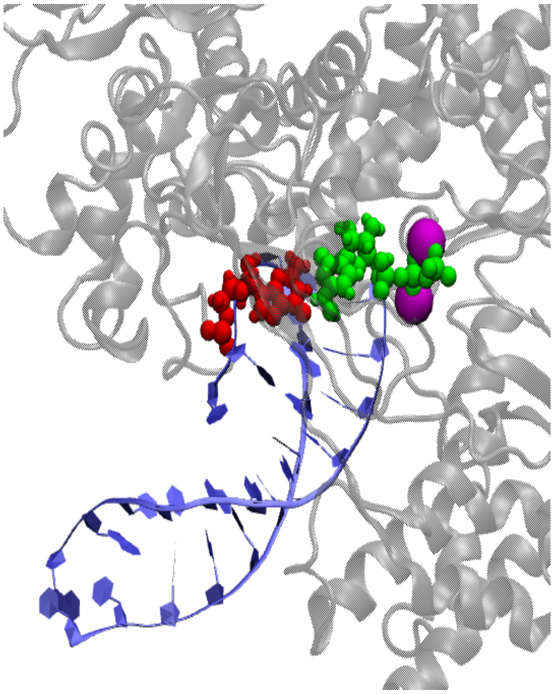

E:63	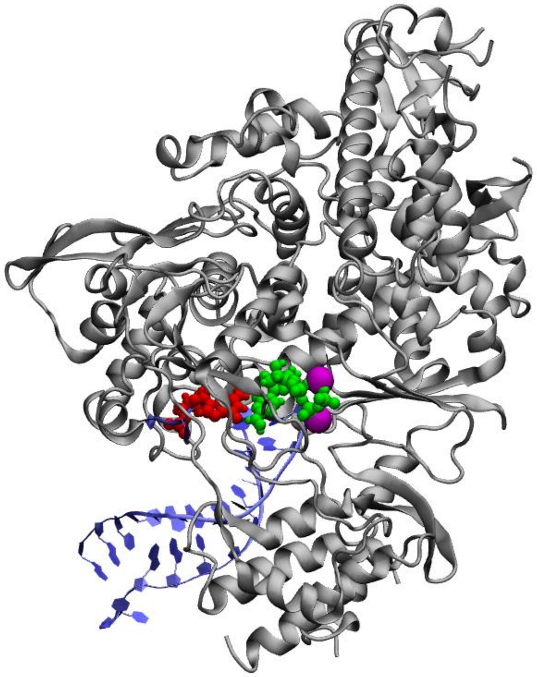	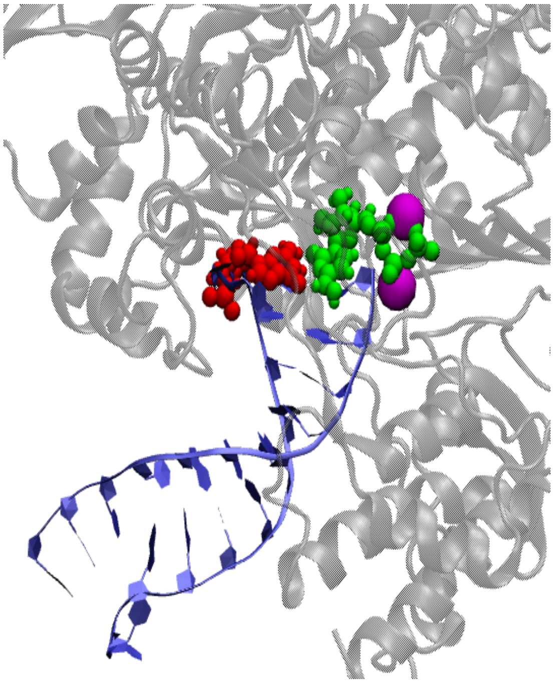
E:64	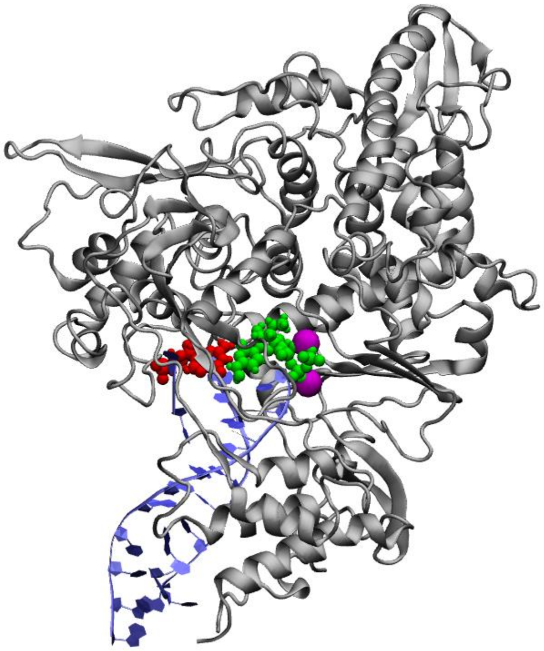	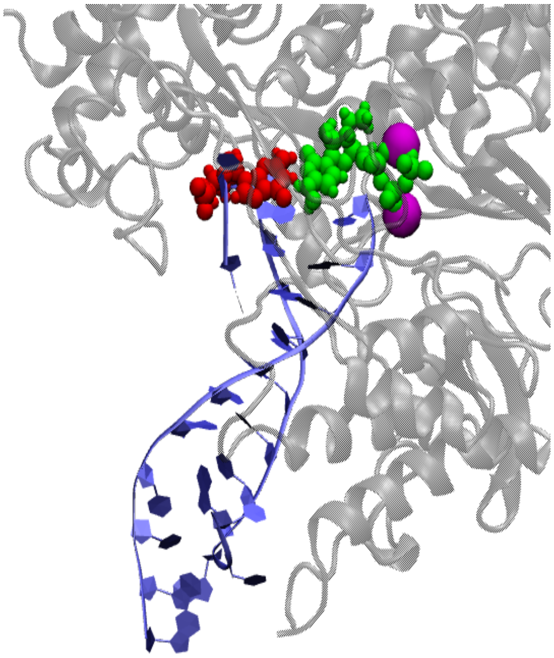

E:66	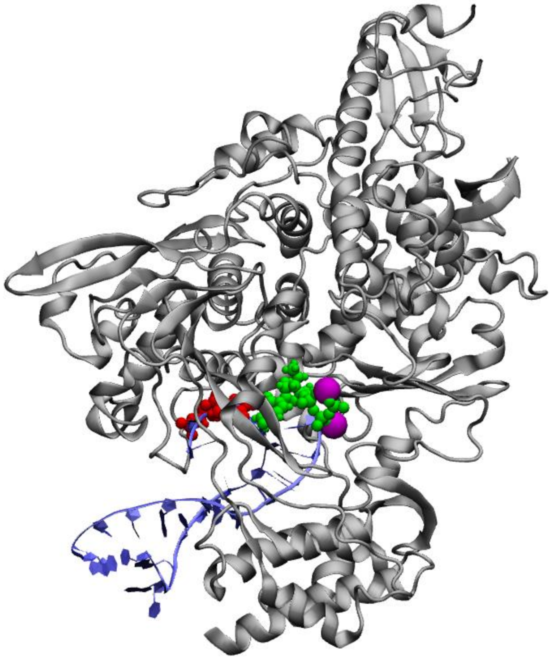	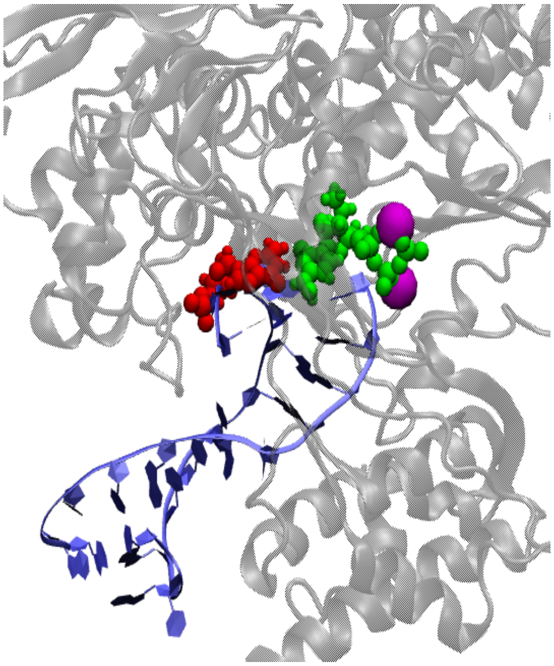
E:67	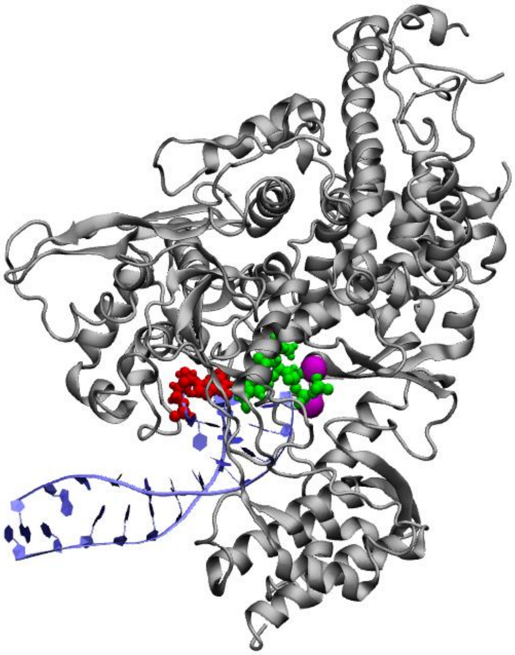	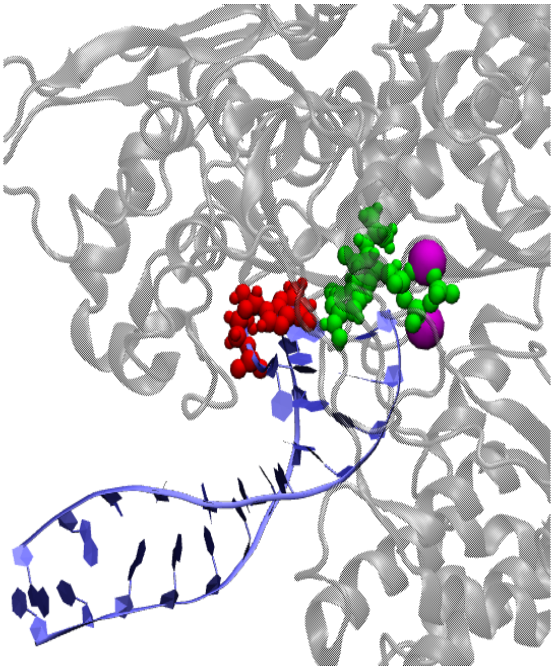

E:71	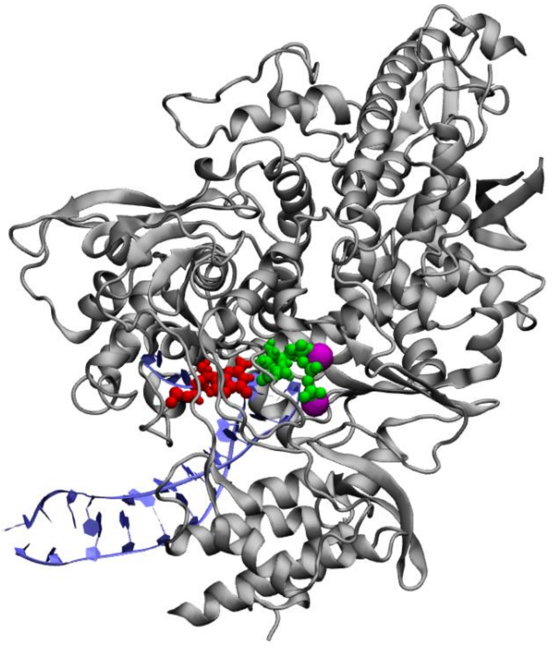	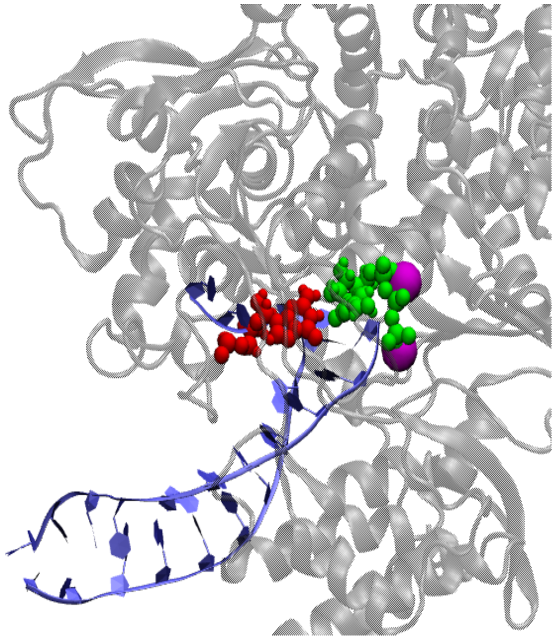

Figure S16. 3D structure of SARS-COV-2 RdRp (gray) with enumerated ligand (green) of the last snapshot of the MD simulations. RNA strands shown in blue and G10 mutation for hydrogen bonding shown in red. Mg^{2+} ions are shown as purple spheres**.**

A)

Atom#	X	Y	Z	Charge	RES	AtomName
1	28.289028	3.181765	-2.347406	0.205	E71	C1
2	28.531851	2.422004	-3.663144	0.48	E71	C2
3	28.496851	0.918891	-3.426023	-0.56	E71	N1
4	29.660475	0.166408	-3.343687	0.1	E71	C3
5	29.647387	-1.159478	-3.093355	-0.06	E71	C4
6	28.352165	-1.782518	-2.909424	0.46	E71	C5
7	28.248657	-3.077862	-2.559317	-0.32	E71	N2
8	29.345148	-3.850707	-2.143705	-0.5507	E71	O2
9	27.243423	0.276172	-3.276132	0.55	E71	C6
10	27.245338	-1.070562	-3.015381	-0.54	E71	N3
11	26.186522	0.902358	-3.318175	-0.48	E71	O3
12	29.806999	2.789781	-4.123128	-0.4	E71	O4
13	30.451307	3.705528	-3.22911	0.17	E71	C7
14	31.98184	3.428988	-3.189295	0.08	E71	C8
15	32.700138	4.102749	-2.14472	-0.61	E71	O5
16	33.201893	3.323163	-0.716437	1.3611	E71	P1
17	34.736389	2.708882	-1.085793	-0.5855	E71	O6
18	36.246677	3.317034	-1.614547	1.8411	E71	P2
19	36.059433	3.040377	-3.243145	-0.6633	E71	O7
20	36.564831	3.606485	-4.642325	1.8725	E71	P3
21	35.848248	2.832761	-5.743543	-1.11997	E71	O8
22	38.039028	3.32608	-4.75619	-1.11997	E71	O9
23	36.217678	5.073658	-4.633192	-1.11997	E71	O10
24	37.306244	2.46623	-1.040249	-1.058	E71	O11
25	36.248219	4.791953	-1.343227	-1.058	E71	O12
26	32.276016	2.218646	-0.389052	-0.97	E71	O13
27	33.44812	4.458613	0.230608	-0.97	E71	O14
28	29.70516	3.515576	-1.905809	0.205	E71	C9
29	27.770721	2.56431	-1.613169	0.06	E71	H2
30	27.776743	2.606036	-4.430246	0.1	E71	H3
31	30.627554	0.629468	-3.463948	0.1	E71	H4
32	30.586281	-1.688413	-3.013352	0.1	E71	H5
33	27.394451	-3.634459	-2.56435	0.37	E71	H6
34	29.466301	-3.657379	-1.227487	0.4507	E71	H7
35	32.422352	3.723819	-4.142232	0.06	E71	H9
36	32.164795	2.359386	-3.092146	0.06	E71	H10
37	30.101768	2.653459	-1.375134	0.06	E71	H11
38	27.537678	4.366059	-2.541924	-0.683	E71	O15
39	27.936926	5.008559	-1.966334	0.418	E71	H12
40	29.731409	4.666179	-1.072259	-0.683	E71	O16
41	29.939457	4.396173	-0.187034	0.418	E71	H13
42	30.270727	4.715638	-3.601593	0.03	E71	H14

B)

Atom#	X	Y	Z	Charge	RES	AtomName
1	28.121765	2.1142	-2.098344	-0.06	E03	C1
2	28.197458	1.466607	-3.503257	0.48	E03	C2
3	28.473515	-0.035514	-3.345816	-0.56	E03	N1
4	29.74218	-0.592397	-3.404176	0.1	E03	C3
5	30.01141	-1.851752	-2.999866	-0.06	E03	C4
6	28.890097	-2.650616	-2.559491	0.46	E03	C5
7	29.042559	-3.916155	-2.141734	-0.32	E03	N2
8	30.275137	-4.593213	-2.126832	-0.5507	E03	O2
9	27.393484	-0.867185	-2.977795	0.55	E03	C6
10	27.672758	-2.145203	-2.579329	-0.54	E03	N3
11	26.248219	-0.433746	-2.944014	-0.48	E03	O3
12	29.254742	2.080928	-4.155689	-0.4	E03	O4
13	30.087261	2.835393	-3.27778	0.2	E03	C7
14	31.514679	2.346736	-3.503902	0.08	E03	C8
15	32.42881	3.317698	-3.113469	-0.61	E03	O5
16	34.053165	3.174645	-3.473535	1.3611	E03	P1
17	34.630856	1.67501	-3.059618	-0.5855	E03	O6
18	34.91143	0.967708	-1.565926	1.8411	E03	P2
19	35.331161	2.240202	-0.522427	-0.6633	E03	O7
20	36.336311	3.416245	-0.027793	1.8725	E03	P3
21	37.084263	2.930564	1.206429	-1.11997	E03	O8
22	37.268326	3.765995	-1.161269	-1.11997	E03	O9
23	35.372837	4.574495	0.256739	-1.11997	E03	O10
24	33.609814	0.440325	-1.090322	-1.058	E03	O11
25	36.044113	0.037696	-1.710346	-1.058	E03	O12
26	34.302914	3.324921	-4.920606	-0.97	E03	O13
27	34.649387	4.123707	-2.543589	-0.97	E03	O14
28	29.588491	2.493935	-1.858131	0.12	E03	C9
29	27.763769	1.323445	-1.467628	0.06	E03	H2
30	27.298788	1.511744	-4.121975	0.1	E03	H3
31	30.568077	-0.027577	-3.788375	0.1	E03	H4
32	31.027296	-2.212551	-3.037182	0.1	E03	H5
33	28.262512	-4.56537	-2.101252	0.37	E03	H6
34	30.703949	-4.357433	-1.316288	0.4507	E03	H7
35	31.75565	1.456783	-2.929551	0.06	E03	H9
36	31.650175	2.104589	-4.54228	0.06	E03	H10
37	30.09164	1.578157	-1.543189	0.06	E03	H11
38	27.085245	3.240294	-1.879902	0.32	E03	C10
39	26.247082	3.382064	-2.9993	-0.683	E03	O15
40	26.310875	3.048734	-0.56554	-0.115	E03	C11
41	26.895254	2.461591	0.581629	-0.115	E03	C12
42	26.216614	2.397189	1.816736	-0.115	E03	C13
43	24.967651	3.025095	1.95036	-0.115	E03	C14
44	24.41917	3.703139	0.847253	-0.115	E03	C15
45	25.036261	3.617949	-0.414063	-0.115	E03	C16
46	27.582949	4.204303	-1.79507	0.06	E03	H12
47	25.453281	3.817273	-2.736571	0.418	E03	H13
48	27.915819	2.116215	0.566369	0.115	E03	H14
49	26.688284	1.939775	2.676231	0.115	E03	H15
50	24.463041	3.007471	2.901824	0.115	E03	H16
51	23.447227	4.123513	0.930278	0.115	E03	H17
52	24.539267	4.109554	-1.241642	0.115	E03	H18
53	29.863735	3.526092	-0.834917	-0.9	E03	N4
54	29.895563	4.459701	-1.250975	0.36	E03	H19
55	29.168459	3.532998	-0.083889	0.36	E03	H20
56	29.910959	4.308515	-3.7189	-0.18	E03	C17
57	30.499258	4.988276	-3.100592	0.06	E03	H21
58	30.250654	4.440289	-4.749469	0.06	E03	H22
59	28.87298	4.635021	-3.675509	0.06	E03	H23

C)

Atom#	X	Y	Z	Charge	RES	AtomName
1	27.704716	2.908648	-1.933015	-0.06	E12	C1
2	27.82509	2.490681	-3.436007	0.48	E12	C2
3	27.877867	0.976564	-3.642509	-0.56	E12	N1
4	26.762644	0.267877	-4.054981	0.1	E12	C3
5	26.67477	-1.076575	-3.925183	-0.06	E12	C4
6	27.847935	-1.765239	-3.443002	0.46	E12	C5
7	27.848141	-3.07702	-3.169623	-0.32	E12	N2
8	28.927986	-3.731301	-2.543643	-0.5507	E12	O2
9	29.042093	0.247318	-3.337855	0.55	E12	C6
10	28.955145	-1.100725	-3.24869	-0.54	E12	N3
11	30.098869	0.777481	-3.056604	-0.48	E12	O3
12	28.989222	3.057747	-3.94483	-0.4	E12	O4
13	29.542492	4.018214	-3.044575	0.2	E12	C7
14	30.835497	3.517751	-2.399572	0.08	E12	C8
15	31.858654	3.181757	-3.312018	-0.61	E12	O5
16	33.333591	2.704658	-2.663135	1.3611	E12	P1
17	34.481796	2.861182	-3.90651	-0.5855	E12	O6
18	36.155193	2.550585	-3.965783	1.8411	E12	P2
19	36.891075	3.519756	-2.720953	-0.6633	E12	O7
20	37.097801	3.82306	-1.085426	1.8725	E12	P3
21	37.35844	2.517941	-0.362979	-1.11997	E12	O8
22	38.280914	4.75713	-0.977696	-1.11997	E12	O9
23	35.848461	4.523089	-0.54384	-1.11997	E12	O10
24	36.341087	1.140302	-3.594333	-1.058	E12	O11
25	36.648277	2.923104	-5.343652	-1.058	E12	O12
26	33.627445	3.723097	-1.633472	-0.97	E12	O13
27	33.194172	1.300897	-2.24108	-0.97	E12	O14
28	28.506645	4.211941	-1.918553	0.205	E12	C9
29	28.247124	2.14317	-1.380348	0.06	E12	H2
30	27.002039	2.850026	-4.053385	0.1	E12	H3
31	25.890144	0.800934	-4.40275	0.1	E12	H4
32	25.738846	-1.570749	-4.139393	0.1	E12	H5
33	27.047302	-3.703479	-3.222133	0.37	E12	H6
34	29.449156	-4.085175	-3.253738	0.4507	E12	H7
35	31.211489	4.316648	-1.759616	0.06	E12	H9
36	30.658031	2.685215	-1.721674	0.06	E12	H10
37	28.982864	4.353788	-0.945065	0.06	E12	H11
38	26.22665	3.032662	-1.413437	0.32	E12	C10
39	25.455593	2.123366	-2.12746	-0.683	E12	O15
40	25.927469	2.840995	0.079572	-0.115	E12	C11
41	26.768589	2.123092	0.959541	-0.115	E12	C12
42	26.363537	1.839878	2.280224	-0.115	E12	C13
43	25.132917	2.318644	2.763082	-0.115	E12	C14
44	24.304691	3.073688	1.915575	-0.115	E12	C15
45	24.698484	3.323271	0.586367	-0.115	E12	C16
46	25.800615	3.994738	-1.692185	0.06	E12	H12
47	25.077999	2.573082	-2.868263	0.418	E12	H13
48	27.72942	1.755681	0.651072	0.115	E12	H14
49	27.013535	1.27578	2.933429	0.115	E12	H15
50	24.833904	2.115624	3.781356	0.115	E12	H16
51	23.362181	3.45311	2.280423	0.115	E12	H17
52	24.02947	3.873844	-0.05846	0.115	E12	H18
53	27.720982	5.366029	-2.143679	-0.683	E12	O17
54	28.301352	6.113203	-2.184988	0.418	E12	H19
55	29.88158	5.247949	-3.878172	-0.18	E12	C17
56	30.286238	6.060829	-3.276756	0.06	E12	H20
57	30.644241	4.925292	-4.582229	0.06	E12	H21
58	29.048258	5.623921	-4.457032	0.06	E12	H22

D)

Atom#	X	Y	Z	Charge	RES	AtomName
1	27.86693	2.887613	-1.771226	-0.06	E48	C1
2	27.815372	2.580532	-3.310063	0.48	E48	C2
3	27.785971	1.093892	-3.639762	-0.56	E48	N1
4	26.627737	0.527847	-4.135003	0.1	E48	C3
5	26.434525	-0.805477	-4.143674	-0.06	E48	C4
6	27.538889	-1.63285	-3.735788	0.46	E48	C5
7	27.446629	-2.962284	-3.74007	-0.32	E48	N2
8	28.248297	-3.776215	-2.932992	-0.5507	E48	O2
9	28.868626	0.23956	-3.34287	0.55	E48	C6
10	28.670723	-1.095843	-3.377454	-0.54	E48	N3
11	29.964727	0.635588	-3.014397	-0.48	E48	O3
12	28.902681	3.177837	-3.929849	-0.4	E48	O4
13	29.569897	4.042428	-3.018273	0.2	E48	C7
14	30.836655	3.29576	-2.646858	0.08	E48	C8
15	31.752222	4.066501	-1.941904	-0.61	E48	O5
16	33.081474	3.280037	-1.308811	1.3611	E48	P1
17	34.171051	3.528877	-2.631647	-0.5855	E48	O6
18	35.701996	2.953052	-3.199445	1.8411	E48	P2
19	36.735325	3.918389	-2.237241	-0.6633	E48	O7
20	37.153374	4.414396	-0.730537	1.8725	E48	P3
21	35.848503	4.926066	-0.143654	-1.11997	E48	O8
22	37.764622	3.272532	0.061444	-1.11997	E48	O9
23	38.092548	5.572059	-0.924293	-1.11997	E48	O10
24	35.854233	1.541526	-2.817071	-1.058	E48	O11
25	35.881348	3.30982	-4.643139	-1.058	E48	O12
26	33.448689	4.123189	-0.149336	-0.97	E48	O13
27	32.781796	1.851391	-1.115819	-0.97	E48	O14
28	28.625441	4.21473	-1.804828	0.205	E48	C9
29	28.497566	2.135837	-1.30686	0.06	E48	H2
30	26.932192	3.007175	-3.788534	0.1	E48	H3
31	25.798882	1.153702	-4.436601	0.1	E48	H4
32	25.469837	-1.184795	-4.432204	0.1	E48	H5
33	26.885277	-3.508105	-4.392919	0.37	E48	H6
34	27.614977	-4.278814	-2.426298	0.4507	E48	H7
35	30.588682	2.516056	-1.937135	0.06	E48	H9
36	31.326162	2.856368	-3.518471	0.06	E48	H10
37	29.18355	4.395872	-0.883514	0.06	E48	H11
38	26.445024	2.855737	-1.111136	0.32	E48	C10
39	25.848597	1.722624	-1.643451	-0.683	E48	O15
40	26.25001	2.71685	0.402542	-0.115	E48	C11
41	25.018549	3.117753	0.96462	0.22	E48	C12
42	24.686527	2.840748	2.305549	-0.115	E48	C13
43	25.58189	2.118458	3.106341	-0.115	E48	C14
44	26.819227	1.722107	2.572259	-0.115	E48	C15
45	27.165611	2.064381	1.248731	-0.115	E48	C16
46	24.106968	3.723731	0.186567	-0.22	E48	F1
47	25.865255	3.706748	-1.463518	0.06	E48	H12
48	25.724352	1.863744	-2.571898	0.418	E48	H13
49	23.733253	3.15169	2.708959	0.115	E48	H14
50	25.326033	1.876281	4.127301	0.115	E48	H15
51	27.522154	1.183393	3.188594	0.115	E48	H16
52	28.140215	1.767638	0.902696	0.115	E48	H17
53	27.762875	5.312494	-2.035547	-0.683	E48	O16
54	28.289717	6.022089	-2.389083	0.418	E48	H18
55	29.964071	5.308078	-3.772471	-0.18	E48	C17
56	30.52972	6.000627	-3.145296	0.06	E48	H19
57	30.623711	4.973085	-4.57289	0.06	E48	H20
58	29.125233	5.845385	-4.207289	0.06	E48	H21

Atom#	X	Y	Z	Charge	RES	AtomName
1	27.398237	2.125626	-3.54298	-0.06	E53	C1
2	28.42646	1.152209	-4.153688	0.48	E53	C2
3	28.614944	-0.167081	-3.432237	-0.56	E53	N1
4	29.896322	-0.676211	-3.235039	0.1	E53	C3
5	30.117496	-1.93213	-2.792761	-0.06	E53	C4
6	28.950235	-2.749301	-2.543748	0.46	E53	C5
7	29.038647	-4.027763	-2.143628	-0.32	E53	N2
8	30.238689	-4.754126	-2.14368	-0.5507	E53	O2
9	27.497854	-1.012914	-3.220737	0.55	E53	C6
10	27.741501	-2.26846	-2.743458	-0.54	E53	N3
11	26.340807	-0.656695	-3.436612	-0.48	E53	O3
12	29.631538	1.852479	-4.138194	-0.4	E53	O4
13	29.470634	3.217621	-3.77875	0.17	E53	C7
14	30.777315	3.690483	-3.156819	0.08	E53	C8
15	31.867146	3.02101	-3.743578	-0.61	E53	O5
16	33.246696	2.820228	-2.82627	1.3611	E53	P1
17	34.478127	2.560701	-3.960748	-0.5855	E53	O6
18	35.904987	3.39013	-4.354146	1.8411	E53	P2
19	36.727184	3.573674	-2.834899	-0.6633	E53	O7
20	36.648212	4.267481	-1.319235	1.8725	E53	P3
21	35.483425	3.702424	-0.543656	-1.11997	E53	O8
22	36.24826	5.722897	-1.487478	-1.11997	E53	O9
23	37.959694	4.083047	-0.593622	-1.11997	E53	O10
24	35.535011	4.734377	-4.828047	-1.058	E53	O11
25	36.648304	2.52427	-5.343666	-1.058	E53	O12
26	33.040718	1.593479	-2.022662	-0.97	E53	O13
27	33.448154	4.123102	-2.143624	-0.97	E53	O14
28	28.294182	3.124305	-2.808352	0.14	E53	C9
29	26.740419	1.65175	-2.817701	0.06	E53	H2
30	28.189619	0.82166	-5.165794	0.1	E53	H3
31	30.774031	-0.080018	-3.444424	0.1	E53	H4
32	31.132986	-2.278524	-2.663661	0.1	E53	H5
33	28.226202	-4.638082	-2.143596	0.37	E53	H6
34	30.367989	-5.026923	-3.044466	0.4507	E53	H7
35	30.778463	3.486924	-2.085179	0.06	E53	H9
36	30.91507	4.763761	-3.285392	0.06	E53	H10
37	28.645191	2.645314	-1.89298	0.06	E53	H11
38	26.554546	2.731265	-4.693711	0.145	E53	C10
39	27.191166	2.912102	-5.94628	-0.683	E53	O15
40	25.665993	2.122802	-4.860433	0.06	E53	H12
41	26.248243	3.722743	-4.394723	0.06	E53	H13
42	26.630615	3.434118	-6.502306	0.418	E53	H14
43	27.608137	4.383702	-2.459198	-0.78	E53	N4
44	28.449389	5.415627	-1.855666	0.02	E53	C11
45	26.861776	4.157069	-1.808722	0.38	E53	H15
46	27.837999	6.232542	-1.472967	0.06	E53	H16
47	29.035757	5.021573	-1.023977	0.06	E53	H17
48	29.132153	5.848736	-2.585765	0.06	E53	H18
49	29.240366	3.802871	-4.668905	0.03	E53	H19

E)

F)

Atom#	X	Y	Z	Charge	RES	AtomName
1	27.295422	2.533121	-2.850427	0.205	E63	C1
2	27.896011	1.6262	-3.947937	0.48	E63	C2
3	28.176378	0.223328	-3.455141	-0.56	E63	N1
4	29.465891	-0.294542	-3.350902	0.1	E63	C3
5	29.708099	-1.559976	-2.944567	-0.06	E63	C4
6	28.551941	-2.370928	-2.631186	0.46	E63	C5
7	28.648422	-3.643892	-2.210706	-0.32	E63	N2
8	29.848646	-4.364407	-2.143786	-0.5507	E63	O2
9	27.072346	-0.614356	-3.20326	0.55	E63	C6
10	27.337486	-1.87807	-2.765145	-0.54	E63	N3
11	25.91395	-0.226898	-3.343119	-0.48	E63	O3
12	29.092587	2.198295	-4.352843	-0.4	E63	O4
13	29.497017	3.291779	-3.543088	0.2	E63	C7
14	30.831255	3.001525	-2.879538	0.08	E63	C8
15	31.930937	2.919811	-3.743755	-0.61	E63	O5
16	33.439137	2.923342	-3.013194	1.3611	E63	P1
17	34.553391	3.090214	-4.289421	-0.5855	E63	O6
18	35.911293	4.058756	-4.657952	1.8411	E63	P2
19	36.851151	4.143437	-3.218905	-0.6633	E63	O7
20	36.865086	4.749439	-1.687939	1.8725	E63	P3
21	36.248257	6.122982	-1.74332	-1.11997	E63	O8
22	38.255398	4.69724	-1.095863	-1.11997	E63	O9
23	35.848221	4.032135	-0.845271	-1.11997	E63	O10
24	35.45224	5.42116	-4.978725	-1.058	E63	O11
25	36.647461	3.322963	-5.74357	-1.058	E63	O12
26	33.579952	1.589383	-2.392944	-0.97	E63	O13
27	33.448322	4.123374	-2.143047	-0.97	E63	O14
28	28.45953	3.398865	-2.410237	0.12	E63	C9
29	26.903732	1.946603	-2.019071	0.06	E63	H2
30	27.242157	1.481143	-4.810828	0.1	E63	H3
31	30.329979	0.29625	-3.613422	0.1	E63	H4
32	30.727161	-1.912849	-2.874332	0.1	E63	H5
33	27.832344	-4.254419	-2.145632	0.37	E63	H6
34	30.001417	-4.680812	-3.027389	0.4507	E63	H7
35	30.792229	2.085901	-2.289875	0.06	E63	H9
36	31.015339	3.804203	-2.164848	0.06	E63	H10
37	28.850868	2.895231	-1.524968	0.06	E63	H11
38	26.257656	3.346309	-3.342295	-0.683	E63	O15
39	26.510002	3.662291	-4.195431	0.418	E63	H12
40	28.099457	4.776015	-2.011698	-0.9	E63	N4
41	28.303026	5.435505	-2.762163	0.36	E63	H13
42	27.098833	4.816882	-1.819058	0.36	E63	H14
43	29.572281	4.470214	-4.519957	-0.18	E63	C10
44	29.775686	5.422431	-4.02897	0.06	E63	H15
45	30.361391	4.303892	-5.253307	0.06	E63	H16
46	28.648258	4.524476	-5.08304	0.06	E63	H17

G)

Atom#	X	Y	Z	Charge	RES	AtomName
1	27.361195	3.145279	-2.740321	0.205	E65	C1
2	27.564631	2.286038	-4.009218	0.48	E65	C2
3	27.807489	0.841627	-3.636798	-0.56	E65	N1
4	29.084805	0.303368	-3.562955	0.1	E65	C3
5	29.307045	-0.989927	-3.247478	-0.06	E65	C4
6	28.143105	-1.794414	-2.943561	0.46	E65	C5
7	28.248096	-3.082325	-2.574914	-0.32	E65	N2
8	29.468582	-3.753496	-2.405731	-0.5507	E65	O2
9	26.692888	0.034385	-3.34056	0.55	E65	C6
10	26.934559	-1.266709	-2.99445	-0.54	E65	N3
11	25.551323	0.491624	-3.343813	-0.48	E65	O3
12	28.687181	2.769825	-4.691245	-0.4	E65	O4
13	29.25218	3.86966	-3.987432	0.17	E65	C7
14	30.769802	3.952948	-4.13525	0.08	E65	C8
15	31.420074	2.923963	-3.4173	-0.61	E65	O5
16	32.94632	3.13416	-2.688281	1.3611	E65	P1
17	34.063587	2.926068	-3.972159	-0.5855	E65	O6
18	35.628784	3.502402	-4.392652	1.8411	E65	P2
19	36.580204	3.586485	-2.970708	-0.6633	E65	O7
20	36.643219	4.258311	-1.482498	1.8725	E65	P3
21	36.258354	5.723033	-1.606805	-1.11997	E65	O8
22	37.955402	3.972769	-0.78983	-1.11997	E65	O9
23	35.448257	3.73741	-0.717948	-1.11997	E65	O10
24	35.475143	4.880621	-4.889029	-1.058	E65	O11
25	36.251587	2.523041	-5.343661	-1.058	E65	O12
26	33.079838	1.99735	-1.752472	-0.97	E65	O13
27	33.048168	4.522939	-2.143326	-0.97	E65	O14
28	28.770466	3.699274	-2.553658	0.14	E65	C9
29	27.069748	2.525066	-1.892281	0.06	E65	H2
30	26.704552	2.272695	-4.682631	0.1	E65	H3
31	29.953074	0.916583	-3.744278	0.1	E65	H4
32	30.322458	-1.356747	-3.208356	0.1	E65	H5
33	27.465164	-3.726944	-2.486444	0.37	E65	H6
34	29.648594	-4.168933	-3.241632	0.4507	E65	H7
35	31.07023	4.922726	-3.742768	0.06	E65	H9
36	31.045174	3.905622	-5.182685	0.06	E65	H10
37	29.346943	2.886518	-2.110676	0.06	E65	H11
38	26.339588	4.109869	-2.942224	-0.683	E65	O15
39	25.522726	3.653073	-3.074635	0.418	E65	H12
40	28.935526	4.874501	-1.684749	-0.78	E65	4
41	28.483469	6.177545	-2.171819	0.02	E65	C10
42	29.923529	4.944837	-1.455059	0.38	E65	H13
43	28.63224	6.92264	-1.394924	0.06	E65	H14
44	29.051476	6.502974	-3.044369	0.06	E65	H15
45	27.425543	6.196965	-2.419187	0.06	E65	H16
46	28.819691	4.760131	-4.432784	0.03	E65	H17

H)

Atom#	X	Y	Z	Charge	RES	AtomName
1	27.764765	3.321969	-2.107522	0.205	E66	C1
2	27.705109	2.699224	-3.505557	0.48	E66	C2
3	27.88875	1.186121	-3.406523	-0.56	E66	N1
4	29.120794	0.571831	-3.552317	0.1	E66	C3
5	29.314449	-0.748964	-3.343665	-0.06	E66	C4
6	28.153742	-1.52955	-2.9714	0.46	E66	C5
7	28.243271	-2.842262	-2.681574	-0.32	E66	N2
8	29.453903	-3.536734	-2.54362	-0.5507	E66	O2
9	26.7703	0.390317	-3.083506	0.55	E66	C6
10	26.9701	-0.95123	-2.876698	-0.54	E66	N3
11	25.659098	0.887619	-2.943729	-0.48	E66	O3
12	28.740957	3.282132	-4.251001	-0.4	E66	O4
13	29.635254	4.0661	-3.43865	0.2	E66	C7
14	31.050081	3.680427	-3.89025	0.08	E66	C8
15	31.599621	2.640825	-3.128218	-0.61	E66	O5
16	33.181133	2.716354	-2.554319	1.3611	E66	P1
17	34.081364	2.404189	-3.949623	-0.5855	E66	O6
18	35.735931	2.502995	-4.301555	1.8411	E66	P2
19	36.340569	3.728644	-3.248098	-0.6633	E66	O7
20	36.648216	4.265983	-1.710706	1.8725	E66	P3
21	35.645435	3.69783	-0.733844	-1.11997	E66	O8
22	36.248219	5.72309	-1.743655	-1.11997	E66	O9
23	38.079464	4.030958	-1.302411	-1.11997	E66	O10
24	35.848209	2.923084	-5.743655	-1.058	E66	O11
25	36.382198	1.227976	-3.957452	-1.058	E66	O12
26	33.315292	1.617823	-1.573729	-0.97	E66	O13
27	33.448143	4.123107	-2.143651	-0.97	E66	O14
28	29.242928	3.679528	-1.986086	0.14	E66	C9
29	27.451202	2.612095	-1.341465	0.06	E66	H2
30	26.752754	2.844075	-4.01911	0.1	E66	H3
31	29.978155	1.148428	-3.834288	0.1	E66	H4
32	30.308975	-1.159711	-3.444509	0.1	E66	H5
33	27.452763	-3.46506	-2.543996	0.37	E66	H6
34	29.594877	-3.970761	-3.378491	0.4507	E66	H7
35	31.719067	4.523068	-3.798675	0.06	E66	H9
36	31.048008	3.418711	-4.943645	0.06	E66	H10
37	29.756348	2.759169	-1.70837	0.06	E66	H11
38	26.93853	4.468723	-2.015414	-0.683	E66	O15
39	27.447634	5.108195	-1.53313	0.418	E66	H12
40	29.47571	4.692728	-0.944222	-0.78	E66	N4
41	30.881098	4.9491	-0.666535	0.02	E66	C10
42	29.048773	4.376733	-0.076997	0.38	E66	H13
43	30.989145	5.670506	0.143293	0.06	E66	H14
44	31.393208	4.034706	-0.364646	0.06	E66	H15
45	31.387302	5.352875	-1.540972	0.06	E66	H16
46	29.442642	5.566127	-3.756327	-0.18	E66	C11
47	30.241648	6.174024	-3.331626	0.06	E66	H17
48	29.448294	5.745522	-4.831429	0.06	E66	H18
49	28.519833	5.963724	-3.343638	0.06	E66	H19

Figure S17. OPLS3e Force field parameters for (**A**) NHC-TP/E:71, (**B**) E:03, (**C**) E:12, (**D**) E:48, (**E**) E:53, (**F**) E:63, (**G**) E:65, (**H**) E:66.
